# Hexacoordinate Actinide(IV) Compounds: Benchmark Complexes for Magnetism and Electronic Structure Analysis

**DOI:** 10.1021/jacs.6c09492

**Published:** 2026-08-28

**Authors:** Till Sawallisch, Dennis Grödler, Adrian Näder, Clara L. Silva, Peter Kaden, Michael Patzschke, Lucia Amidani, Kristina O. Kvashnina, Solveig Pospiech, Jan van Leusen, Moritz Schmidt, Thorsten Stumpf, Robert Gericke

**Affiliations:** † 28414 Helmholtz-Zentrum Dresden − Rossendorf, Institute of Resource Ecology, Bautzner Landstraße 400, Dresden 01328, Germany; ‡ The Rossendorf Beamline at ESRF − The European Synchrotron, CS40220, Grenoble Cedex 9 38043, France; § RWTH Aachen University, Institute of Inorganic Chemistry, Landoltweg 1, Aachen 52074, Germany; ∥ Brandenburg University of Technology Cottbus-Senftenberg, Institute of Materials Chemistry, Senftenberg 01968, Germany

## Abstract

Two series of actinide tetrachloride complexes with the monodentate carboxylic amide ligands phthalimidine (H-phthal) and 2-pyridone (HPyO) of the general formula [An^IV^Cl_4_(L)_2_] (An = U, Np, Pu) were synthesized and characterized in the solid state and in solution. SC-XRD analysis revealed an octahedral coordination environment with *trans* orientation of either H-phthal or HPyO in all cases, with and without THF molecules hydrogen-bonded to the NH group of the ligands. In the case of [NpCl_4_(H-phthal)_2_], the absence of THF leads to a significant distortion of the coordination geometry around the metal, including the shortest Np–Cl bonds observed so far (2.4394(8) Å). A quantum chemical bonding analysis (QTAIM) showed that the main bonding interaction is found between the actinide and chloride ligands, with the amides rather loosely bound. Despite their unusually low coordination number, the complexes were found to be stable, even in coordinating solvents. This allowed for an in-depth analysis of their ^1^H and ^13^C NMR spectroscopy. Due to their *pseudo* *D*_4h_ symmetry in the first coordination sphere, these complexes are excellent model systems to analyze their electronic structure and magnetic properties. We applied SQUID magnetometry to extract magnetic properties (μ_eff_) and crystal field parameters ( $B_{q}^{k}$ , Wybourne) in combination with quantum chemical calculations, which allows for the comprehensive description of the electronic ground state from an experimental and theoretical point of view.

## Graphical abstract

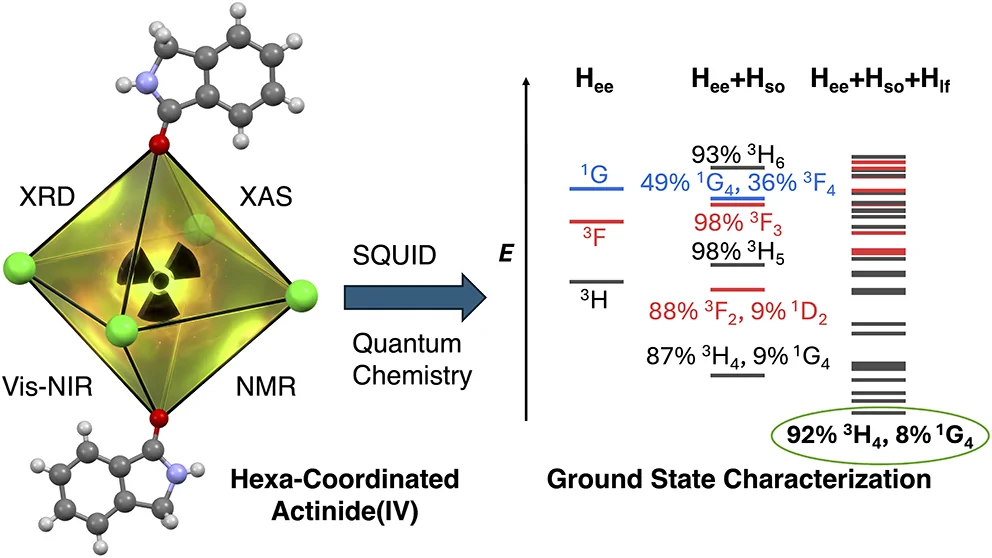

## Introduction

The actinides are among the elements with the largest atomic radii in the periodic table. As such, they prefer high coordination numbers like 7, 8 and even above.^1^ Lower coordination numbers usually require bulky ligands which do not allow the coordination of further ligands, especially in their lower oxidation states where no actinyl (AnO_2_^*n*+^) ions are formed. Complexes with smaller ligands usually involve coordinating solvent molecules to fill the coordination sphere of the metal.

However, hexacoordinated metal centers are especially interesting due to the high symmetry of an octahedral environment. The synthesis of an isostructural series of highly symmetric actinide complexes, including the transuranium elements neptunium and plutonium, can offer fascinating insights into the fundamental properties of these still underexplored heavy elements. Especially SQUID magnetometry is a useful tool in this regard, as it allows deep insights into the electronic structure of the actinides, which is influenced by factors such as electron–electron interactions, spin–orbit coupling, and the ligand field.^2^ For the actinides, all of these influences are of similar magnitude. While the former two are accessible through quantum chemical calculations, the ligand field interaction, evaluated in terms of ligand field parameters, $B_{q}^{k}$ , requires an experimental approach. That these electronic structure parameters have “real-world” implications, is obvious when considering recent developments for the extraction and separation of actinides,^3,4^ or in the development of ligands for the removal of actinides from the human body.^5^ These case studies show that selective actinide bonding relies on variable covalent contributions. Hence, an accurate description of chemical bonding is essential to designing tailored ligand systems, which, in turn, is only possible on the basis of a correct description of their electronic ground state, including the ligand field parameters $B_{q}^{k}$ . Unfortunately, these are only available for the actinides in a few high-symmetry systems to this date. Since the number of $B_{q}^{k}$ values depend on the symmetry around the metal ion (up to 27 for point group *C*_1_), the necessary fitting procedure is greatly facilitated by symmetric molecules. Indeed, for octahedral complexes, point group *O*_h_, only two independent parameters are necessary, which increases to five for *D*_4h_ symmetry. Ligand field studies of tetravalent actinides in octahedral coordination environment have focused mainly on [UX_6_]^2–^ compounds (X = F, Cl, Br, or I) with slightly different outcomes depending on the method,^6–10^ while studies on the heavier actinides Np and Pu remain scarce,^11^ especially in the case of reduced symmetry (*D*_4h_).

Actinide complexes of the general composition [An^IV^X_4_(L)_2_] (X = halogen; L = monodentate oxygen donor ligand) underwent an interesting development over the last 60 years. With the first studies dating from the 1960s and mainly relying on elemental analysis as well as IR data for characterization, the first crystal structure was reported in 1971,^12^ followed by several others reaching a maximum in the mid to late 1980s. After that, such complexes have occurred rarely in the literature, before gaining more attention fairly recently.^13–15^ The ligands with crystallographically characterized complexes are summarized in Chart [Fig chart1]. The vast number of publications from the early, noncrystallographic research in this area makes an overview about the existing data difficult, and so far, just a few systematic series of complexes which vary either the metal or the halide ligand have emerged, although a general trend for the donor strength of neutral ligands toward uranium­(IV) could be determined as As=O > P=O > S=O ∼ C=O.^16^

**1 chart1:**
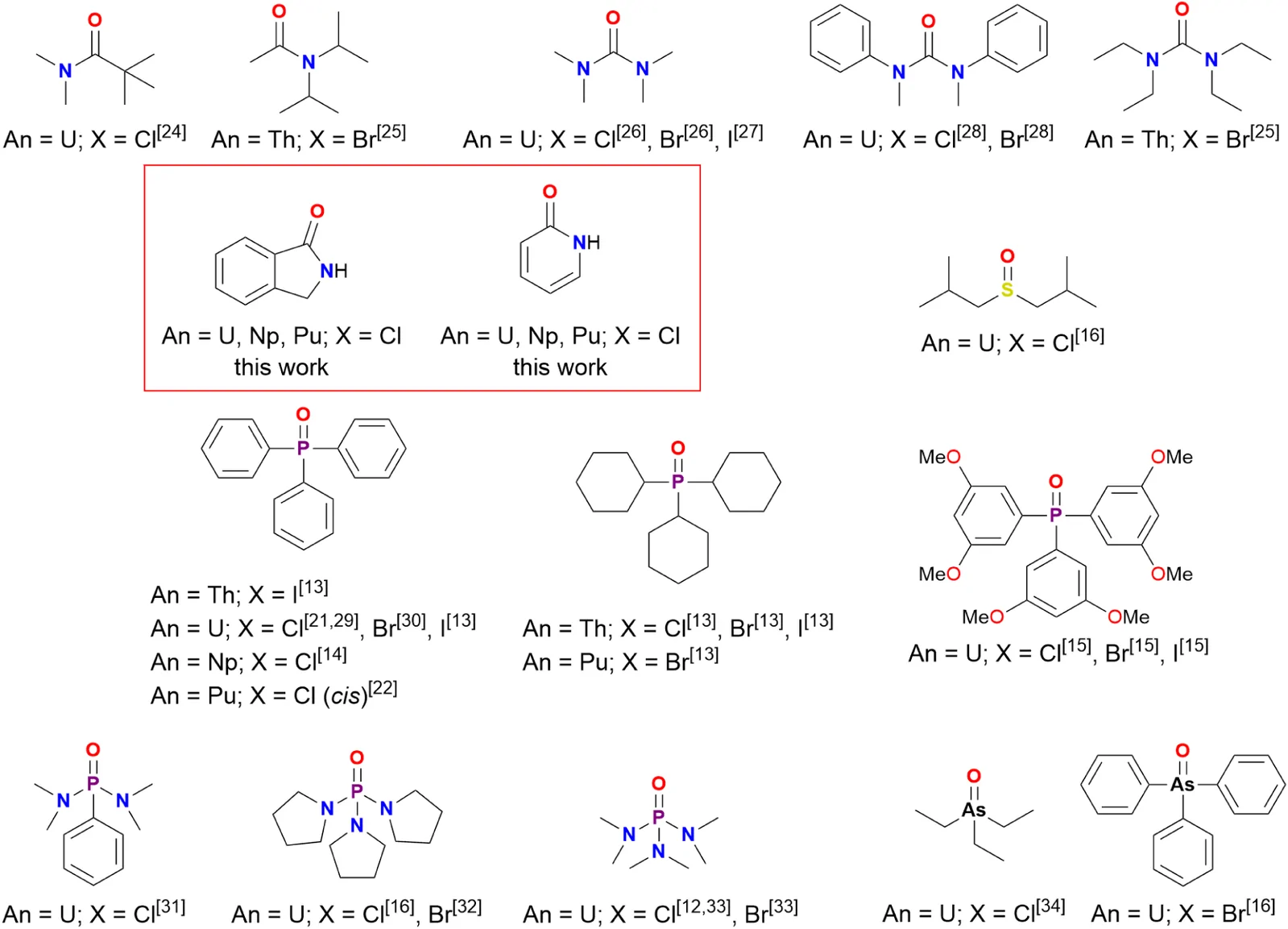
Literature Overview of Monodentate Oxygen Donor Ligands Used to Form Complexes of the Type [AnX_4_(L)_2_], Which Have Been Characterized Crystallographically^a^ ^,^ ^12–16,21,22,24–34^

a Ligands used in this study are shown in the red frame.

Besides the well-known octahedral anions of the type [An^IV^Cl_6_]^2–^, which have been characterized with a wide variety of counter cations,^17–20^ one series that was crystallographically characterized is [UX_4_(tmu)_2_] (X = Cl, Br, I; tmu = tetramethylurea) while for some other substituted ureas and phosphoramides, uranium tetrachloride and tetrabromide complexes have been characterized and compared (Chart [Fig chart1]). As a consequence of the above-mentioned ranking, omitting the arsenic compounds, the most complete series of [AnX_4_(L)_2_] complexes are based on phosphine oxide ligands. These include [UX_4_(OPPh_3_)_2_] (X = Cl,^21^ Br, I); [ThX_4_(OPCy_3_)_2_] (X = Cl, Br, I); and [AnCl_4_(OPPh_3_)_2_] (An = U, Np,^14^ Pu^22^). In this latter series, however, plutonium was found to form an octahedral complex with a *cis* arrangement while for uranium both *cis* and *trans* complexes are known. The larger thorium ion, on the other hand, forms a 1:3 complex with triphenylphosphine oxide.^23^ These findings already highlight the difficulties associated with the synthesis of isostructural complexes of the early actinides, which exhibit a similar but clearly not identical chemical behavior.

Here, we present two series of isostructural actinide complexes with hexacoordinated actinide centers of the type [An^IV^Cl_4_(L)_2_] (An = U, Np, Pu), with two sterically undemanding ligands L = 2-pyridone and phthalimidine. In order to gain deeper insight in the electronic structure of octahedral coordinated An^IV^, these compounds were thoroughly characterized in solid state using SC-XRD, SQUID magnetometry, X-ray absorption spectroscopy, as well as in solution using paramagnetic NMR spectroscopy. Our experimental findings are supported and compared with quantum chemical calculations, including an in-depth analysis of the ligand field parameters determined by combining experimental SQUID measurements and energy levels of spin–orbit coupling states (SOC states).

## Results and Discussion

### Synthesis and Structural Characterization

The [An^IV^Cl_4_(L)_2_]·2THF (An = U, Np, Pu) complexes were synthesized by careful layering of metal chloride- and ligand-containing tetrahydrofuran (THF) solutions and letting them stand for 6 to 48 h upon which colorless or pale crystals deposit (Scheme 1, Figures S1–S6). If such obtained crystals were not suitable for single-crystal X-ray diffraction (SC-XRD), a CH_2_Cl_2_ (DCM) ligand solution was alternatively layered with an [AnCl_4_(DME)_*x*_] (An = U (*x* = 0), Np (*x* = 2), Pu (*x* = 2)) THF solution. Suspending [An^IV^Cl_4_(L)_2_]·2THF in DCM resulted in the formation of the respective [An^IV^Cl_4_(L)_2_]. Crystals suitable for single-crystal X-ray diffraction of the solvent free complexes [An^IV^Cl_4_(L)_2_] could be obtained from hot acetonitrile (NCMe). However, no product of the composition [An^IV^Cl_4_(L)_2_]·2THF or [An^IV^Cl_4_(L)_2_] could be obtained in case of thorium, most likely due to its larger ionic radius. This is supported by the fact that only [ThCl_4_(HPyO)_4_] was isolated from the reaction of [ThCl_4_(DME)_2_] with 2 equiv of HPyO (Figure S7).

**Scheme 1:**
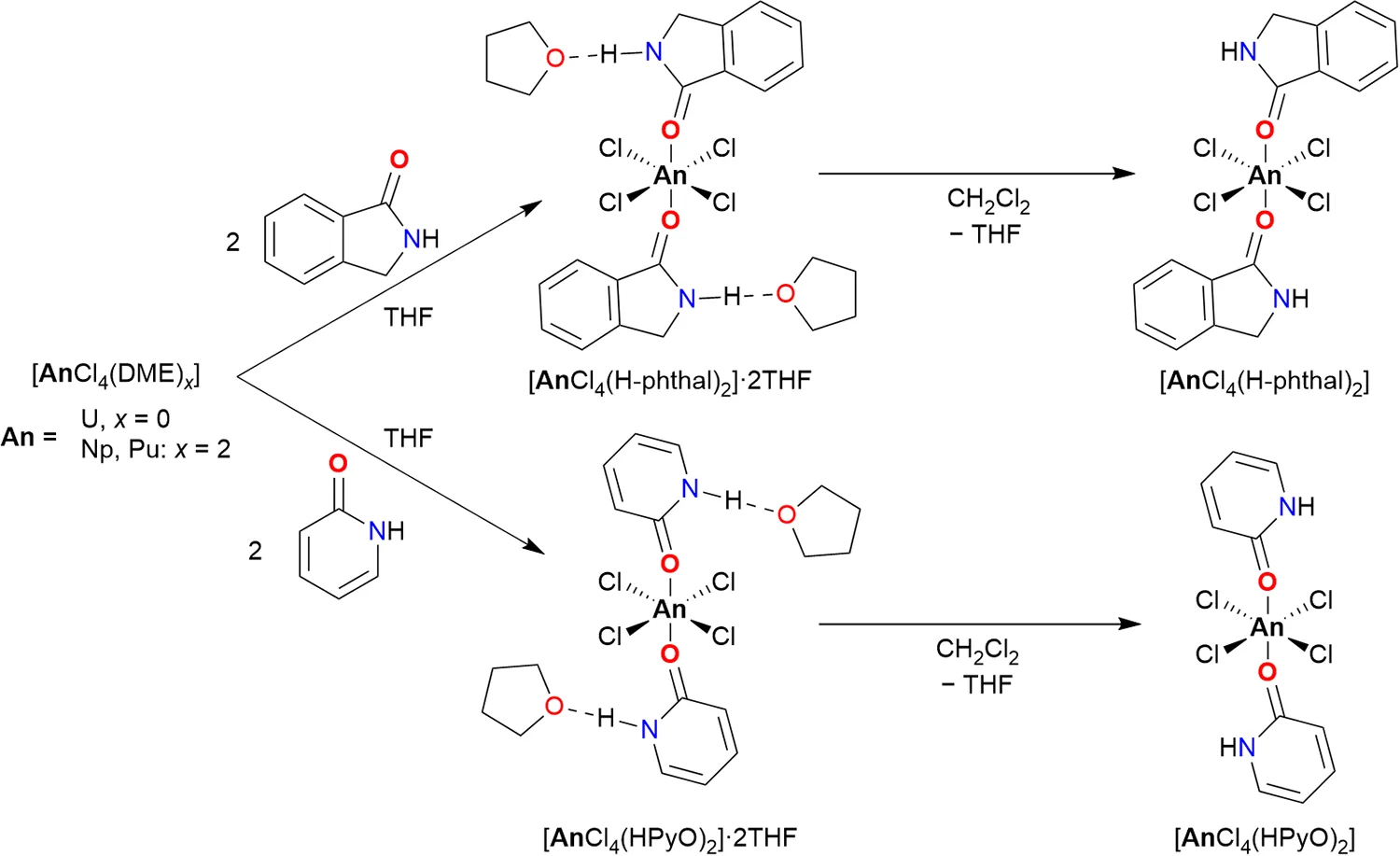
Synthesis of [AnCl_4_(L)_2_]·2THF and [AnCl_4_(L)_2_] Complexes

IR spectroscopy of the bulk material reveals very similar band positions along the complex series [AnCl_4_(L)_2_]·2THF (An = U, Np, Pu), which supports that the complexes are isostructural in solid state. The presence of the THF solvent molecules in the bulk material is confirmed by bands in the regions from 3100 to 2600 cm^–1^ and at about 860 cm^–1^ (Figure 1). The IR spectra of the [AnCl_4_(L)_2_] complexes show absence of vibrations in this region, supporting the successful removal of THF by suspending the complexes in DCM. For the HPyO complexes synthesized in THF, the IR-spectra of the bulk material exhibit the absence of THF related vibrations. Thus, we assume that only complexes of the form [AnCl_4_(HPyO)_2_] could be obtained in pure state upon drying. The phthalimidine complexes show a distinctive ν_CO_ stretching band around 1615 cm^–1^ in the IR spectra. This corresponds to a shift of about 60 cm^–1^ compared to the free ligand (1675 cm^–1^) upon complexation, indicating weakening of the C=O bond. For the THF-containing complexes, this band is found at 1612 cm^–1^ (U and Pu) or 1614 cm^–1^ (Np). After removal of THF, the bands shift to slightly higher wavenumbers for U and Pu (1618 and 1615 cm^–1^, respectively), and to lower wavenumbers for Np (1612 cm^–1^). The 2-pyridone complexes do not show a clear C=O stretching band in their IR spectra, which might be due to a partial delocalization of the electron density across the aromatic HPyO ligand. The full IR spectra including band positions are shown in the Supporting Information (Figures S8–S16).

**Figure 1:**
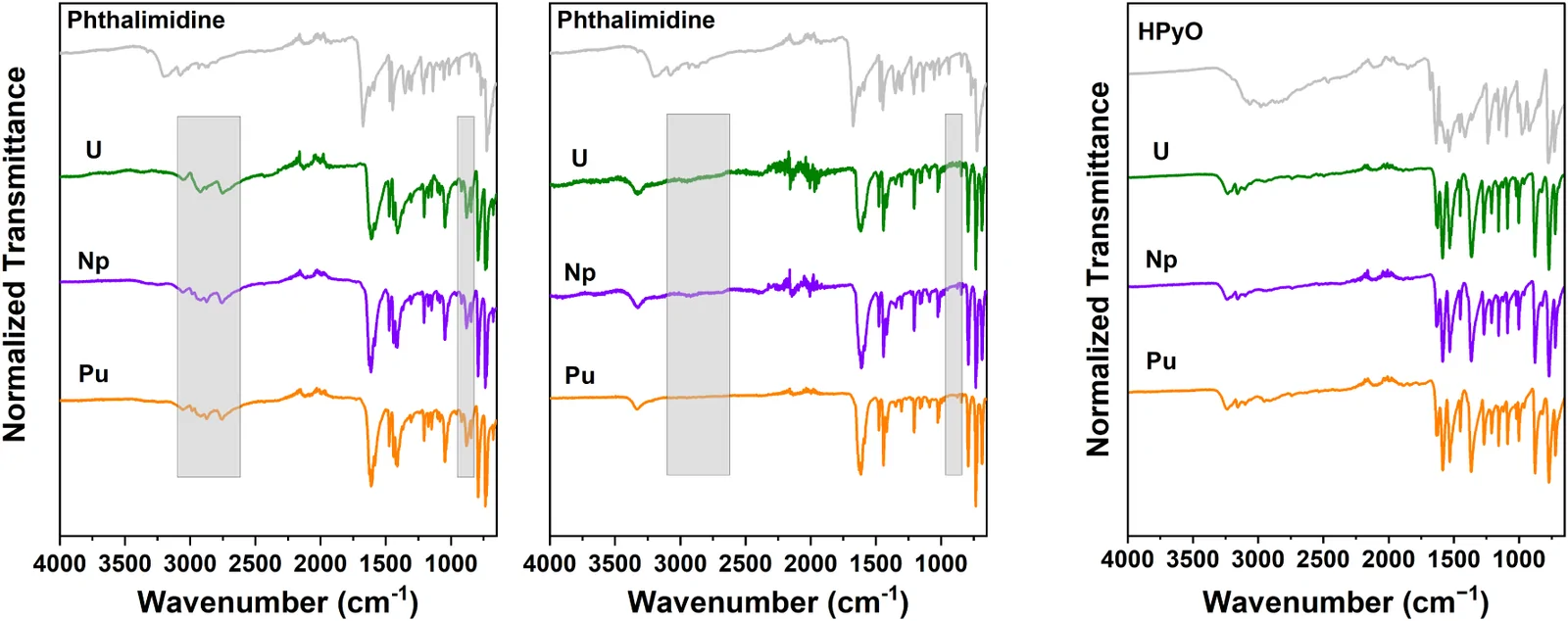
IR spectra of the [AnCl_4_(H-phthal)_2_]·2THF (*left*), [AnCl_4_(H-phthal)_2_] (*middle*) and [AnCl_4_(HPyO)_2_] (*right*). Areas showing significant differences due to THF are highlighted in gray.

Analysis of the obtained crystals by single-crystal X-ray diffraction (SC-XRD) revealed a slightly distorted octahedral coordination around the actinide centers with four chloride ligands in the equatorial plane and two organic ligands coordinating via the oxygen atoms in *trans* position for all compounds. The continuous shape measures indicate similar distortion along the complex series (0.51–0.71, Tables 1, 2). However, [NpCl_4_(H-phthal)_2_] shows an unusual distortion (CShM: OC-6 = 1.324), which we attribute to packing effects (*vide infra*). All crystal structures obtained from THF, with the exception of [NpCl_4_(HPyO)_2_], contain solvent molecules, which do not coordinate to the metal center. Instead, hydrogen bonds between the NH groups and the THF oxygen atom are observed (Figure 2 and Figures S17–S22).

**Table 1:** Selected Bond Lengths (Å) and Angles (°) of [AnCl_4_(H-phthal)_2_]·2THF and [AnCl_4_(H-phthal)_2_] (An = U, Np, Pu)

	**[AnCl**_**4**_ **(H-phthal)**_**2**_ **]·*x*THF**					
An	U**·**2THF	Np**·**2THF	Pu**·**2THF	U	Np	Pu
An1–O1	2.2282(11)	2.227(2)	2.2137(17)	2.240(2)	2.228(2)	2.2219(9)
An1–Cl1	2.6268(4)	2.6050(9)	2.5929(6)	2.6263(7)	2.8003(9)	2.5883(3)
An1–Cl2	2.6174(4)	2.5954(8)	2.5808(6)	2.6013(7)	2.4394(8)	2.5689(3)
An1–Cl_avg._	2.622(5)	2.600(5)	2.587(6)	2.614(13)	2.62(18)	2.579(10)
C=O	1.2742(19)	1.274(4)	1.275(3)	1.268(3)	1.278(4)	1.2666(14)
Cl1–An–Cl2	89.410(14)	89.67(3)	89.59(2)	87.35(2)	89.11(3)	87.727(11)
α^a^	87.24	87.19	87.03	87.90	80.54	87.96
CShM OC-6 ^35,36^	0.592	0.543	0.552	0.561	1.324	0.513

a The tilt angle α was defined as the minimal angle between the (An1–Cl1–Cl1*–Cl2–Cl2*)-plane and the An1–O1 bond axis.

**Table 2:** Selected Bond Lengths (Å) and Angles (°) of [AnCl_4_(HPyO)_2_]·2THF and [AnCl_4_(HPyO)_2_] (An = U, Np, Pu)

	**[AnCl**_**4**_ **(HPyO)**_**2**_ **]·*x*THF**			
An	U**·**2THF	U	Np	Pu**·**2THF
An1–O1	2.2011(17)	2.1962(18)	2.188(6)	2.194(5)
An1–Cl1	2.6326(6)	2.6550(6)	2.635(2)	2.594(2)
An1–Cl2	2.6271(7)	2.6155(6)	2.596(2)	2.590(2)
An1–Cl_avg._	2.630(3)	2.635(20)	2.615(20)	2.592(2)
C=O	1.295(3)	1.293(3)	1.290(10)	1.294(7)
Cl1–An–Cl2	89.84(2)	87.89(2)	87.80(7)	89.53(6)
α^a^	88.46	89.81	89.82	87.09
CShM OC-6 ^35,36^	0.668	0.714	0.691	0.627

a The tilt angle α was defined as the minimal angle between the (An1–Cl1–Cl1*–Cl2–Cl2*)-plane and the An1–O1 bond axis.

**Figure 2:**
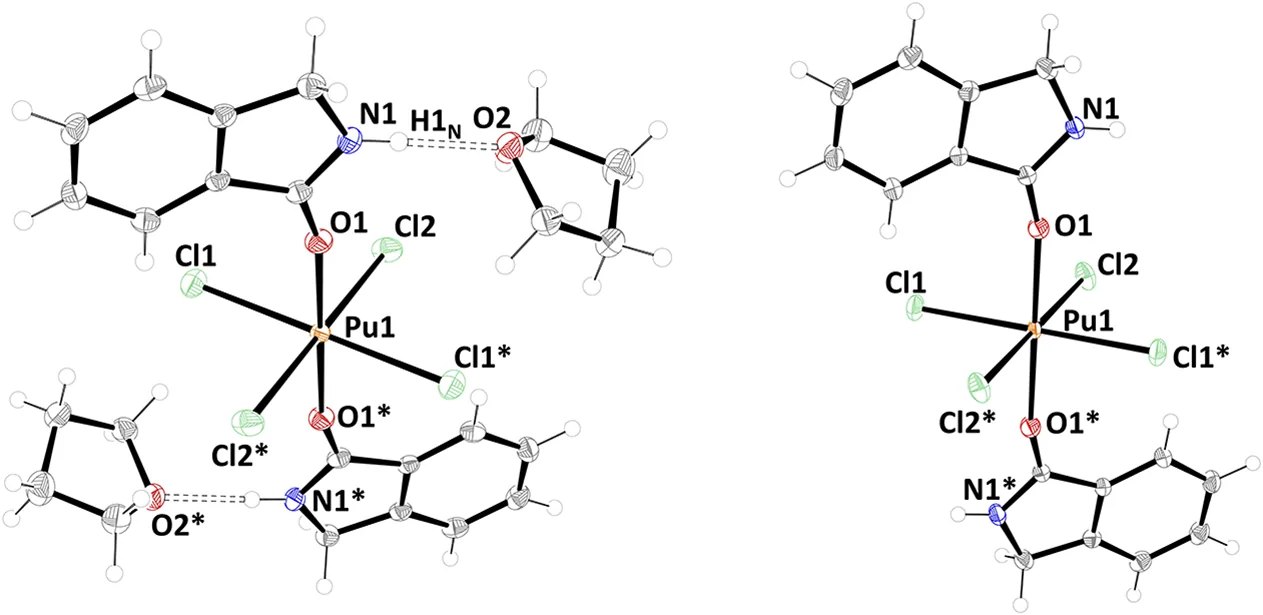
Molecular structures of [PuCl_4_(H-phthal)_2_]·2THF (*left*) and [PuCl_4_(H-phthal)_2_] (*right*). Ellipsoids are shown at 50% probability level. Symmetry elements * are −*x*, −*y* + 1, −*z* + 1 and −*x* + 2, −*y* + 1, −*z* + 2, respectively.

In more detail, all phthalimidine containing structures, [AnCl_4_(H-phthal)_2_]·2THF and [AnCl_4_(H-phthal)_2_], crystallize in the triclinic space group *P*1̅ and with consistent cell parameters along the actinide series (Tables S1–S4). The complexes containing 2-pyridone on the other hand show a less systematic behavior along the series and crystallize in either the monoclinic space group *P*2_1_/*c* or triclinic *P*1̅. They also show a wider variety in unit cell parameters. In all structures, the asymmetric unit contains half a complex molecule with the actinide center located on a center of inversion. One exception was found in case of [PuCl_4_(HPyO)_2_]·2THF, where the asymmetric unit contains one and a half complex molecules (one of the Pu atoms is located at a center of inversion), with only marginal deviations in bond distances and angles within the crystal structure.

Within the corresponding actinide series of isostructural complexes, the An1–O1 and An1–Cl_avg._ bond lengths decrease from U to Pu, as expected (Figure S23). In case of the uranium compounds, the bond lengths around the actinide of [UCl_4_(L)_2_]·2THF and [UCl_4_(L)_2_] (L = H-phthal, HPyO) can be compared with crystallographically characterized literature examples containing carboxylic amide or urea ligands (Chart [Fig chart1], *top*; Figure 3). The complexes from this study are well in line with the previously reported bond lengths, which range from 2.209(7) Å (L = *N,N,N′,N′*-tetramethyl urea, tmu) to 2.268(18) Å (L = *N,N′*-dimethyl-*N,N′*-diphenyl urea, ddu) for the U–O bonds and from 2.606(15) Å (L = ddu) to 2.622(4) Å (L = tmu) for the U–Cl bonds. The graphical representation (Figure 3) clearly suggests a correlation between the two bond lengths – shorter U–O bonds lead to longer U–Cl bonds. This is associated with the steric bulk of the ligand, where a smaller ligand (e.g., tmu) leads to shorter U–O bonds, thus, increasing the steric demand at the metal center and yielding longer U–Cl bonds. Based on this, the HPyO ligand is the smallest ligand of the series while H-phthal is larger than tmu but smaller than *N,N*-dimethylpivalamide^24^ and ddu.

**Figure 3:**
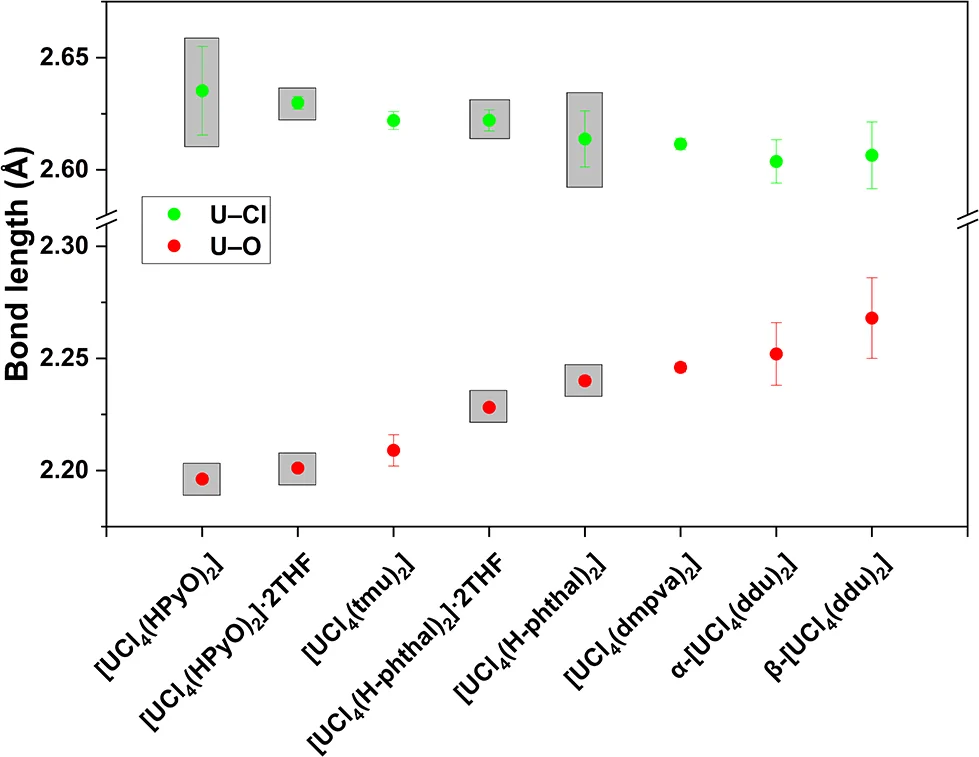
U–Cl and U–O bond lengths in various [UCl_4_(L)_2_] complexes (L = HPyO, tetramethyl urea (tmu),^26^ H-phthal, dimethylpivalamide (dmpva),^24^ dimethyldiphenyl urea (ddu)^28^) in the order of increasing U–O bond length correlating well with the steric bulk of the ligands. Complexes from this study are highlighted in gray.

The C=O bond lengths in the [AnCl_4_(H-phthal)_2_]·2THF (An = U, Np, Pu) complexes do not show significant differences between the actinides, all being about 1.274(3) Å (Table 1). This is significantly longer than in free phthalimidine (1.243(3) Å).^37^ The [AnCl_4_(HPyO)_2_]·*x*THF complexes exhibit a similar behavior irrespective of the THF molecules in the structure, with C=O distances of about 1.293(6) Å (Table 2) and a free ligand value of 1.251(1) Å.^38^ However, this changes if the THF is removed from the [AnCl_4_(H-phthal)_2_] complexes. For U and Pu, the removal of THF leads to a shortening of the C=O bond to 1.268(3) and 1.267(1) Å, respectively, while the bond is slightly elongated to 1.278(4) Å for Np, which is also reflected in the IR spectra (*vide supra*).

A closer investigation of the crystal structures reveals that the [AnCl_4_(L)_2_]·2THF and [AnCl_4_(L)_2_] complexes contain a longer and a shorter An–Cl bond (Tables 1, 2), which are *cis* oriented to each other. This is a common feature in many pseudo-octahedral actinide complexes (see Chart [Fig chart1]), indicating an inverse *trans* influence to be present in this type of actinide complexes.^39,40^ The difference in bond length between An1–Cl1 and An1–Cl2 is in the range of 0.004(3)–0.361(1) Å. The average difference in THF containing structures is 0.01 Å, whereas the deviation is on average higher for the solvent free structures and reaches its maximum in [NpCl_4_(H-phthal)_2_] with 0.361(1) Å. In fact, the extreme difference in [NpCl_4_(H-phthal)_2_] leads to the shortest Np–Cl bonds observed so far: with only 2.439(1) Å in comparison to 2.568(2) Å in [NpCl_4_(THF)_3_][Bibr ref41] and 2.568(1) Å in [(^Mes^PDP^Ph^)­NpCl_2_(THF)].^42^ The difference may be rationalized by considering the intermolecular interactions in the crystal structures (Figures S24 and S25). Here, the shorter bond shows short contacts to the aromatic protons of the neighboring complex molecule, potentially leading to a repulsion of the chloride atom closer to its actinide center. In turn, the orthogonally located chloride ligand is pushed out of the coordination sphere into a free space in the crystal structure. Due to differences in the packing, this is not possible for the other actinides, where the other chloride atom is restrained by a neighboring complex molecule. This results in An–Cl distances of 2.6013(7) and 2.6263(7) Å for U, and 2.5689(3) and 2.5883(3) Å for Pu. In case of [NpCl_4_(HPyO)_2_], the difference in Np–Cl bond lengths is about 0.039 Å, similar to the corresponding uranium complex. The crystal packing of [NpCl_4_(H-phthal)_2_] shows some hydrogen bonding from the NH moiety of the phthalimidine to the chloride ligands of the neighboring complex molecule, replacing the interaction to the solvent molecule in the THF-containing structures. With a N···Cl distance of 3.188 Å, this interaction can be classified as a moderate to weak H-bond (A···B distance of 2.5–3.2 Å), with the N–H···Cl angle of 134° (130–180°).^43^ In case of [UCl_4_(H-phthal)_2_] and [PuCl_4_(H-phthal)_2_], the N···Cl distance is found to be 3.301 Å (U) and 3.308 Å (Pu). Interestingly, a similar effect can be observed for the THF-containing structures, where the Np complex shows a N···O­(THF) distance of 2.680(4) Å compared to 2.711(2) Å for U and 2.717(2) Å for Pu. In addition, [NpCl_4_(H-phthal)_2_] is the only complex in this series, in which the An–O bond is significantly tilted toward the AnCl_4_-plane, showing a tilt angle of 81°. In all other cases, the O–An–O-axis is nearly perpendicular to this plane (>87°).

### Bonding Analysis

In order to better understand the rather unexpected stability of the 6-fold coordinated actinides and to gain a deeper insight into the characteristics of the bonds, electron density- and orbital-based bonding analyses have been performed. To furthermore bring the results in line with the whole series of early actinides, the theoretical studies were extended to complexes of Th and Pa, which could not be obtained experimentally. The basis of all calculations were the obtained XRD-structures, which were then optimized with implicit solvation (def-SVP^44^ + ECP­(An)/PBE0 ^45,46^ /COSMO­(THF)^47^) using the TURBOMOLE 7.8.1 program package.^48^ After validation of the found minima via frequency analysis, these structures were used to perform all-electron single-point calculations via ORCA 6 ^49^ that explicitly consider scalar-relativistic effects present due to the actinides (X2C-TZVPALL+SARC-DKH-TZVP^50^/PBE0). Finally, the converged wave functions were used to perform QTAIM- (via AIMAll^51^) and Natural Population Analyses (via NBO7 ^52^).

The diagrams in Figure 4 show the An–L bond lengths (*top*) as well as the associated Delocalization Indices (DI) from the QTAIM analysis (*middle*) and the calculated charges of the central metal ions (*bottom*). As mentioned, both the An–Cl and An–O bond lengths decrease with increasing atomic numbers, from U to Pu. The theoretical Th and Pa compounds precede this trend. H-phthal and HPyO complexes only slightly differ with respect to their bond lengths, in that the An–Cl bonds for H-phthal are slightly shorter, which in turn leads to elongated An–O bonds in good accordance with the crystallographic data. The DIs are a measure of covalent bond order.^53^ They reveal that significantly higher covalent contributions can be found in the An–Cl bonds, although the An–O bonds are much shorter. Moreover, while the DIs of the An–Cl bond seem to continuously increase from Th to Pu, the respective values of the oxygen bonds reach an apparent plateau starting with U. Mirroring the differences between the bond lengths of H-phthal and HPyO species, slightly increased DIs of An–Cl bonds (around 0.01) and a corresponding decrease in DIs (0.02–0.03) of An–O bonds can be found for H-phthal complexes.

**Figure 4:**
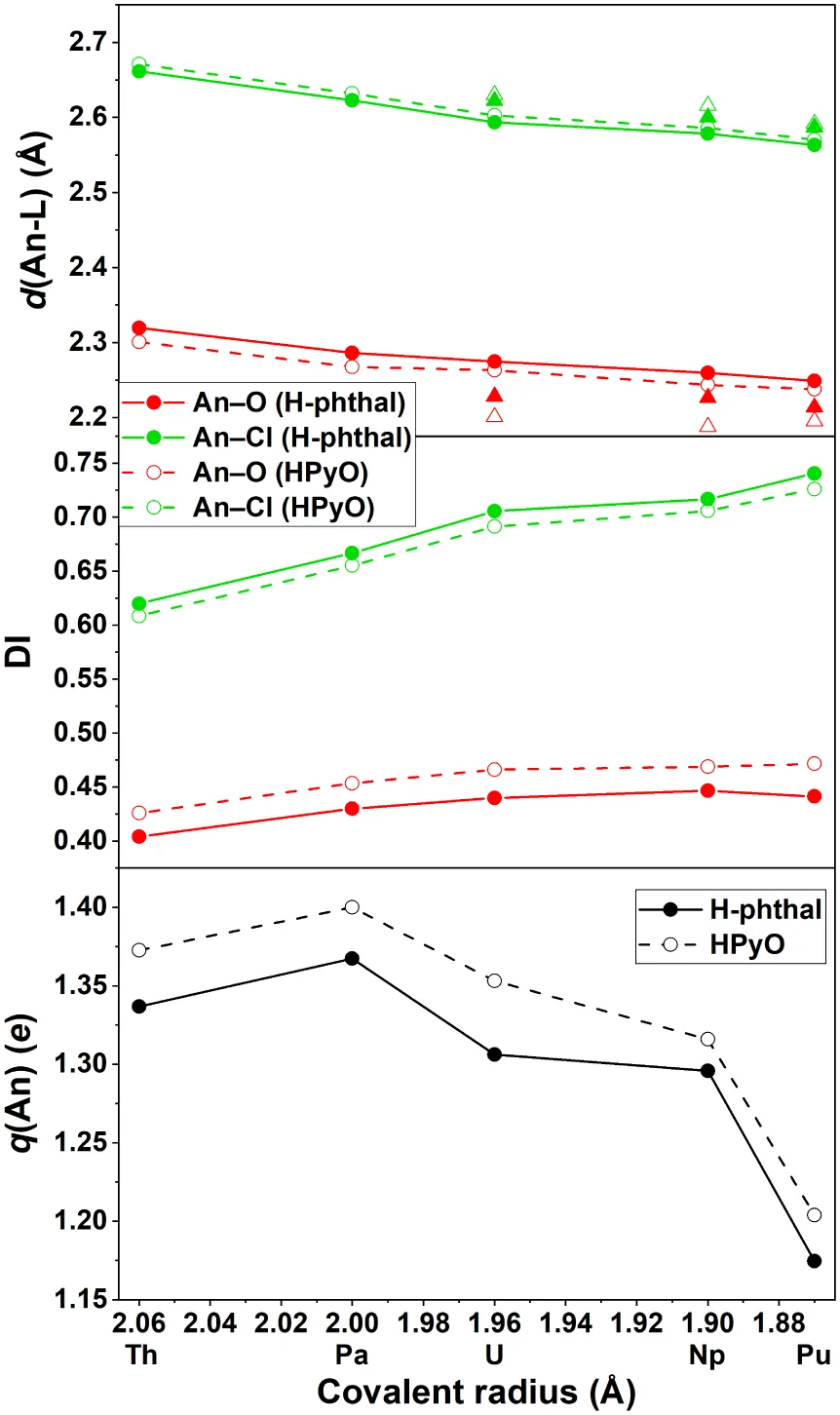
Diagrams of the An–L bond lengths (*top*) and different quantum chemical measures of covalency for HPyO and H-phthal complexes of Th to Pu: Delocalization indices (*middle*) and natural charges of An (*bottom*). Experimental bond lengths obtained from SC-XRD are indicated as triangles.

Extending the picture to natural orbital-based population analysis, the trend of increasing covalency of actinide ligand bonding is validated by the natural charges of the An^IV^, obtained from natural population analysis.^54^ Apart from an opposing initial increase from Th to Pa, the charge values experience a continuous decrease toward Pu, indicating increasing charge transfer from the ligand to the metal. The comparison between H-phthal and HPyO species reveals that the H-phthal complexes feature the overall higher amount of covalency in An–Cl bonds, whereas the An–O bonds are less covalent based on DI values.

### Characterization in Solution

The solution state characterization with Nuclear Magnetic Resonance spectroscopy (NMR) of the [AnCl_4_(L)_2_]·2THF complexes was hampered by their low solubility in common NMR solvents like CD_2_Cl_2_ or THF-*d*_8_ (Figure S26). The low solubility prevented the detection of ^13^C NMR signals as well as the full assignment using 2D NMR techniques in particular for the 2-pyridone complex family. In the corresponding ^1^H NMR spectra, however, both complex series showed one set of signals, indicating that the overall symmetry is retained so that both ligands are chemically equivalent. Signals of the free ligands were not observed, underlining the stability of the complexes even in coordinating solvents (see Section Conformer Search and Molecular Dynamics Simulations). The [AnCl_4_(HPyO)_2_]·2THF complexes in THF-*d*_8_ solution therefore showed a group of four signals of the aromatic CH protons and one signal of the NH proton. The [AnCl_4_(H-phthal)_2_]·2THF complexes in CD_2_Cl_2_, on the other hand, exhibited four signals due to the aromatic CH groups, one signal of the NH group and one signal of the aliphatic CH_2_ group.

If the crystals obtained from the reaction were used directly after isolation, significant THF signals obscured the spectra revealing the presence of THF in the crystal structure. The corresponding signals, however, showed no significant paramagnetic shifts, therefore suggesting THF molecules as solvates within the structure. A large excess of noncoordinating solvent like DCM, leads to removal of the THF molecule. Therefore, obtaining solvent-free compounds [AnCl_4_(L)_2_] was possible by stirring the crystals from the synthesis in DCM as described above. For the coordinating solvents THF and NCMe, additional reaction path calculations were performed (Figures S27–S28, Tables S11–S24), to validate the hexacoordination at the actinide center in solution. Only the theoretical case of [ThCl_4_(H-phthal)_2_] shows a significant stabilization by coordination of a THF molecule to the metal, supporting our hypothesis of the too large ionic radius of Th for such a hexacoordinated arrangement. [UCl_4_(H-phthal)_2_] shows a slight decrease in energy upon introducing a THF molecule to the coordination sphere, while in all other cases, the hypothetical heptacoordinated structures are less stable than the hexacoordinated ones. In case of NCMe, the hexacoordination of An^IV^ is always preferred (Figure S28). However, due to the rather low energy barrier, the occurrence of an intermediate heptacoordination in solution cannot be ruled out, neither in the bulk nor in average of the NMR time scale.

Encouraged by the indicated stability of the complexes even in the presence of coordinating solvents, we turned to NCMe for NMR spectroscopy. Indeed, the phthalimidine complexes in particular showed a reasonable solubility in NCMe-*d*_3_ forming pale solutions, which enabled the recording of solution state NMR spectra (Figures S29–S59, Tables S25–S30). While the signals appear rather broad at room temperature, they become increasingly sharp at elevated temperatures – all the way to 65 °C, the highest available temperature for acetonitrile in variable temperature NMR (VT-NMR) studies. At this high temperature, all ^1^H and most ^13^C NMR signals could be identified and assigned using two-dimensional NMR techniques (Table 3, see Figures 6 and 7 for structural representation of [UCl_4_(L)_2_] at 65 °C).

**Table 3:** ^1^H and ^13^C NMR Chemical Shifts of the [AnCl_4_(H-phthal)_2_] Complexes (ppm, NCMe-d_3_, 65 °C, Assignment See Figure S29)

^1^H	NH	H3	H4	H5	H6	H7
H-phthal	6.77	4.39	7.55	7.59	7.48	7.72
U	10.40	3.10	7.71	7.87	7.59	8.16
Np	7.92	–0.10	6.51	7.03	6.78	5.08
Pu	8.49	2.80	7.44	7.61	7.27	6.78

^13^C	C1	C3	C3a	C4	C5	C6	C7	C7a
H-phthal	171.6	46.1	145.4	124.0	132.5	128.7	124.6	133.4
U	194.9	53.1	145.4	125.8	134.4	130.3	124.7	135.0
Np	144.4	51.6	140.7	124.0	132.9	128.7	121.3	128.9
Pu	-	51.1	143.8	124.5	134.1	129.2	124.1	130.0

The complexes [AnCl_4_(H-phthal)_2_] show the expected signals for the ligand atoms, i.e., four aromatic CH protons, one NH proton and one CH_2_ group in the aliphatic region in addition to six aromatic carbon atoms, an aliphatic one for the CH_2_ group, and one signal for the carbonyl carbon atom, which could not be located unambiguously for [PuCl_4_(H-phthal)_2_]. Only one set of signals is observed for the two ligands, which points to at least *C*_*2*_ symmetry in solution. Although a paramagnetic center is present, the change in chemical shifts of the signals in the complexes compared to the free ligand is only relatively small. The main exception are the protons of the CH_2_ group (H3, see Figure S29), which experience a significant upfield shift in all cases. Additionally, the aromatic proton pointing toward the metal center (H7) exhibits a significant broadening and is shifted more strongly than the other aromatic proton signals (Figure 5, *top left*). Generally, these effects are strongest in the Np complex where the CH_2_ signal (H3) is shifted to −0.10 ppm, while this signal in the U and Pu complexes is found at 3.11 and 2.80 ppm, respectively. [NpCl_4_(H-phthal)_2_] is also the only case where the other aromatic CH signals exhibit a significant shift compared to the free ligand (Figure 5). In the ^13^C NMR spectrum, especially the carbonyl carbon shows a significant shift (194.9 ppm for U, 144.4 ppm for Np), possibly due to a Fermi contact contribution from the paramagnetic metals over two bonds. For [PuCl_4_(H-phthal)_2_], this signal could not be identified. As in the case of the ^1^H spectra, the strongest shifts overall are observed for the Np complexes, especially the carbon atoms C3a and C7.

**Figure 5:**
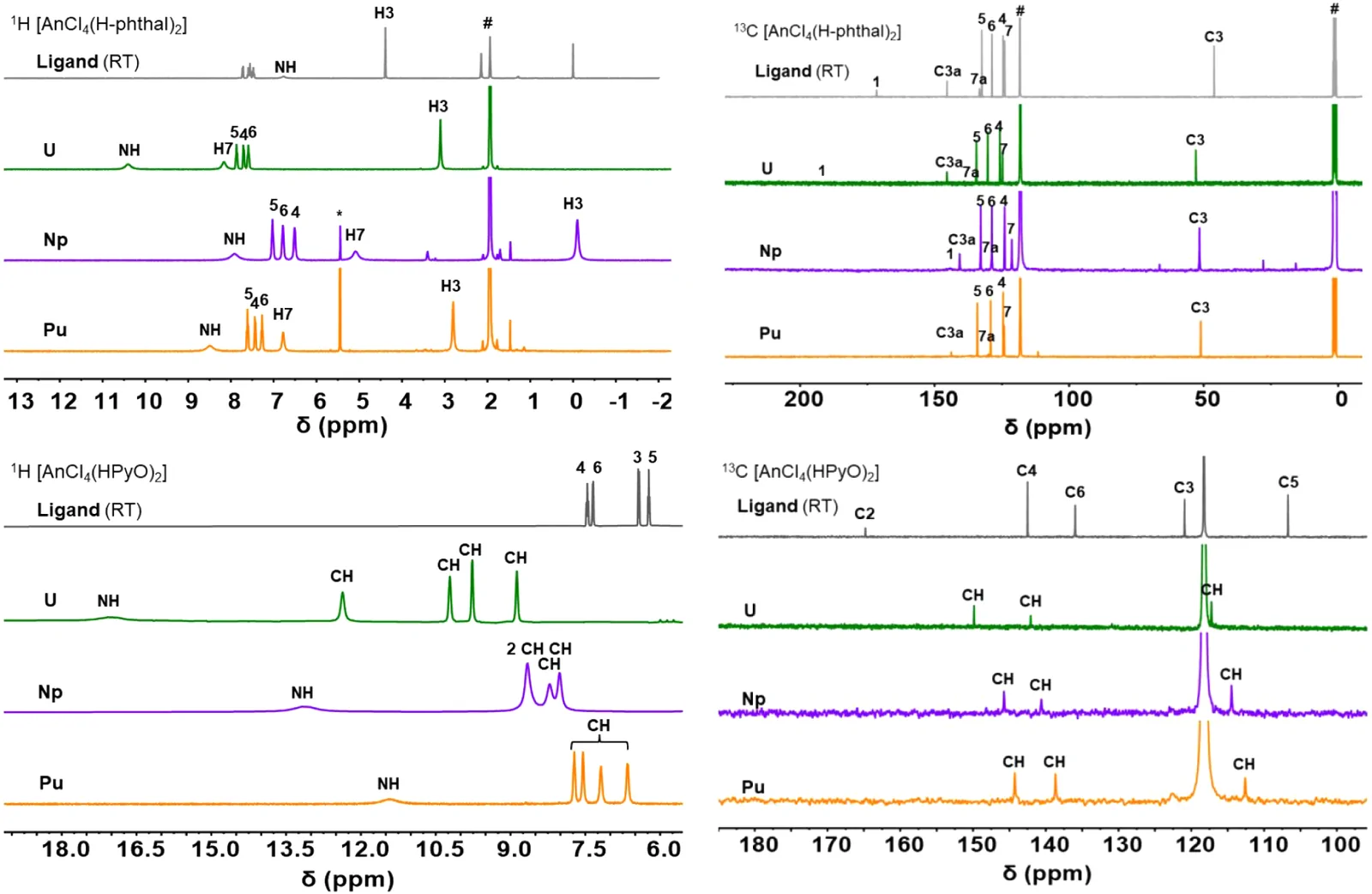
^1^H (*left*) and ^13^C NMR spectra (*right*) of [AnCl_4_(H-phthal)_2_] (An = U, Np, Pu) (*top*) and [AnCl_4_(HPyO)_2_] (*bottom*) in NCMe-d_3_ at 65 °C in comparison to phthalimidine and 2-pyridone (*gray*), respectively (*: DCM; #: NCMe).

The 2-pyridone complexes showed a significantly poorer solubility in NCMe-*d*_3_, leading to lower signal intensities. Furthermore, the presence of paramagnetic metal centers causes substantial line broadening. These effects combined to prevent an unambiguous assignment of both proton and carbon signals. However, one set of signals is observed for every complex in the ^1^H NMR spectra, indicating a high molecular symmetry consistent with the retention of the octahedral coordination in solution. Only a few ^13^C NMR signals could be resolved and identified as CH-bound carbon atoms. Despite these limitations, the general trends resemble those observed for the phthalimidine analogues: sharper resonances are obtained at elevated temperatures, and the broadest signals are found for the neptunium complexes. Interestingly, the paramagnetic shift of the signals shows a different trend, with the largest shifts for U instead of Np as seen from the overall range of the CH signals (12.37–8.87 ppm for U to 7.72–6.65 ppm for Pu), with the signals of Pu essentially in the diamagnetic region.

### Conformer Search and Molecular Dynamics Simulations

Since the sterically sparse coordination environment of the two complex species does not force the organic ligands into a rigid orientation, and thus allows for a high spatial flexibility, both species are assumed to exist as ensembles of many energetically close-lying conformers in solution. To better understand the behavior of the [AnCl_4_(L)_2_] complexes in solution, we applied two theoretical approaches. The first one is the Global Optimizer Algorithm module (GOAT)^55^ of ORCA, which allows for a stochastic exploration of the potential energy hypersurface yielding the full ensemble of possible conformers as well as the global minimum. In order to correctly describe the involved actinides with an appropriate basis set, DFT with the composite functional r^2^SCAN-3c^56^ (and the associated def2-mTZVPP basis) had to be employed. As this option is computationally time-consuming, only the two U complexes [UCl_4_(H-phthal)_2_] and [UCl_4_(HPyO)_2_] were chosen for this investigation as representatives. The NCMe solvent was simulated via the Conductor-like Polarizable Continuum Model (CPCM).^57^ The diagram on the left in Figure 6 depicts the energy landscape of all found *trans* conformers, which are 291 for [UCl_4_(H-phthal)_2_] and 182 for [UCl_4_(HPyO)_2_]. According to *RT*, only conformers up to 0.67 kcal/mol are reachable at 65 °C (depicted as gray area Δ*G vs* conformer plot in Figure 6, *left*). However, reoptimization of the conformers with more accurate functionals like PBE0 can change the energy substantially (Figure S60). The results show that the optimized crystal structure has the lowest energy and represents about half of the population. The other half is distributed among all of the chosen GOAT conformers. This demonstrates that at this temperature these complexes exist as an ensemble of various energetically close conformers. The GOAT conformers exhibit virtually every rotational degree for the ligands while maintaining U–O–C angles of 138.4–175.4° ([UCl_4_(H-phthal)_2_]) and 141.6–157.9° ([UCl_4_(HPyO)_2_]). Cl–U–O angles vary between 84.4–96.6° ([UCl_4_(H-phthal)_2_]) and 85.0–95.6° ([UCl_4_(HPyO)_2_]) demonstrating that the orthogonality of the distorted octahedron remains mostly intact.

**Figure 6:**
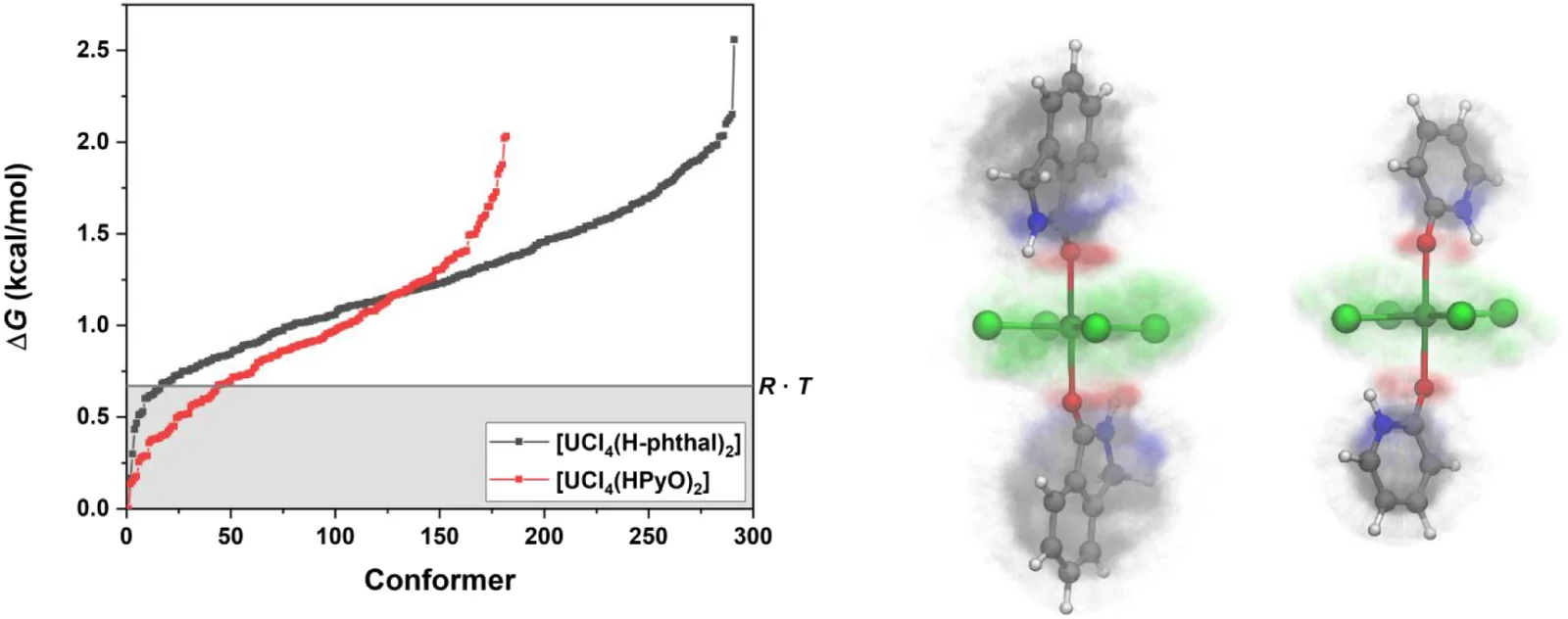
Plot of Δ*G* values of all found GOAT *trans* conformers for [UCl_4_(H-phthal)_2_] and [UCl_4_(HPyO)_2_] (*left*). Structural overlay of conformers for [UCl_4_(H-phthal)_2_] and [UCl_4_(HPyO)_2_] (*right*). The energetically lowest conformer is drawn with full opacity, while all other structures are depicted transparent.

Our second approach applied molecular dynamics (MD) simulations for [UCl_4_(H-phthal)_2_] and [UCl_4_(HPyO)_2_] utilizing the GFN force field,^58^ which has recently been parameterized for actinides.^59^ The setup for the MD was a cluster of the complex molecule surrounded by 120 molecules of NCMe. The placement of the solvent was handled with ORCA’s SOLVATOR algorithm (stochastic mode). To prevent the molecules from escaping, a spherical logfermi potential was applied for the MD simulation. The MD simulations were run for 300 ps (step length of 1 fs) at 65 °C including a temperature equilibration time of 30 ps. The resulting trajectories were then analyzed with TRAVIS^60^ and spatial as well as combined angular distribution functions could be extracted (see Figure 7). The spatial distribution functions (SDF) were plotted at 0.1 (*blue*), 5 (*orange*), and 25 (*red*) occurrences per nm^–3^ with maximal values of 69.8 nm^–3^ ([UCl_4_(H-phthal)_2_]) and 92.8 nm^–3^ ([UCl_4_(HPyO)_2_]). The blue regions validate the rotational freedom of the ligands already seen in the GOAT conformers. From the orange regions it can be derived that the ligands rotate around a virtual axis prolonged from the U–O bond, where the ligand plane and the axis are mostly coplanar, with occasionally some small tilts. The red parts show that the distorted octahedral coordination environment is roughly maintained, although more so for the [UCl_4_(HPyO)_2_] complex than for [UCl_4_(H-phthal)_2_]. This is further confirmed by the combined angular distribution functions diagram, where [UCl_4_(HPyO)_2_] has a strongly confined maximum at 90° for the Cl–U–O angle while [UCl_4_(H-phthal)_2_] shows one peak slightly above and another slightly below 90°, meaning that the U–O axis is slightly tilted for most of the structures. In addition, the diagram shows that a wider range is strongly populated for the U–O–C angle in the case of [UCl_4_(H-phthal)_2_], and the maximum is also shifted to higher values compared to [UCl_4_(HPyO)_2_]. This reflects the bigger domain of possible U–O–C angles already obtained from the GOAT calculation. In conclusion, the GOAT and MD simulation are able to give structural insight of [AnCl_4_(L)_2_] complexes at 65 °C and support the experimental NMR findings.

**Figure 7:**
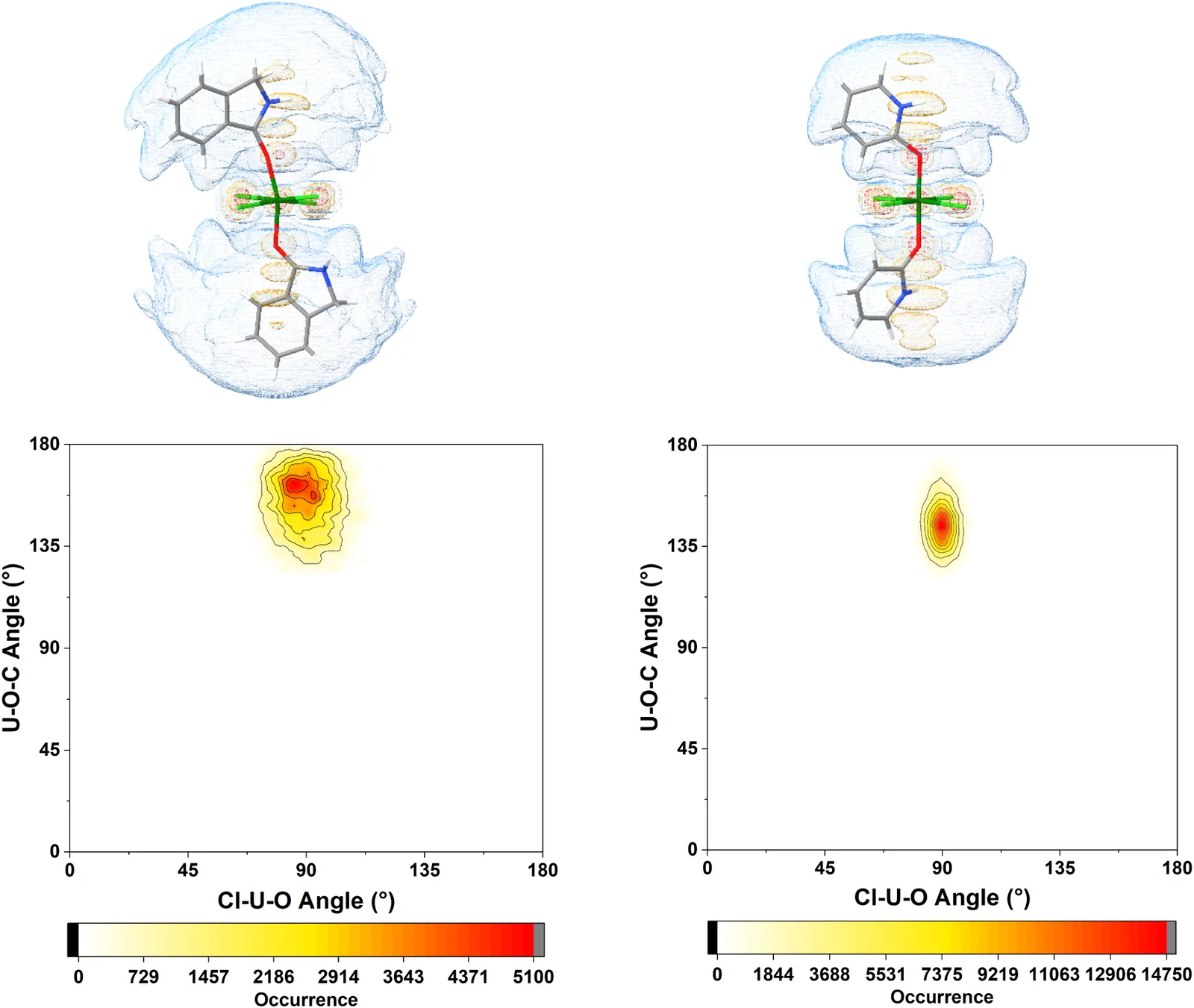
Spatial distribution functions (*top*) of [UCl_4_(H-phthal)_2_] (*left*) and [UCl_4_(HPyO)_2_] (*right*) derived from 300 ps-MD runs. The isosurfaces with different colors represent 0.1 (*blue*), 5 (*orange*), and 25 (*red*) occurrences per nm^–3^ with maximal values of 69.8 nm^–3^ (H-phthal) and 92.8 nm^–3^ (HPyO). Combined angular distribution functions diagrams (*bottom*) of [UCl_4_(H-phthal)_2_] (*left*) and [UCl_4_(HPyO)_2_] (*right*) derived from 300 ps-MD runs with the U–O–C angle on the *Y*-axis and the Cl–U–O angle on the *X*-axis.

### Actinide Oxidation States Determination

To gain insight into the electronic properties of the actinide ions, e.g., to confirm the oxidation state, the [AnCl_4_(L)_2_] complexes were analyzed by X-ray Absorption Near-Edge Structure (XANES) spectroscopy in solid state. This method employs X-rays generated by a synchrotron to excite core electrons into empty valence states. Due to its sensitivity toward the electronic structure, the An ions were investigated at their respective M_4_ edge that corresponds to the transition from 3*d*_3/2_ to 5*f*_5/2_ states.^61^ The short lifetime of these core-holes causes a significant broadening of the obtained spectra. To circumvent this problem, the measurements were performed in the High Energy Resolution Fluorescence Detection (HERFD) mode, where the signal detection occurs by measuring the fluorescence as the core-hole is filled by an outer shell 4*f*_5/2_ electron. This method of measurement enables a significantly higher resolution to determine the oxidation state of the actinide by comparison with an appropriate reference.^62^

The HERFD-XANES spectra at the An-M_4_ edge of complexes of the type [AnCl_4_(H-phthal)_2_]·2THF or [AnCl_4_(HPyO)_2_] (An = U, Np, Pu) are shown in Figure 8, together with the respective actinide dioxides as tetravalent references. The absorption peaks exhibit perfect overlap with the AnO_2_ references, confirming that the actinides are in their +IV oxidation state in all cases. Remarkably, the overlap persists despite strongly different coordination environment (cubic vs octahedral coordination at An^IV^; O_8_ coordination vs O_2_Cl_4_). The energies of the absorption maximum of each actinide M_4_ edge are identical for the respective complex series and for AnO_2_, found at 3725.1, 3850.1, and 3970.7 eV for U, Np, and Pu. These results highlight that HERFD-XANES measurements at the M_4_ edge can be highly valuable to accurately determine the oxidation states of samples containing actinides, independent of their coordination environment. This proves especially useful in environmental samples where the oxidation state is not easily determined by other methods and the coordinating donor atoms and geometry remain unclear.^63–68^

**Figure 8:**
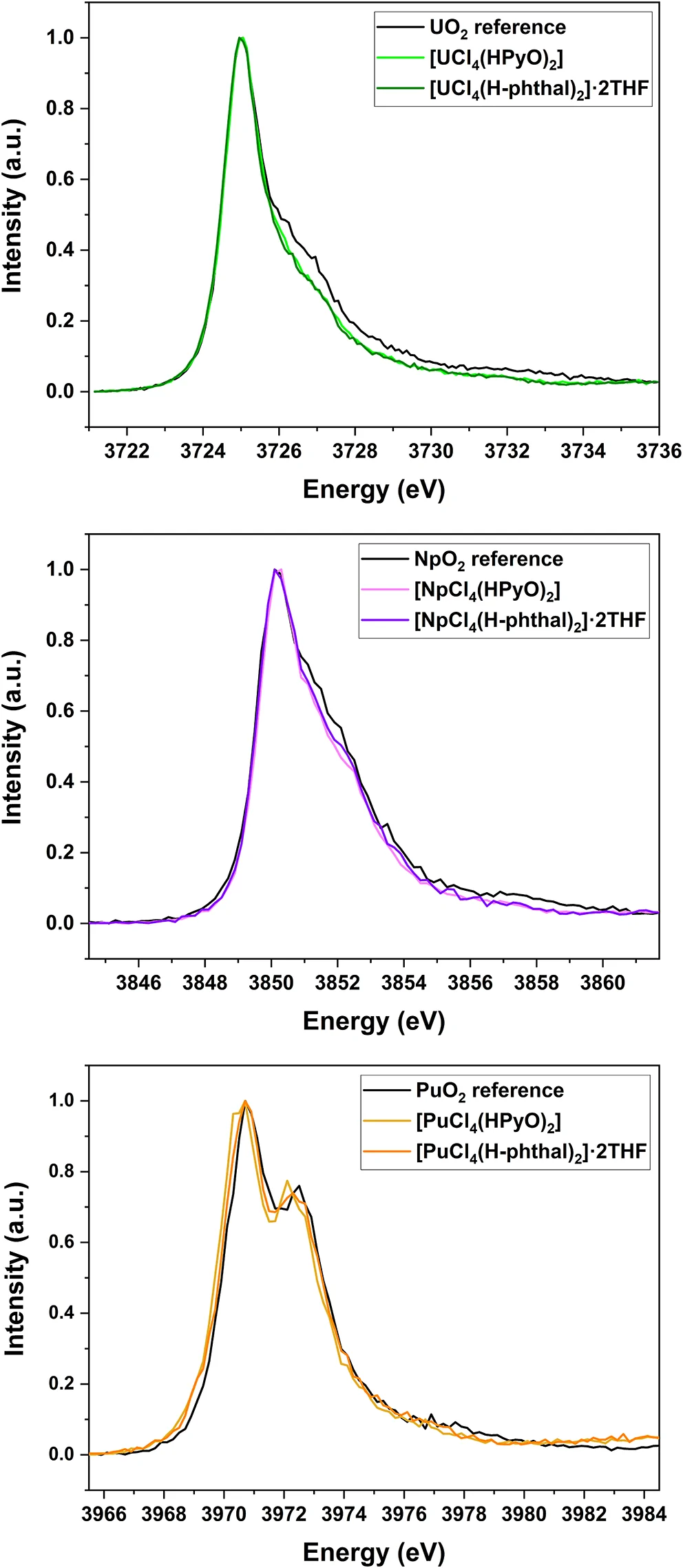
Normalized HERFD-XANES spectra at the An-M_4_ edge of [AnCl_4_(H-phthal)_2_]·2THF and [AnCl_4_(HPyO)_2_] complexes (An = U, Np, Pu) at 100 K.

The oxidation state of actinides in solution can be further verified using Vis-NIR spectroscopy. The solubility of the [AnCl_4_(H-phthal)_2_] complexes in acetonitrile allowed for the recording of solution state Vis-NIR spectra. This method would enable us to (i) confirm the oxidation state of the metals in solution, and (ii) investigate whether the high symmetry of the complexes would lead to a lower extinction coefficient as several electronic transitions are forbidden in an octahedral coordination environment. The obtained spectra in the range of 400–1100 nm are shown in Figure 9 with the band positions and extinction coefficients given in the Supporting Information. For actinides, this region is characteristic for *f-f* transitions while the region below 400 nm is obscured by a strong charge transfer band. For the region with *f-f* transitions, it is only valid to compare Vis-NIR spectra between compounds with the same *f*-electron count. As multiple *f-f* transitions are visible and not resolved at ambient temperature, due to spin–orbit coupling and ligand field effects, a clear assignment of the transitions cannot be made.^69^ However, the spectra can be analyzed as supporting “fingerprint” evidence. For U^IV^, the overall structure of the spectrum is similar to previously reported [UX_4_(L)_2_] complexes, although the bands in the visible region are slightly shifted to higher wavelengths more similar to the iodo derivative of tetramethylurea.^27^ The band positions are also similar to the tetravalent actinides in dimethyl sulfoxide solutions.^70^ The band positions of the Np^IV^ and Pu^IV^ complexes are in good agreement with similar complexes reported in the literature, respectively.^71^ The extinction coefficients of the *f-f* transitions are generally quite low for all our complexes, in particular for U^IV^ and Pu^IV^ (<30 M^–1^ cm^–1^), which is even lower than for the actinide aquo ions in perchloric acid solution (up to 60.9 for U^IV^ ^72^ and 56.6 M^–1^ cm^–1^ for Pu^IV^ ^73^ ). The absorption of [NpCl_4_(H-phthal)_2_] is stronger with a maximum at 756 nm, in line with the observations of Np^IV^ aquo ion in 2 M HClO_4_ solution. For Np^IV^, the extinction coefficient is also slightly reduced compared to the aqueous systems (around 125 M^–1^ cm^–1^ compared with 85 M^–1^ cm^–1^ here).^73^ Interestingly, one of the main features of Np^IV^ in both HClO_4_ and DMSO, i.e., a strong sharp band at 960 nm, is only visible as a shoulder in [NpCl_4_(H-phthal)_2_]. This absorption at 960 nm is also reduced in intensity in the complex [Np­(O^*t*^Bu)_4_(Pyr)_2_] with a similar hexacoordination around Np^IV^,^74^ and therefore suggests that the hexacoordination of Np in [NpCl_4_(H-phthal)_2_] remains intact in solution. Further, the reduced extinction coefficient supports the predicted idealized *D*_4h_ symmetry in the first coordination sphere of An^IV^ in solution, as indicated by GOAT and MD simulations. All Vis-NIR spectra thus confirm the presence of An^IV^ in symmetrical coordination environments.

**Figure 9:**
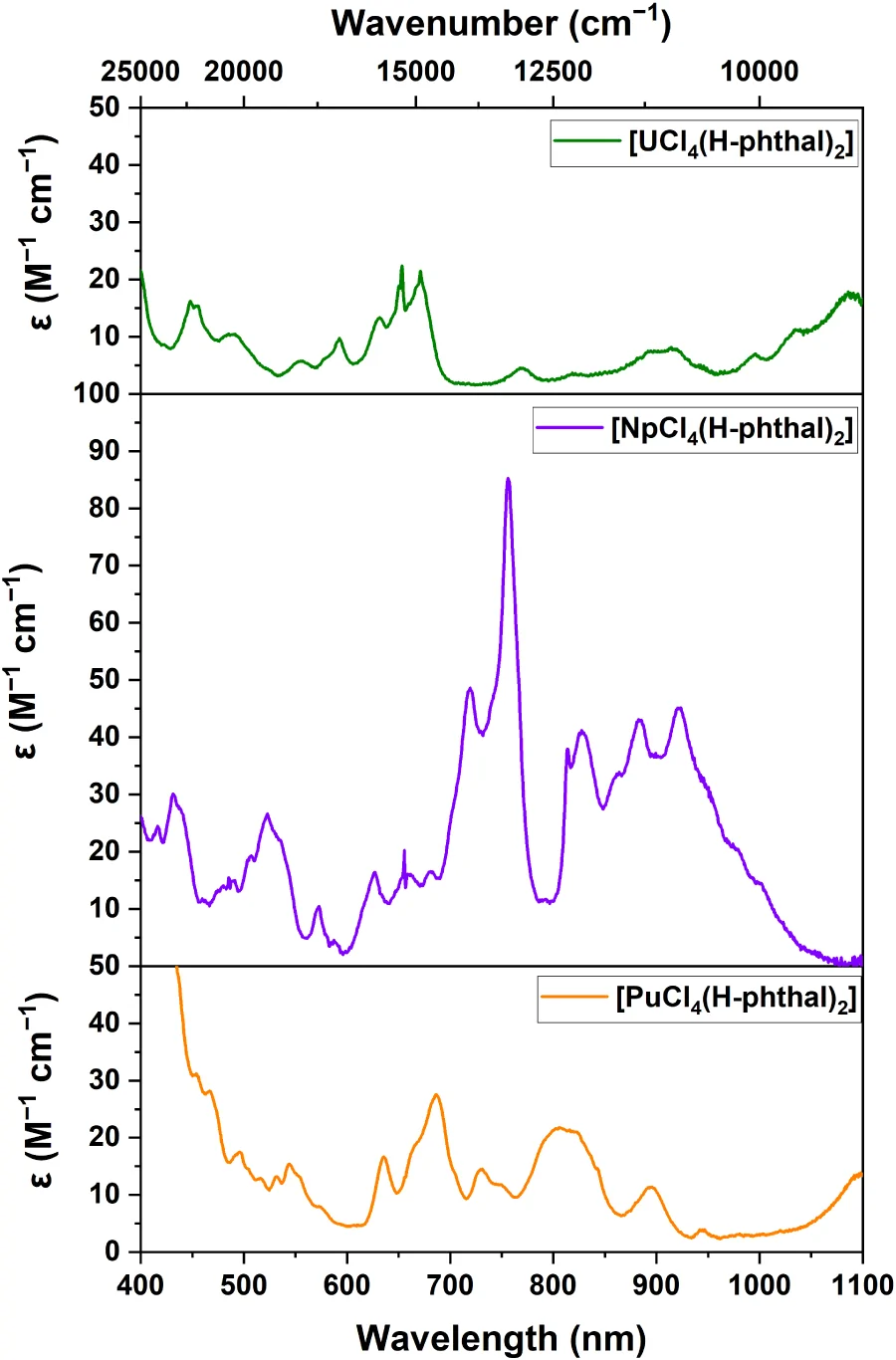
Vis-NIR spectra of [AnCl_4_(H-phthal)_2_] complexes in NCMe solution between 400 and 1100 nm at ambient temperature.

### Magnetic Properties and Electronic Structure

The confirmation of the +IV oxidation state by HERFD-XANES and Vis-NIR spectroscopy provides the basis for further analysis of the electronic structure and the magnetic properties of the [AnCl_4_(L)_2_] complexes. The complexes were therefore analyzed using direct current – Superconducting Quantum Interference Device (dc-SQUID) magnetometry. The results are shown in Figure 10, as plots of effective magnetic moment (μ_eff_) versus temperature. All complexes show paramagnetic behavior over the whole temperature range from 3.5 to 300 K as would be expected for *f* ^2^, *f* ^3^, and *f* ^4^ systems, respectively. The respective pairs of H-phthal and HPyO complexes exhibit very similar curves due to their similar molecular structure.

**Figure 10:**
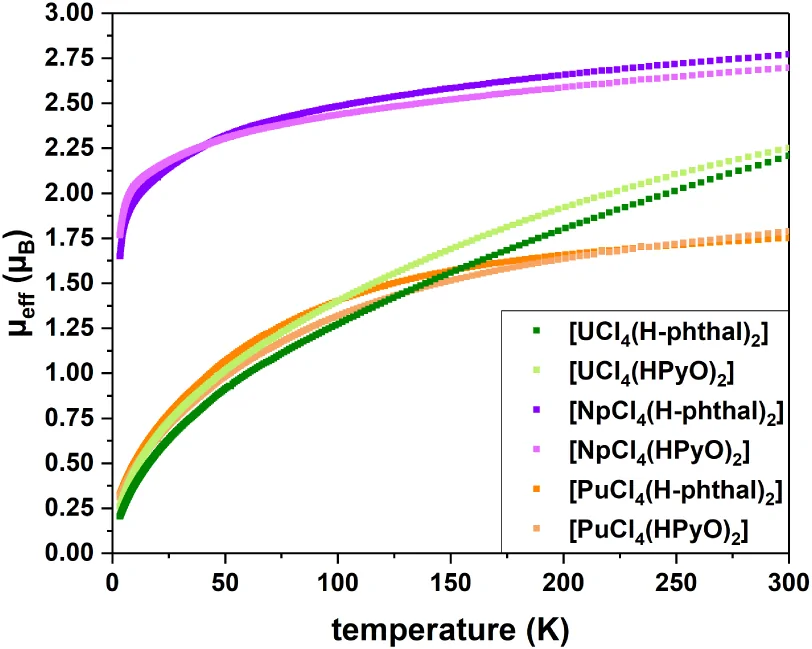
Experimental effective magnetic moment (in μ_B_) vs temperature (in K) data for [AnCl_4_(L)_2_] complexes from SQUID magnetometry at 3.5 T.

Upon cooling, both the U^IV^ (5*f* ^2^) and the Pu^IV^ (5*f* ^4^) complexes exhibit a decreasing magnetic moment tending toward zero. The Np^IV^ complexes on the other hand (*f* ^3^) retain an effective magnetic moment of 1.6 (L = H-phthal) or 1.8 μ_B_ (L = HPyO) at 3.5 K due to their odd number of electrons. With increasing temperature, the effective magnetic moment of the samples increases, with the Np and Pu complexes approaching values of ∼2.7 (Np) and ∼1.8 μ_B_ (Pu) at 300 K. In contrast, the magnetic moment of the [UCl_4_(L)_2_] complexes increases much beyond their room temperature values (Figure S61).

In order to get an in-depth description of the electronic configuration and magnetism of these complexes, quantum chemical calculations at N-electron valence state perturbation theory (CASSCF-NEVPT2) level including spin–orbit coupling (SOC, via QDPT (quasi-degenerate perturbation theory)) were performed. Including only the 5*f* orbitals in the active space was sufficient to reproduce the experimentally observed μ_eff_-*T*-plots (Figures S62–S64, *top*), with a somewhat more substantial deviation from the experiment for the complexes [PuCl_4_(H-phthal)_2_] and [PuCl_4_(HPyO)_2_]. However, μ_eff_ and the magnetization (Figures S62–S64, *middle*) of the two Pu complexes are well reproduced by the NEVPT2 calculations at low temperature (3.5 K). The experimentally obtained magnetic data was used to determine the Wybourne ligand field splitting parameters, $B_{q}^{k}$ , using the software package CONDON 3.0.^75^ The program considers a “full model” ligand field theory Hamiltonian taking all relevant aspects for the magnetic properties into account, including electron–electron interactions (*H*_*ee*_), spin–orbit coupling (*H*_*so*_) and ligand field interactions (*H*_*lf*_). The spin–orbit coupling constant (ζ) was taken from NEVPT2 and the Slater–Condon parameters (*F* ^2^, *F* ^4^, and *F* ^6^) were calculated with the *ab initio* ligand field theory (AILFT) on top of the NEVPT2 wave function. Leaving these parameters constant, CONDON 3.0 was used to fit the magnetic data to extract ligand field parameters with the Levenberg–Marquardt algorithm implemented in the code.

As starting values, ligand field parameters for [UCl_6_]^2–^ from the literature^11^ were used. Its octahedral geometry requires only two independent crystal field parameters, $B_{0}^{4}$ and $B_{0}^{6}$ , and two which are dependent on these, $B_{4}^{4}$ = (5/14)^1/2^ $B_{0}^{4}$ and $B_{4}^{6}$ = −(7/2)^1/2^ $B_{0}^{6}$ . The [AnCl_4_(L)_2_] complexes have approximately *D*_4h_ symmetry in their first coordination sphere, which removes the strict relation between these parameters and adds $B_{0}^{2}$ representing a stretching (negative $B_{0}^{2}$ ) or compression (positive $B_{0}^{2}$ ) of the octahedron.^76^ Based on the Point Charge Model (PCM), the structural similarity between the octahedral hexachloro dianions and the [AnCl_4_(L)_2_] complexes suggests similar signs of the $B_{q}^{k}$ values, with a positive sign of the additional $B_{0}^{2}$ . In our first approach, an *O*_h_ symmetry was assigned to fit $B_{0}^{4}$ and $B_{0}^{6}$ and the resulting ligand field parameters were used as starting values for an additional refinement in *D*_4h_. However, this approach did not lead to a similar set of ligand field parameters for all complexes, but much higher values for Np than U or Pu with also differences in signs and inconsistencies within complex pairs with the same actinide. The results were also highly dependent on the starting parameters.

To overcome this problem, a quasi-Monte Carlo approach for varying the $B_{q}^{k}$ parameters with defined signs from the PCM model was developed.^77^ Here, we start from a broad range for $B_{q}^{k}$ = ±15,000 cm^–1^ with the respective signs taken from PCM. From each $B_{q}^{k}$ parameters set, the fit quality against the experimental data (root-mean-square error, SQX) was extracted. CONDON 3.0 also gives the energy of the microstates (numbers of microstates for *f* ^2^ = 91, *f* ^3^ = 364, *f* ^4^ = 1001), which were compared against the respective SOC state energies obtained by NEVPT2. From Boltzmann population analysis it was clear that only the first nine states for U^IV^ and Pu^IV^ and five doubly degenerate states for Np^IV^ are populated. From the quasi-Monte Carlo sampling each $B_{q}^{k}$ parameter set was given a fit quality by normalization of SQX and normalization of the energy difference (ΔE_i_) of the respective microstate and SOC state (ΔE_i_ = |E_C,i_ – E_N,i_|, E_C,i_ is the energy of the *i* ^th^ microstate given in CONDON 3.0, E_N,i_ is the energy of the *i* ^th^ SOC state obtained from NEVPT2). A fit variable (*fit*) was defined following eq 1, which weights the experimental fit quality of the magnetic data (SQX) by 70% and the energy difference (Δ*E*_*i*_) of all selected exited states by 30%.

1 $$fit=\frac{SQX-SQX_{min}}{SQX_{max}-SQX_{min}}\times 0.7+\frac{0.3}{n-1}\times \overset{i=n}{\underset{2}{\sum }}\frac{\Delta E_{i}-{\Delta E_{i}}_{min}}{{\Delta E_{i}}_{max}-{\Delta E_{i}}_{min}}$$

The quasi-Monte Carlo sample is ordered by *fit* in decreasing order (sample size ∼16000). 10% of the sample was taken and new limits for the respective $B_{q}^{k}$ parameters were generated by averaging and addition or subtraction of the standard deviation. The quasi-Monte Carlo sampling was repeated with new limits until convergence was reached. The $B_{q}^{k}$ parameters obtained by this method are summarized in Table 4, plots of μ_eff_ *vs T* are given in Figures S62–S64 (*top*) and energy levels are given in Figures S62–S64 (*bottom*).

**Table 4:** Ligand Field Parameters $B_{q}^{k}$ for Complexes [AnCl_4_(L)_2_] Given in Wybourne Notation^a^

	**U**		**Np**		**Pu**	
L	H-phthal	HPyO	H-phthal	HPyO	H-phthal	HPyO
*F* ^2^	54293.5	53934.8	57294.6	57297.8	59918.6	59910.4
*F* ^4^	42675.8	42438.2	40586.7	40549.2	43519.1	43542.2
*F* ^6^	29994.2	29741.4	33196.6	33203.4	34757.2	34772.7
ζ	1908.92	1900.22	2184.51	2182.62	2471.74	2470.07
$B_{0}^{2}$	2901(53)	3522(37)	2448(109)	2359(121)	4434(128)	5140(148)
$B_{0}^{4}$	4936(122)	5498(156)	4633(91)	4683(85)	6070(199)	5857(216)
$B_{4}^{4}$	3448(78)	3203(45)	5287(72)	4660(84)	9498(96)	9162(122)
$B_{0}^{6}$	7710(158)	7624(166)	3933(81)	4382(104)	5335(85)	5216(128)
$B_{4}^{6}$	–8030(107)	–7478(45)	–4334(39)	–4789(35)	–10092(62)	–10366(120)
SQX_average_	0.013(1)	0.040(1)	0.034(1)	0.025(1)	0.103(1)	0.062(1)
Δ*E*_average_	171(6)	195(3)	161(5)	229(4)	479(7)	512(10)
^2*S*+1^ *L*_*J*_ (CONDON)	^3^ *H*_4_ (94.7)	^3^ *H*_4_ (94.4)	^4^ *I*_9/2_ (85.2)	^4^ *I*_9/2_(85.4)	^5^ *I*_4_ (75.8)	^5^ *I*_4_ (75.5)
	^1^ *G*_4_ (4.2)	^1^ *G*_4_ (4.7)	^4^ *F*_9/2_ (1.2)	^4^ *F*_9/2_ (1.3)	^5^ *G*_4_ (1.7)	^5^ *G*_4_ (1.8)
			^2^ *H*_9/2_ (12.0)	^2^ *H*_9/2_ (11.8)	^3^ *H*_4_ (16.3)	^3^ *H*_4_ (16.2)
			^2^ *G*_9/2_ (0.7)	^2^ *G*_9/2_ (0.6)	^3^ *G*_4_ (1.5)	^3^ *G*_4_ (1.5)
					^1^ *G*_4_ (1.2)	^1^ *G*_4_ (1.1)
^2*S*+1^ *L*_*J*_ (NEVPT2)	^3^ *H*_4_ (91.0)	^3^ *H*_4_ (91.2)	^4^ *I*_9/2_ (84.8)	^4^ *I*_9/2_ (84.8)	^5^ *I*_4_ (79.4)	^5^ *I*_4_ (79.4)
	^1^ *G*_4_ (7.9)	^1^ *G*_4_ (8.0)	^2^ *H*_9/2_ (12.1)	^2^ *H*_9/2_ (12.0)	^3^ *H*_4_ (15.8)	^3^ *H*_4_ (15.7)
			^2^ *G*_9/2_ (1.5)	^2^ *G*_9/2_ (1.5)	^3^ *G*_4_ (0.5)	^3^ *G*_4_ (0.6)
					^1^ *G*_4_ (0.8)	^1^ *G*_4_ (0.7)

a Slater-condon parameters and spin-orbit coupling constants were obtained from NEVPT2 calculations. All parameters are given in cm^–1^. Main Russell–Saunders terms (in %) for the ground state composition obtained with CONDON^78^ and NEVPT2.

These ligand field parameters are able to reproduce both the experimental trends of the μ_eff_ *vs T* data and the energy of the states in a satisfactory manner. The comparison of $B_{q}^{k}$ parameters between H-phthal and HPyO complexes show very similar values for each respective actinide. Along an actinide series, stronger deviations are visible. Various reasons can lead to such fluctuations, namely changes of An–Cl and An–O bond lengths and angles, bonding composition (ionic vs covalent bond contributions), or charge variation on actinide and ligand side, which would be overcompensated by the method. Comparisons with the literature are difficult due to the limited amount of available data for actinide complexes with *D*_4h_ symmetry. Ligand field parameters of *f*-element ions in isostructural solids obtained from optical measurements are usually very similar, although most studies have been limited to more ionic compounds such as AnF_4_ ^79^ or ions doped into host crystal lattices,^11^ in which above-mentioned effects play a less significant role. In an early study, marked deviations in $B_{q}^{k}$ values were also obtained for comparable PaX_6_^2–^ and UX_6_^2–^ compounds (X = F, Cl, Br, I), where no clear trend between Pa^IV^ and U^IV^ of the same halide complexes was observed.^10^ Overall, our derived parameters are in a realistic order of magnitude ranging from ±1000 to ±10000 cm^–1^ and thus significantly smaller than the Slater–Condon parameters *F* ^*k*^.

Based on the CONDON results, we were able to describe the ground state composition of each complex using the Russell–Saunders term (^2*S*+1^ *L*_*J*_), which are listed in Table 4.^75,78^ The obtained values are consistent within each of the actinide complexes showing only slight deviations between H-phthal and HPyO. In addition, the experimental values are well reproduced by NEVPT2 calculations. This consistency might seem surprising as the underlying methodology differs quite a bit in both approaches. The similarity is of course, that in both cases the ligand field and spin–orbit coupled eigenstates are expanded in a basis of free ion Russell–Saunders terms. In the NEVPT2 case, the parentage is obtained by projecting the SOC states onto the states derived from the free ion (Tables S36–S41). In CONDON, a semi empirical parameterization of the free ion, the ligand field and spin–orbit interactions are used. Despite the methodological differences, both approaches try to quantify the same objects, namely how the SOC eigenstates derive from the free ion terms. Interestingly, the degree of mixing increases significantly along the actinide series. For uranium, the ground state was found to consist of ∼95% ^3^ *H*_4_ with a small contribution of ∼4% ^1^ *G*_4_. This mixing is smaller than observed in U^IV^:CaF_2_, where the ground state was found to contain 88% ^3^ *H*_4_ and 11% ^1^ *G*_4_,^80^ or 90% ^3^ *H*_4_ and 8% ^1^ *G*_4_ in pure UF_4_, obtained from spectroscopic and magnetic data.^75,79,81^ The mixing increases when going to Np and Pu, where the nominal ground states of ^4^ *I*_9/2_ (Np^IV^) or ^5^ *I*_4_ (Pu^IV^) are only contributing with approximately 85% and 76%, respectively. Spectroscopic analyses in the literature have yielded similar results with only about 80% ^4^ *I*_9/2_ character in Np^IV^:ThO_2_.^82,83^ For Pu, such analyses have so far not been performed, but the contributing states are similar to isoelectronic Np^III^ cases.^84^

## Conclusion

We have presented two series of octahedral actinide complexes [AnCl_4_(L)_2_] (An = U, Np, Pu) with L being the small, monodentate O-donor ligands phthalimidine and 2-pyridone. Despite their small size, these ligands stabilize the actinide ions in their low coordination number of six, with four chloride ligands in the equatorial plane and the organic ligands in axial *trans* position. HERFD-XANES spectroscopy at the An-M_4_ edges confirmed the tetravalent oxidation state in all cases, which is also supported by Vis-NIR spectroscopy. Solvent molecules (THF), which are bound via hydrogen bonds at the NH moiety of the amide ligands, can be removed easily in CH_2_Cl_2_ yielding solvent-free complexes. There, the strong polarization of the NH unit leads to strong distortions due to intermolecular H bonding with neighboring Cl ligands, which in combination with packing effects leads to the shortest Np–Cl bond observed so far. QTAIM analyses of the complexes reveal the strongest covalent contributions for the An–Cl bonds while the oxygen donors exhibit predominantly ionic interactions. In solution, this leads to a high flexibility of these ligands as shown by NMR spectroscopy and quantum chemical calculations.

Finally, these complexes, with their idealized *D*_4h_ symmetry in the first coordination sphere, were used for analysis of their electronic structure by SQUID magnetometry in combination with quantum chemical calculations. A new quasi-Monte Carlo approach was successfully applied taking both the SQUID magnetic data and the calculated electronic splitting (SOC states) into account. Sets of Wybourne ligand field parameters $B_{q}^{k}$ were derived for all complexes for the first time in *D*_4h_ symmetry for actinide complexes, showing consistent values within each of the actinides. Based on these ligand field parameters, the ground state compositions of the An^IV^ ions could be assigned experimentally and supported by theoretical calculations showing an increasing mixing of states from U^IV^ to Pu^IV^. Our methodology opens up the possibilities to provide access to more reliable ligand field parameters through combination of magnetic and theoretical data in systems with lower local symmetry around the actinide center and therefore a better understanding of their electronic structure.

## Supplementary Material



## Notes

Additional experimental details, synthesis, IR spectra, SC-XRD structures, Cartesian coordinates of optimized molecular structures, reaction path calculations, NMR spectra, SQUID data, and spin–orbit parentage analysis (PDF)
