# Supplementary material for: Hexacoordinate Actinide(IV) Compounds: Benchmark Complexes for Magnetism and Electronic Structure Analysis
Source: J Am Chem Soc. 2026 Aug 28;148(41):44156–71. doi: 10.1021/jacs.6c09492 (PMC13693446; doi:10.1021/jacs.6c09492)
Supplement: Supplementary file 1 [file ja6c09492_si_001.pdf]

Supporting Information for:

Hexacoordinate Actinide(IV) Compounds: Benchmark Complexes for Magnetism and Electronic Structure Analysis

*Till Sawallisch,[a] Dennis Grödler,[a] Adrian Näder,[a] Clara L. Silva,[a,b] Peter Kaden,[a] Michael Patzschke,[a] Lucia Amidani,[a,b] Kristina O. Kvashnina,[a,b] Solveig Pospiech,[a] Jan van Leusen,[c] Moritz Schmidt,[a,d] Thorsten Stumpf,[a] Robert Gericke[a]**

[a] Helmholtz-Zentrum Dresden – Rossendorf, Institute of Resource Ecology, Bautzner Landstraße 400, 01328 Dresden, Germany.

[b] The Rossendorf Beamline at ESRF – The European Synchrotron, CS40220, 38043 Grenoble Cedex 9, France.

[c] RWTH Aachen University, Institute of Inorganic Chemistry, Landoltweg 1, 52074 Aachen, Germany.

[d] Brandenburg University of Technology Cottbus-Senftenberg, Institute of Materials Chemistry, 01968 Senftenberg, Germany.

E-Mail: r.gericke@hzdr.de

Table of Contents

Experimental Section **S4**

Single-Crystal X-ray Diffraction (SC-XRD) S4

Nuclear Magnetic Resonance (NMR) Spectroscopy S4

IR Spectroscopy S4

High Energy-Resolution Fluorescence-Detected X-ray Spectroscopy S4

Superconducting Quantum Interference Device Magnetometry Measurements S5

Visible-Near Infrared (Vis-NIR) Spectroscopy in solution S5

Quantum Chemical Calculations S5

Synthesis **S6**

$[UCl_4(phthalimidine)_2]$ S6

$[NpCl_4(phthalimidine)_2]$ S7

$[PuCl_4(phthalimidine)_2]$ S8

$[UCl_4(2\text{-}pyridone)_2]$ S9

$[NpCl_4(2\text{-}pyridone)_2]$ S10

$[PuCl_4(2\text{-}pyridone)_2]$ S11

IR spectra **S13**

SC-XRD Structures **S17**

Cartesian coordinates (in Å) of THF-free molecular structures with idealized D_{4h} symmetry in the first coordination sphere of the actinides **S26**

Reaction path calculations **S30**

Cartesian coordinates (in Å) of reactants and hypothetical solvent-coordinated product complexes from reaction path calculations performed via *woelfling* S32

NMR Characterization of $[AnCl_4(H\text{-}phthal)_2]$ complexes **S46**

$[UCl_4(H\text{-}phthal)_2]\cdot 2THF$ S46

$[UCl_4(H\text{-}phthal)_2]$ S48

$[NpCl_4(H\text{-}phthal)_2]$ S49

$[PuCl_4(H\text{-}phthal)_2]$ S51

NMR Characterization of $[AnCl_4(HPyO)_2]$ complexes **S54**

$[UCl_4(HPyO)_2]\cdot 2THF$ S54

$[NpCl_4(HPyO)_2]$ S56

$[PuCl_4(HPyO)_2]$ S58

Cartesian coordinates (in Å) of re-optimized GOAT conformers of $[UCl_4(H\text{-}phthal)_2]$ **S61**

Superconducting Quantum Interference Device (SQUID) magnetization measurements **S65**

Spin–orbit parentage analysis **S69**

$[UCl_4(H\text{-}phthal)_2]$ S71

$[UCl_4(HPyO)_2]$ S75

[$NpCl_4$(H-phthal)$_2$] S79
[$NpCl_4$(HPyO)$_2$] S84
[$PuCl_4$(H-phthal)$_2$] S89
[$PuCl_4$(HPyO)$_2$] S96
Literature S104

Experimental Section

Caution! The early actinides thorium, uranium, neptunium, and plutonium contain exclusively radioactive isotopes, including long-lived alpha emitters: ^{232}Th ($t_{1/2}$ = 1.41·10^{10} a), ^{235}U ($t_{1/2}$ = 7,04·10^{8} a), ^{238}U ($t_{1/2}$ = 4,47·10^{9} a), ^{237}Np ($t_{1/2}$ = 2,14·10^{6} a) and ^{242}Pu ($t_{1/2}$ = 3,75·10^{5} a).[1] Special safety requirements are necessary for the safe handling of radioactive substances. These include certified laboratories with appropriate equipment. All experiments were carried out in the controlled laboratory at the Institute of Resource Ecology, Helmholtz-Zentrum Dresden – Rossendorf.

All manipulations and reactions were carried out under careful exclusion of moisture and oxygen in nitrogen filled glove boxes or using Schlenk techniques. All solvents were dried and distilled from Na/benzophenon or calcium hydride and stored over activated 3 Å molecular sieves prior to use. The actinide starting materials UCl_4,[2] [$NpCl_4$(DME)$_2$] (DME = dimethoxyethane) and [$PuCl_4$(DME)$_2$][3] were prepared according to literature procedures. Other chemicals were used as received without further purification.

Single-Crystal X-ray Diffraction (SC-XRD): Single crystals of compounds were placed in mineral oil, cut, mounted on a 100 µm MiTeGen Dual Thickness MicroMounts and located in the temperature-controlled N_2 gas flow at 100 K from Oxford instruments. The data was collected on a Bruker D8 VENTURE diffractometer equipped with a Photon II 7 array detector and micro focus Mo K_α radiation (λ = 0.71073 Å) with mirror optics monochromator. The computer programs SMART and SAINT, which are implemented in the APEX4 software suite,[4] were used for data collection in ϕ- and ω-scan modes and data processing, respectively. Absorption correction was performed using SADABS.[5] All structures were solved using ShelXT and all atoms were refined with full-matrix least-squares methods on F^2 using the SHELX-TL package.[6, 7] ORTEP-3[8] was used for visualization.

Nuclear Magnetic Resonance (NMR) Spectroscopy: NMR spectra were recorded on an Agilent MR 400 spectrometer with a ^{1}H frequency of 401.74 MHz and a ^{13}C frequency of 100.03 MHz. All spectra were recorded on an Agilent ONE probe head with z gradient. NMR experiments are performed using J Young Teflon valve-sealed NMR tubes. Deuterated solvents were purchased at Deutero GmbH and dried over Na/benzophenone or CaH_2 and stored under 3 Å molecular sieve prior to use. Chemical shifts for ^{1}H and ^{13}C NMR are referenced and reported to internal reference tetramethylsilane (TMS, 0 ppm) or to the solvent used. Coupling constants (J) are reported in Hertz (Hz), and splitting patterns are indicated as s (singlet), d (doublet), t (triplet), m (multiplet) and br (broad). ^{1}H and ^{13}C NMR spectra were recorded directly and signals of ^{1}H and ^{13}C were correlated for all complexes using two-dimensional ^{1}H,^{1}H COSY, ^{1}H,^{13}C HSQC, and ^{1}H,^{13}C HMBC spectra.

IR Spectroscopy: IR spectra were measured on an Agilent Cary 630 FT-IR spectrometer equipped with a single-reflection attenuated total reflection (ATR) accessory made of diamond. The measurements were performed in an inert glove box with N_2 atmosphere. Solid samples were pressed onto the measuring crystal with a screw stamp. Spectra were recorded between 4000 and 650 cm^{-1}.

High Energy-Resolution Fluorescence-Detected X-ray Spectroscopy: The solid samples of the An complexes were placed on the adhesive surfaces of the sample holders and were sealed with Kapton foil. To prevent humidity and oxygen from affecting the samples, a Dewar container with liquid nitrogen or an inert gas container with a nitrogen atmosphere was used as a transport container. The X-ray absorption spectra were recorded at the ROBL beamline of the ESRF in Grenoble, France,[9] under cryo-conditions with a cryostream. The incident energy was tuned to the An M_4 edges with a fixed-exit Si(111) double crystal monochromator. The suppression of higher harmonics was achieved by the total reflection from a Si mirror at 2.5 mrad. XANES spectra were measured in HERFD mode using an X-ray emission spectrometer[10] with the sample, analyzer crystal, and photon detector (Ketek detector) arranged in a vertical *Rowland* geometry. A set of 5 Si(220) bent-striped crystal analyzers was used to detect the M_4 emission lines of U, Np and Pu.

Superconducting Quantum Interference Device Magnetometry Measurements: Temperature-dependent measurements (3.5–300 K) were carried out on an MPMS3 SQUID magnetometer from Quantum Design. Raw data were recorded as SQUID voltage vs sample position. Kel-F sample holders were attached to quartz glass holders with Ge-Varnish. Analog measurements (35000 Oe, 30 mm sample movement) were carried out for the blank (empty sample holder) and the actinide complexes. The SquidLab program (version 2.9.1) was used for background correction and data adjustment using the Levenberg–Marquardt method.[11] A Pd standard was used to determine the calibration factor.

Visible-Near Infrared (Vis-NIR) Spectroscopy in solution: Electronic absorption spectra in MeCN solutions were collected in 1 cm quartz cuvettes using an AIS DT2000 Deuterium-Tungsten Light Source and an Avantes AvaSpec-ULS2048x64-EVO detector connected with fiber optic cable through the glovebox. AvaSoft 8 software was used to determine integration times, collecting background and dark spectra.

Quantum Chemical Calculations: Bond analysis was performed from the optimized structures as described in the main text. The CASSCF(n,7)-SOC-NEVPT2 calculations for predicting energies of the SOC states, susceptibility and magnetization behavior were performed using Orca 6.0.1. Prior, the molecular structures of the form [$AnCl_4(L)_2$] were optimized with restriction of the first coordination sphere of the actinide in an idealized D_{4h} symmetry (Table S5-S10). Scalar-relativistic effects were treated explicitly within the Douglas-Kroll-Hess formalism and appropriate basis sets were used (DKH-DEF2-TZVPP, SARC-DKH-TZVPP(An)). As an example, for [$NpCl_4$(H-phthal$)_2$] the final total energy for idealized D_{4h} symmetry in the first coordination sphere is about 26 kcal/mol lower in energy as from the solid-state structure where the H atom positions are optimized. The prediction of magnetic data for molecular complexes is dependent on the underlying structure. For the state averaged (SA) CASSCF(n,o) setup (n = active 5f electrons, o = active 5f orbitals) of U:CAS(2,7), Np:CAS(3,7) and Pu:CAS(4,7), all possible configuration state functions (roots) were included, amounting to 21 triplets and 28 singlets for U, 35 quartets and 112 doublets for Np, and 35 quintets, 210 triplets and 196 singlets for Pu. Spin-orbit coupling was included by state interaction of the resulting spin-free states via quasi-degenerate perturbation theory (QDPT). Strongly contracted NEVPT2 was performed on top to address dynamical electron correlation effects. The calculated magnetic data obtained from structures with idealized D_{4h} symmetry (Table S5-S10) exhibits the best match between CASSCF-NEVPT2 and experiment and are therefore reported in Figure S61-S64.

Synthesis

[UCl_4(phthalimidine)$_2$]: Phthalimidine (7.6 mg, 0.057 mmol, 2 eq.) and UCl_4 (10.5 mg, 0.028 mmol, 1 eq.) were dissolved in CH_2Cl_2 or THF (0.2 ml), respectively. Both solutions were centrifuged to remove insoluble impurities. The ligand solution was transferred to a vial and layered first with CH_2Cl_2, then with THF (0.5 ml each). Finally, the UCl_4 solution was layered on top. The vial was left to stand for 12 h, upon which pale green crystals were formed. The solution was decanted, the crystals were washed with THF (3 × 0.25 ml) and dried under reduced pressure for 1 h. Yield: 13.2 mg, 0.017 mmol, 60%. The structure was confirmed by SC-XRD as [UCl_4(phthalimidine)$_2$]·2THF. The THF solvent molecules were removed by stirring the crystals in CH_2Cl_2 (0.5 ml) for 30 min. Solvent-free crystals suitable for SC-XRD analysis were obtained from MeCN solution in a J. Young NMR tube.

^{1}H-NMR (CD_2Cl_2, 401.7 MHz, 298 K): δ = 9.84 (br s, 2H, NH), 7.76 (t, 2H, H5), 7.66 (d, 2H, H4), 7.24 (t, 2H, H6), 6.81 (br s, 2H, H7), 3.06 (s, 4H, H3) ppm.

^{1}H-NMR (CD_3CN, 401.7 MHz, 338 K). δ = 10.40 (br s, 2H, NH), 8.16 (br, 2H, H7), 7.87 (t, $^{3}J_{HH}$ = 7.23, 7.39, 2H, H5), 7.71 (d, $^{3}J_{HH}$ = 7.35 Hz, 2H, H4), 7.59 (t, $^{3}J_{HH}$ = 6.79 Hz, 6.95, 2H, H6), 3.10 (s, 4H, H3) ppm.

^{13}C-NMR (CD_3CN, 101.03 MHz, 338 K): δ = 194.9 (C1), 145.4 (C3a), 135.0 (C7a), 134.4 (C5), 130.3 (C6), 125.8 (C4), 124.7 (C7), 53.1 (C3) ppm.

IR (ATR, THF adduct): $\tilde{\nu}$ = 677 (w), 727 (vs), 736 (vs), 792 (vs), 845 (m), 879 (m), 920 (w), 1046 (m), 1101 (w), 1148 (w), 1178 (w), 1206 (m), 1276 (w), 1306 (w), 1368 (w), 1410 (s), 1441 (m), 1474 (m), 1583 (s), 1612 (vs), 1626 (s), 2748 (w), 2924 (w) cm^{-1}.

IR (ATR, solvent-free): $\tilde{\nu}$ = 687 (s), 734 (vs), 793 (s), 1026 (m), 1207 (m), 1418 (m), 1442 (s), 1478 (m), 1587 (s), 1618 (vs), 1631 (s), 3328 (w, br) cm^{-1}.

Vis-NIR (CH_3CN, 15 mM): λ (ε) = 448 (16), 453 (15), 498 (10), 554 (6), 593 (10), 632 (13), 653 (22), 671 (21), 678 (5), 818 (4), 892 (8), 915 (8), 996 (7), 1037 (11), 1086 (18) nm (M^{-1} cm^{-1}).

Vis-NIR (solid state): λ = 393, 448, 475, 488, 518, 567, 574, 585, 620, 627, 638, 645, 653, 660, 667, 676, 755, 806, 813, 879, 1054 nm.

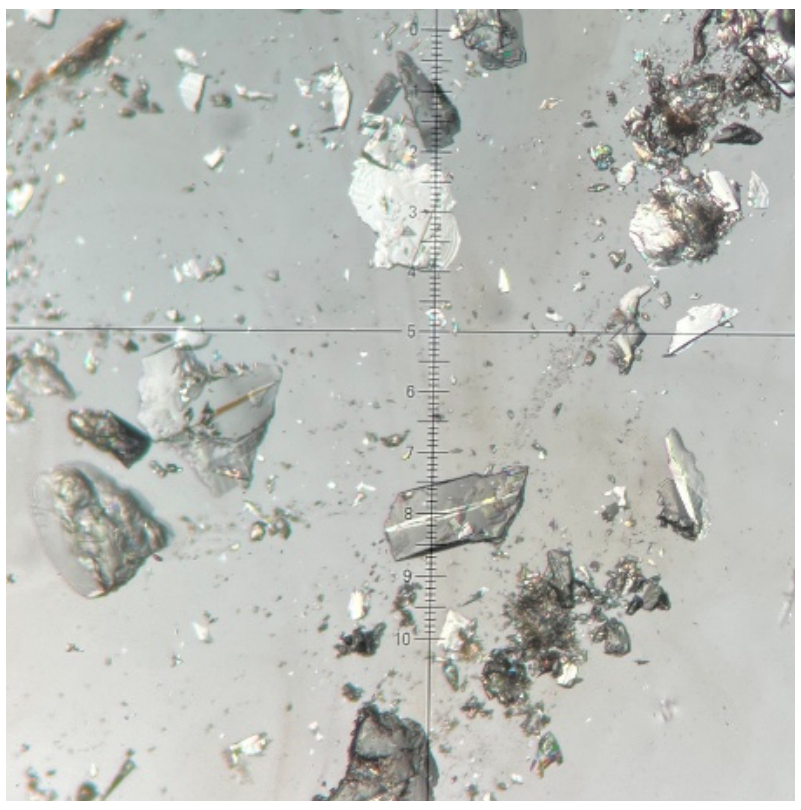

Figure S1. Crystals of [UCl_4(H-phthal)$_2$]·2THF.

[$NpCl_4$(phthalimidine)$_2$]: [$NpCl_4(DME)_2$] (10 mg, 0.018 mmol, 1 eq.) and Phthalimidine (4.9 mg, 0.037 mmol, 2.1 eq.) were dissolved separately in THF (0.2, and 0.3 mL, respectively). Both solutions were centrifuged to remove possible insoluble impurities. The pink Np solution was filled in a vial and layered first with 0.5 ml THF and then with the ligand solution. The vial was closed and left to stand for three days, upon which grey-colorless solids formed at the bottom of the vial. The solids were isolated and washed with THF (2 × 0.25 mL). Yield: 8.1 mg, 0.010 mmol, 57%. Crystals suitable for SC-XRD analysis were obtained from a CD_2Cl_2/THF mixture in a J. Young NMR tube. The THF solvent molecules were removed by stirring the crystals in CH_2Cl_2 (0.5 ml) for 30 min. Solvent-free crystals suitable for SC-XRD analysis were obtained from MeCN solution in a J. Young NMR tube.

^{1}H-NMR (CD_2Cl_2, 401.7 MHz, 298 K): δ = 9.20 (br s, 2H, NH), 6.76 (br s, 2H, CH), 6.59 (br s, 2H, CH), 5.71 (br s, 4H, CH), −0.15 (br s, 4H, -CH_2-) ppm.

^{1}H-NMR (CD_3CN, 401.7 MHz, 338 K). δ = 7.92 (br s, 2H, NH), 7.03 (br t, 2H, H5), 6.78 (br t, 2H, H6), 6.51 (br d, 2H, H4), 5.08 (br, 2H, H7), −0.10 (s, 4H, H3) ppm.

^{13}C-NMR (CD_3CN, 101.03 MHz, 338 K): δ = 144.4 (C1), 140.7 (C3a), 132.9 (C5), 128.9 (C7a), 128.7 (C6), 124.0 (C4), 121.3 (C7), 51.6 (C3) ppm.

IR (ATR, THF adduct): $\tilde{\nu}$ = 677 (w), 727 (vs), 736 (vs), 792 (vs), 845 (w), 882 (m), 919 (w), 1017 (vw), 1046 (s), 1085 (w), 1149 (w), 1174 (w), 1206 (m), 1306 (w), 1369 (w), 1412 (s), 1422 (s), 1442 (s), 1474 (m), 1585 (m), 1614 (vs), 1627 (vs), 2755 (w), 2875 (w), 2917 (w) cm^{-1}.

IR (ATR, solvent-free): $\tilde{\nu}$ = 686 (s), 733 (vs), 791 (s), 1017 (m), 1025 (m), 1207 (m), 1304 (w), 1347 (w), 1417 (m), 1442 (s), 1477 (m), 1586 (s), 1612 (vs), 1627 (s), 3328 (w, br) cm^{-1}.

Vis-NIR (CH_3CN, 9.3 mM): λ (ε) = 416 (24), 431 (30), 485 (16), 507 (19), 522 (27), 535 (22), 572 (10), 627 (16), 658 (16), 683 (16), 719 (49), 756 (85), 814 (38), 828 (41), 864 (34), 883 (43), 921 (45) nm (M^{-1} cm^{-1}).

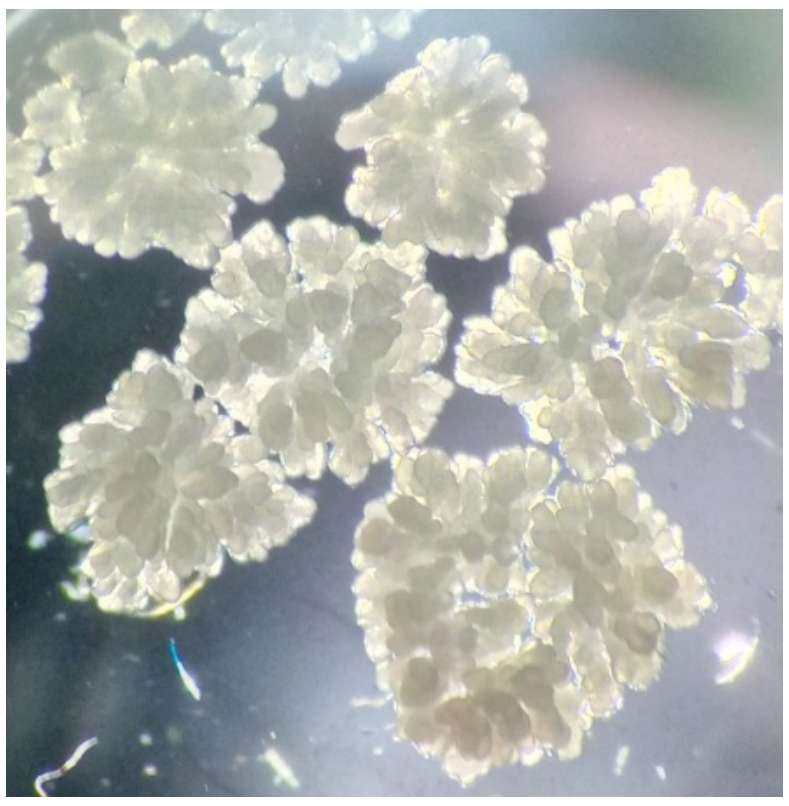

Figure S2. Solids of [$NpCl_4$(H-phthal)$_2$]·2THF as obtained from the reaction mixture.

[$PuCl_4$(phthalimidine)$_2$]: [$PuCl_4$(DME)$_2$] (15 mg, 0.027 mmol, 1 eq.) and Phthalimidine (7.1 mg, 0.053 mmol, 2 eq.) were dissolved separately in THF (0.75, and 0.5 mL, respectively). Both solutions were centrifuged to remove possible insoluble impurities. The yellow Pu solution was filled in a vial and layered first with 0.5 ml THF and then with the ligand solution. The vial was closed and left to stand for three days, upon which pale yellow-brown solids formed at the bottom of the vial. The solids were isolated and washed with *n*-pentane (2 × 0.5 mL). Yield: 11.5 mg, 0.014 mmol, 52%. Crystals suitable for SC-XRD analysis were obtained from a CD_2Cl_2/THF mixture in a J. Young NMR tube. The THF solvent molecules were removed by stirring the crystals in CH_2Cl_2 (0.5 ml) for 30 min.

^{1}H-NMR (CD_2Cl_2, 401.7 MHz, 298 K): δ = 9.70 (br s, 2H, NH), 7.52 (t, 2H, CH_{ar}), 7.37 (d, 2H, CH_{ar}), 6.98 (t, 2H, CH_{ar}), 5.83 (d, 2H, CH_{ar}), 2.58 (s, 4H, -CH_2-) ppm.

^{13}C-NMR (CD_2Cl_2, 101.03 Hz, 298 K): 133.6 (CH_{ar}), 128.8 (CH_{ar}), 123.9 (CH_{ar}), 123.2 (CH_{ar}), 50.1 (-CH_2-) ppm. Signals of the quarternary carbon atoms could not be detected.

^{1}H-NMR (CD_3CN, 401.7 MHz, 338 K): δ = 8.49 (br s, 2H, NH), 7.61 (dd, $^3J_{HH}$ = 7.39, 7.55 Hz, 2H, H5), 7.44 (d, $^3J_{HH}$ = 7.23 Hz, 2H, H4), 7.27 (dd, $^3J_{HH}$ = 7.15, 7.07 Hz, 2H, H6), 6.78 (br, 2H, H7), 2.80 (s, 4H, H3) ppm.

^{13}C-NMR (CD_3CN, 101.03 Hz, 338 K): 143.8 (C3a), 134.1 (C5), 130.0 (C7a), 129.2 (C7a), 129.2 (C6), 124.5 (C4), 124.1 (C7), 51.1 (C3) ppm.

IR (ATR, THF adduct): $\tilde{\nu}$ = 677 (w), 727 (vs), 736 (vs), 792 (vs), 845 (w), 882 (m), 919 (w), 1017 (vw), 1046 (s), 1085 (w), 1101 (w), 1149 (w), 1173 (w), 1206 (m), 1306 (w), 1411 (s), 1421 (s), 1442 (s), 1474 (m), 1584 (s), 1612 (vs), 1626 (s), 2755 (w), 2873 (w) cm^{-1}.

IR (ATR, solvent-free): $\tilde{\nu}$ = 686 (s), 733 (vs), 792 (s), 1025 (m), 1207 (m), 1418 (m), 1442 (s), 1477 (m), 1587 (s), 1615 (vs), 1628 (s), 3329 (w, br) cm^{-1}.

Vis-NIR (CH_3CN, 1.5 mM): λ (ε) = 453 (31), 467 (28), 496 (18), 516 (13), 531 (13), 544 (15), 636 (17), 686 (28), 731 (15), 749 (12), 790–825 (22), 894 (11), 1101 (14) nm (M^{-1} cm^{-1}).

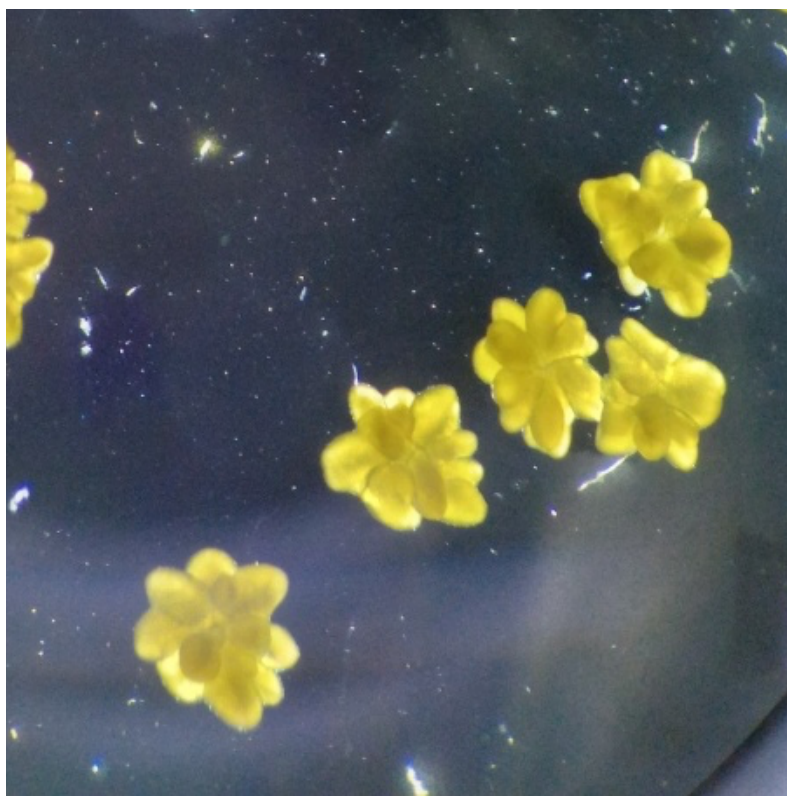

Figure S3. Solids of [$PuCl_4$(H-phthal)$_2$]·2THF as obtained from the reaction mixture.

[UCl_4(2-pyridone)$_2$]: 2-pyridone (15 mg, 0.158 mmol, 2 eq.) and UCl_4 (20.0 mg, 0.053 mmol, 1 eq.) were dissolved in 0.5 ml THF, respectively. The uranium containing solution was then carefully layered with the ligand containing solution. The vial was left to stand for 18 h, upon which colorless crystals formed. The solution was evaporated and the crystals were washed with 0.5 ml THF. Suitable single crystals were then measured by SC-XRD. The structure was confirmed by SC-XRD as [UCl_4(2-pyridone)$_2$]·2THF. After washing the solids with 0.5 ml DCM, [UCl_4(2-pyridone)$_2$] was obtained as a pale green powder. Yield: 16.5 mg, 0.029 mmol, 55%.

^{1}H-NMR (CD_3CN, 401.7 MHz, 338 K): δ = 17.44 (br s, 2H, NH), 12.71 (br d, 2H, CH), 10.35 (br t, 2H, CH), 9.90 (br d, 2H, CH), 9.00 (br t, 2H, CH) ppm.

^{13}C-NMR (CD_3CN, 101.03 MHz, 338 K): δ = 148.7 (CH), 141.1 (CH), 130.2 (CH), 116.13 (CH) ppm. Signals of the quarternary carbon atoms could not be detected.

IR (ATR): $\tilde{\nu}$ = 722 (s), 771 (vs), 879 (s), 1003 (m), 1021 (w), 1089 (m), 1157 (m), 1211 (w), 1270 (m), 1363 (s), 1451 (m), 1532 (s), 1587 (vs), 1625 (m), 1633 (m), 3158 (w), 3233 (w) cm^{-1}.

Vis-NIR (solid state): λ = 403, 444, 477, 492, 517, 567, 573, 581, 619, 627, 643, 658, 666, 673, 750, 799, 859, 887, 959, 1035 nm.

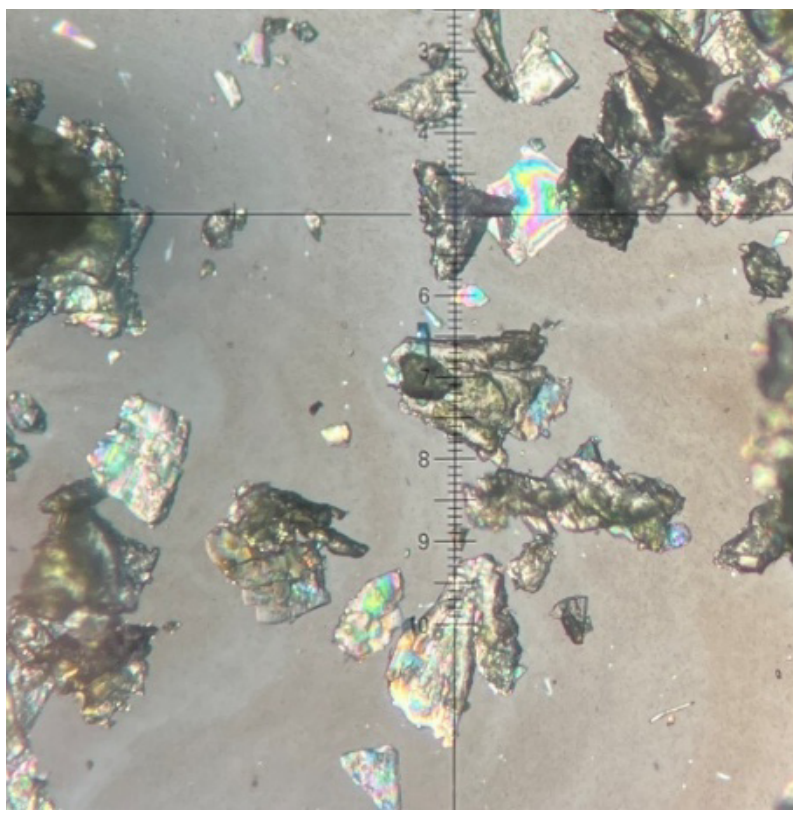

Figure S4. Crystals of [UCl_4(2-pyridone)$_2$]·2THF.

[NpCl$_4$(2-pyridone)$_2$]: 2-pyridone (6.8 mg, 0.072 mmol, 2 eq.) and [NpCl$_4$(DME)$_2$] (20.0 mg, 0.036 mmol, 1 eq.) were dissolved in 0.5 ml THF, respectively. The neptunium containing solution was then carefully layered with the ligand containing solution. The vial was left to stand for 18 h, upon which greyish crystals formed. The solution was evaporated and the crystals were washed with 0.5 ml THF. Suitable single crystals were then measured by SC-XRD. After washing the solids with 0.5 ml DCM [NpCl$_4$(2-pyridone)$_2$] was obtained as a pale grey powder. Yield: 9.4 mg, 0.017 mmol, 46%. The structure was confirmed by SC-XRD as [NpCl$_4$(2-pyridone)$_2$].

^{1}H-NMR (CD$_3$CN, 401.7 MHz, 338 K): δ = 13.12 (br s, 2H, NH), 8.66 (br s, 4H, CH), 8.21 (br s, 2H, CH), 8.02 (br s, 2H, CH) ppm.

^{13}C-NMR (CD$_3$CN, 101.03 MHz, 338 K): δ = 145.8 (CH), 140.1 (CH), 122.7 (CH), 114.5 (CH) ppm. Signals of the quarternary carbon atoms could not be detected.

IR (ATR): $\tilde{\nu}$ = 721 (s), 770 (vs), 822 (w), 878 (vs), 1002 (m), 1020 (w), 1088 (m), 1121 (w), 1157 (m), 1211 (m), 1268 (m), 1367 (vs), 1451 (m), 1531 (s), 1585 (vs), 1624 (m), 1633 (m), 3237 (w) cm^{-1}.

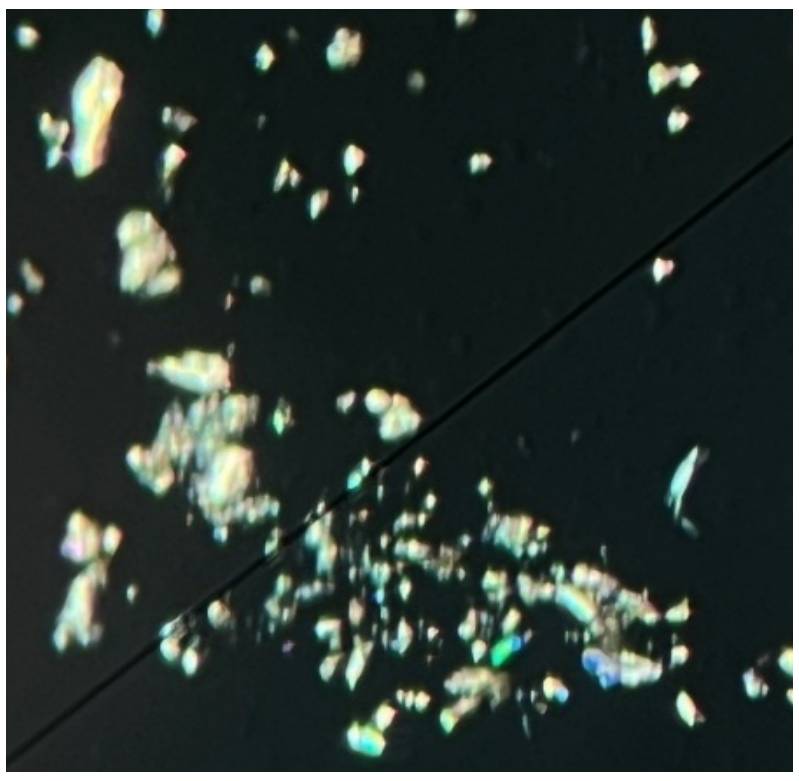

Figure S5. Crystals of [NpCl$_4$(2-pyridone)$_2$].

[$PuCl_4$(2-pyridone)$_2$]: 2-pyridone (5.1 mg, 0.053 mmol, 2 eq.) and [$PuCl_4$(DME)$_2$] (15.0 mg, 0.027 mmol, 1 eq.) were dissolved in 0.5 ml THF, respectively. The plutonium containing solution was then carefully layered with the ligand containing solution. The vial was left to stand for 48 h, upon which colorless crystals formed. The solution was evaporated and the crystals were washed with 0.5 ml THF. Suitable single crystals were then measured by SC-XRD. The structure was confirmed by SC-XRD as [$PuCl_4$(2-pyridone)$_2$]·2THF. After washing the solids with 0.5 ml DCM [$PuCl_4$(2-pyridone)$_2$] was obtained as a pale orange powder. Yield: 7.2 mg, 0.013 mmol, 57%.

^{1}H-NMR (CD_3CN, 401.7 MHz, 338 K): δ = 11.45 (br s, 2H, NH), 7.72 (br t, 2H, CH), 7.55 (br d, 2H, CH), 7.19 (br t, 2H, CH), 6.65 (br d, 2H, CH) ppm.

^{13}C-NMR (CD_3CN, 101.03 MHz, 338 K): δ = 144.3 (CH), 138.7 (CH), 122.7 (CH), 112.6 (CH) ppm. Signals of the quarternary carbon atoms could not be detected.

IR (ATR): $\tilde{\nu}$ = 694 (w), 721 (s), 771 (vs), 821 (w), 878 (vs), 961 (w), 1002 (m), 1020 (w), 1037 (w), 1088 (m), 1157 (w), 1167 (w), 1210 (m), 1268 (m), 1367 (s), 1451 (m), 1531 (s), 1584 (s), 1624 (m), 1633 (m), 3155 (w), 3238 (w) cm^{-1}.

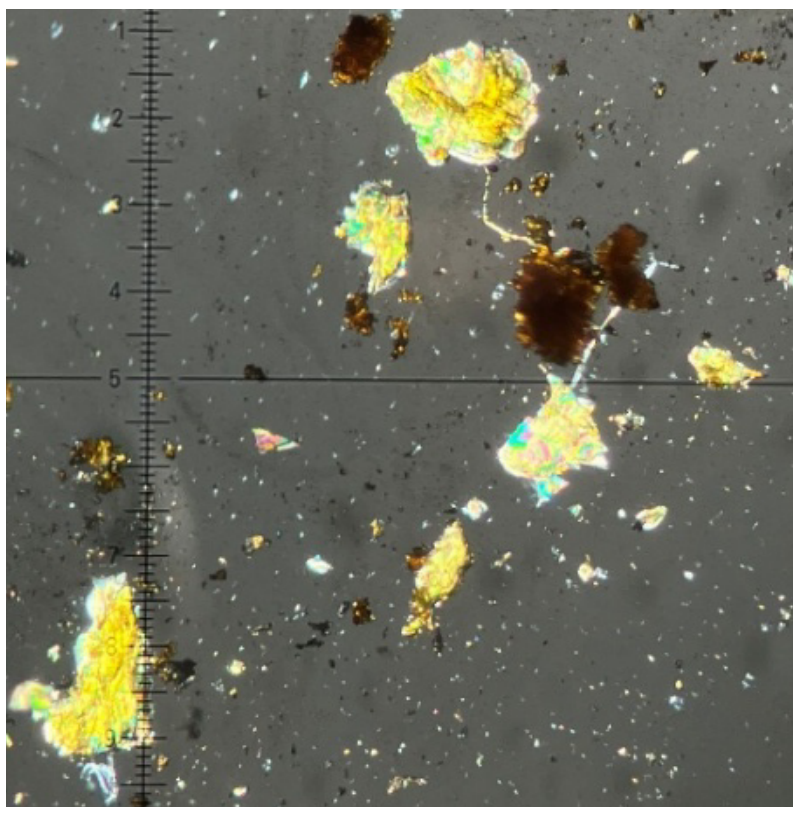

Figure S6. Crystals of [$PuCl_4$(2-pyridone)$_2$]·2THF.

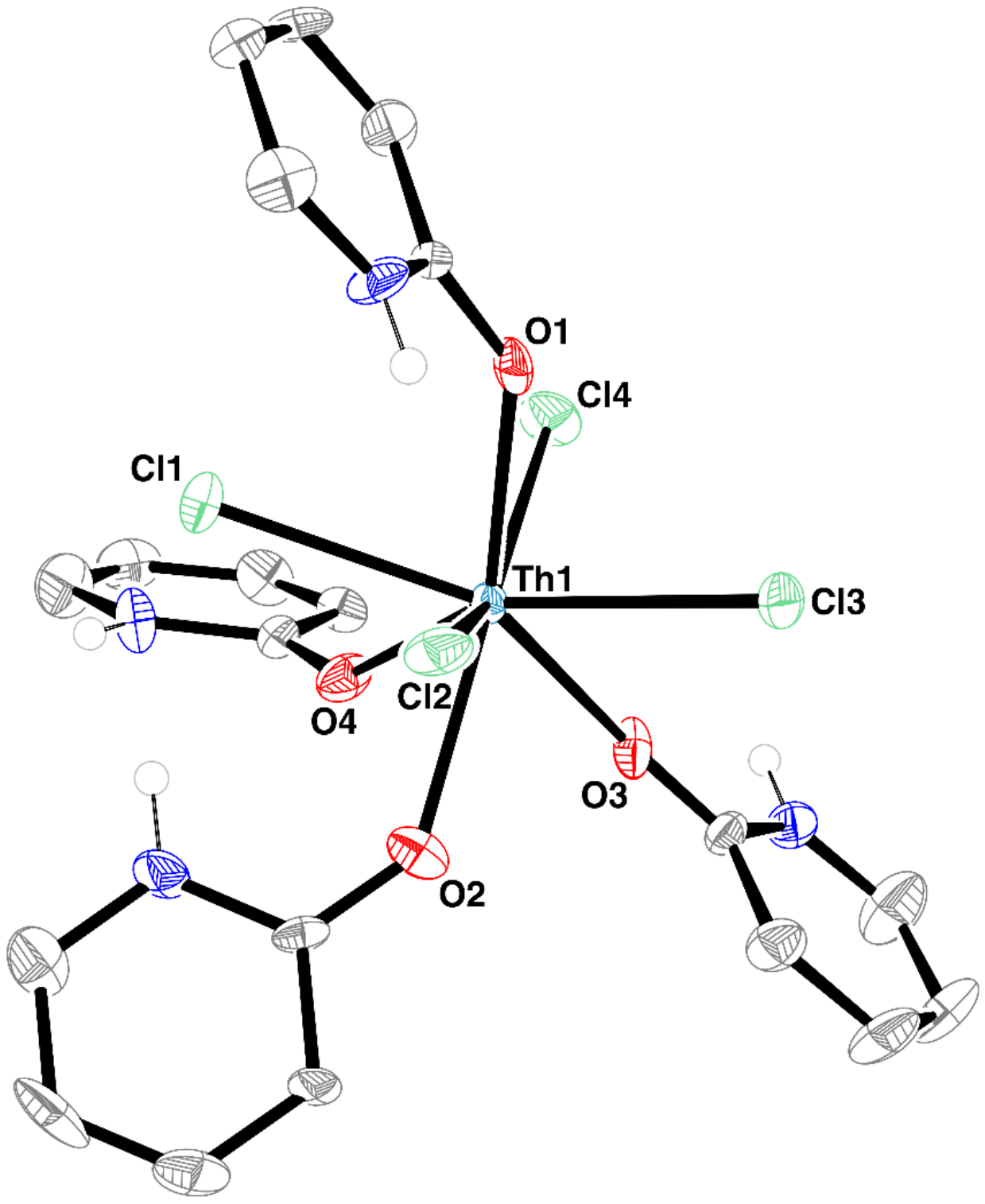

Figure S7. Molecular structure of [$ThCl_4(HPyO)_4$], isolated from the reaction of [$ThCl_4(DME)_2$] with 2 equiv. HPyO. Ellipsoids are shown at 50% probability level.

IR spectra

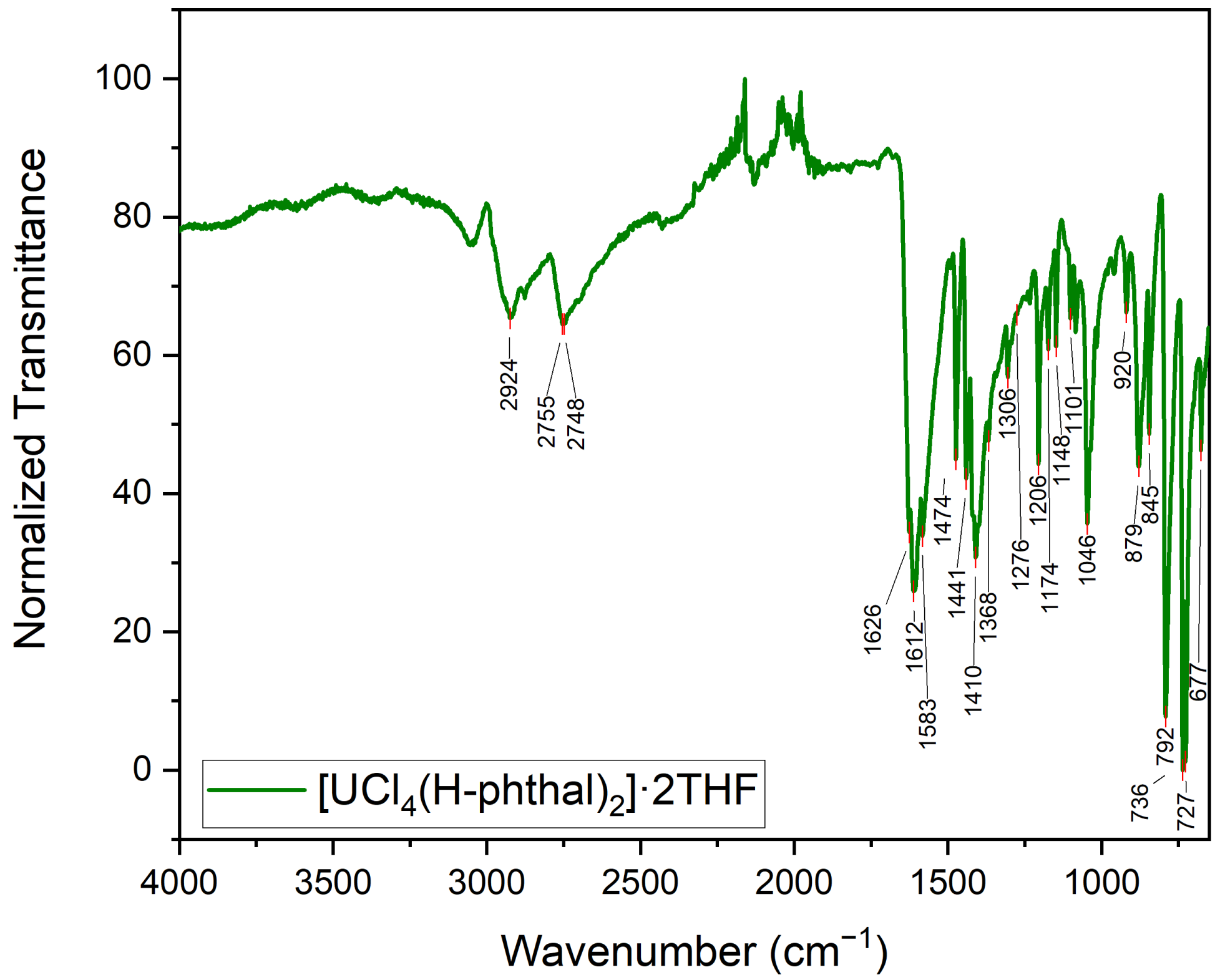

Figure S8. IR-ATR spectrum of [UCl_4(H-phthal)$_2$]·2THF.

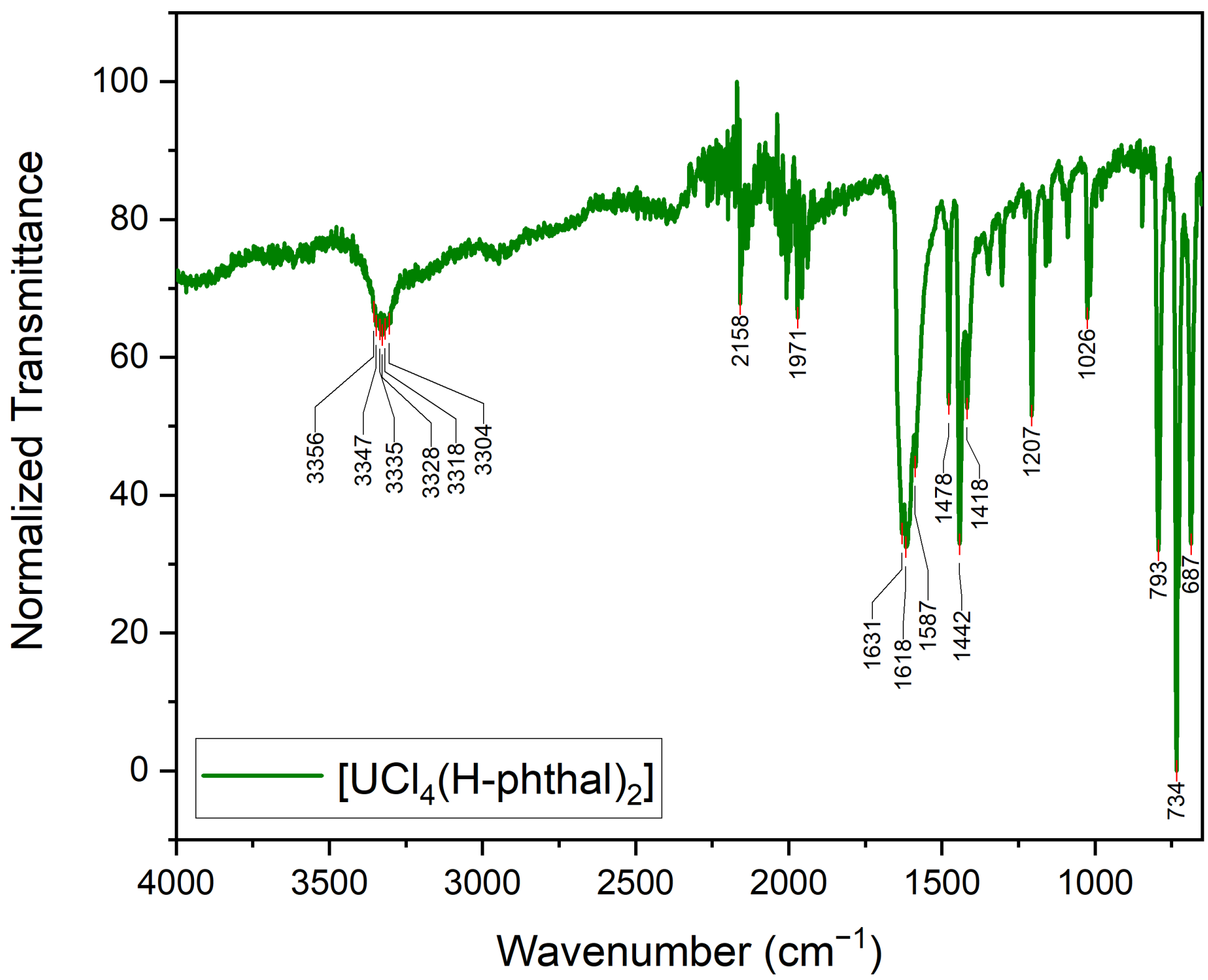

Figure S9. IR-ATR spectrum of [UCl_4(H-phthal)$_2$].

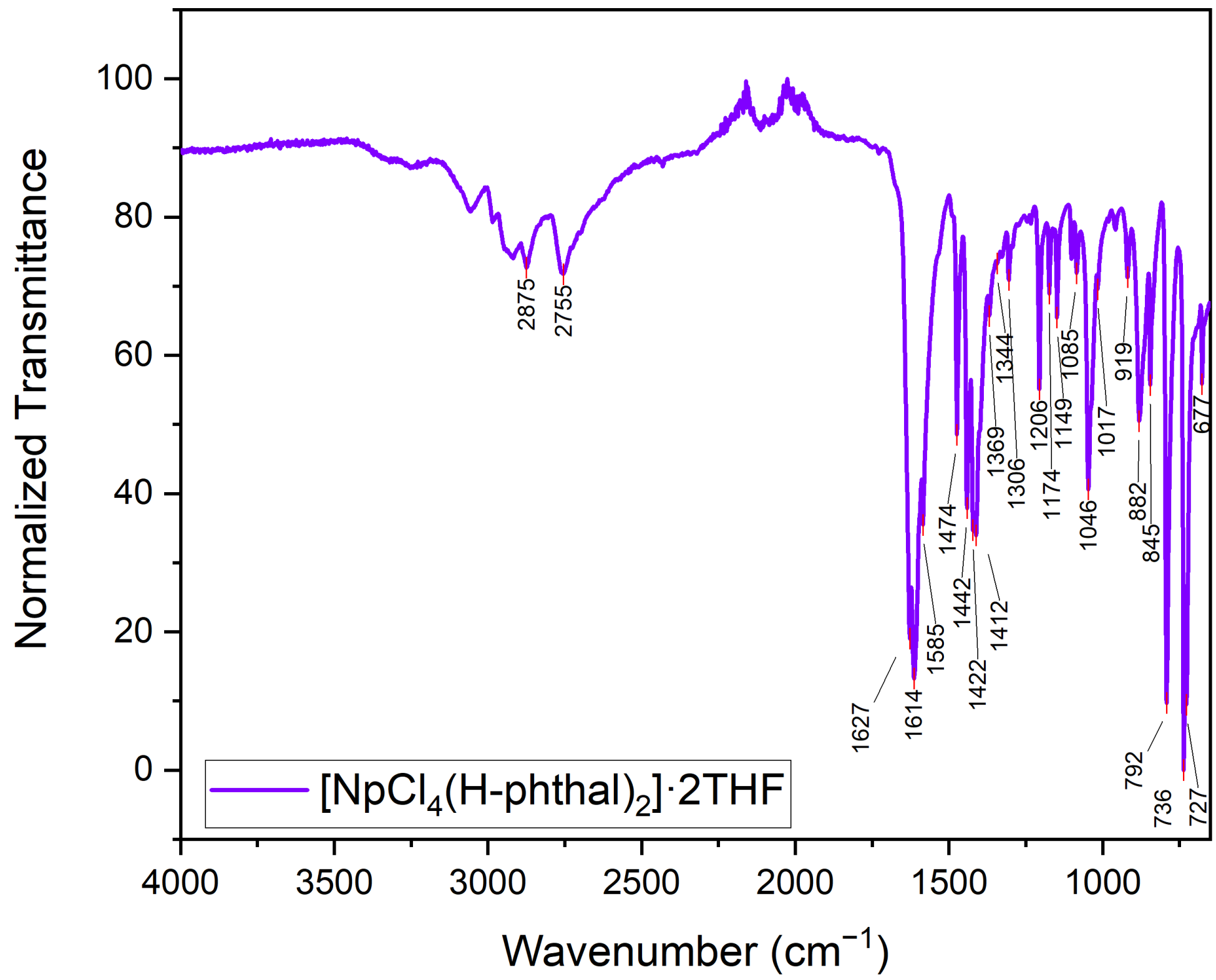

Figure S10. IR-ATR spectrum of [NpCl$_4$(H-phthal)$_2$]·2THF.

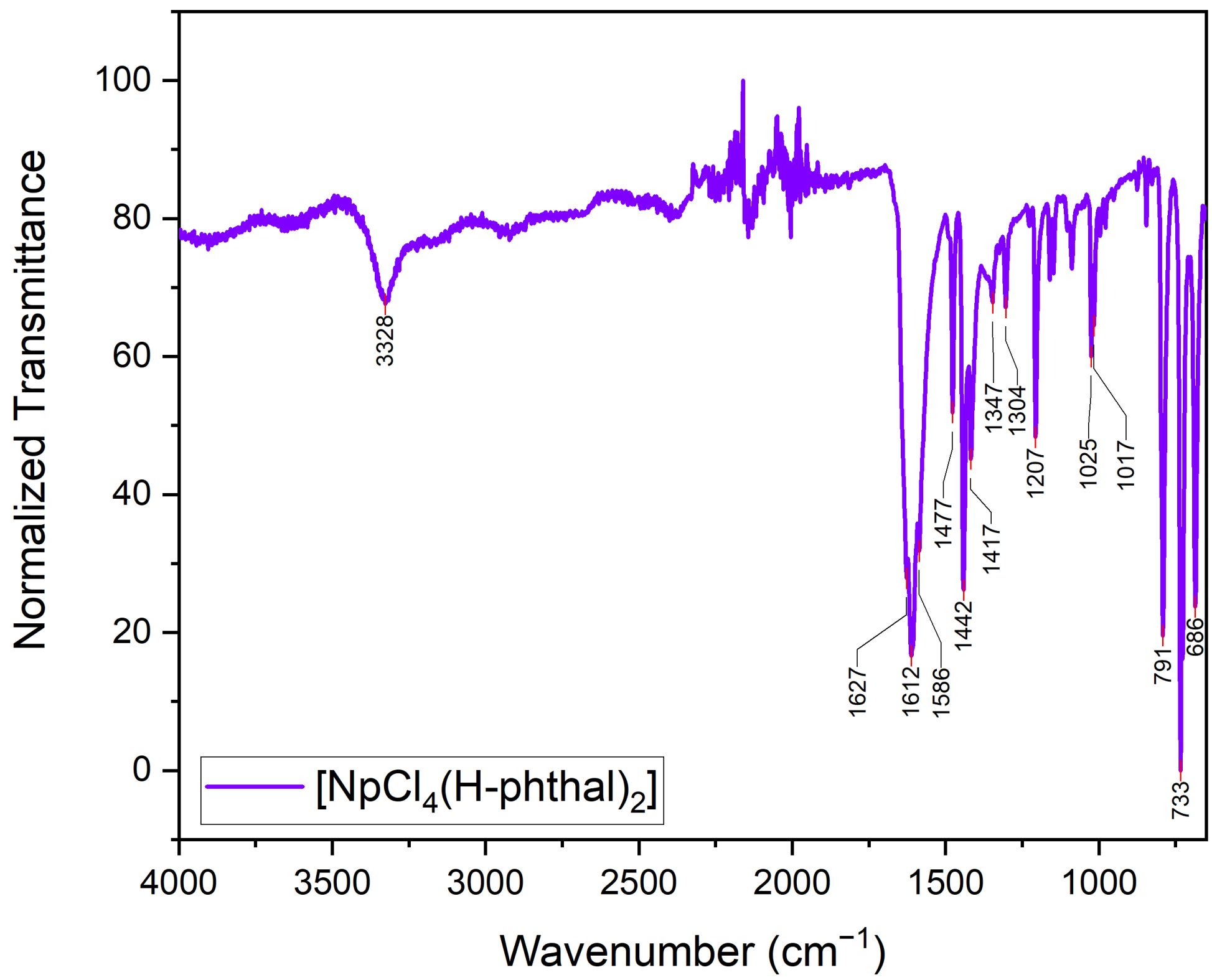

Figure S11. IR-ATR spectrum of [NpCl$_4$(H-phthal)$_2$].

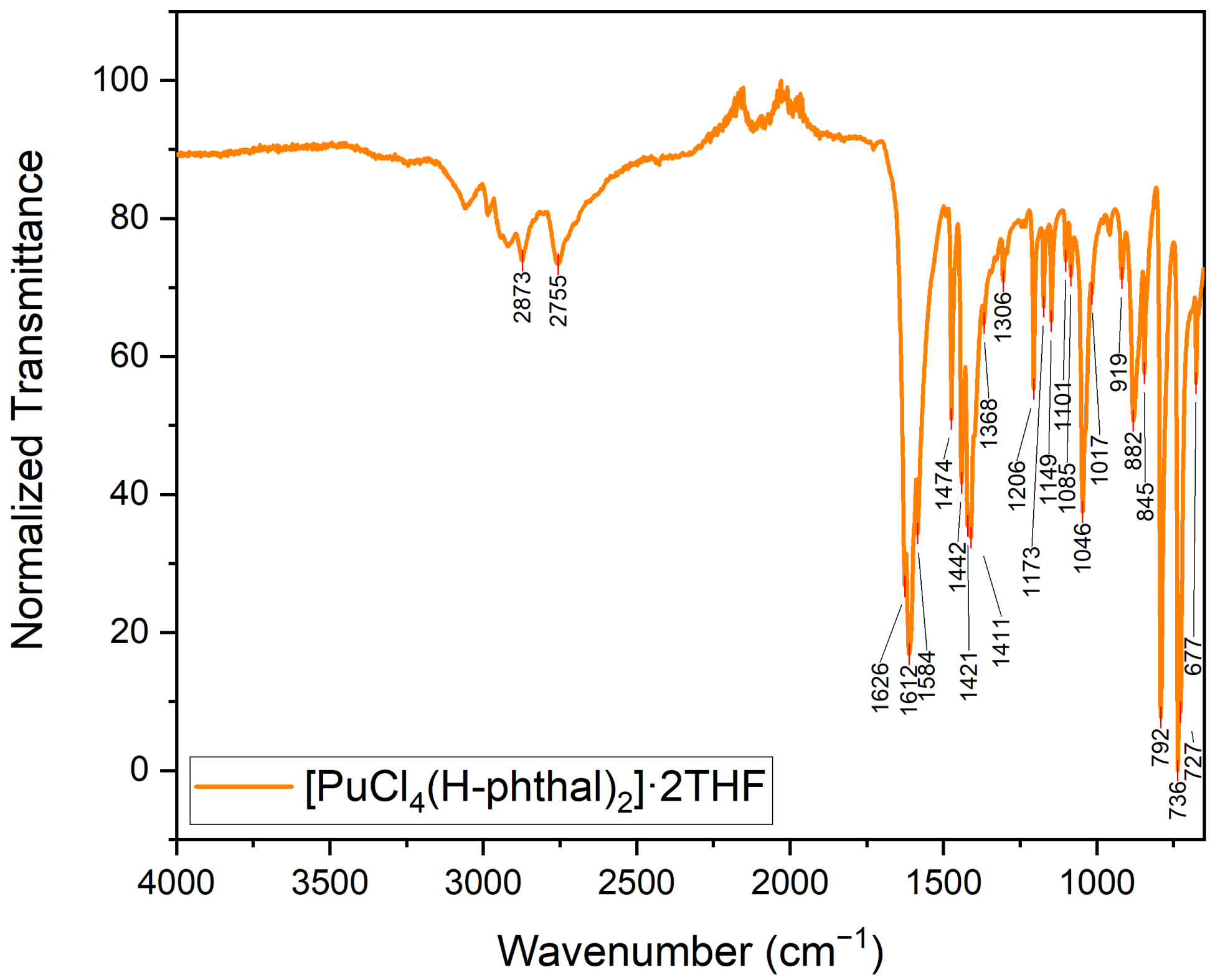

Figure S12. IR-ATR spectrum of [$PuCl_4$(H-phthal)$_2$]·2THF.

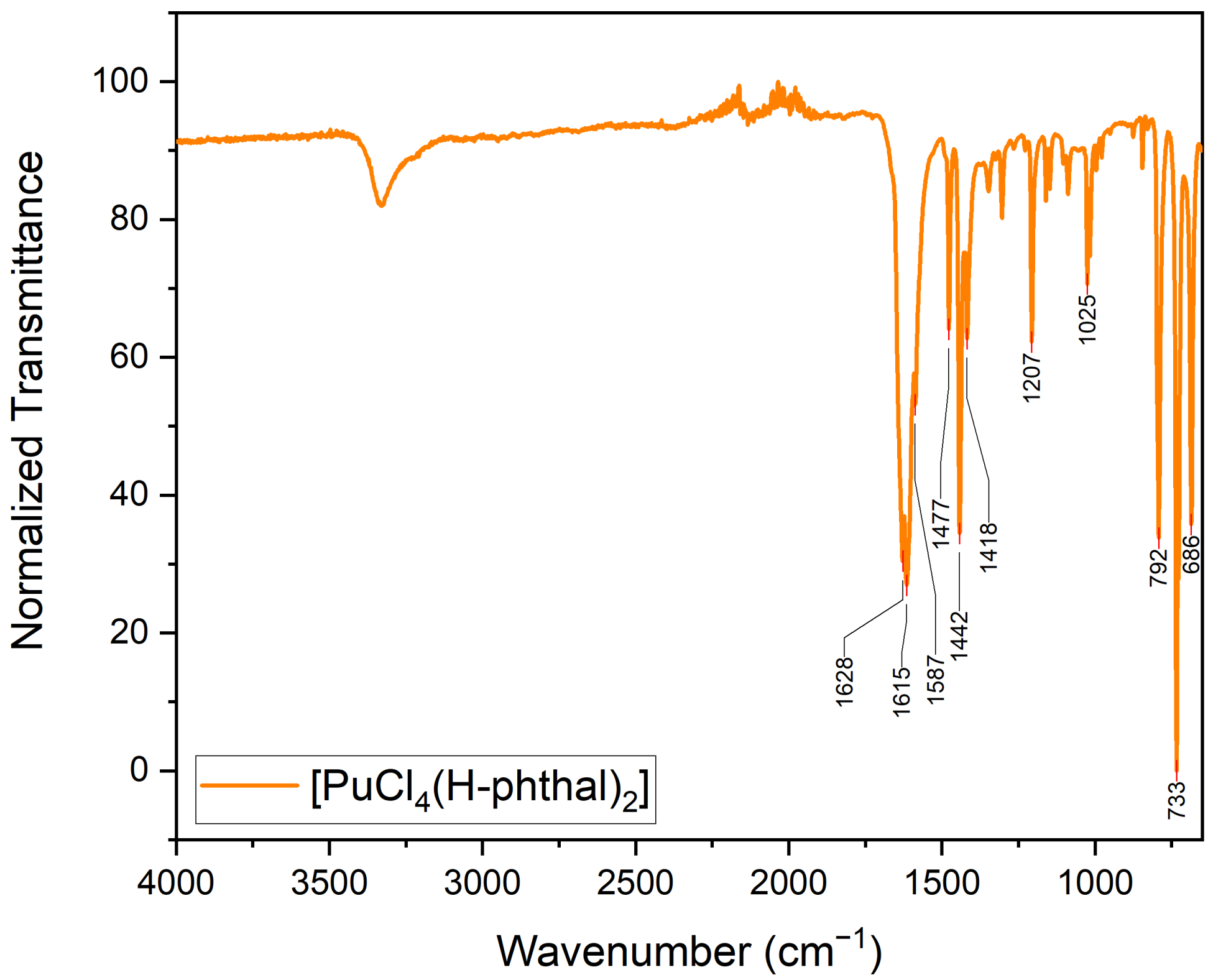

Figure S13. IR-ATR spectrum of [$PuCl_4$(H-phthal)$_2$].

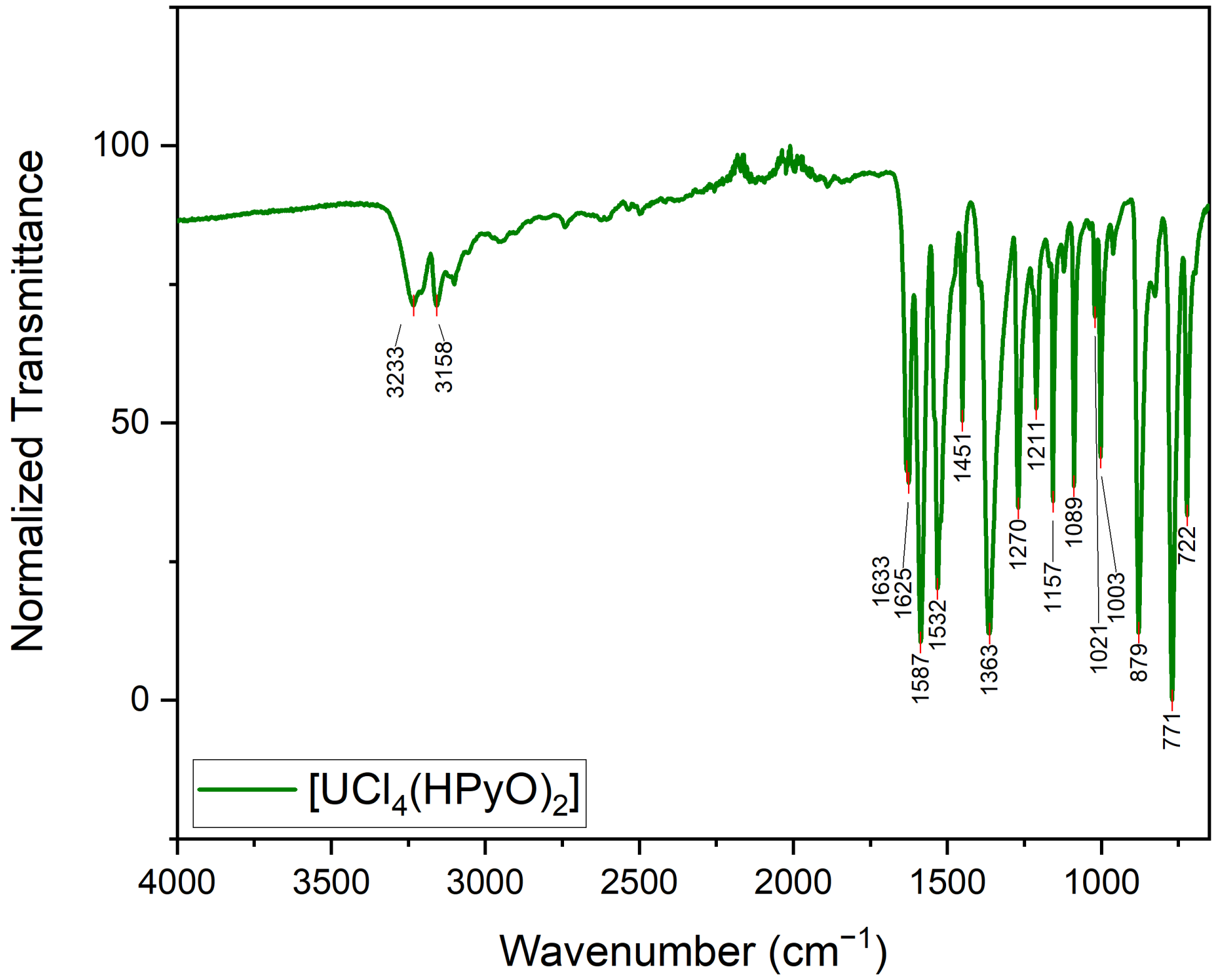

Figure S14. IR-ATR spectrum of $[UCl_4(HPyO)_2]$.

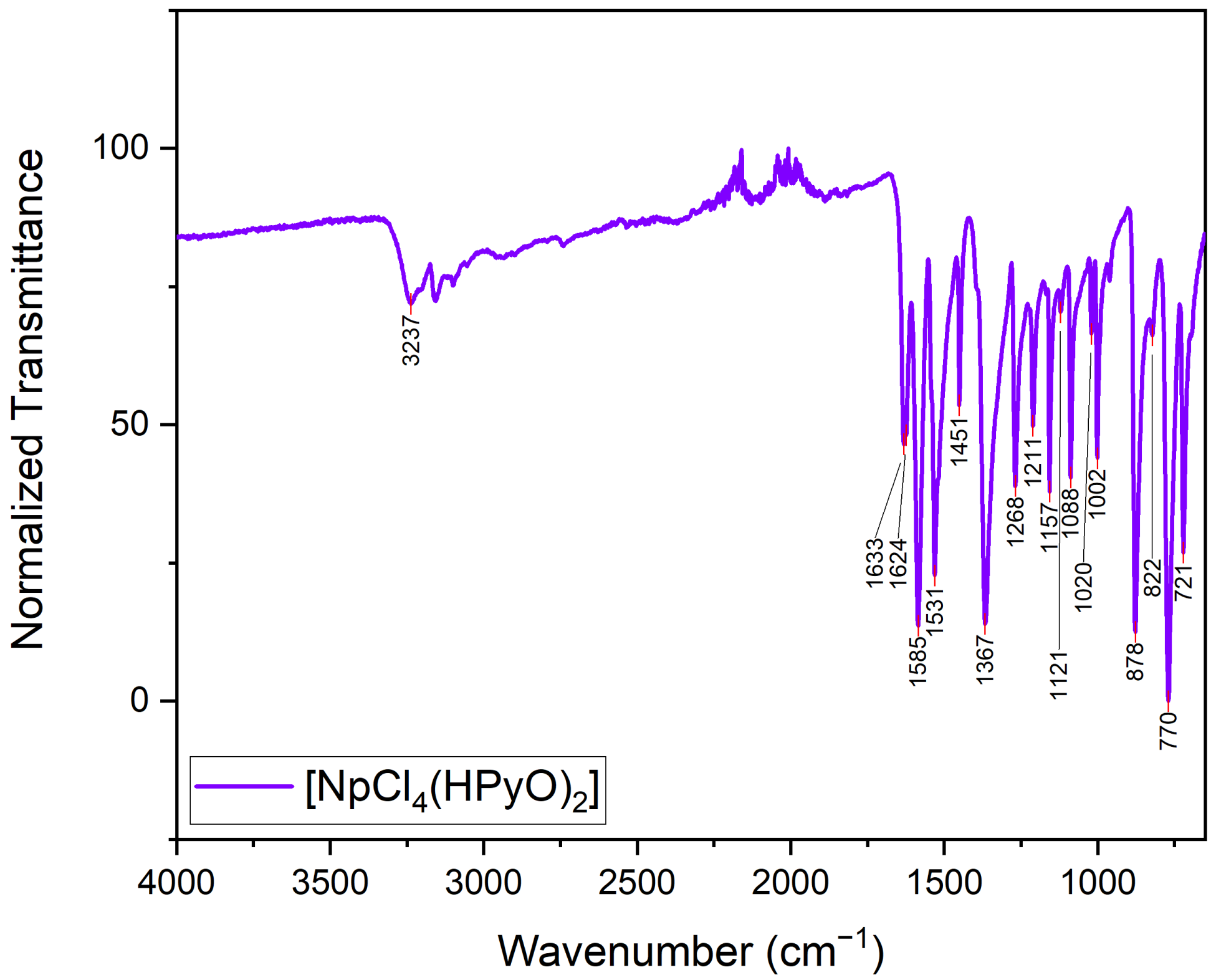

Figure S15. IR-ATR spectrum of $[NpCl_4(HPyO)_2]$.

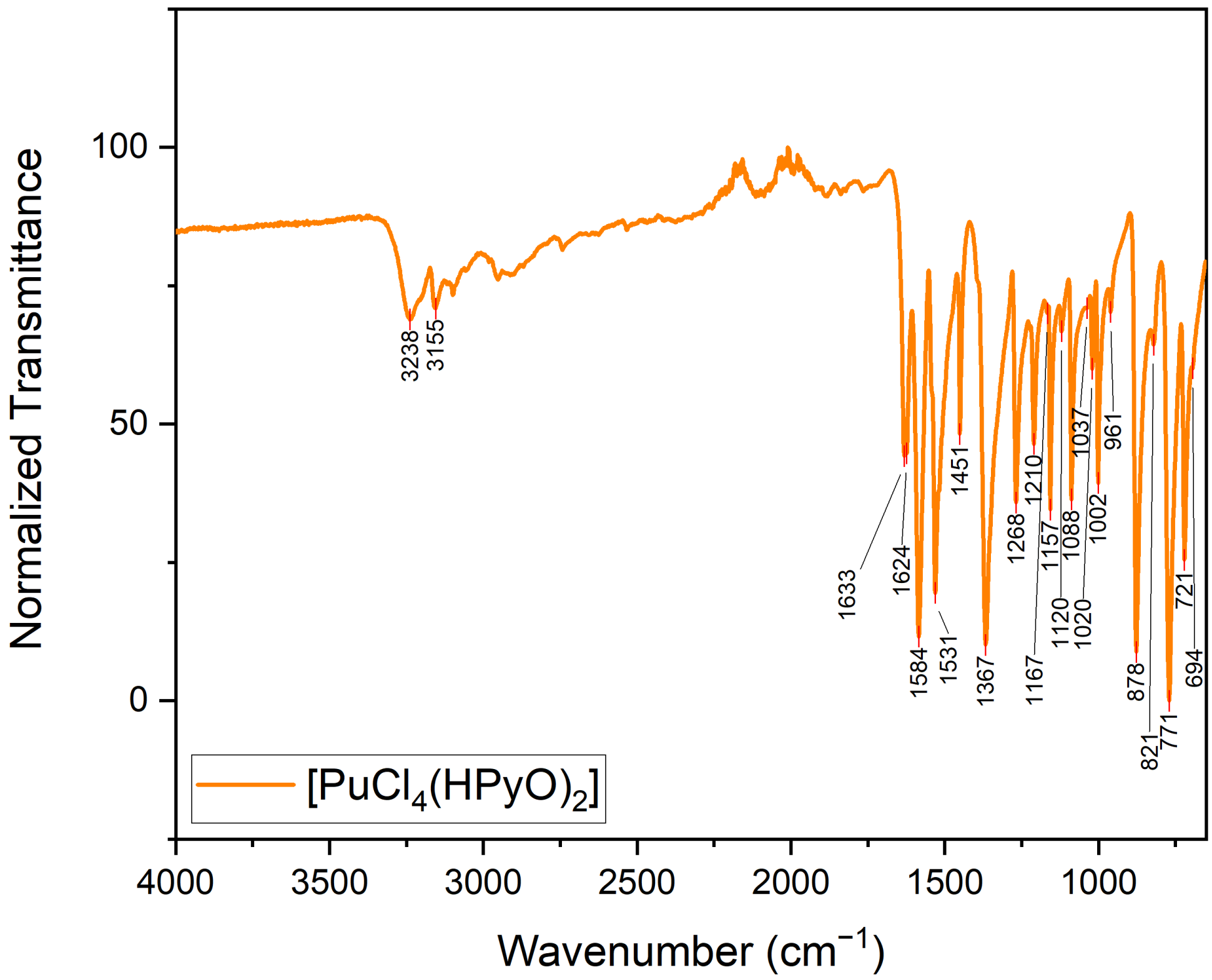

Figure S16. IR-ATR spectrum of [$PuCl_4(HPyO)_2$].

SC-XRD Structures

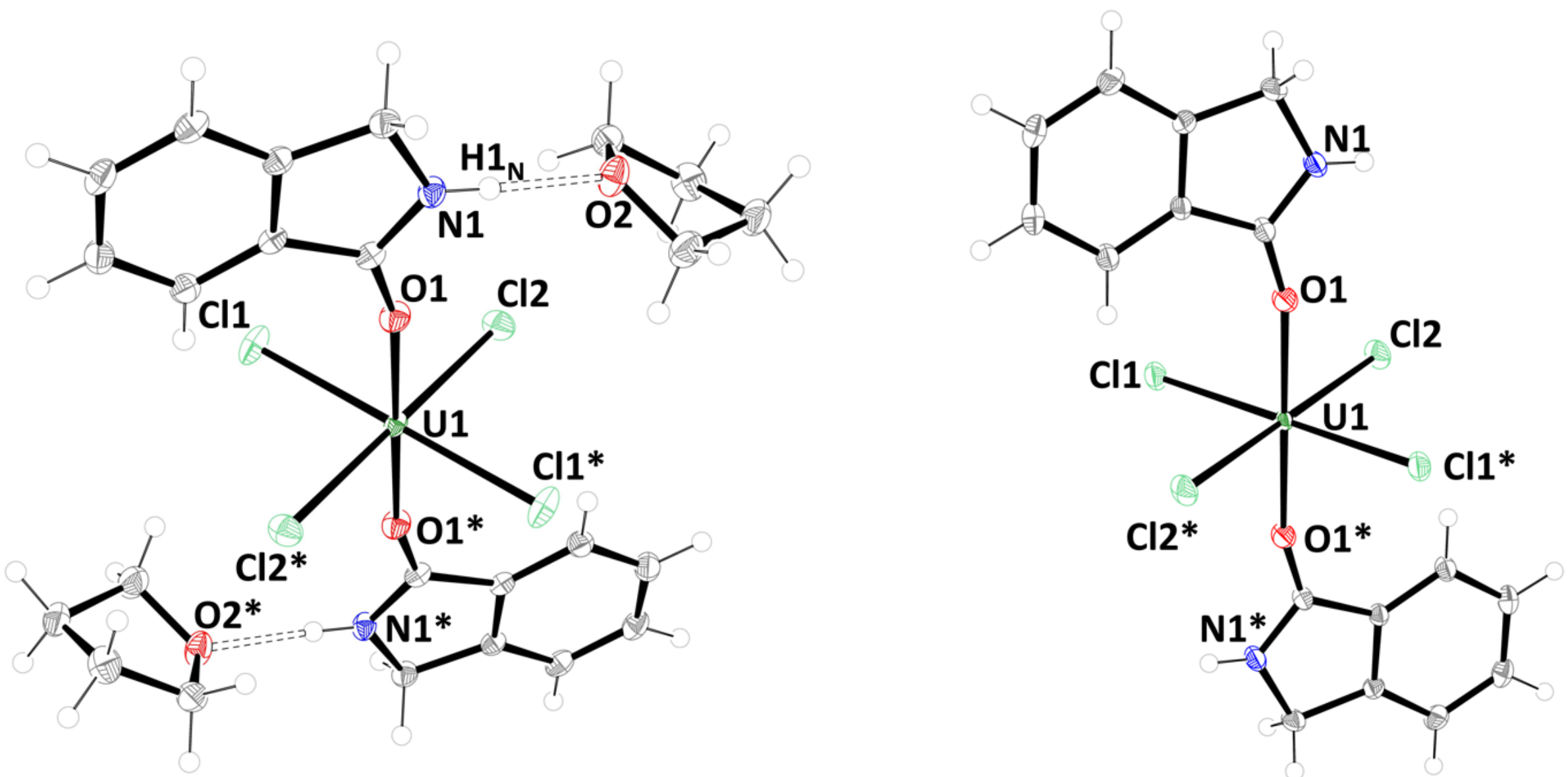

Figure S17. Molecular structures of [UCl_4(H-phthal)$_2$]·2THF (left) and [UCl_4(H-phthal)$_2$] (right). Ellipsoids are shown at 50% probability level. Symmetry elements * are $-x$, $-y+1$, $-z+1$ and $-x+2$, $-y+1$, $-z+2$, respectively.

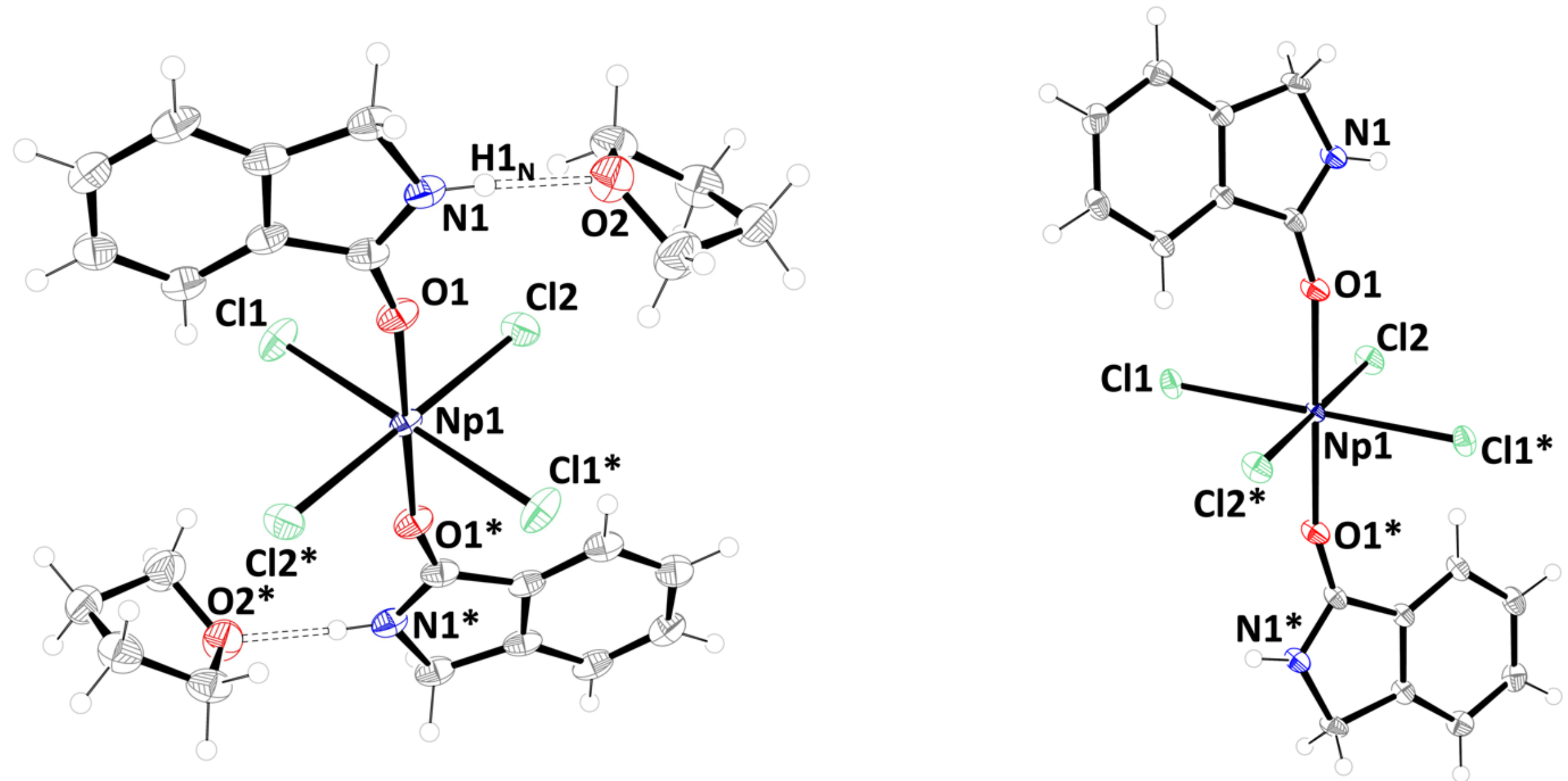

Figure S18. Molecular structures of [$NpCl_4$(H-phthal)$_2$]·2THF (left) and [$NpCl_4$(H-phthal)$_2$] (right). Ellipsoids are shown at 50% probability level. Symmetry elements * are −*x*, −*y*+1, −*z*+1 and −x+2, −y+1, −z, respectively.

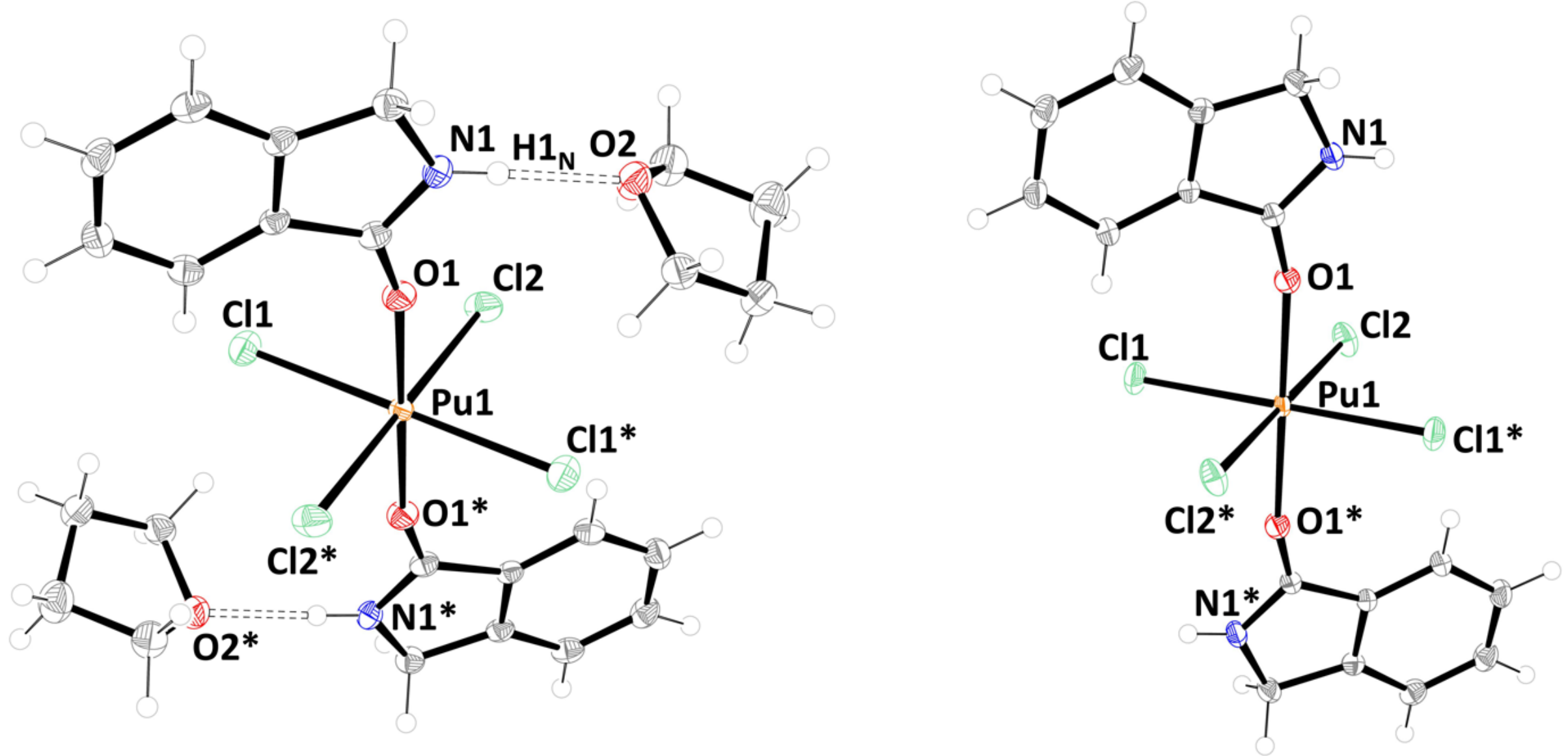

Figure S19. Molecular structures of [$PuCl_4$(H-phthal)$_2$]·2THF (left) and [$PuCl_4$(H-phthal)$_2$] (right). Ellipsoids are shown at 50% probability level. Symmetry elements * are −*x*, −*y*+1, −*z*+1 and −*x*+2, −*y*+1, −*z*+2, respectively.

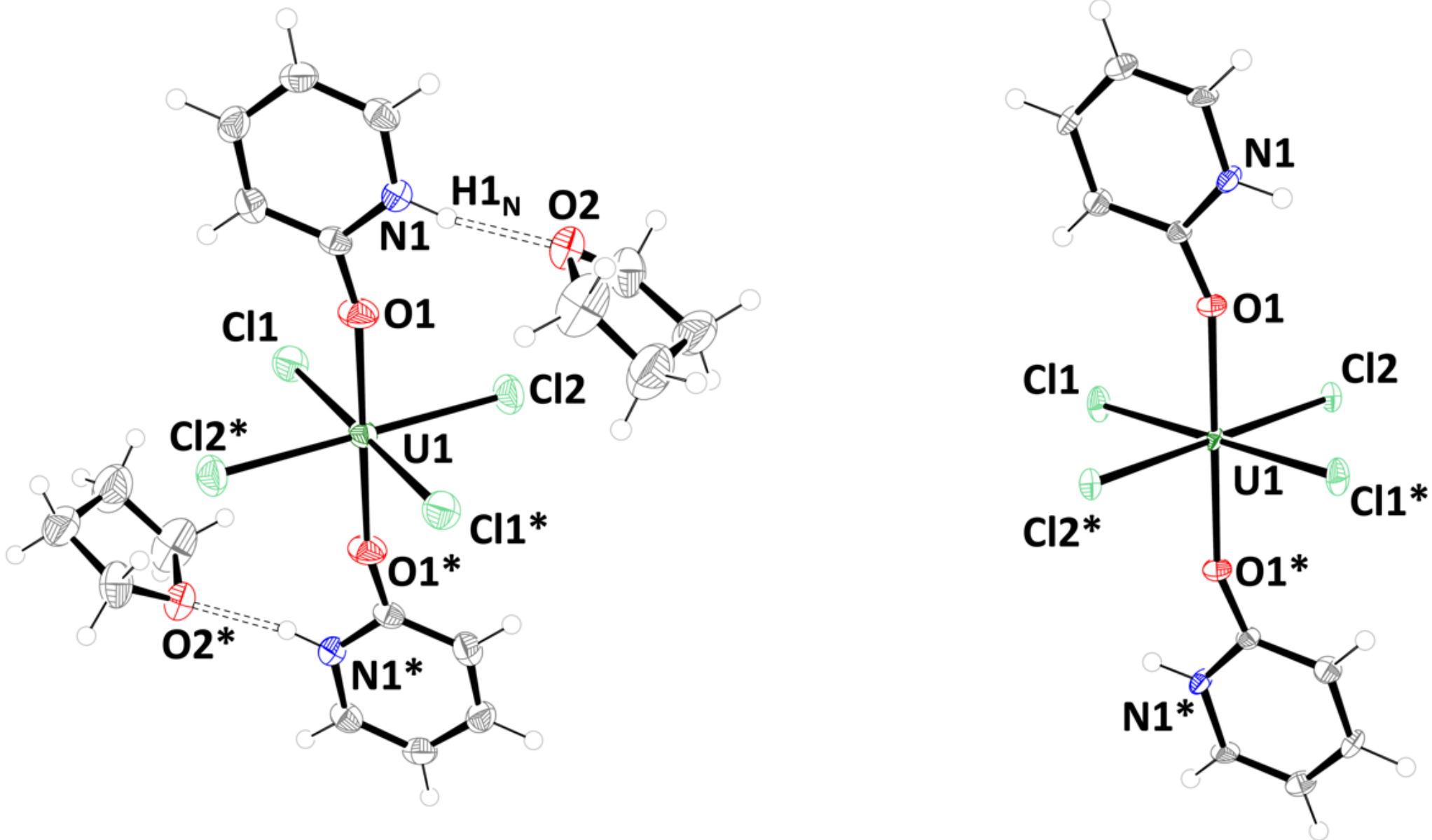

Figure S20. Molecular structures of $[UCl_4(HPyO)_2]\cdot 2THF$ (left) and $[UCl_4(HPyO)_2]$ (right). Ellipsoids are shown at 50% probability level. Symmetry elements * are −*x*, −*y*+1, −*z*+1 and −x, −y+1, −z+2, respectively.

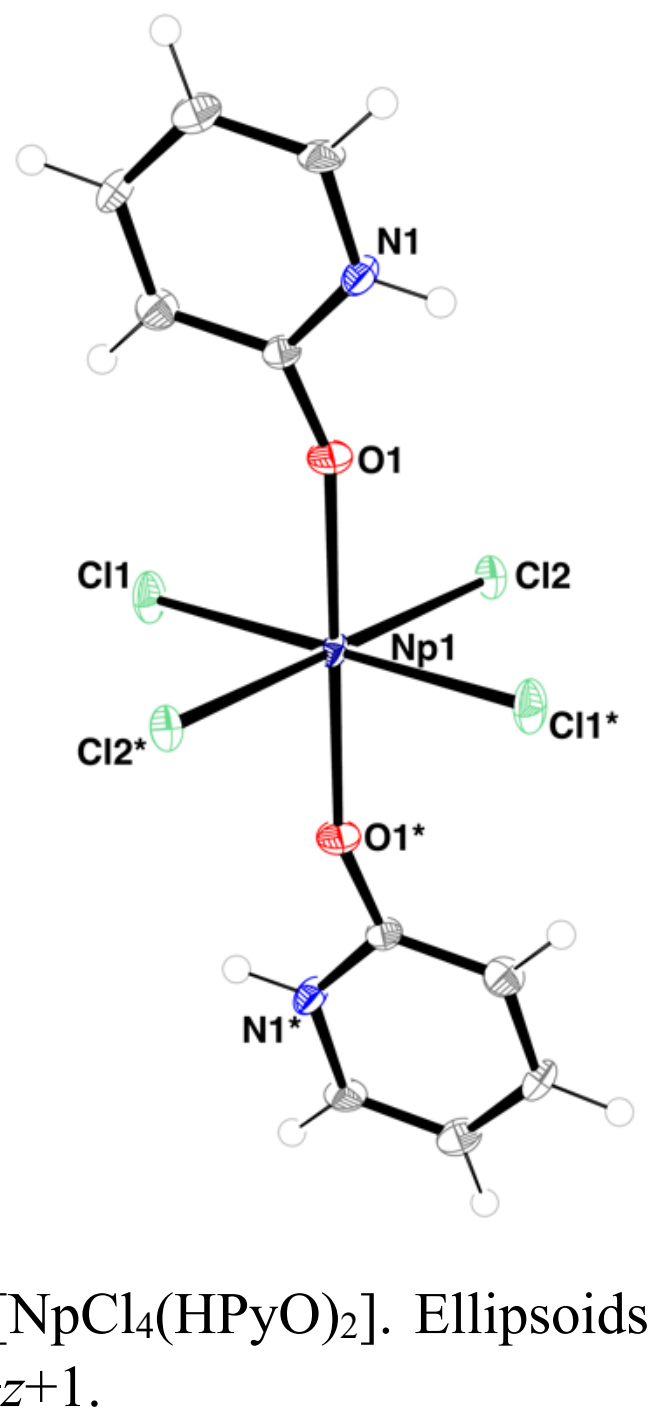

Figure S21. Molecular structure of $[NpCl_4(HPyO)_2]$. Ellipsoids are shown at 50% probability level. Symmetry elements * are −*x*, −*y*+1, −*z*+1.

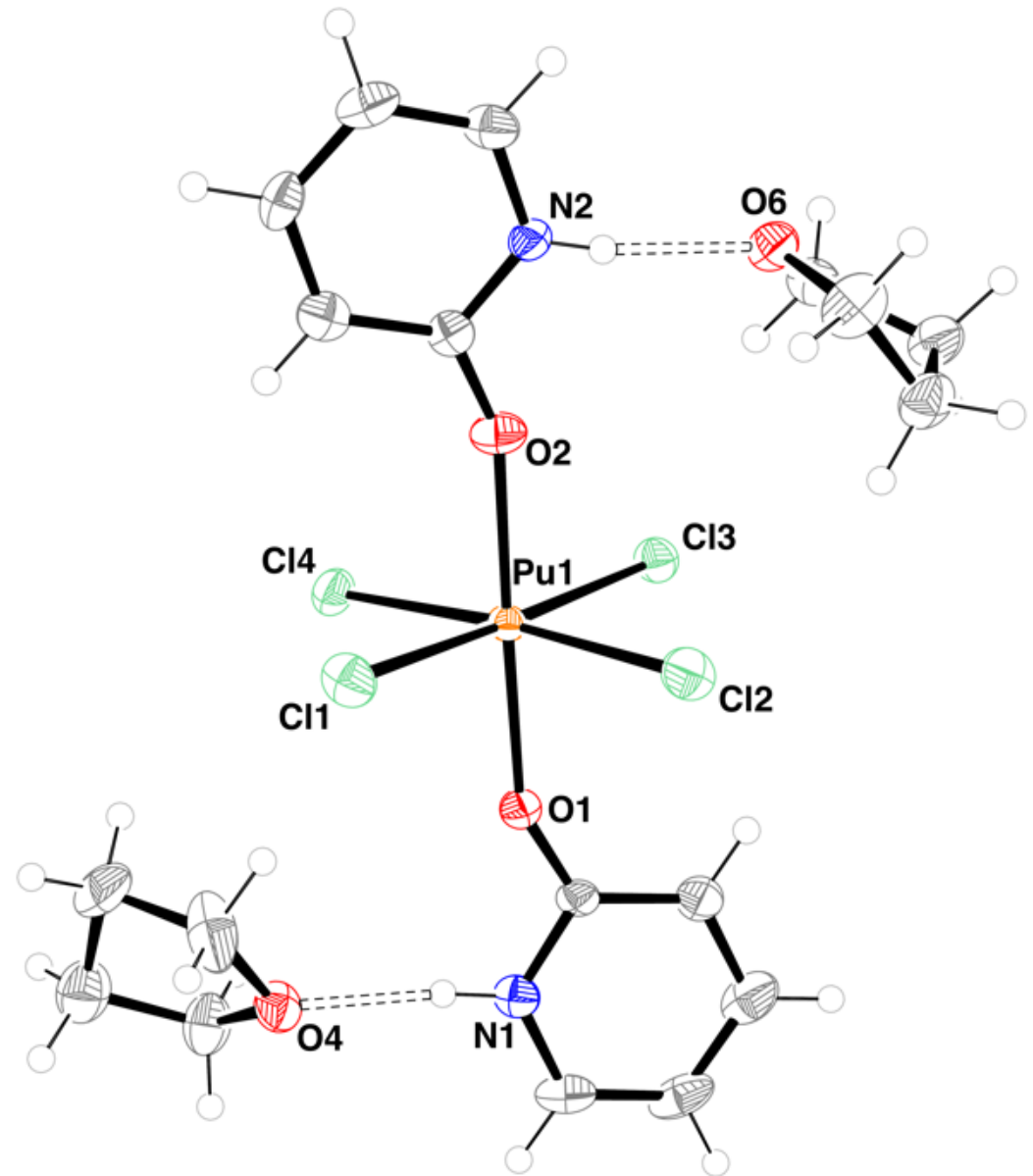

Figure S22. Molecular structure of [$PuCl_4(HPyO)_2$]·2THF. Ellipsoids are shown at 50% probability level. The asymmetric unit contains 1.5 complex molecules and three THF molecules. Only one molecule is depicted for clarity.

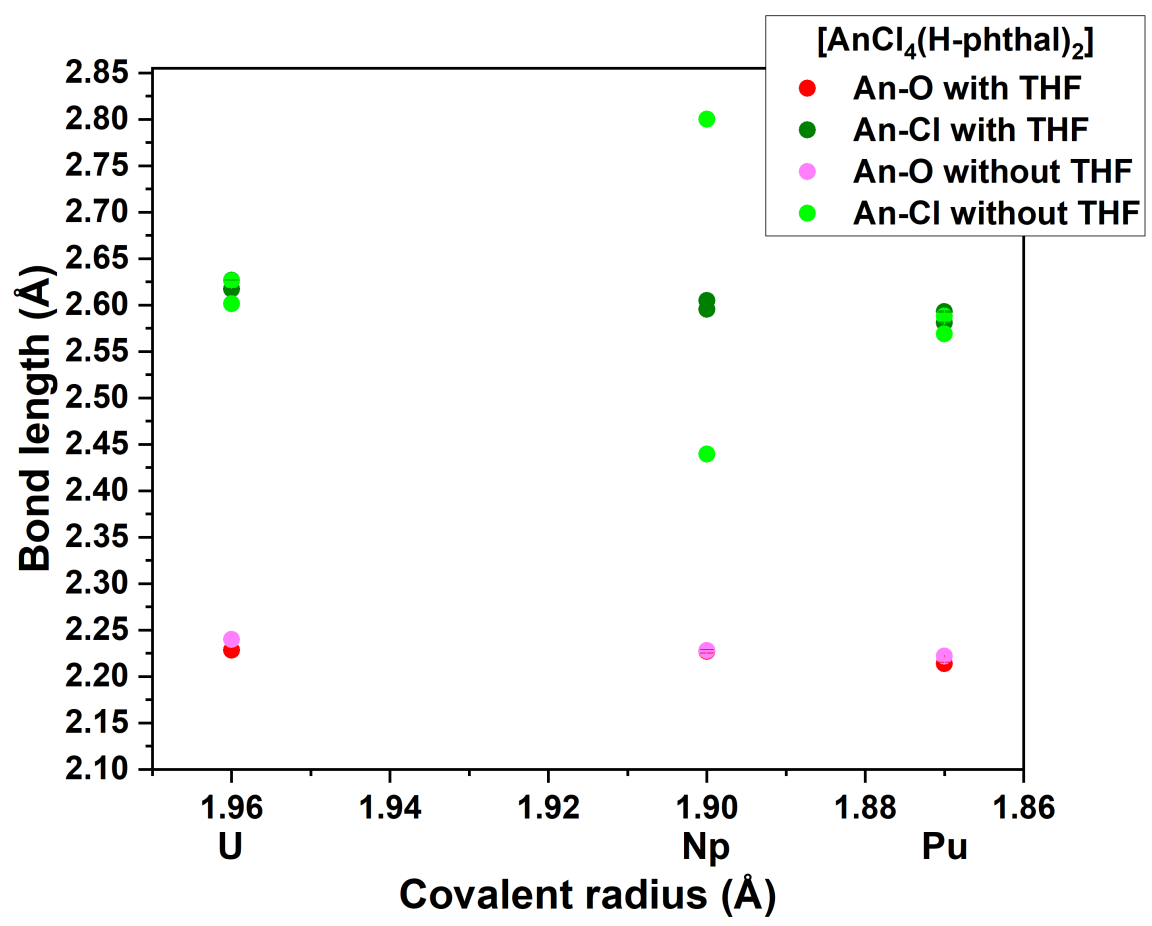

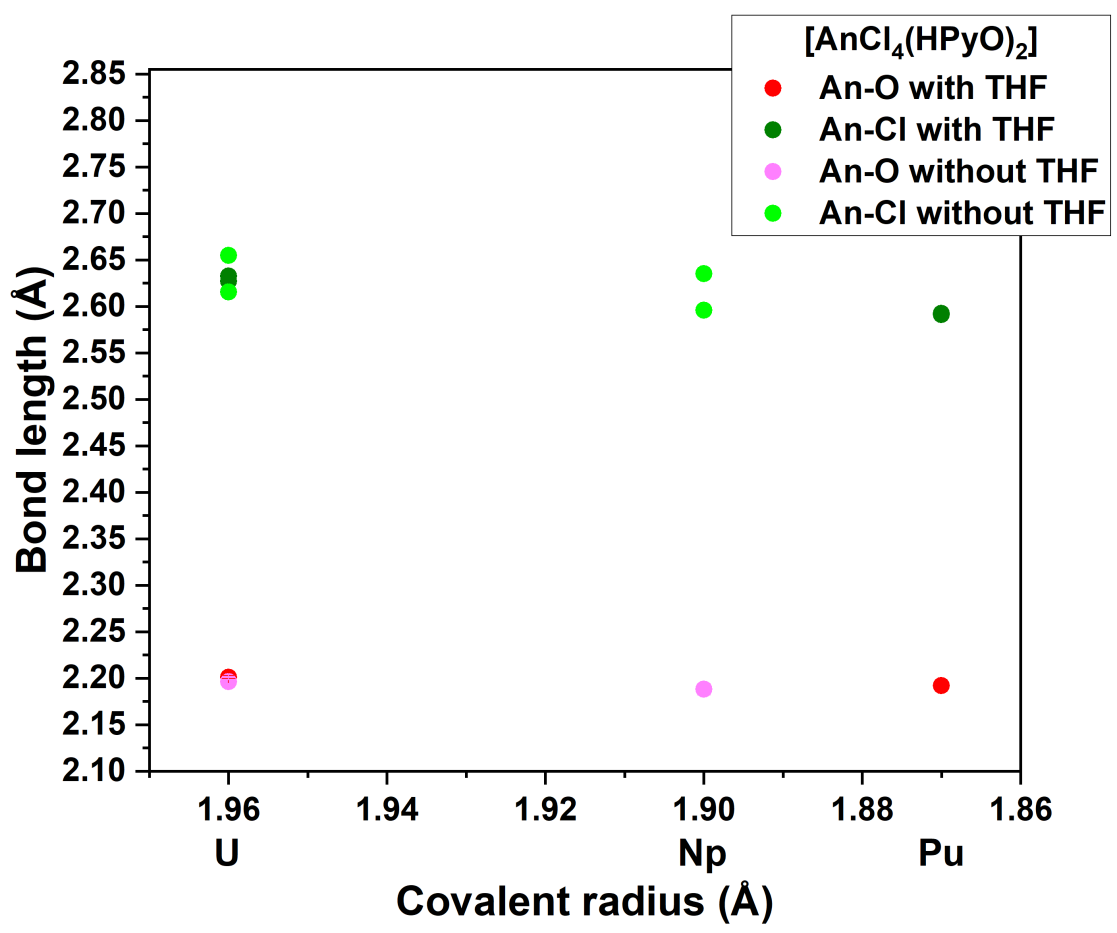

Figure S23. An–O and An–Cl bond lengths of [$AnCl_4$(H-phthal)$_2$] (*top*) and [$AnCl_4(HPyO)_2$] (*bottom*) plotted against the covalent radii[12] of the actinides (An = U, Np, Pu).

Table S1. SC-XRD crystal parameters of $[AnCl_4(Hphthal)_2]\cdot 2THF$ complexes.

	**[UCl$_4$(H-phthal)$_2$]·2(THF)**	**[NpCl$_4$(H-phthal)$_2$]·2(THF)**	**[PuCl$_4$(H-phthal)$_2$]·2(THF)**
**Crystal data**			
**CCDC**	2550531	2550532	2550527
**Chemical formula**	$C_{16}H_{14}Cl_4N_2O_2U\cdot 2(C_4H_8O)$	$C_{16}H_{14}Cl_4N_2NpO_2\cdot 2(C_4H_8O)$	$C_{16}H_{14}Cl_4N_2O_2Pu\cdot 2(C_4H_8O)$
M_r	790.33	789.30	794.30
**Crystal system, space group**	Triclinic, *P*1	Triclinic, *P*1	Triclinic, *P*1
**Temperature (K)**	100	100	100
***a*, *b*, *c* (Å)**	8.9540 (2), 9.6448 (2), 9.7527 (2)	8.9351 (5), 9.6096 (5), 9.6892 (5)	8.9398 (3), 9.6450 (3), 9.6889 (3)
***α*, *β*, *γ* (°)**	109.2222 (8), 91.9060 (8), 115.7717 (8)	109.4133 (15), 91.9212 (16), 115.5148 (16)	109.0549 (11), 91.9809 (12), 115.8526 (11)
V (Å^3)	700.60 (3)	692.69 (6)	695.07 (4)
Z	1	1	1
**Radiation type**	Mo *K*α	Mo *K*α	Mo *K*α
**μ (mm^{-1})**	6.21	4.17	2.79
**Crystal size (mm)**	0.10 × 0.07 × 0.05	0.13 × 0.06 × 0.02	0.10 × 0.06 × 0.02
**Data collection**			
**Diffractometer**	Bruker D8 Venture	Bruker D8 Venture	Bruker D8 Venture
**Absorption correction**	Multi-scan *SADABS*	Multi-scan *SADABS*	Multi-scan *SADABS*
T_{min}, T_{max}	0.620, 0.747	0.641, 0.746	0.658, 0.746
**No. of measured, independent and observed [$I > 2\sigma(I)$] reflections**	70683, 4087, 4084	48707, 3184, 3142	51724, 3359, 3341
R_{int}	0.044	0.065	0.060
**(sin θ/λ)$_{max}$ (Å^{-1})**	0.703	0.650	0.661
**Refinement**			
$R[F^2 > 2\sigma(F^2)]$, $wR(F^2)$, S	0.014, 0.030, 1.06	0.027, 0.055, 1.08	0.021, 0.038, 1.08
**No. of reflections**	4087	3184	3359
**No. of parameters**	173	173	163
**No. of restraints**	7	3	1
**H-atom treatment**	H atoms treated by a mixture of independent and constrained refinement	H atoms treated by a mixture of independent and constrained refinement	H atoms treated by a mixture of independent and constrained refinement
$\Delta\rho_{max}$, $\Delta\rho_{min}$ (e Å^{-3})	0.49, −0.43	1.38, −0.92	0.86, −0.64

Table S2. SC-XRD crystal parameters of [$AnCl_4$(H-phthal)$_2$] complexes.

	**[UCl_4(H-phthal)$_2$]**	**[$NpCl_4$(H-phthal)$_2$]**	**[$PuCl_4$(H-phthal)$_2$]**
**Crystal data**			
**CCDC**	2550524	2550529	2550533
**Chemical formula**	$C_{16}H_{14}Cl_4N_2O_2U$	$C_{16}H_{14}Cl_4N_2NpO_2$	$C_{16}H_{14}Cl_4N_2O_2Pu$
M_r	646.12	645.09	650.09
**Crystal system, space group**	Triclinic, *P*1	Triclinic, *P*1	Triclinic, *P*1
**Temperature (K)**	100	100	100
***a*, *b*, *c* (Å)**	7.4190 (4),<br>7.4319 (4),<br>9.8814 (6)	7.4048 (2),<br>7.4089 (3),<br>9.8676 (5)	7.4026 (2),<br>7.4154 (2),<br>9.8428 (3)
***α*, *β*, *γ* (°)**	105.607 (2),<br>110.909 (2),<br>92.899 (2)	105.596 (2),<br>110.808 (2),<br>92.934 (1)	105.7269 (8),<br>110.5971 (8),<br>93.0819 (9)
V (Å^3)	483.64 (5)	480.90 (3)	480.12 (2)
Z	1	1	1
**Radiation type**	Mo *K*α	Mo *K*α	Mo *K*α
**μ (mm^{-1})**	8.96	5.97	4,00
**Crystal size (mm)**	0.33 × 0.16 × 0.05	0.10 × 0.05 × 0.05	0.14 × 0.09 × 0.04
**Data collection**			
**Diffractometer**	Bruker D8 Venture	Bruker D8 Venture	Bruker D8 Venture
**Absorption correction**	Multi-scan<br>*SADABS*	Multi-scan<br>*SADABS*	Multi-scan<br>*SADABS*
T_{min}, T_{max}	0.394, 0.747	0.653, 0.746	0.643, 0.747
**No. of measured, independent and observed [$I > 2σ(I)$] reflections**	3776, 3776, 3776	2303, 2303, 2301	72593, 4875, 4875
R_{int}	0.050	0.052	0.033
**(sin θ/λ)$_{max}$ (Å^{-1})**	0.777	0.660	0.847
**Refinement**			
$R[F^2 > 2σ(F^2)]$, $wR(F^2)$, S	0.021, 0.060, 1.15	0.019, 0.058, 1.20	0.014, 0.030, 1.13
**No. of reflections**	3776	2303	4875
**No. of parameters**	119	119	118
**No. of restraints**	1	3	1
**H-atom treatment**	H atoms treated by a mixture of independent and constrained refinement	H atoms treated by a mixture of independent and constrained refinement	H atoms treated by a mixture of independent and constrained refinement
$Δρ_{max}$, $Δρ_{min}$ (e Å^{-3})	1.52, −1.83	1.72, −0.88	0.76, −1.14

Table S3. SC-XRD crystal parameters of $[AnCl_4(HPyO)_2]$ complexes.

	**$[UCl_4(HPyO)_2]\cdot(2THF)$**	**$[UCl_4(HPyO)_2]$**	**$[NpCl_4(HPyO)_2]$**	**$[PuCl_4(HPyO)_2]\cdot(2THF)$**
**Crystal data**				
**CCDC**	2550525	2550526	2550523	2550528
**Chemical formula**	$C_{10}H_{10}Cl_4N_2O_2U\cdot 2(C_4H_8O)$	$C_{10}H_{10}Cl_4N_2O_2U$	$C_{10}H_{10}Cl_4N_2NpO_2$	$C_{10}H_{10}Cl_4N_2O_2Pu\cdot 2(C_4H_8O)$
**M_r**	714.24	570.03	569.00	718.21
**Crystal system, space group**	Monoclinic, $P2_1/c$	Triclinic, $P1$	Triclinic, $P1$	Monoclinic, $P2_1/c$
**Temperature (K)**	100	100	100	100
***a*, *b*, *c* (Å)**	8.9636 (2), 16.2356 (4), 9.4803 (2)	6.8520 (2), 7.7725 (3), 8.5956 (3)	6.8180 (9), 7.798 (1), 8.5667 (10)	9.2707 (4), 16.2608 (6), 24.3989 (9)
**α, β, γ (°)**	116.8118 (10)	62.6784 (12), 72.9999 (13), 84.7344 (15)	63.202 (4), 72.451 (4), 84.060 (4)	97.1291 (14)
**V (Å^3)**	1231.34 (5)	388.45 (2)	387.34 (9)	3649.7 (2)
Z	2	1	1	6
**Radiation type**	Mo *K*α	Mo *K*α	Mo *K*α	Mo *K*α
**μ (mm^{-1})**	7.05	11.13	7.39	3.17
**Crystal size (mm)**	0.20 × 0.10 × 0.03	0.20 × 0.14 × 0.11	0.11 × 0.07 × 0.06	0.08 × 0.05 × 0.05
**Data collection**				
**Diffractometer**	Bruker D8 Venture	Bruker D8 Venture	Bruker D8 Venture	Bruker D8 Venture
**Absorption correction**	Multi-scan *SADABS*	Multi-scan *SADABS*	Multi-scan *SADABS*	Multi-scan *SADABS*
**T_{min}, T_{max}**	0.603, 0.746	0.475, 0.747	0.581, 0.746	0.568, 0.745
**No. of measured, independent and observed [$I > 2\sigma(I)$] reflections**	58682, 2969, 2346	3400, 3400, 3399	1672, 1672, 1658	141653, 6794, 5862
**R_{int}**	0.048	0.049	0.069	0.089
**$(\sin \theta/\lambda)_{max}$ (Å^{-1})**	0.661	0.807	0.639	0.606
**Refinement**				
**$R[F^2 > 2\sigma(F^2)]$, $wR(F^2)$, S**	0.018, 0.035, 1.05	0.018, 0.047, 1.07	0.033, 0.099, 1.11	0.036, 0.062, 1.10
**No. of reflections**	2969	3400	1672	6794
**No. of parameters**	137	89	89	403
**No. of restraints**	1			1
**H-atom treatment**	H atoms treated by a mixture of independent and constrained refinement	H-atom parameters constrained	H-atom parameters constrained	H atoms treated by a mixture of independent and constrained refinement $w = 1/[\sigma^2(F_o^2) + (0.0077P)^2 + 21.8051P]$ where $P = (F_o^2 + 2F_c^2)/3$
**$\Delta\rho_{max}$, $\Delta\rho_{min}$ (e Å^{-3})**	0.50, −0.36	0.84, −1.63	1.75, −2.69	1.33, −1.28

Table S4. SC-XRD crystal parameters of $[ThCl_4(HPyO)_4]$.

	$[ThCl_4(HPyO)_4]$
**Crystal data**	
**CCDC**	2550530
**Chemical formula**	$C_{20}H_{20}Cl_4N_4O_4Th$
M_r	754.24
**Crystal system, space group**	Monoclinic, *Pn*
**Temperature (K)**	100
***a*, *b*, *c* (Å)**	10.5527 (10),<br>8.6647 (8),<br>13.4770 (11)
**β (°)**	90.144 (3)
V (Å^3)	1232.28 (19)
Z	2
**Radiation type**	Mo *K*α
**μ (mm^{-1})**	6.52
**Crystal size (mm)**	0.08 × 0.08 × 0.07
**Data collection**	
**Diffractometer**	Bruker D8 Venture
**Absorption correction**	Multi-scan<br>*SADABS*
T_{min}, T_{max}	0.582, 0.746
**No. of measured, independent and observed [$I > 2\sigma(I)$] reflections**	63513, 6956, 6452
R_{int}	0.075
**(sin θ/λ)$_{max}$ (Å^{-1})**	0.703
**Refinement**	
$R[F^2 > 2\sigma(F^2)]$, $wR(F^2)$, S	0.028, 0.055, 1.14
**No. of reflections**	6956
**No. of parameters**	301
**No. of restraints**	32
**H-atom treatment**	H-atom parameters constrained
$\Delta\rho_{max}$, $\Delta\rho_{min}$ (e Å^{-3})	1.14, −2.11
**Absolute structure**	Twinning involves inversion, so Flack parameter cannot be determined

Computer programs: Bruker *APEX4*, Bruker *SAINT*, *SHELXT* 2014/5 (Sheldrick, 2014), *SHELXL2019*/2 (Sheldrick, 2019), *ORTEP-32* (Farugia), Ciftab (Sheldrick).

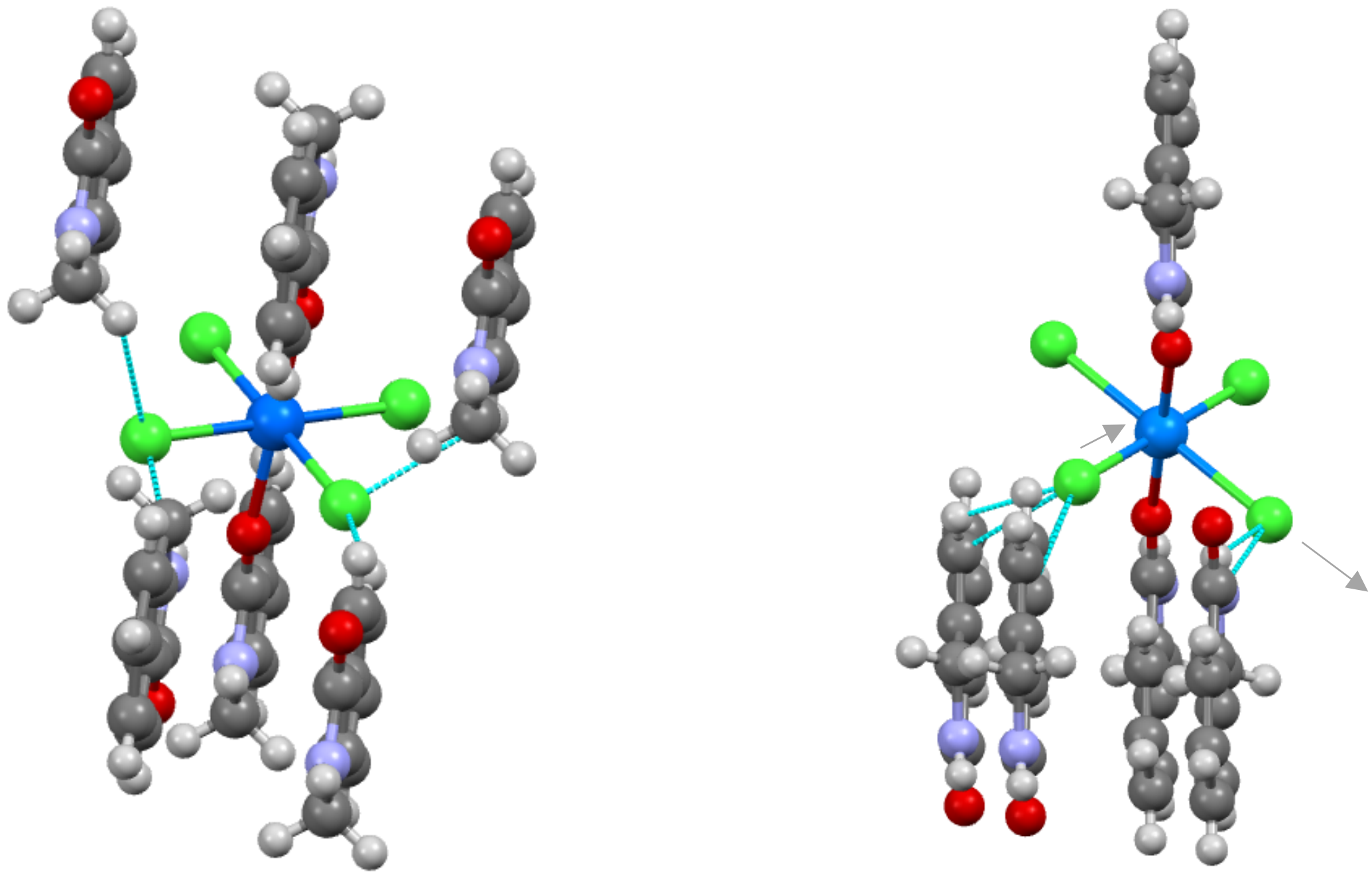

Figure S24. View of crystal structures of [$PuCl_4$(H-phthal)$_2$] (*left*) and [$NpCl_4$(H-phthal)$_2$] (*right*) highlighting different intermolecular interactions between Cl ligands and H-phthal ligands of neighboring molecules.

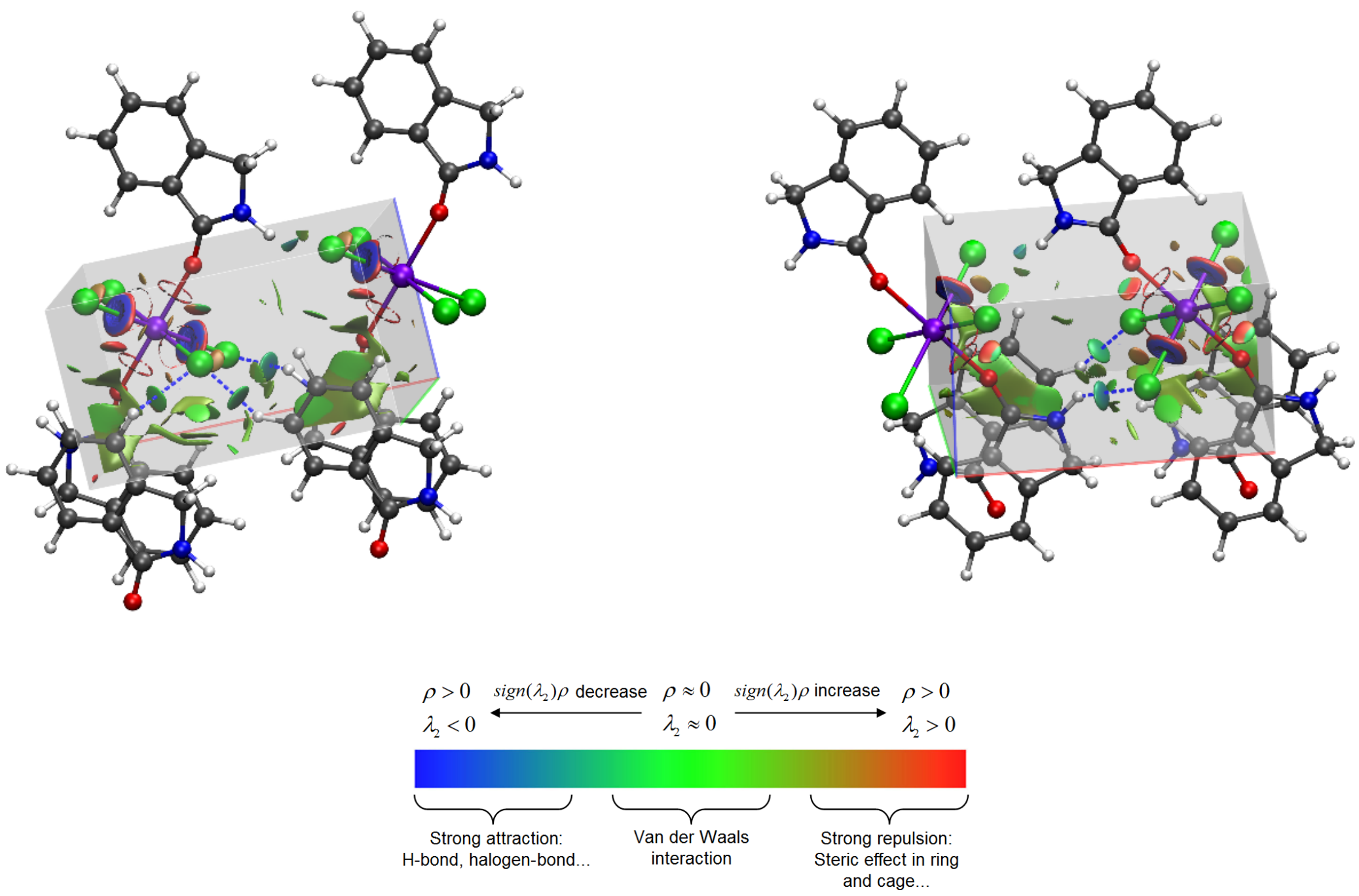

Figure S25. Non-covalent interaction (NCI) plots of a snippet of the [$NpCl_4$(H-phthal)$_2$] crystal structure containing two complex molecules. The plots were calculated with Multiwfn[13] and are based on a def-TZVPP / PBE0 calculation. The legend of the color code for the NCI isosurface was taken from the Multiwfn manual (Version 3.8).

Cartesian coordinates (in Å) of THF-free molecular structures with idealized D_{4h} symmetry in the first coordination sphere of the actinides

- optimizations performed with ORCA 6.0.1

Table S5. [UCl_4(H-phthal)$_2$]

39				C	1.58322	-0.93380	-5.32155
U	0.00000	0.00000	0.00000	C	2.72513	-0.94138	-4.35309
Cl	0.00000	-2.62400	0.00000	H	3.18257	-1.93070	-4.26005
Cl	2.62400	0.00000	0.00000	H	3.50924	-0.23007	-4.62855
Cl	0.00000	2.62400	0.00000	N	-2.08716	0.53035	3.12325
Cl	-2.62400	0.00000	0.00000	H	-2.54982	0.43343	2.21952
O	0.00000	0.00000	-2.33266	C	-0.77960	0.32749	3.24964
O	0.00000	0.00000	2.33250	C	-0.42766	0.55619	4.64994
N	2.08311	-0.55231	-3.11913	C	0.80152	0.47498	5.28033
H	2.54629	-0.45316	-2.21591	H	1.68918	0.20388	4.72310
C	0.77745	-0.33876	-3.24760	C	0.84153	0.75534	6.63515
C	0.42463	-0.57130	-4.64704	H	1.78174	0.70512	7.16936
C	-0.80338	-0.48289	-5.27874	C	-0.31957	1.10473	7.32247
H	-1.68910	-0.20155	-4.72349	H	-0.26145	1.32004	8.38245
C	-0.84481	-0.76970	-6.63217	C	-1.54754	1.18467	6.67951
H	-1.78419	-0.71424	-7.16734	H	-2.43977	1.45981	7.22875
C	0.31379	-1.13235	-7.31684	C	-1.58873	0.90553	5.32707
H	0.25459	-1.35249	-8.37578	C	-2.73143	0.90823	4.35952
C	1.54059	-1.21943	-6.67258	H	-3.19719	1.89411	4.27145
H	2.43084	-1.50489	-7.21978	H	-3.50934	0.18911	4.63227

Table S6. [$NpCl_4$(H-phthal)$_2$]

39				H	-1.20839	-1.02429	5.97528
Np	-0.06509	-0.00454	-0.00004	Cl	-2.63339	0.11075	0.12663
Cl	2.50312	-0.11983	-0.12670	Cl	-0.15542	-2.52098	0.45875
Cl	0.02525	2.51187	-0.45883	N	0.49779	-0.27801	-4.42074
N	-0.63292	0.26704	4.41908	H	1.25330	-0.93052	-4.29886
H	-1.38834	0.91948	4.29621	O	-0.19500	-0.41245	-2.26298
O	0.06483	0.40336	2.26285	C	-0.28884	0.07498	-3.39924
C	0.15584	-0.08502	3.39895	C	-1.22772	1.07810	-3.89641
C	1.09353	-1.08875	3.89702	C	-2.24474	1.74737	-3.23534
C	2.11159	-1.75755	3.23705	H	-2.44786	1.56006	-2.18815
H	2.31684	-1.56886	2.19052	C	-2.97948	2.66155	-3.97003
C	2.84450	-2.67292	3.97207	H	-3.78081	3.21087	-3.49280
H	3.64661	-3.22197	3.49582	C	-2.69798	2.88861	-5.31535
C	2.56012	-2.90157	5.31653	H	-3.28839	3.61224	-5.86433
H	3.14914	-3.62613	5.86577	C	-1.67746	2.21037	-5.96849
C	1.53851	-2.22386	5.96851	H	-1.47076	2.39858	-7.01502
H	1.32962	-2.41340	7.01436	C	-0.94132	1.29850	-5.23844
C	0.80419	-1.31075	5.23815	C	0.20611	0.42785	-5.64795
C	-0.34399	-0.44032	5.64613	H	-0.06531	-0.26632	-6.44879
H	-0.07416	0.25293	6.44831	H	1.06966	1.01164	-5.97965

Table S7. [$PuCl_4$(H-phthal)$_2$]

39			
Pu	-0.06590	-0.00385	0.00061
Cl	2.48724	-0.09126	-0.14357
Cl	-0.00828	2.49195	-0.49221
N	-0.63108	0.28329	4.39940
H	-1.38425	0.93844	4.27776
O	0.07611	0.43666	2.24813
C	0.16222	-0.06189	3.38001
C	1.09518	-1.07190	3.87526
C	2.11628	-1.73683	3.21623
H	2.32775	-1.53940	2.17271
C	2.84179	-2.66120	3.94725
H	3.64526	-3.20815	3.47086
C	2.54758	-2.90228	5.28737
H	3.13028	-3.63425	5.83355
C	1.52378	-2.22762	5.93893
H	1.30727	-2.42662	6.98148
C	0.79693	-1.30539	5.21243
C	-0.35117	-0.43548	5.62164
H	-0.08406	0.24956	6.43176
H	-1.21885	-1.02033	5.94055
Cl	-2.61908	0.08356	0.14479
Cl	-0.12351	-2.49970	0.49344
N	0.49391	-0.29681	-4.40027
H	1.24639	-0.95287	-4.27919
O	-0.20789	-0.44435	-2.24686
C	-0.29634	0.05169	-3.37963
C	-1.22928	1.06176	-3.87477
C	-2.24779	1.72951	-3.21462
H	-2.45688	1.53470	-2.17014
C	-2.97406	2.65311	-3.94587
H	-3.77562	3.20212	-3.46868
C	-2.68304	2.89072	-5.28730
H	-3.26622	3.62216	-5.83367
C	-1.66179	2.21325	-5.93996
H	-1.44778	2.40954	-6.98354
C	-0.93423	1.29177	-5.21323
C	0.21164	0.41941	-5.62347
H	-0.05855	-0.26725	-6.43121
H	1.07923	1.00232	-5.94616

Table S8. [UCl_4(HPyO)$_2$]

29			
U	0.00061	0.00031	0.00046
Cl	2.57885	0.04287	0.03950
Cl	0.04195	-2.58493	0.08900
Cl	-2.57760	-0.04225	-0.03857
Cl	-0.04072	2.58558	-0.08807
O	0.03722	-0.07894	-2.33069
O	-0.03599	0.07956	2.33161
N	-0.53857	0.21438	4.50806
C	-0.68553	-0.39228	3.29373
C	-1.16787	-0.17185	5.64020
H	-0.95384	0.40849	6.52638
C	-2.20377	-1.90370	4.39057
H	-2.87359	-2.75409	4.34919
C	-2.01228	-1.23357	5.61607
H	-2.51762	-1.54519	6.51777
C	-1.56468	-1.50431	3.25467
H	-1.69350	-2.00404	2.30405
H	0.08637	1.00803	4.51532
N	0.53794	-0.21224	-4.50765
C	0.68625	0.39330	-3.29294
C	1.16653	0.17458	-5.63999
H	0.95143	-0.40491	-6.52647
C	2.20450	1.90475	-4.38976
H	2.87485	2.75470	-4.34819
C	2.01160	1.23576	-5.61566
H	2.51639	1.54785	-6.51751
C	1.56610	1.50478	-3.25367
H	1.69604	2.00362	-2.30273
H	-0.08752	-1.00548	-4.51510

Table S9. $[NpCl_4(HPyO)_2]$

29			
Np	0.00097	-0.00031	0.00047
Cl	2.56452	-0.00732	-0.04554
Cl	-0.00520	-2.57409	0.04936
Cl	-2.56249	0.00671	0.04647
Cl	0.00713	2.57335	-0.04843
O	-0.04052	-0.04400	-2.30472
O	0.04246	0.04339	2.30563
N	-0.52104	0.21843	4.46203
C	-0.63524	-0.41319	3.25808
C	-1.17860	-0.14534	5.58519
H	-0.98889	0.45396	6.46422
C	-2.17793	-1.90486	4.34518
H	-2.84518	-2.75722	4.30339
C	-2.02021	-1.20959	5.56074
H	-2.54870	-1.50372	6.45504
C	-1.50970	-1.52757	3.21803
H	-1.61202	-2.04704	2.27467
H	0.10024	1.01513	4.46787
N	0.52109	-0.21668	-4.46178
C	0.63620	0.41374	-3.25729
C	1.17761	0.14838	-5.58514
H	0.98729	-0.45006	-6.46461
C	2.17760	1.90688	-4.34420
H	2.84467	2.75937	-4.30213
C	2.01902	1.21279	-5.56031
H	2.54670	1.50792	-6.45476
C	1.51041	1.52831	-3.21686
H	1.61340	2.04686	-2.27309
H	-0.10004	-1.01348	-4.46789

Table S10. $[PuCl_4(HPyO)_2]$

29			
Pu	0.00117	-0.00026	0.00064
Cl	2.54972	0.00298	-0.04038
Cl	0.00524	-2.55934	0.05189
Cl	-2.54746	-0.00349	0.04167
Cl	-0.00291	2.55897	-0.05061
O	-0.03570	-0.04626	-2.29339
O	0.03803	0.04574	2.29459
N	-0.51862	0.21918	4.45258
C	-0.63724	-0.41079	3.24788
C	-1.17313	-0.14555	5.57737
H	-0.98004	0.45232	6.45665
C	-2.17840	-1.90208	4.33770
H	-2.84678	-2.75363	4.29698
C	-2.01583	-1.20877	5.55402
H	-2.54172	-1.50369	6.44960
C	-1.51375	-1.52398	3.20894
H	-1.61969	-2.04052	2.26456
H	0.10370	1.01499	4.45755
N	0.51880	-0.21763	-4.45208
C	0.63841	0.41137	-3.24696
C	1.17197	0.14833	-5.57724
H	0.97820	-0.44887	-6.45682
C	2.17783	1.90421	-4.33714
H	2.84590	2.75600	-4.29640
C	2.01426	1.21188	-5.55388
H	2.53907	1.50778	-6.44977
C	1.51453	1.52486	-3.20799
H	1.62124	2.04069	-2.26331
H	-0.10323	-1.01367	-4.45708

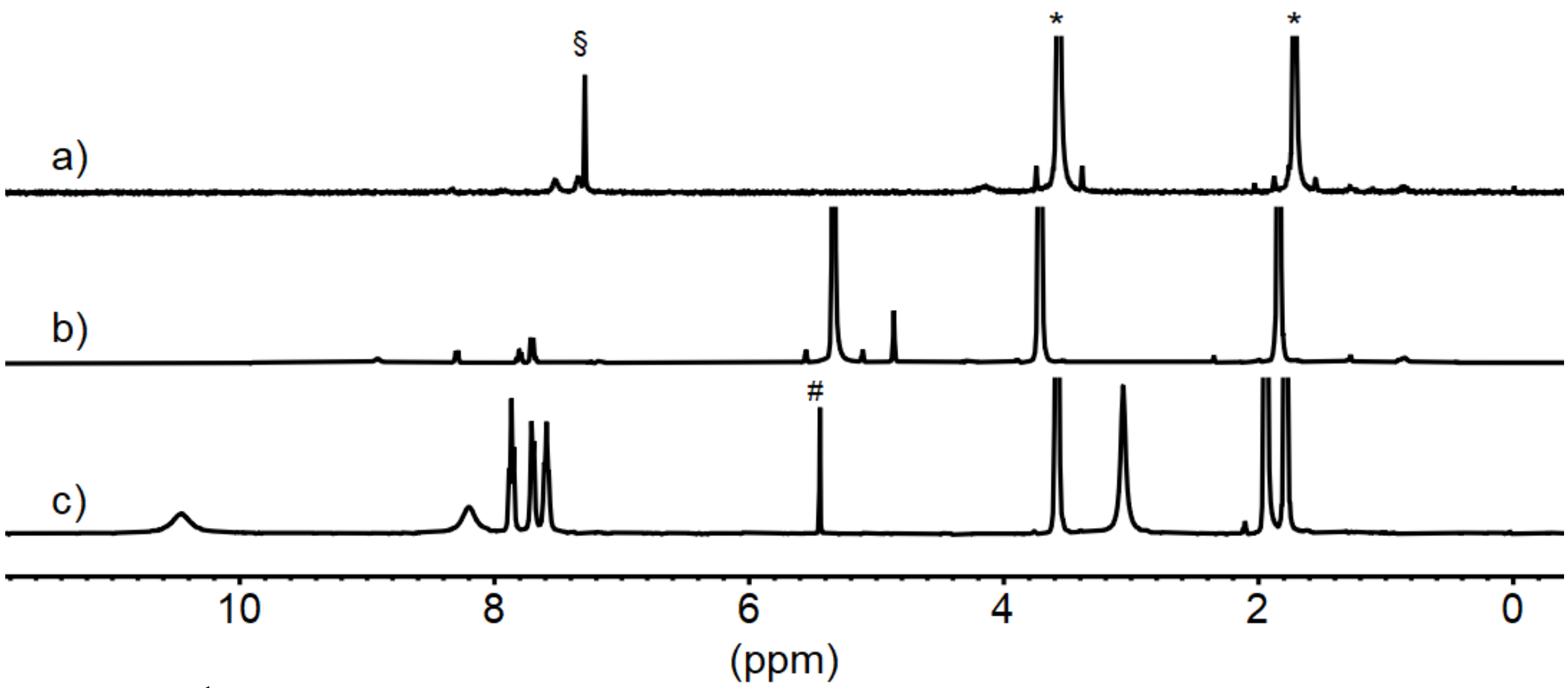

Figure S26. ^{1}H NMR spectra of $[UCl_4(H\text{-}phthal)_2]\cdot 2THF$ in different solvents: a) THF-d_8, b) CD_2Cl_2, c) CD_3CN demonstrating the varying solubility of the complex. Solvent impurities are labeled with § ($CHCl_3$), # (CH_2Cl_2), and * (THF). These shifts vary slightly based on the used NMR solvent.

Reaction path calculations

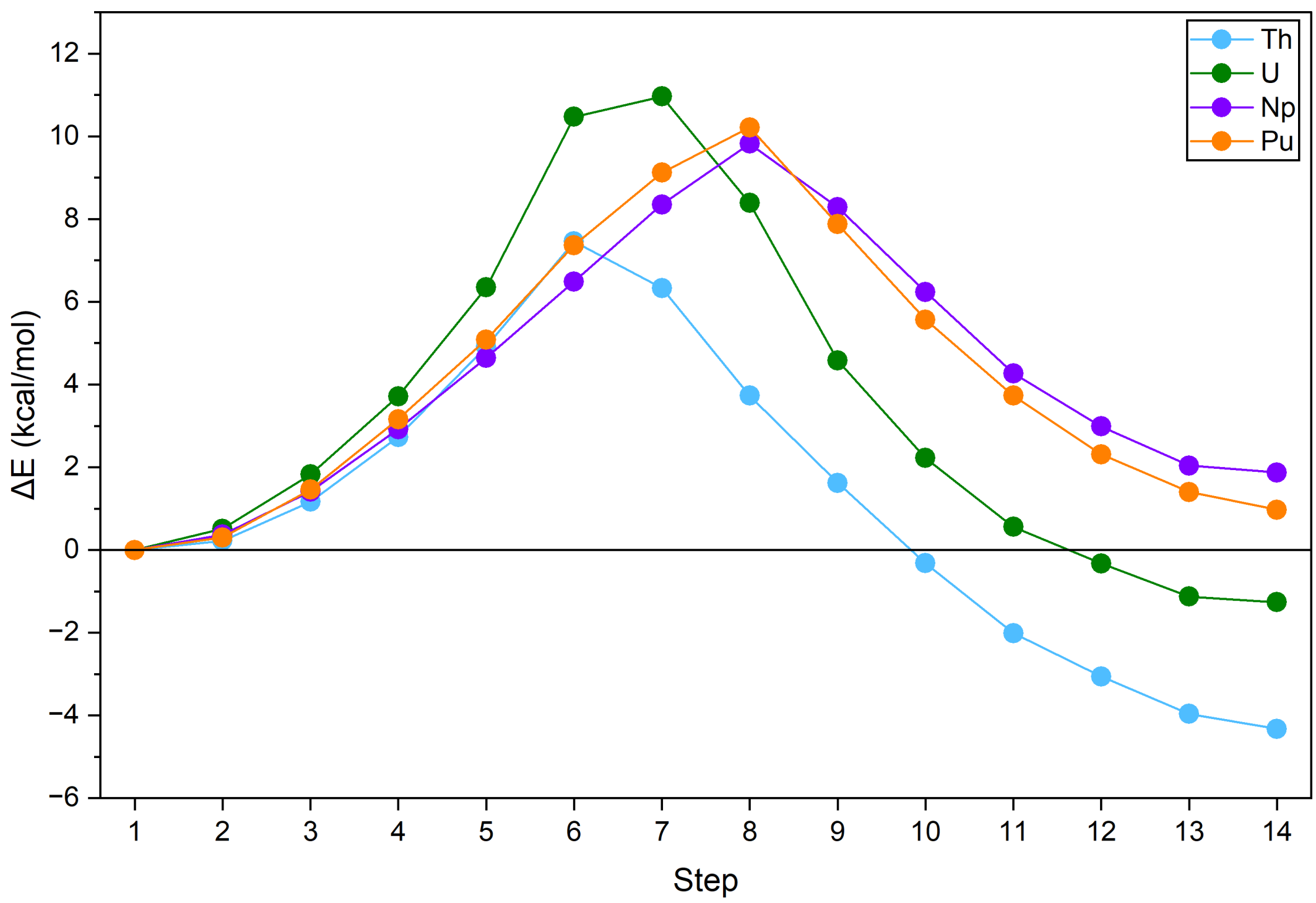

Figure S27. Calculated reaction paths for the coordination of an initially H-bound THF molecule to $[AnCl_4(H\text{-}phthal)_2]$. Performed via Turbomole's WOELFLING module (def-SVP/PBE0/COSMO(THF)).

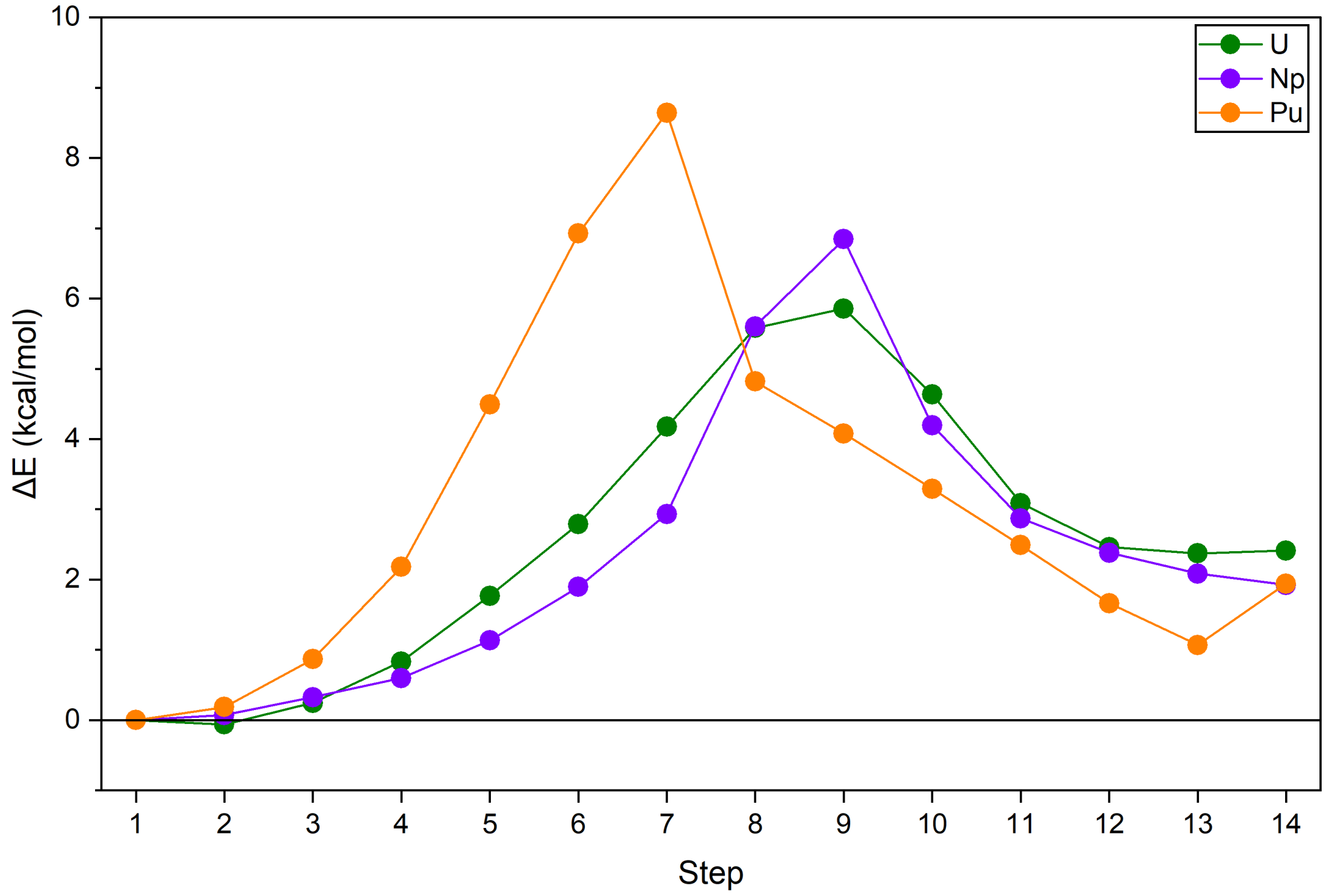

Figure S28. Calculated reaction paths for the coordination of an initially H-bound NCMe molecule to $[AnCl_4(H\text{-phthal})_2]$. Performed via Turbomole's WOELFLING module (def-SVP/PBE0/COSMO(MeCN)).

Cartesian coordinates (in Å) of reactants and hypothetical solvent-coordinated product complexes from reaction path calculations performed via *woelfling*

Table S11. $[ThCl_4(H\text{-}phthal)_2]\cdot THF$

52				C	-3.59058	2.20286	0.20822
				C	-4.47369	1.03758	0.29232
Th	-0.21078	1.46830	0.48703	C	-4.23031	-0.24265	0.78844
Cl	0.45808	3.22803	-1.39468	H	-3.25673	-0.50819	1.20726
Cl	-1.22721	-0.15459	-1.37404	C	-5.27808	-1.15735	0.72935
N	3.61990	-0.91904	0.04099	H	-5.13394	-2.17204	1.10689
H	3.03191	-1.78464	0.03462	C	-6.51996	-0.79158	0.18917
O	1.85439	0.51264	0.26673	H	-7.32405	-1.53084	0.15415
C	3.08894	0.28620	0.14176	C	-6.75369	0.49415	-0.30425
C	4.15936	1.28236	0.08258	H	-7.72642	0.76390	-0.72156
C	4.10187	2.67360	0.14379	C	-5.71023	1.41102	-0.24592
H	3.14721	3.19320	0.25622	C	-5.63795	2.84196	-0.68463
C	5.30811	3.36383	0.05397	H	-6.35913	3.48443	-0.15349
H	5.31328	4.45516	0.09633	H	-5.81111	2.96045	-1.76677
C	6.51967	2.67148	-0.09112	O	1.92556	-3.03247	-0.08735
H	7.45136	3.23859	-0.15934	C	1.20530	-3.04353	-1.32402
C	6.56480	1.27615	-0.15130	H	1.83859	-3.51762	-2.08892
H	7.51714	0.75337	-0.26525	H	0.98248	-2.00676	-1.63288
C	5.36253	0.58321	-0.06313	C	-0.07637	-3.80106	-1.02883
C	5.06157	-0.88526	-0.09667	H	0.09292	-4.88801	-1.09691
H	5.37522	-1.35840	-1.04187	H	-0.88601	-3.52931	-1.72074
H	5.54572	-1.43426	0.72788	C	-0.35538	-3.37798	0.40979
Cl	-0.79599	-0.30586	2.40389	H	-0.82720	-2.38442	0.42519
Cl	0.68157	3.17492	2.31990	H	-1.00534	-4.07607	0.95619
N	-4.27655	3.19857	-0.34059	C	1.04181	-3.30225	1.00944
H	-3.87251	4.11478	-0.50689	H	1.13308	-2.49835	1.75700
O	-2.39170	2.28647	0.57722	H	1.34848	-4.25466	1.47482

Table S12. [$ThCl_4$(H-phthal)$_2$(THF)]

52				C	-3.83924	1.29955	-0.37939
				C	-5.03631	0.64710	0.15691
Th	-0.68183	0.44413	-1.42537	C	-5.50606	-0.64995	-0.03432
Cl	0.45924	0.73038	-3.80568	H	-4.95915	-1.35106	-0.66942
Cl	-1.79711	-1.81324	-2.37397	C	-6.68496	-1.00289	0.61792
N	2.12195	0.40896	1.47143	H	-7.09319	-2.00908	0.49936
H	1.18211	0.21592	1.82398	C	-7.35624	-0.07624	1.43003
O	1.39594	1.24127	-0.54393	H	-8.27943	-0.37853	1.93077
C	2.30172	0.92625	0.25684	C	-6.87307	1.22164	1.61667
C	3.74809	1.02998	0.02875	H	-7.40750	1.92856	2.25546
C	4.44573	1.49225	-1.08485	C	-5.69577	1.57651	0.96700
H	3.91243	1.86986	-1.96062	C	-4.91708	2.85926	0.96239
C	5.83674	1.44616	-1.03229	H	-4.56486	3.13948	1.96878
H	6.42567	1.79599	-1.88300	H	-5.50032	3.70519	0.56271
C	6.49195	0.95296	0.10592	O	1.01525	-1.42810	-1.06109
H	7.58434	0.92672	0.12182	C	2.17595	-1.61115	-1.89354
C	5.78240	0.49344	1.21836	H	2.63100	-0.62718	-2.06277
H	6.30861	0.11116	2.09605	H	1.85718	-2.02097	-2.86579
C	4.39333	0.53663	1.16709	C	3.02984	-2.58597	-1.11464
C	3.35664	0.11653	2.16679	H	3.56739	-2.05887	-0.30893
H	3.42471	-0.95536	2.41766	H	3.77200	-3.09081	-1.74806
H	3.42743	0.68395	3.10945	C	1.97089	-3.52724	-0.54860
Cl	-1.05365	-0.07320	1.22446	H	1.66971	-4.25753	-1.31504
Cl	-0.97707	3.15374	-1.34107	H	2.30710	-4.08406	0.33693
N	-3.80332	2.54043	0.09491	C	0.81462	-2.59042	-0.22507
H	-3.03492	3.16871	-0.14692	H	-0.17351	-3.01306	-0.45509
O	-2.99331	0.78691	-1.14773	H	0.81793	-2.24471	0.81873

Table S13. $[UCl_4(H\text{-phthal})_2]\cdot THF$

52				C	-3.63417	2.07634	-0.00726
				C	-4.52429	0.91690	0.10674
U	-0.26180	1.26788	-0.03557	C	-4.22778	-0.43527	0.27467
Cl	0.42985	2.84636	-1.97696	H	-3.19364	-0.77670	0.34574
Cl	-1.15337	-0.50792	-1.72194	C	-5.30160	-1.31843	0.35077
N	3.70153	-0.83101	0.23073	H	-5.11638	-2.38676	0.48256
H	3.20359	-1.74901	0.24846	C	-6.62133	-0.85275	0.26084
O	1.80169	0.38574	-0.10236	H	-7.44467	-1.56865	0.32357
C	3.05127	0.30453	0.03552	C	-6.90806	0.50418	0.09388
C	4.02071	1.40262	0.00657	H	-7.94123	0.85239	0.02648
C	3.83286	2.77134	-0.17992	C	-5.83886	1.38927	0.01751
H	2.83413	3.18417	-0.33733	C	-5.81232	2.87753	-0.15510
C	4.96834	3.57769	-0.15998	H	-6.32039	3.40628	0.66808
H	4.86960	4.65615	-0.30207	H	-6.27560	3.20058	-1.10181
C	6.23993	3.02055	0.04019	O	2.18424	-3.07880	0.06856
H	7.11337	3.67736	0.05201	C	1.59005	-3.11593	-1.23178
C	6.41684	1.64654	0.22211	H	2.31958	-3.55159	-1.93066
H	7.41432	1.22766	0.37368	H	1.34417	-2.08997	-1.56066
C	5.28624	0.83749	0.20097	C	0.32702	-3.93834	-1.06108
C	5.13215	-0.64608	0.35296	H	0.56121	-5.01491	-1.08908
H	5.66910	-1.20666	-0.43013	H	-0.41752	-3.72035	-1.83971
H	5.49229	-1.00981	1.32960	C	-0.12622	-3.50774	0.33042
Cl	-0.92692	-0.29131	1.94425	H	-0.65986	-2.54809	0.26941
Cl	0.62204	3.03083	1.64511	H	-0.78435	-4.23694	0.82389
N	-4.39191	3.15951	-0.15235	C	1.19164	-3.32397	1.07411
H	-3.99930	4.09006	-0.25151	H	1.15728	-2.47121	1.77136
O	-2.38139	2.09483	0.02196	H	1.48794	-4.22660	1.63456

Table S14. [UCl_4(H-phthal)$_2$(THF)]

52			
U	-0.61868	0.46162	-1.06744
Cl	-0.07999	0.98104	-3.53467
Cl	-1.50349	-1.87259	-1.91737
N	2.32042	0.74155	1.49823
H	1.40185	0.59331	1.91950
O	1.50368	1.34042	-0.56681
C	2.44337	1.09838	0.22006
C	3.87647	1.12813	-0.09530
C	4.52132	1.43242	-1.29191
H	3.95015	1.72559	-2.17595
C	5.91135	1.34668	-1.30977
H	6.45928	1.57483	-2.22667
C	6.61771	0.96974	-0.15791
H	7.70815	0.90968	-0.19785
C	5.96116	0.66868	1.03815
H	6.52660	0.37588	1.92581
C	4.57303	0.75081	1.05689
C	3.58498	0.48655	2.15413
H	3.63614	-0.55151	2.52330
H	3.72811	1.15513	3.01900
Cl	-0.87894	0.03134	1.52501
Cl	-1.01200	3.06441	-0.65488
N	-3.80836	2.17656	0.58377
H	-2.98202	2.77689	0.57420
O	-2.92592	0.63151	-0.87412
C	-3.85165	1.07493	-0.16032
C	-5.18042	0.47577	0.00661
C	-5.70733	-0.69036	-0.54254
H	-5.10462	-1.31142	-1.20953
C	-7.01563	-1.02305	-0.19888
H	-7.47233	-1.92850	-0.60479
C	-7.75553	-0.20493	0.66801
H	-8.78024	-0.48778	0.92178
C	-7.21391	0.96040	1.21713
H	-7.80342	1.58355	1.89371
C	-5.90810	1.29489	0.87483
C	-5.04278	2.44942	1.28734
H	-4.87895	2.48139	2.37719
H	-5.46473	3.42140	0.98244
O	1.15138	-1.29098	-0.66120
C	2.04454	-1.59405	-1.74490
H	2.48651	-0.64987	-2.09091
H	1.46585	-2.04466	-2.56929
C	3.02479	-2.57216	-1.13710
H	3.76445	-2.03180	-0.52347
H	3.56480	-3.15342	-1.89695
C	2.09772	-3.41903	-0.26910
H	1.59584	-4.17813	-0.88829
H	2.61672	-3.93742	0.54896
C	1.08094	-2.40768	0.24839
H	0.05076	-2.79009	0.25271
H	1.32097	-2.02345	1.25077

Table S15. $[NpCl_4(H\text{-}phthal)_2]\cdot THF$

52				C	-3.50608	2.15251	0.19718
				C	-4.41338	1.00853	0.32051
Np	-0.22672	1.32513	0.33185	C	-4.18581	-0.27139	0.82458
Cl	0.35736	2.98314	-1.55576	H	-3.20580	-0.55561	1.21446
Cl	-1.31011	-0.27841	-1.38451	C	-5.25740	-1.16005	0.81248
N	3.64239	-0.92782	0.06202	H	-5.12540	-2.17349	1.19783
H	3.11562	-1.82998	0.04041	C	-6.50761	-0.76971	0.31026
O	1.76161	0.35678	0.06019	H	-7.33064	-1.48871	0.31178
C	3.01588	0.23708	0.05869	C	-6.72599	0.51602	-0.19001
C	4.01539	1.30860	0.04814	H	-7.70577	0.80622	-0.57604
C	3.85897	2.69384	0.03201	C	-5.65859	1.40679	-0.17857
H	2.86581	3.14782	0.02737	C	-5.56855	2.83194	-0.63256
C	5.01863	3.46518	0.02118	H	-6.25669	3.49457	-0.08251
H	4.94428	4.55481	0.00877	H	-5.77659	2.94577	-1.70909
C	6.28327	2.85859	0.02572	O	2.03176	-3.10503	-0.13994
H	7.17625	3.48861	0.01763	C	1.30725	-3.01161	-1.36965
C	6.42847	1.46901	0.03827	H	1.93875	-3.42087	-2.17225
H	7.42052	1.01162	0.03891	H	1.08194	-1.95292	-1.59180
C	5.27345	0.69507	0.04793	C	0.02752	-3.78967	-1.12955
C	5.08345	-0.79193	0.05491	H	0.20199	-4.86964	-1.26341
H	5.51914	-1.27543	-0.83513	H	-0.77990	-3.47744	-1.80675
H	5.52639	-1.26974	0.94447	C	-0.25971	-3.45321	0.33050
Cl	-0.71858	-0.33989	2.26176	H	-0.74798	-2.47009	0.39842
Cl	0.73604	2.99479	2.03786	H	-0.90031	-4.19139	0.83368
N	-4.18863	3.15904	-0.33846	C	1.13460	-3.38677	0.94304
H	-3.76819	4.06306	-0.52862	H	1.21495	-2.59199	1.70221
O	-2.29692	2.22170	0.52996	H	1.43964	-4.34343	1.39959

Table S16. [$NpCl_4$(H-phthal)$_2$(THF)]

52				C	-3.69664	1.20748	-0.16776
				C	-5.02082	0.62629	0.06460
Np	-0.57214	0.41180	-1.09852	C	-5.59304	-0.53013	-0.45978
Cl	-0.37073	1.15046	-3.54784	H	-5.03719	-1.15652	-1.16150
Cl	-1.52191	-1.84243	-2.06682	C	-6.88434	-0.84630	-0.04462
N	2.40517	0.62459	1.26798	H	-7.37536	-1.74374	-0.42745
H	1.48114	0.41251	1.65167	C	-7.56349	-0.02193	0.86524
O	1.67587	1.01994	-0.88008	H	-8.57604	-0.29257	1.17490
C	2.57493	0.93732	-0.01645	C	-6.97691	1.13368	1.38800
C	4.01095	1.15094	-0.23363	H	-7.51935	1.76111	2.09899
C	4.69764	1.49030	-1.39702	C	-5.68739	1.45160	0.97580
H	4.16203	1.64807	-2.33598	C	-4.78294	2.59003	1.34607
C	6.08175	1.61944	-1.30892	H	-4.55256	2.60919	2.42401
H	6.66150	1.88397	-2.19603	H	-5.20597	3.57131	1.07496
C	6.74134	1.41430	-0.08807	O	1.03659	-1.50767	-0.58259
H	7.82797	1.52235	-0.04505	C	1.89132	-1.92730	-1.66168
C	6.04272	1.07553	1.07355	H	2.60229	-1.11714	-1.87131
H	6.57179	0.91934	2.01650	H	1.26897	-2.10164	-2.55619
C	4.66099	0.94459	0.98712	C	2.52585	-3.21065	-1.16868
C	3.63747	0.59515	2.02620	H	3.37552	-2.98712	-0.50327
H	3.80808	-0.40190	2.46544	H	2.88918	-3.83877	-1.99376
H	3.61249	1.32446	2.85272	C	1.37280	-3.83106	-0.38865
Cl	-0.82367	-0.01713	1.48963	H	0.65198	-4.28936	-1.08330
Cl	-0.63062	2.97035	-0.43082	H	1.68830	-4.59271	0.33793
N	-3.59533	2.30580	0.56938	C	0.74261	-2.62363	0.28251
H	-2.74036	2.86687	0.55270	H	-0.34642	-2.70424	0.39178
O	-2.81030	0.75058	-0.92968	H	1.18432	-2.40541	1.26780

Table S17. [$PuCl_4$(H-phthal)$_2$]·THF

52				C	-3.48105	2.13913	0.23101
				C	-4.40315	1.00356	0.31824
Pu	-0.22114	1.28325	0.37280	C	-4.19681	-0.28921	0.79769
Cl	0.40393	2.94946	-1.47494	H	-3.22350	-0.59165	1.18987
Cl	-1.35613	-0.27060	-1.33526	C	-5.27867	-1.16433	0.75488
N	3.65157	-0.94092	0.07157	H	-5.16309	-2.18727	1.11990
H	3.12918	-1.84505	0.05355	C	-6.51818	-0.74861	0.24648
O	1.75937	0.32348	0.08695	H	-7.34965	-1.45747	0.22331
C	3.01464	0.21883	0.07222	C	-6.71521	0.54970	-0.22956
C	4.00538	1.29858	0.04708	H	-7.68670	0.85967	-0.62113
C	3.83865	2.68258	0.02787	C	-5.63742	1.42703	-0.18717
H	2.84148	3.12675	0.03353	C	-5.52518	2.85994	-0.61089
C	4.99238	3.46239	-0.00048	H	-6.21261	3.51943	-0.05619
H	4.90961	4.55138	-0.01637	H	-5.71833	2.99776	-1.68741
C	6.26161	2.86567	-0.00925	O	2.02431	-3.10655	-0.12870
H	7.14968	3.50233	-0.03084	C	1.29754	-2.99142	-1.35448
C	6.41732	1.47728	0.00669	H	1.92984	-3.38221	-2.16547
H	7.41265	1.02716	-0.00334	H	1.06623	-1.92961	-1.55604
C	5.26834	0.69481	0.03320	C	0.02262	-3.77977	-1.12461
C	5.09105	-0.79367	0.04637	H	0.20555	-4.85720	-1.26724
H	5.51999	-1.27572	-0.84774	H	-0.78588	-3.46754	-1.80052
H	5.54909	-1.26524	0.93163	C	-0.26955	-3.45640	0.33754
Cl	-0.78106	-0.37483	2.26847	H	-0.77106	-2.48047	0.41026
Cl	0.81812	2.88500	2.07641	H	-0.90156	-4.20562	0.83521
N	-4.14538	3.16428	-0.29324	C	1.12322	-3.37494	0.95440
H	-3.71225	4.06720	-0.45802	H	1.19509	-2.56828	1.70208
O	-2.27694	2.19030	0.58288	H	1.43208	-4.32235	1.42677

Table S18. [$PuCl_4$(H-phthal)$_2$(THF)]

52				C	-3.62310	1.10885	-0.08721
				C	-5.00729	0.65960	0.08668
Pu	-0.53838	0.21107	-1.12649	C	-5.67945	-0.41123	-0.49716
Cl	-0.29099	0.70716	-3.61604	H	-5.17000	-1.06762	-1.20657
Cl	-1.53489	-2.05579	-1.96824	C	-7.00986	-0.60435	-0.13262
N	2.41173	0.63814	1.27492	H	-7.57855	-1.43103	-0.56424
H	1.49510	0.39505	1.66063	C	-7.62947	0.25441	0.78768
O	1.67135	0.87829	-0.88994	H	-8.67445	0.08136	1.05683
C	2.56840	0.89722	-0.02220	C	-6.94341	1.32348	1.37014
C	3.98866	1.19455	-0.24724	H	-7.44105	1.98015	2.08752
C	4.65810	1.52181	-1.42389	C	-5.61532	1.51918	1.00712
H	4.11911	1.59464	-2.37134	C	-4.61220	2.54627	1.44327
C	6.02937	1.75153	-1.33781	H	-4.41960	2.51018	2.52820
H	6.59537	2.01139	-2.23514	H	-4.92358	3.57340	1.19094
C	6.69316	1.65430	-0.10597	O	1.05951	-1.64445	-0.54736
H	7.76931	1.84005	-0.06468	C	1.90776	-2.06625	-1.63412
C	6.01153	1.32652	1.06889	H	2.67477	-1.29598	-1.79010
H	6.54362	1.25549	2.02038	H	1.29148	-2.14888	-2.54553
C	4.64271	1.09587	0.98473	C	2.44892	-3.41530	-1.20520
C	3.63793	0.73134	2.03734	H	3.32944	-3.28702	-0.55536
H	3.87320	-0.22755	2.52854	H	2.74215	-4.03425	-2.06442
H	3.55866	1.49928	2.82458	C	1.27273	-3.97927	-0.41696
Cl	-0.74564	-0.05444	1.48479	H	0.49368	-4.34134	-1.10553
Cl	-0.60730	2.80080	-0.60122	H	1.54754	-4.79569	0.26523
N	-3.43438	2.16411	0.69517	C	0.76609	-2.75679	0.32245
H	-2.53955	2.65845	0.68849	H	-0.31423	-2.77171	0.51166
O	-2.76344	0.59156	-0.83796	H	1.29475	-2.58656	1.27437

Table S19. $[UCl_4(H\text{-}phthal)_2]\cdot NCMe$

45			
U	0.13322	-0.89409	-0.20247
Cl	-0.50388	-2.71360	-1.95767
Cl	1.09796	0.63377	-2.07140
N	-3.77548	1.24007	-0.05728
H	-3.25745	2.13445	-0.07626
O	-1.93006	-0.04897	-0.42913
C	-3.16770	0.07782	-0.22510
C	-4.16522	-0.98969	-0.13275
C	-4.02361	-2.37182	-0.24939
H	-3.04722	-2.81953	-0.44761
C	-5.17459	-3.14293	-0.10643
H	-5.11373	-4.23041	-0.18919
C	-6.41594	-2.53787	0.14271
H	-7.30237	-3.16752	0.25335
C	-6.54691	-1.15107	0.25151
H	-7.52153	-0.69548	0.44069
C	-5.40016	-0.37728	0.10998
C	-5.19932	1.10671	0.17021
H	-5.77345	1.64283	-0.60319
H	-5.48021	1.52991	1.14866
Cl	0.77106	0.89489	1.57150
Cl	-0.84307	-2.42281	1.66218
N	4.18760	-2.90762	0.11306
H	3.76488	-3.83037	0.13667
O	2.21660	-1.75772	0.03588
C	3.47194	-1.79071	0.06275
C	4.40172	-0.65805	0.04836
C	4.15396	0.71368	-0.00046
H	3.13293	1.09865	-0.03447
C	5.25912	1.56100	-0.00160
H	5.11259	2.64279	-0.03933
C	6.56145	1.04165	0.04539
H	7.41019	1.73013	0.04386
C	6.79898	-0.33401	0.09477
H	7.81856	-0.72414	0.13203
C	5.69844	-1.18364	0.09531
C	5.61746	-2.67838	0.13987
H	6.06507	-3.09943	1.05487
H	6.10716	-3.15420	-0.72535
C	-0.54395	5.64852	-0.01811
C	-1.46632	4.53424	-0.06432
N	-2.20546	3.64660	-0.10203
H	-0.25983	5.85248	1.02388
H	-1.02143	6.54186	-0.44482
H	0.35801	5.40930	-0.59913

Table S20. [UCl_4(H-phthal)$_2$(NCMe)]

45				N	3.53233	-2.59749	0.66135
				H	2.86486	-3.31267	0.93038
U	0.14997	0.36002	-0.13669	O	1.98021	-0.97915	0.23374
Cl	-0.15088	-1.66329	-1.83211	C	3.16072	-1.37307	0.28711
Cl	1.36632	1.57844	-2.09675	C	4.38144	-0.61689	-0.02426
N	-4.02135	1.40337	0.00425	C	4.53853	0.70257	-0.44592
H	-3.71947	2.37078	0.03381	H	3.66977	1.34218	-0.61234
O	-2.07123	0.57564	-0.86517	C	5.83923	1.15566	-0.65442
C	-3.23905	0.42249	-0.44453	H	6.00924	2.18083	-0.99144
C	-4.00770	-0.82517	-0.35837	C	6.93666	0.30794	-0.44206
C	-3.63996	-2.12727	-0.69291	H	7.94648	0.68878	-0.61463
H	-2.64282	-2.33437	-1.08976	C	6.76809	-1.01232	-0.01784
C	-4.58810	-3.12833	-0.49654	H	7.63169	-1.66134	0.14466
H	-4.34416	-4.16417	-0.74370	C	5.47067	-1.46808	0.18967
C	-5.85676	-2.82242	0.01915	C	4.96529	-2.80291	0.64633
H	-6.58111	-3.62765	0.16577	H	5.33772	-3.07353	1.64794
C	-6.21475	-1.51363	0.35206	H	5.24074	-3.61564	-0.04586
H	-7.20660	-1.29053	0.75196	C	-1.41310	5.29459	0.22932
C	-5.26975	-0.51197	0.15678	C	-1.03385	3.90553	0.11839
C	-5.33680	0.96235	0.41818	N	-0.72390	2.79676	0.03185
H	-6.12418	1.46350	-0.16801	H	-1.62560	5.53386	1.28115
H	-5.51292	1.19153	1.48220	H	-2.30757	5.48937	-0.37899
Cl	1.55536	1.65061	1.67437	H	-0.58717	5.92645	-0.12734
Cl	-1.12652	-0.78135	1.81926				

Table S21. $[NpCl_4(H\text{-}phthal)_2]\cdot NCMe$

45				N	4.20263	-2.86916	0.23129
				H	3.78957	-3.79426	0.29713
Np	0.16775	-0.88552	-0.29540	O	2.22528	-1.76469	-0.03460
Cl	-0.41303	-2.75127	-1.99815	C	3.47791	-1.77029	0.06024
Cl	1.06008	0.60521	-2.20784	C	4.39534	-0.62797	0.00837
N	-3.74305	1.17458	-0.02128	C	4.13734	0.73323	-0.15297
H	-3.25844	2.08787	-0.01742	H	3.11666	1.10265	-0.26664
O	-1.88123	-0.03799	-0.53690	C	5.23245	1.59336	-0.16249
C	-3.11060	0.03929	-0.26545	H	5.07660	2.66737	-0.28623
C	-4.07309	-1.06080	-0.17105	C	6.53608	1.09686	-0.01500
C	-3.90078	-2.43225	-0.35456	H	7.37692	1.79485	-0.02693
H	-2.92426	-2.84451	-0.61823	C	6.78444	-0.26810	0.14699
C	-5.02289	-3.24150	-0.19469	H	7.80478	-0.64082	0.26141
H	-4.93635	-4.32220	-0.32804	C	5.69370	-1.13021	0.15782
C	-6.26756	-2.68401	0.13548	C	5.62648	-2.61801	0.31259
H	-7.13117	-3.34294	0.25566	H	6.02989	-2.96027	1.27958
C	-6.43013	-1.30765	0.31149	H	6.16567	-3.15226	-0.48650
H	-7.40652	-0.88839	0.56472	C	-0.85541	5.82586	0.03275
C	-5.31203	-0.49607	0.15306	C	-1.66227	4.62447	0.01574
C	-5.15042	0.98928	0.26468	N	-2.30868	3.66650	0.00131
H	-5.77463	1.53712	-0.46029	H	-0.48407	6.00931	1.05087
H	-5.39755	1.36645	1.27065	H	-1.46183	6.68330	-0.29154
Cl	0.77821	0.94201	1.42174	H	-0.00064	5.70926	-0.64846
Cl	-0.77115	-2.35766	1.60709				

Table S22. [$NpCl_4$(H-phthal)$_2$(NCMe)]

45				N	3.18802	-1.69960	-0.83514
				H	2.36070	-2.02488	-1.33670
Np	0.09617	0.54449	0.01622	O	2.00343	-0.48563	0.71340
Cl	-0.01624	-1.55949	-1.59389	C	3.08131	-0.87903	0.20129
Cl	1.82218	1.43347	-1.73760	C	4.43257	-0.52024	0.63527
N	-4.14092	1.25168	0.41691	C	4.84692	0.31788	1.66794
H	-3.90181	2.23329	0.50831	H	4.11587	0.82123	2.30509
O	-2.14832	0.60132	-0.50338	C	6.21847	0.48934	1.83866
C	-3.29990	0.34863	-0.08303	H	6.59196	1.13872	2.63367
C	-3.97905	-0.95039	-0.05880	C	7.12981	-0.16422	0.99488
C	-3.52467	-2.20506	-0.46123	H	8.20100	-0.01261	1.14975
H	-2.51740	-2.32662	-0.86658	C	6.70232	-0.99928	-0.04082
C	-4.40210	-3.27661	-0.31650	H	7.42593	-1.49531	-0.69171
H	-4.09025	-4.27922	-0.61799	C	5.33306	-1.17016	-0.21504
C	-5.68585	-3.08463	0.21722	C	4.55722	-1.96559	-1.22216
H	-6.35375	-3.94347	0.32115	H	4.78052	-3.04395	-1.17586
C	-6.12942	-1.82301	0.62088	H	4.74358	-1.62760	-2.25483
H	-7.13043	-1.68967	1.03746	C	-1.38526	5.23169	-1.57517
C	-5.25590	-0.75035	0.47560	C	-0.99159	3.91859	-1.11932
C	-5.42086	0.70041	0.81229	N	-0.66807	2.87225	-0.75587
H	-6.24400	1.17389	0.25262	H	-1.10612	5.98134	-0.82116
H	-5.60450	0.86530	1.88655	H	-2.47252	5.26105	-1.73344
Cl	0.67574	2.22565	1.91905	H	-0.87423	5.45976	-2.52139
Cl	-0.96361	-0.89337	1.91338				

Table S23. [$PuCl_4$(H-phthal)$_2$]·NCMe

45			
Pu	0.17678	-0.86810	-0.22123
Cl	-0.47105	-2.69486	-1.91842
Cl	1.09690	0.60425	-2.11404
N	-3.76462	1.16732	0.02741
H	-3.29345	2.08660	0.05700
O	-1.86224	-0.00496	-0.41135
C	-3.10324	0.04321	-0.19495
C	-4.05230	-1.07245	-0.14501
C	-3.85383	-2.44118	-0.32277
H	-2.86065	-2.83857	-0.54127
C	-4.97081	-3.26620	-0.21554
H	-4.86281	-4.34524	-0.34693
C	-6.23683	-2.72752	0.05918
H	-7.09591	-3.39853	0.13807
C	-6.42552	-1.35421	0.23223
H	-7.41767	-0.94885	0.44338
C	-5.31271	-0.52701	0.12683
C	-5.17910	0.95967	0.25380
H	-5.78232	1.50326	-0.49182
H	-5.47258	1.32501	1.25174
Cl	0.87611	0.92295	1.47964
Cl	-0.79728	-2.33457	1.64848
N	4.20829	-2.85491	0.23461
H	3.79315	-3.77794	0.31380
O	2.22884	-1.74725	0.01277
C	3.48290	-1.75362	0.07862
C	4.40312	-0.61423	0.00733
C	4.14705	0.74849	-0.14361
H	3.12500	1.11964	-0.23368
C	5.24490	1.60475	-0.17252
H	5.09026	2.67976	-0.28930
C	6.54952	1.10356	-0.05287
H	7.39243	1.79869	-0.07896
C	6.79606	-0.26278	0.09998
H	7.81702	-0.63953	0.19392
C	5.70269	-1.12110	0.12964
C	5.63438	-2.60901	0.28220
H	6.06119	-2.95642	1.23718
H	6.15161	-3.14160	-0.53250
C	-0.91818	5.84328	0.00507
C	-1.73202	4.64728	0.04901
N	-2.38364	3.69332	0.08378
H	-0.51349	6.05351	1.00515
H	-1.52958	6.69505	-0.32477
H	-0.08636	5.70104	-0.69928

Table S24. [$PuCl_4$(H-phthal)$_2$(NCMe)]

45				N	3.11922	-1.82026	0.13190
				H	2.21743	-2.29158	0.05151
Pu	0.24897	0.81941	0.63745	O	2.27092	0.00011	1.24353
Cl	-0.15830	-1.75587	0.18842	C	3.21576	-0.62566	0.69830
Cl	1.39487	0.89670	-1.67588	C	4.60233	-0.17526	0.57643
N	-3.07077	-0.85871	1.89725	C	5.19648	1.01228	0.99756
H	-2.36641	-0.75688	2.62882	H	4.60856	1.77929	1.50741
O	-2.05111	0.59844	0.43363	C	6.55278	1.17644	0.72706
C	-2.95955	-0.20451	0.74641	H	7.06343	2.09146	1.03552
C	-4.09986	-0.58789	-0.09439	C	7.27319	0.17521	0.05736
C	-4.43638	-0.18238	-1.38348	H	8.33663	0.32882	-0.14246
H	-3.81274	0.53287	-1.92470	C	6.66499	-1.01028	-0.36417
C	-5.58845	-0.72665	-1.94639	H	7.24063	-1.77629	-0.88871
H	-5.89119	-0.43782	-2.95544	C	5.31000	-1.17590	-0.09785
C	-6.36687	-1.64679	-1.22790	C	4.36848	-2.29691	-0.42369
H	-7.26563	-2.06084	-1.69181	H	4.66830	-3.25138	0.03892
C	-6.01969	-2.04759	0.06493	H	4.27509	-2.46390	-1.50924
H	-6.63565	-2.76657	0.60999	C	-2.04088	4.61449	-1.92245
C	-4.86957	-1.50464	0.62815	C	-1.39125	3.55349	-1.18932
C	-4.22715	-1.72543	1.96495	N	-0.86732	2.70752	-0.60633
H	-4.88695	-1.44406	2.80213	H	-1.94064	5.55912	-1.36918
H	-3.92344	-2.77450	2.11608	H	-3.10699	4.37949	-2.05021
Cl	1.31767	3.04038	1.44988	H	-1.56939	4.71939	-2.90993
Cl	-0.39959	0.38365	3.13997				

NMR Characterization of [AnCl$_4$(H-phthal)$_2$] complexes

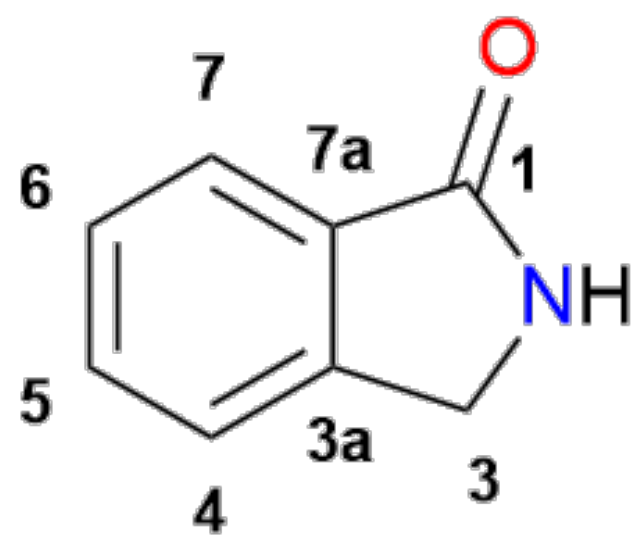

Figure S29. Structure of phthalimidine (H-phthal) indicating the numbering scheme used for NMR characterization.

[UCl$_4$(H-phthal)$_2$]·2THF

^{1}H-NMR (CD$_3$CN, 65 °C): δ = 10.46 (br s, 2H, NH), 8.20 (br, 2H, H7), 7.87 (t, $^3J_{HH}$ = 7.23, 7.39, 2H, H5), 7.70 (d, $^3J_{HH}$ = 7.35 Hz, 2H, H4), 7.59 (t, $^3J_{HH}$ = 6.79 Hz, 6.95, 2H, H6), 3.06 (s, 4H, H3) ppm.

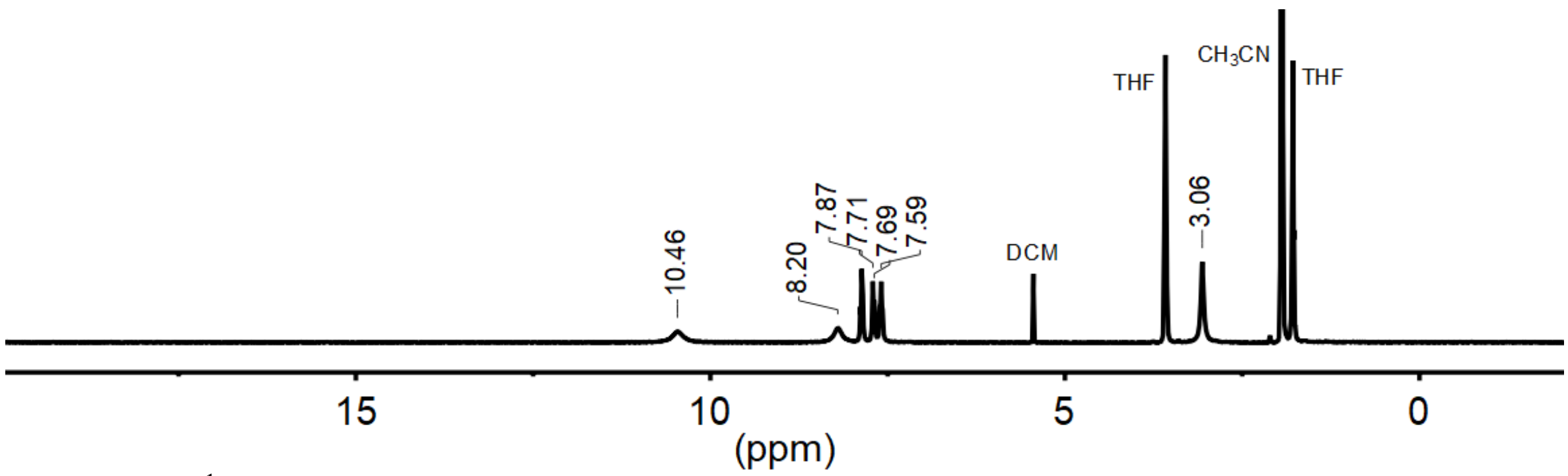

Figure S30. ^{1}H NMR spectrum of [UCl$_4$(H-phthal)$_2$]·2THF in CD$_3$CN at 65 °C.

^{13}C (CD$_3$CN, 65 °C): δ = 194.9 (C1), 145.4 (C3a), 135.0 (C7a), 134.4 (C5), 130.3 (C6), 125.8 (C4), 124.7 (C7), 53.1 (C3) ppm.

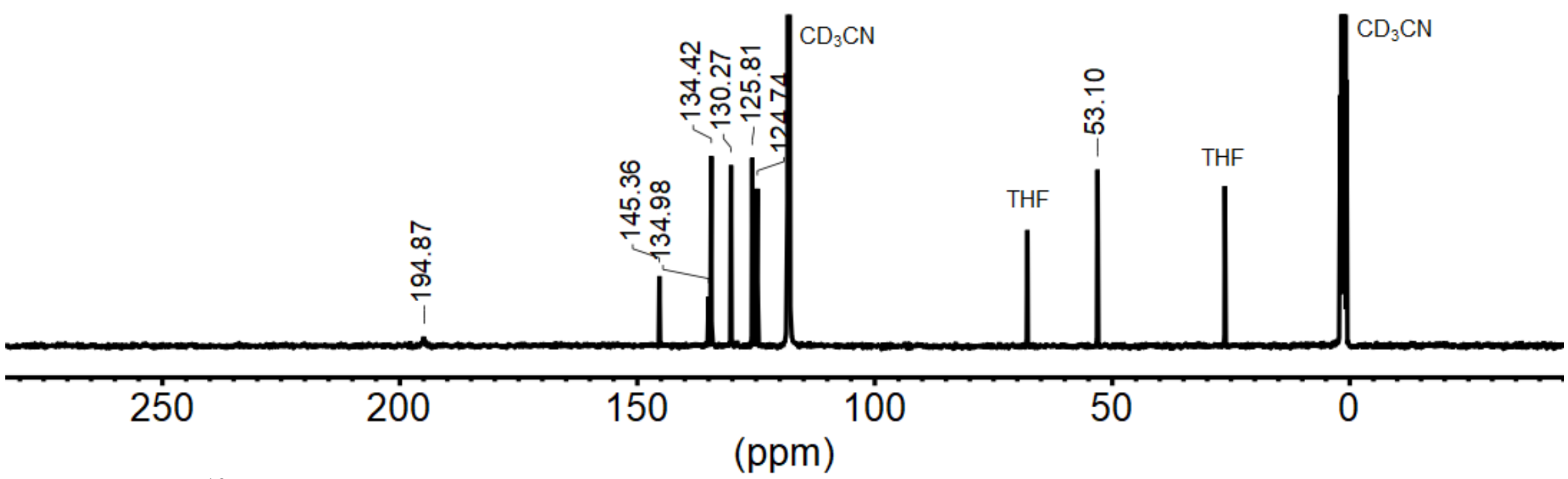

Figure S31. ^{13}C NMR spectrum of [UCl$_4$(H-phthal)$_2$]·2THF in CD$_3$CN at 65 °C.

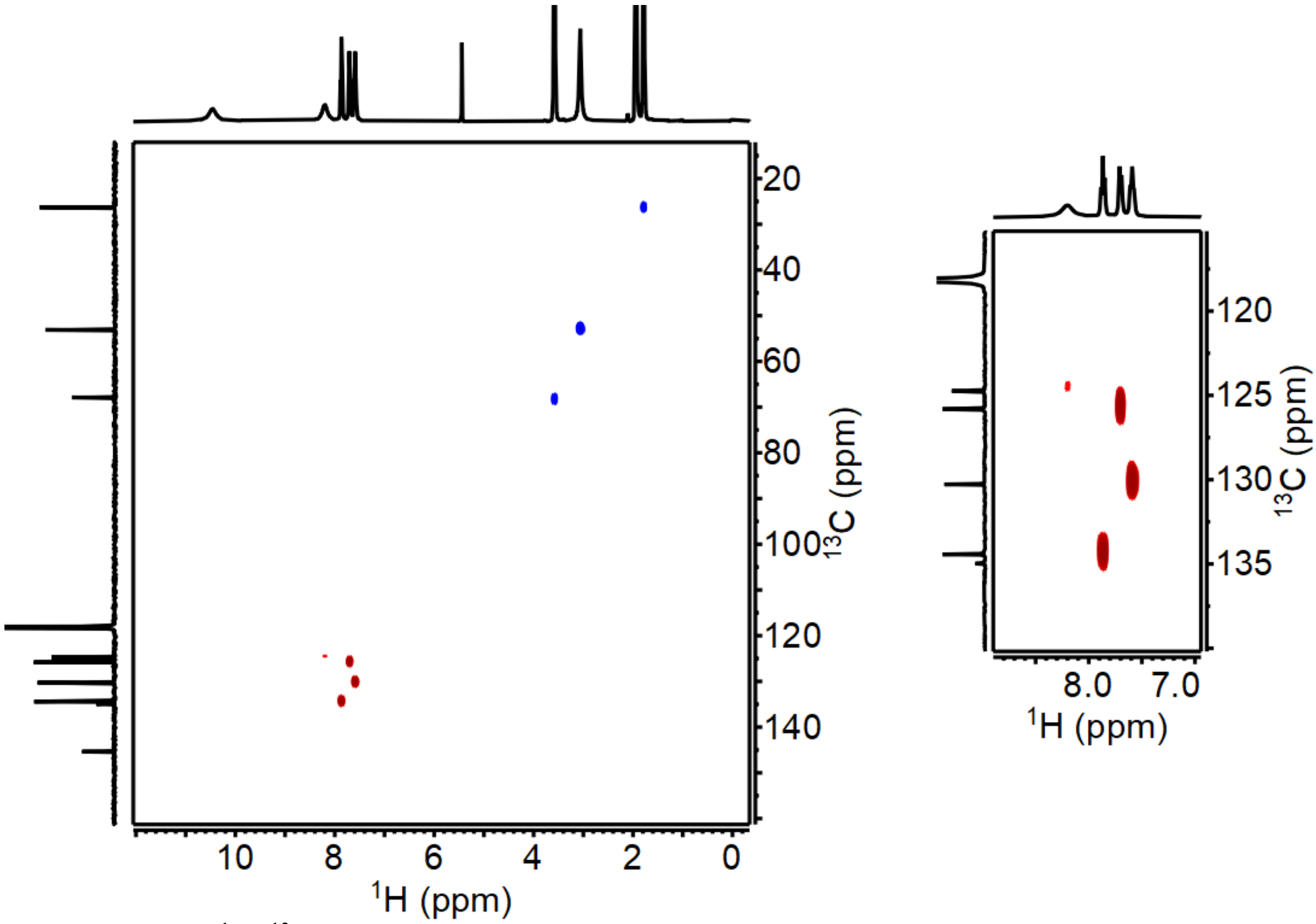

Figure S32. 2D ^{1}H, ^{13}C-HSQC of $[UCl_4(H\text{-}phthal)_2]\cdot 2THF$ in CD_3CN at 65 °C.

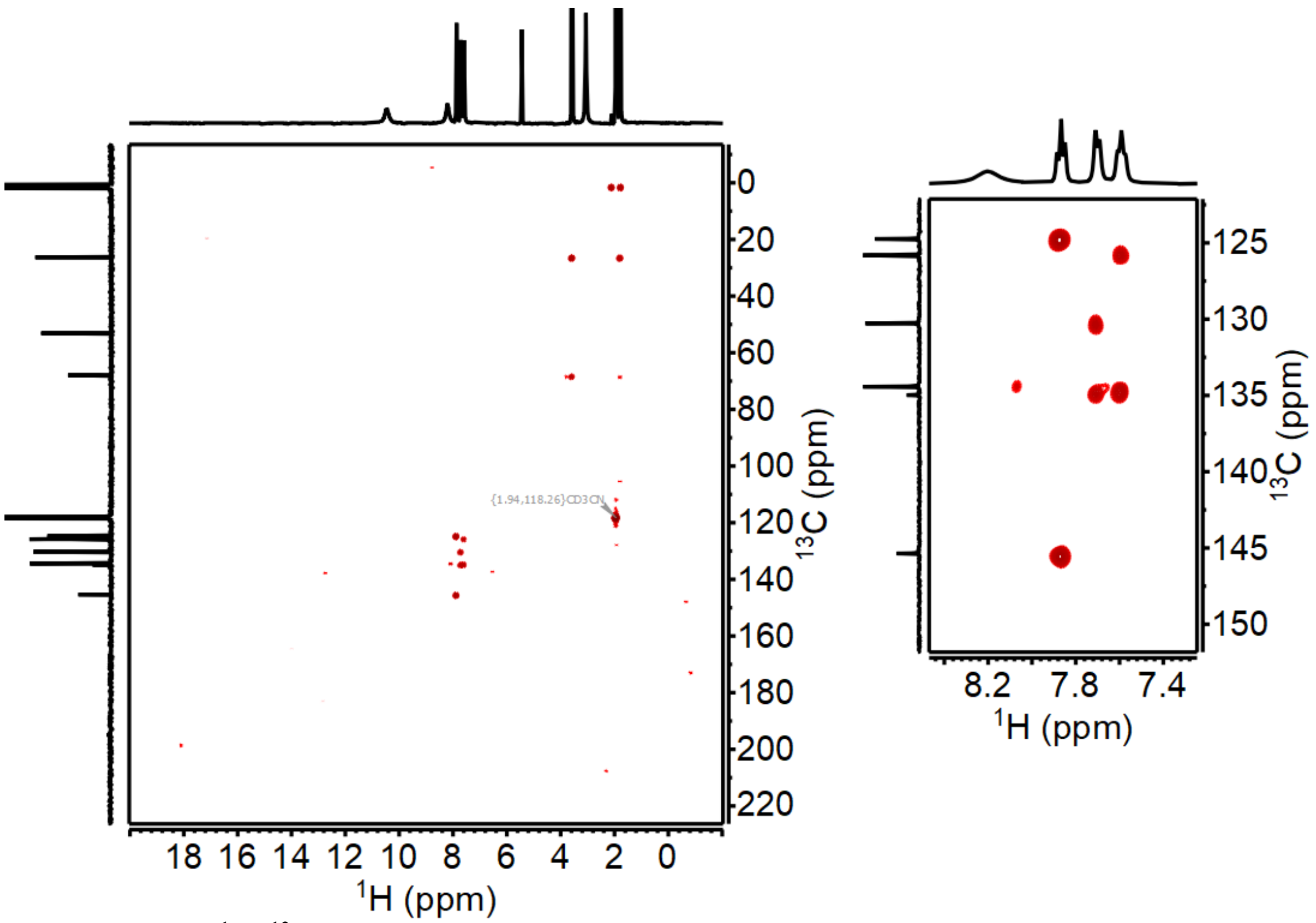

Figure S33. 2D ^{1}H, ^{13}C-HMBC of $[UCl_4(H\text{-}phthal)_2]\cdot 2THF$ in CD_3CN at 65 °C.

[UCl$_4$(H-phthal)$_2$]

^{1}H-NMR (CD$_3$CN, 65 °C): δ = 10.40 (br s, 2H, NH), 8.16 (br, 2H, H7), 7.87 (t, $^3J_{HH}$ = 7.23, 7.39, 2H, H5), 7.71 (d, $^3J_{HH}$ = 7.35 Hz, 2H, H4), 7.59 (t, $^3J_{HH}$ = 6.79 Hz, 6.95, 2H, H6), 3.10 (s, 4H, H3) ppm.

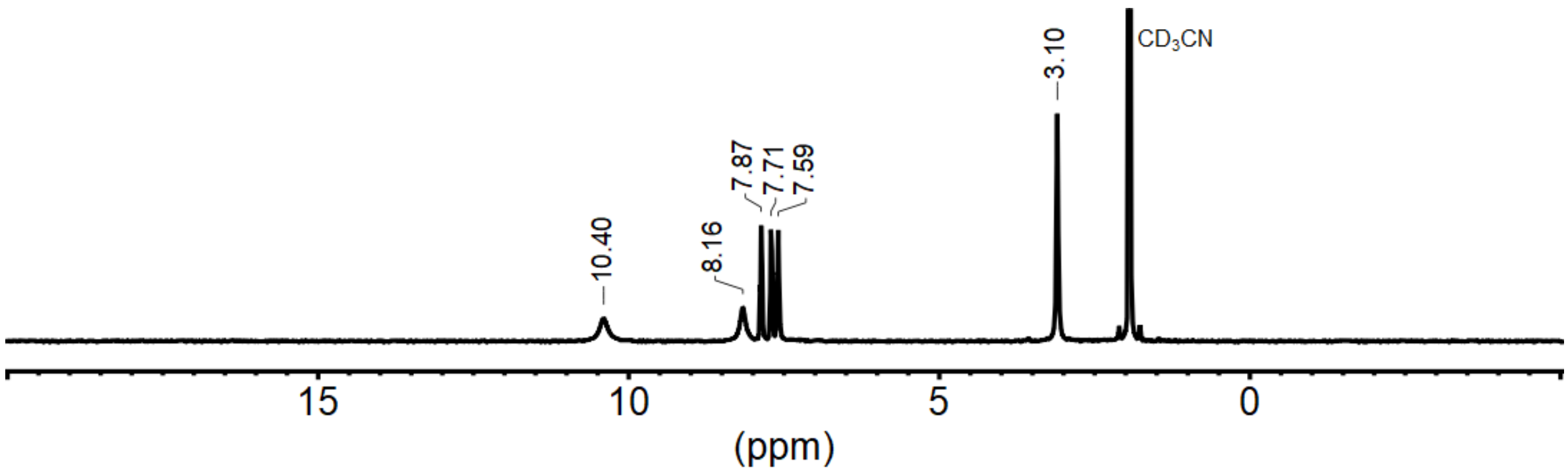

Figure S34. ^{1}H NMR spectrum of [UCl$_4$(H-phthal)$_2$] in CD$_3$CN at 65 °C.

^{13}C (CD$_3$CN, 65 °C): δ = 195.2 (C1), 145.4 (C3a), 134.7 (C7a), 134.5 (C5), 130.3 (C6), 125.8 (C4), 124.7 (C7), 52.9 (C3) ppm.

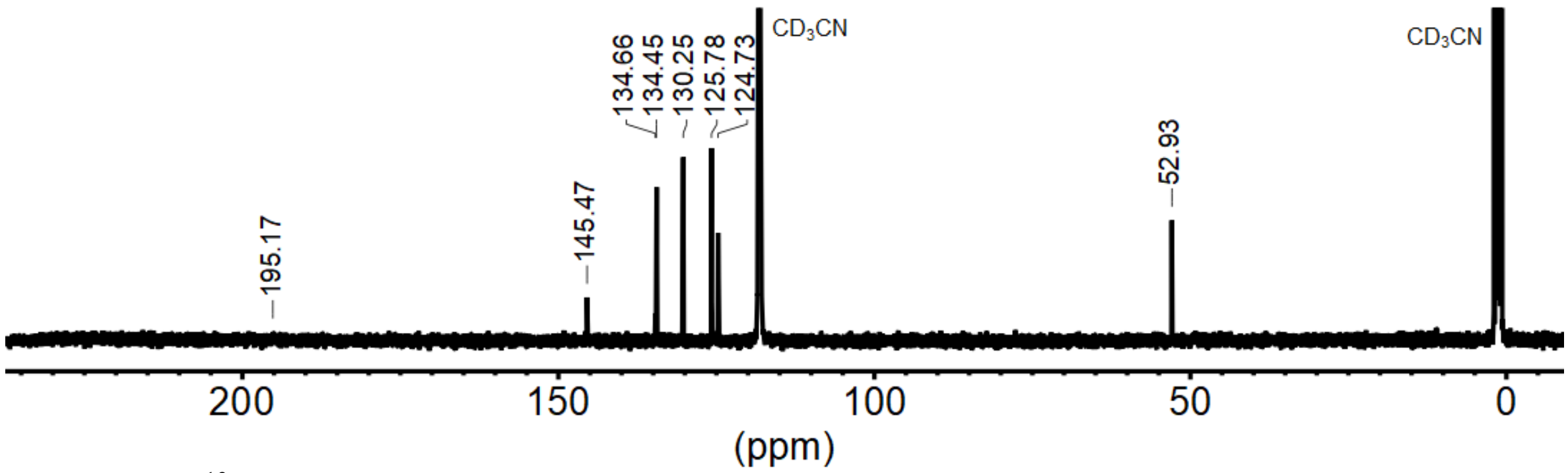

Figure S35. ^{13}C NMR spectrum of [UCl$_4$(H-phthal)$_2$] in CD$_3$CN at 65 °C.

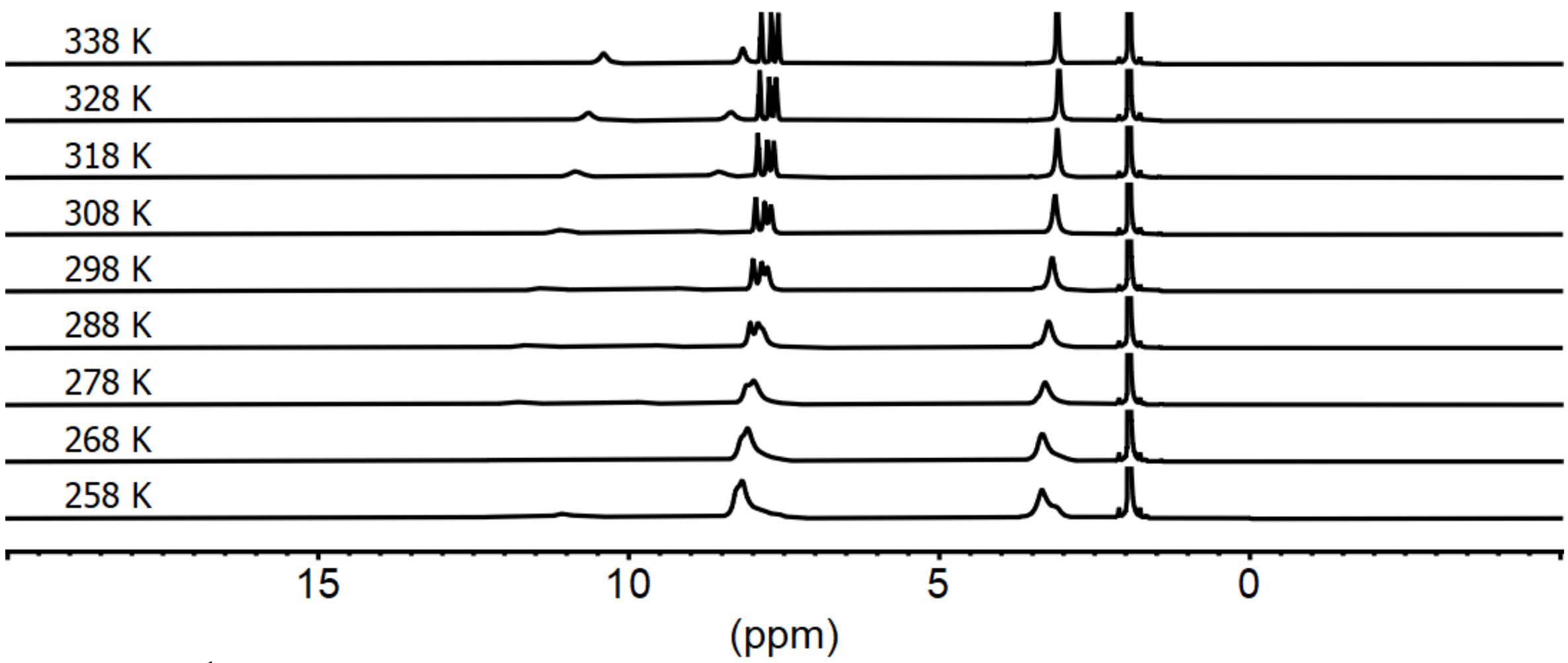

Figure S36. ^{1}H NMR temperature series of [UCl$_4$(H-phthal)$_2$] in CD$_3$CN from 258 K to 338 K (−15 to 65 °C).

Table S25. NMR chemical shifts of [UCl_4(H-phthal)$_2$] in CD_3CN at various temperatures.

**Temperature (K)**	**^{1}H shift (ppm)**					
	NH	CH-7	CH-5	CH-4	CH-6	CH_2-3
**258**						3.35
**268**	11.88	10.19	8.18	8.09		3.34
**278**	11.77	9.83	8.1	7.99		3.3
**288**	11.55	9.48	8.05	7.92	7.85	3.24
**298**	11.3	9.13	8	7.86	7.77	3.19
**308**	11.07	8.8	7.96	7.81	7.71	3.14
**318**	10.85	8.54	7.92	7.77	7.66	3.1
**328**	10.65	8.36	7.89	7.73	7.63	3.08
**338**	10.41	8.17	7.87	7.7	7.59	3.1

[$NpCl_4$(H-phthal)$_2$]

^{1}H-NMR (CD_3CN, 65 °C): δ = 7.92 (br s, 2H, NH), 7.03 (br t, 2H, H5), 6.78 (br t, 2H, H6), 6.51 (br d, 2H, H4), 5.08 (br, 2H, H7), −0.10 (s, 4H, H3) ppm.

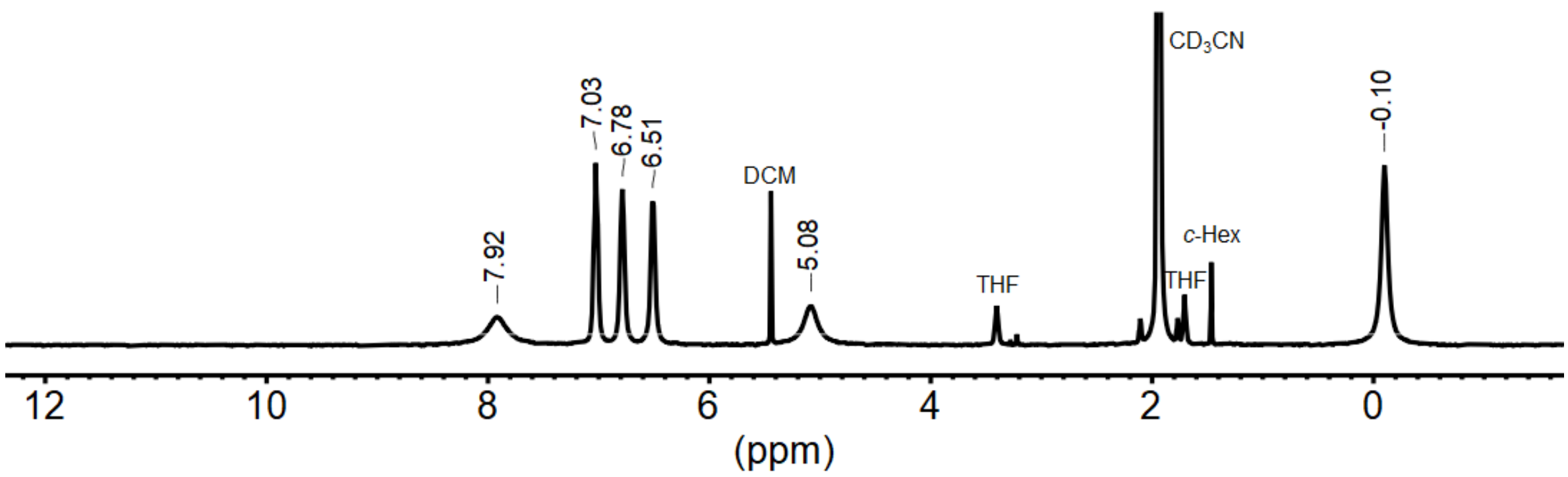

Figure S37. ^{1}H NMR spectrum of [$NpCl_4$(H-phthal)$_2$] in CD_3CN at 65 °C.

^{13}C (CD_3CN, 65 °C): δ = 144.4 (C1), 140.7 (C3a), 132.9 (C5), 128.9 (C7a), 128.7 (C6), 124.0 (C4), 121.3 (C7), 51.6 (C3) ppm.

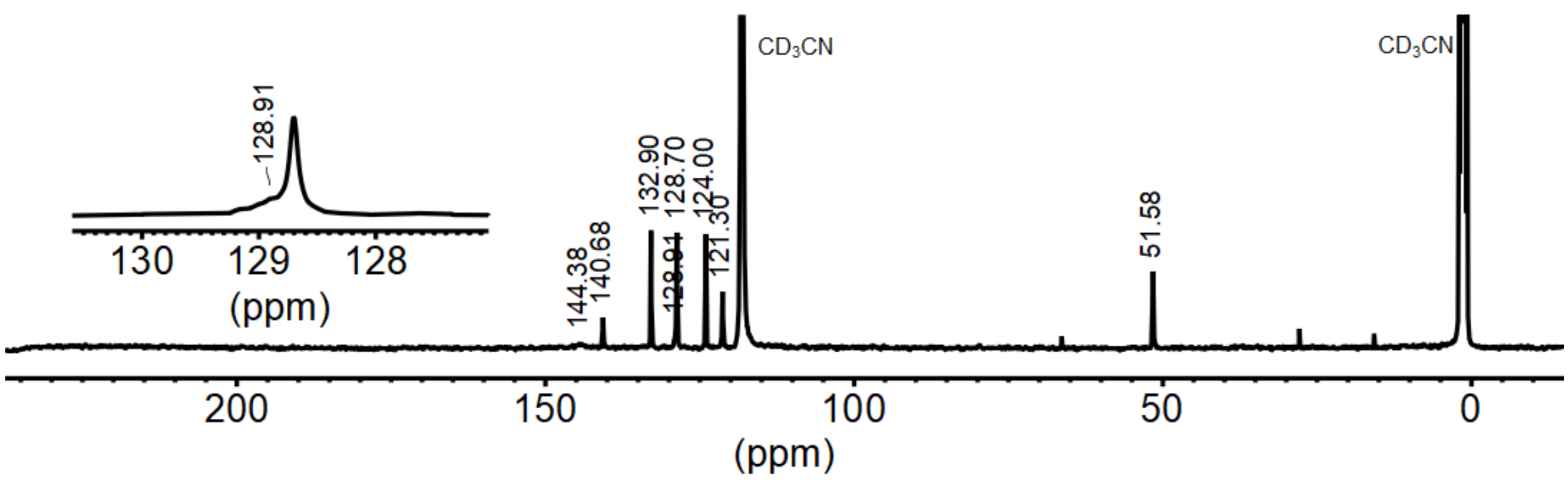

Figure S38. ^{13}C NMR spectrum of [$NpCl_4$(H-phthal)$_2$] in CD_3CN at 65 °C.

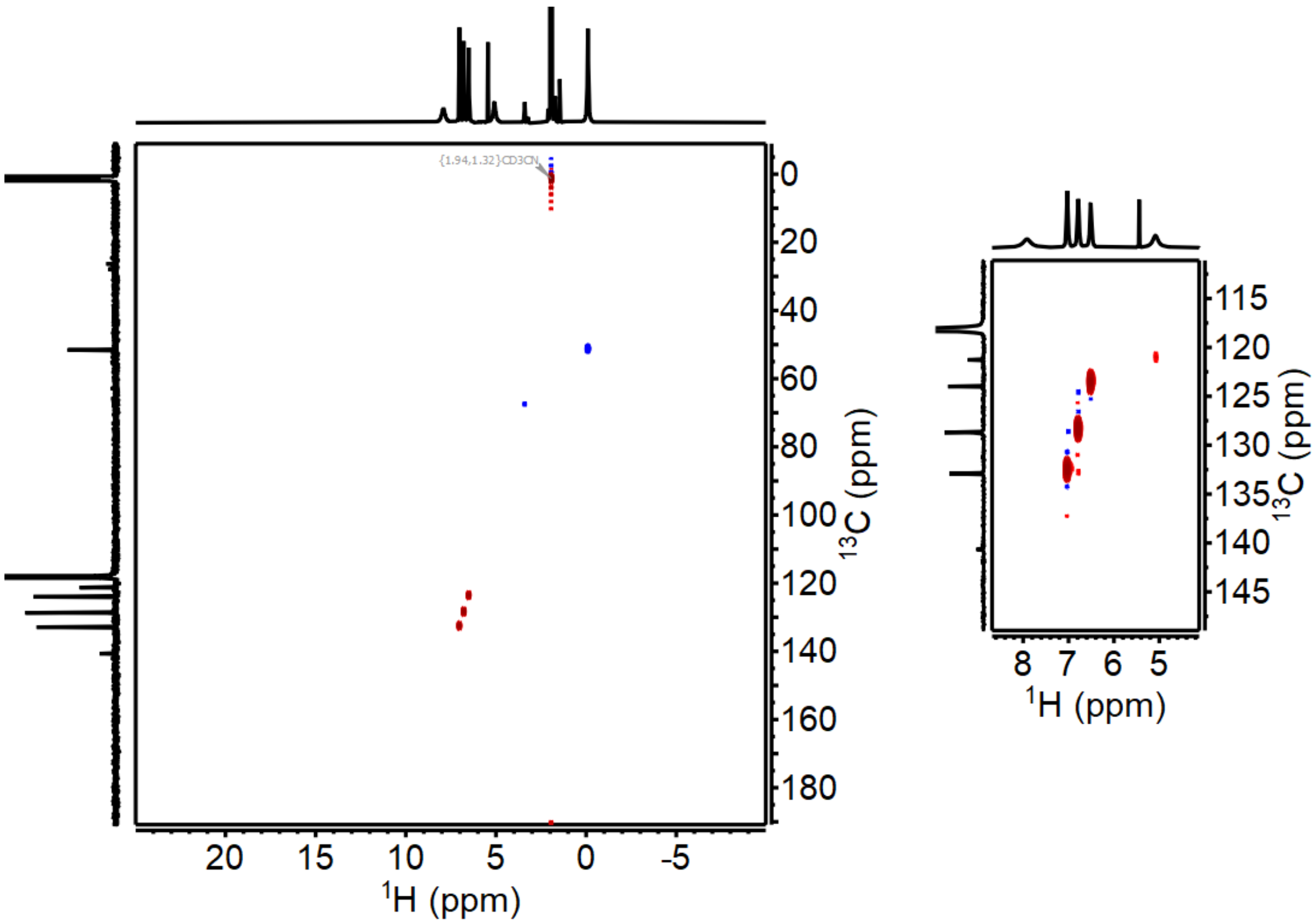

Figure S39. 2D ^{1}H, ^{13}C-HSQC of [$NpCl_4$(H-phthal)$_2$] in CD_3CN at 65 °C.

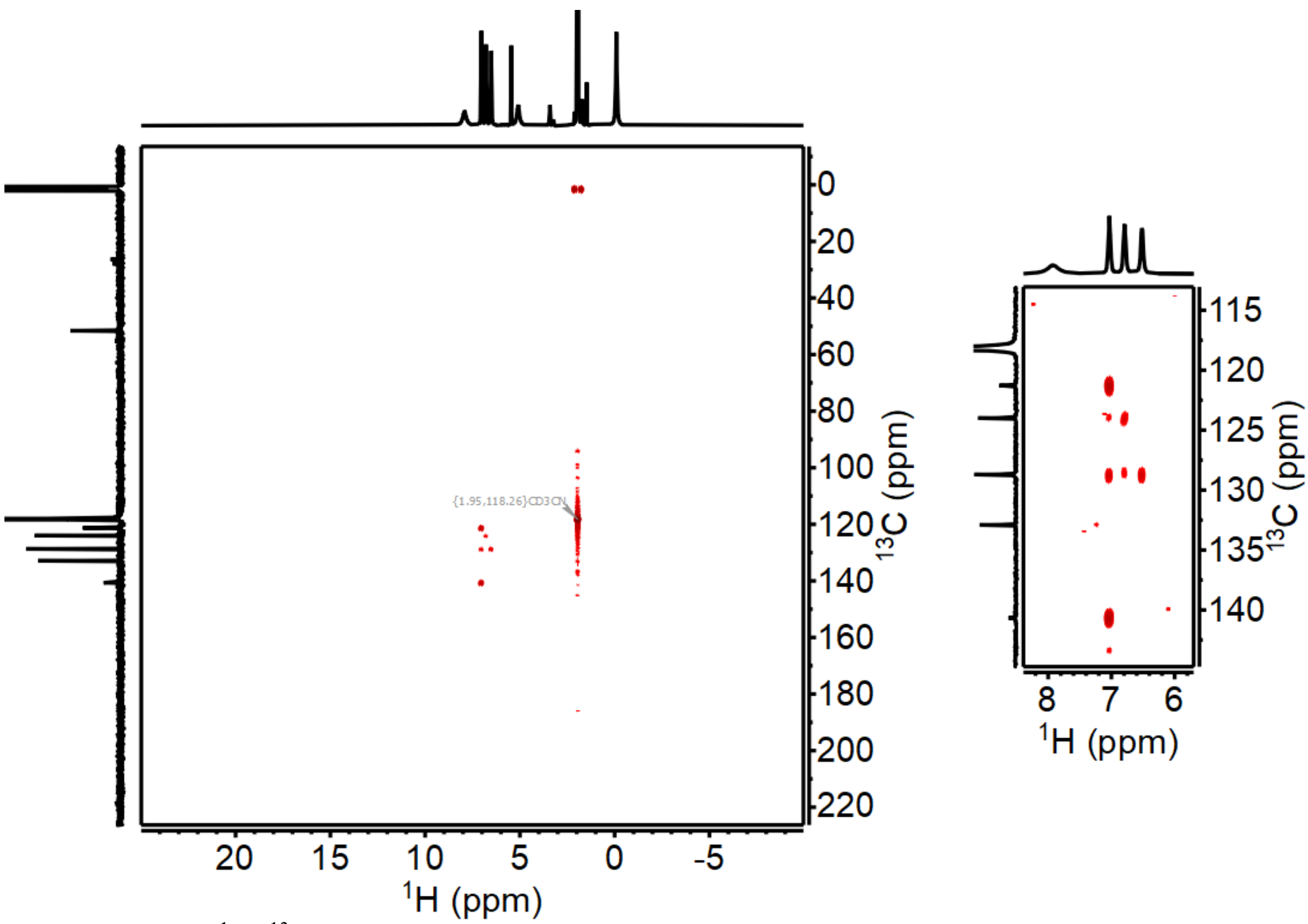

Figure S40. 2D ^{1}H, ^{13}C-HMBC of [$NpCl_4$(H-phthal)$_2$] in CD_3CN at 65 °C.

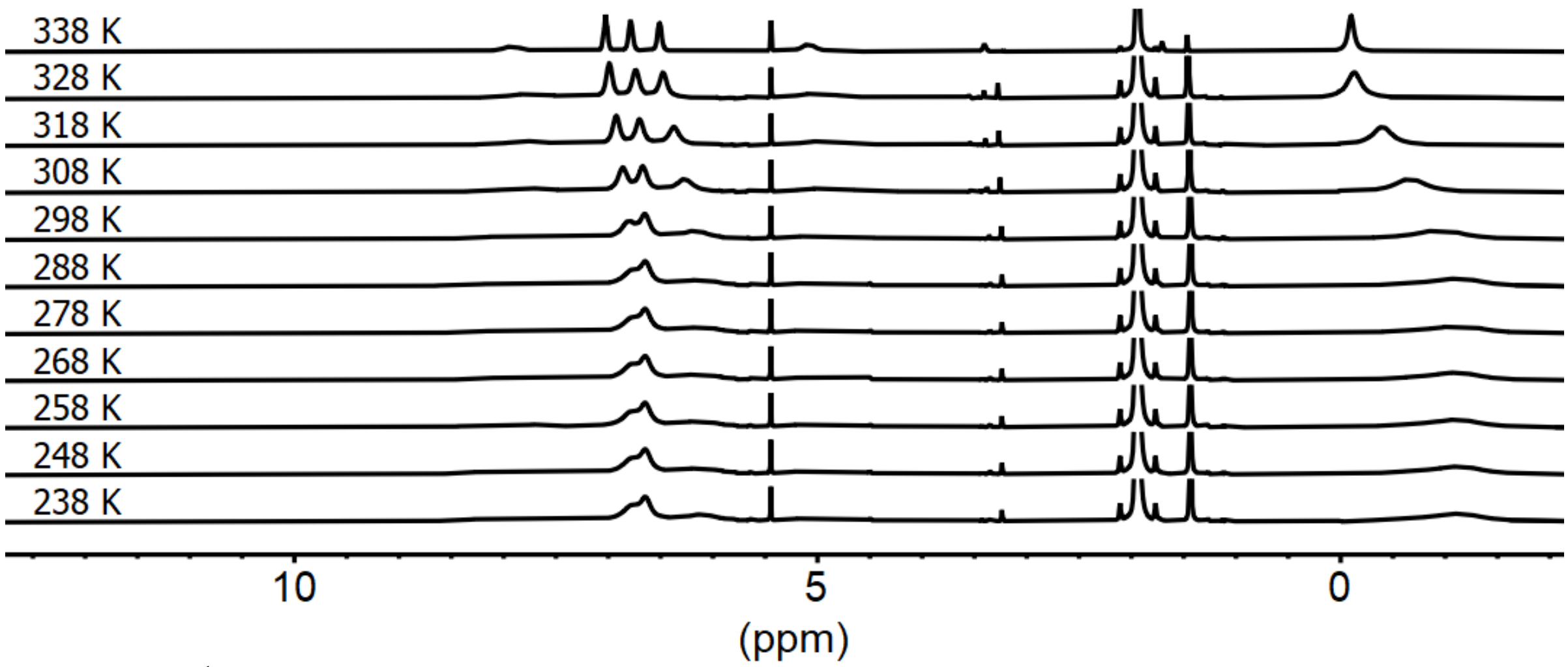

Figure S41. ^{1}H NMR temperature series of $[NpCl_4(H\text{-}phthal)_2]$ in CD_3CN from 238 K to 338 K (−35 to 65 °C).

Table S26. NMR chemical shifts of $[NpCl_4(H\text{-}phthal)_2]$ in CD_3CN at various temperatures.

**Temperature (K)**	**^{1}H shift (ppm)**					
	**NH**	**CH-5**	**CH-6**	**CH-4**	**CH-7**	**CH_2-3**
**288**	8.09	6.77	6.64	6.10	5.09	-1.14
**298**	8.03	6.80	6.65	6.17	5.02	-0.95
**308**	7.78	6.86	6.67	6.27	4.94	-0.66
**318**	7.73	6.92	6.70	6.37	4.93	-0.40
**328**	7.75	6.99	6.74	6.48	4.98	-0.12
**338**	7.92	7.03	6.79	6.51	5.08	-0.10

$[PuCl_4(H\text{-}phthal)_2]$

^{1}H-NMR (CD_3CN, 65 °C): δ = 8.49 (br s, 2H, NH), 7.61 (dd, $^{3}J_{HH}$ = 7.39, 7.55 Hz, 2H, H5), 7.44 (d, $^{3}J_{HH}$ = 7.23 Hz, 2H, H4), 7.27 (dd, $^{3}J_{HH}$ = 7.15, 7.07 Hz, 2H, H6), 6.78 (br, 2H, H7), 2.80 (s, 4H, H3) ppm.

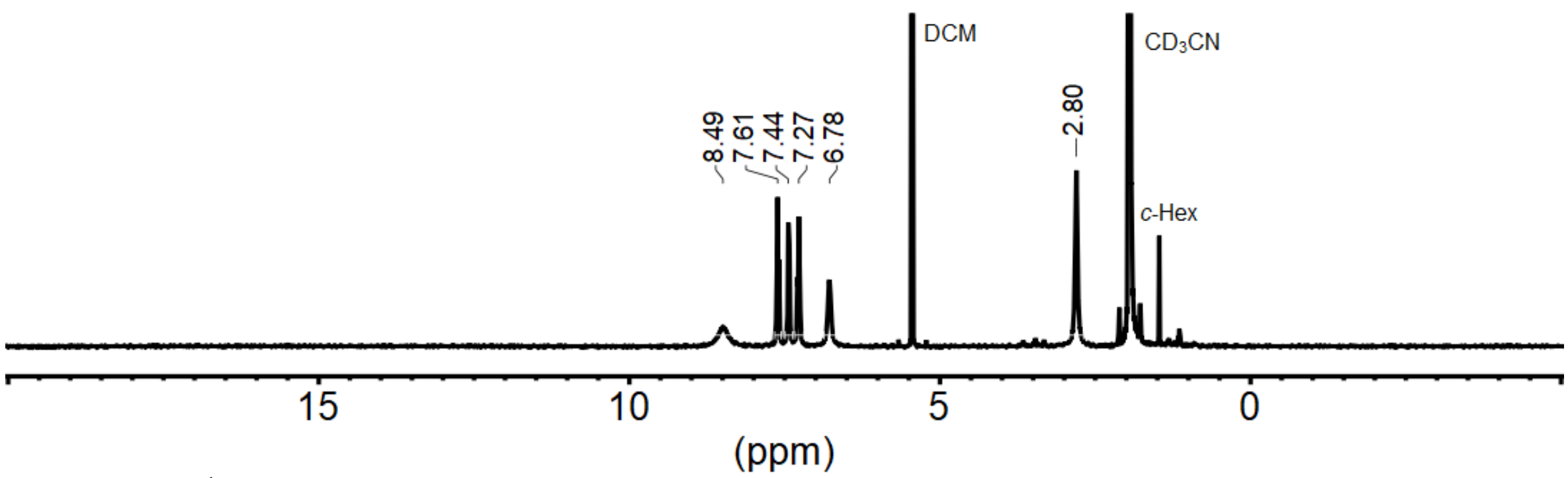

Figure S42. ^{1}H NMR spectrum of $[PuCl_4(H\text{-}phthal)_2]$ in CD_3CN at 65 °C.

^{13}C (CD_3CN, 65 °C): 143.8 (C3a), 134.1 (C5), 130.0 (C7a), 129.2 (C6), 124.5 (C4), 124.1 (C7), 51.1 (C3) ppm.

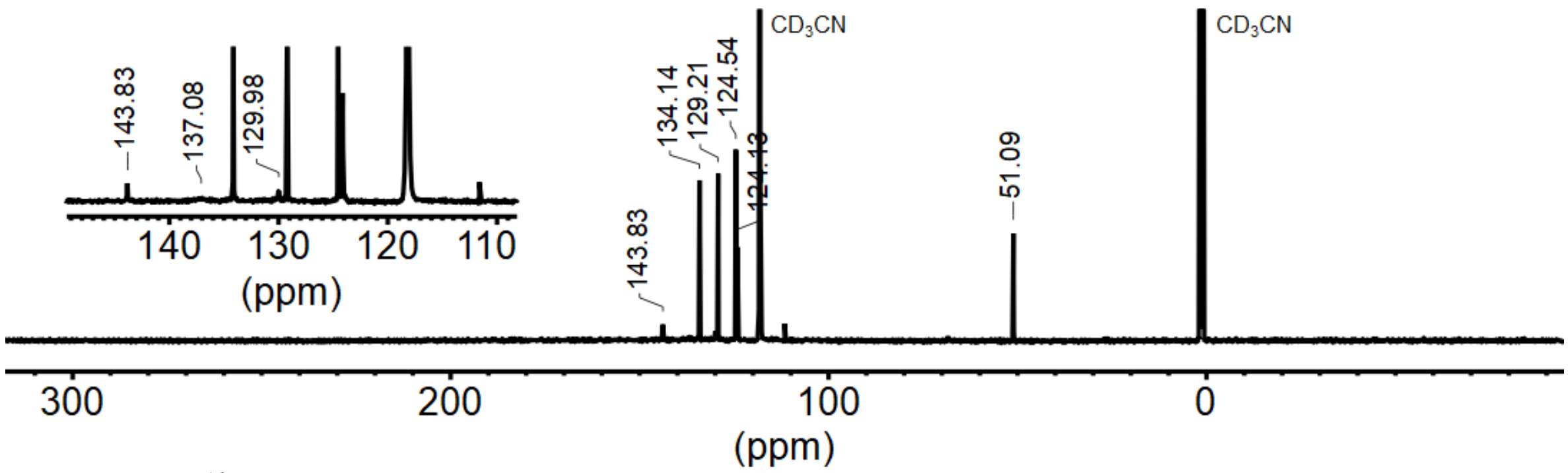

Figure S43. ^{13}C NMR spectrum of $[PuCl_4(H\text{-}phthal)_2]$ in CD_3CN at 65 °C.

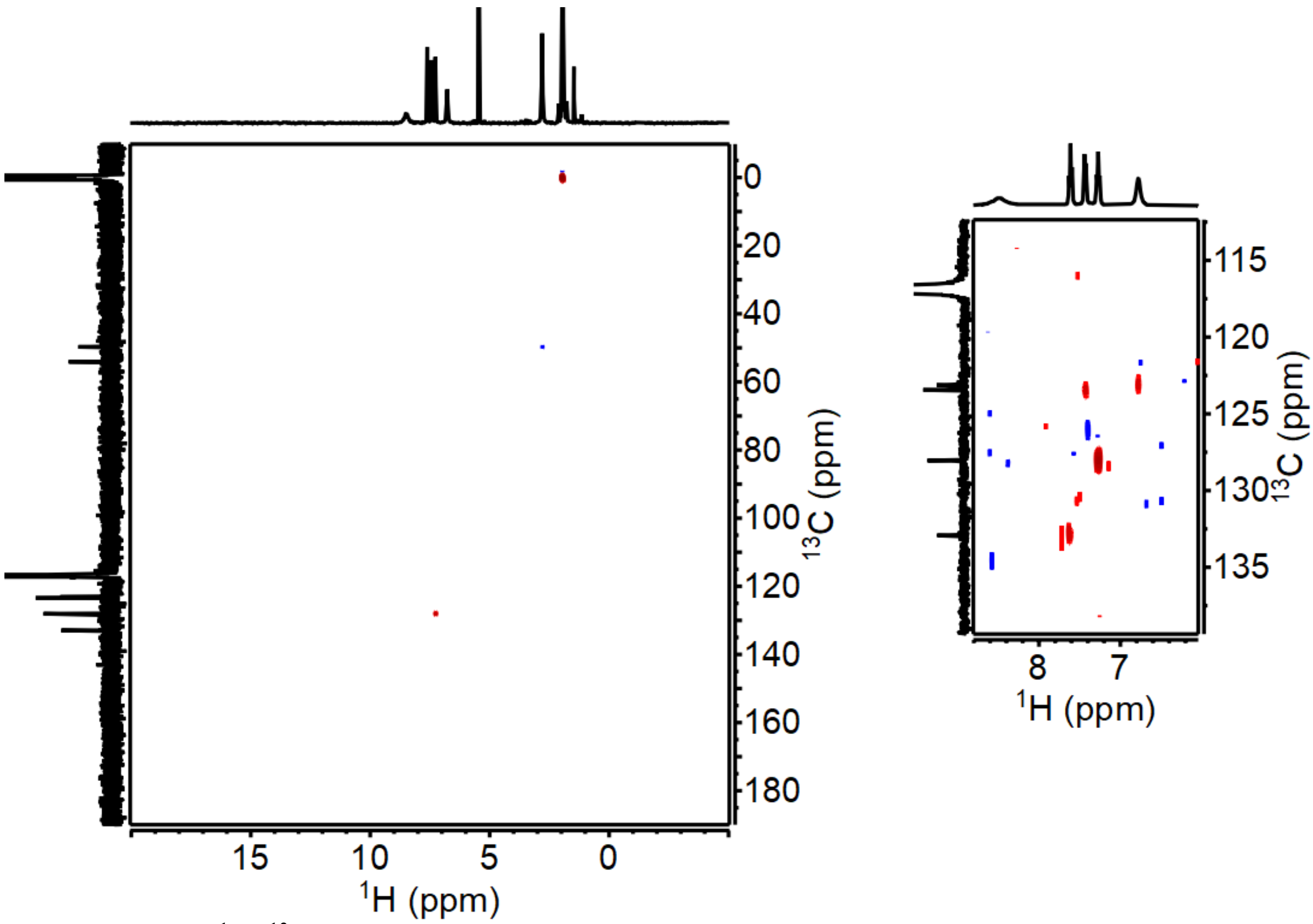

Figure S44. 2D ^1H, ^{13}C-HSQC of $[PuCl_4(H\text{-}phthal)_2]$ in CD_3CN at 65 °C.

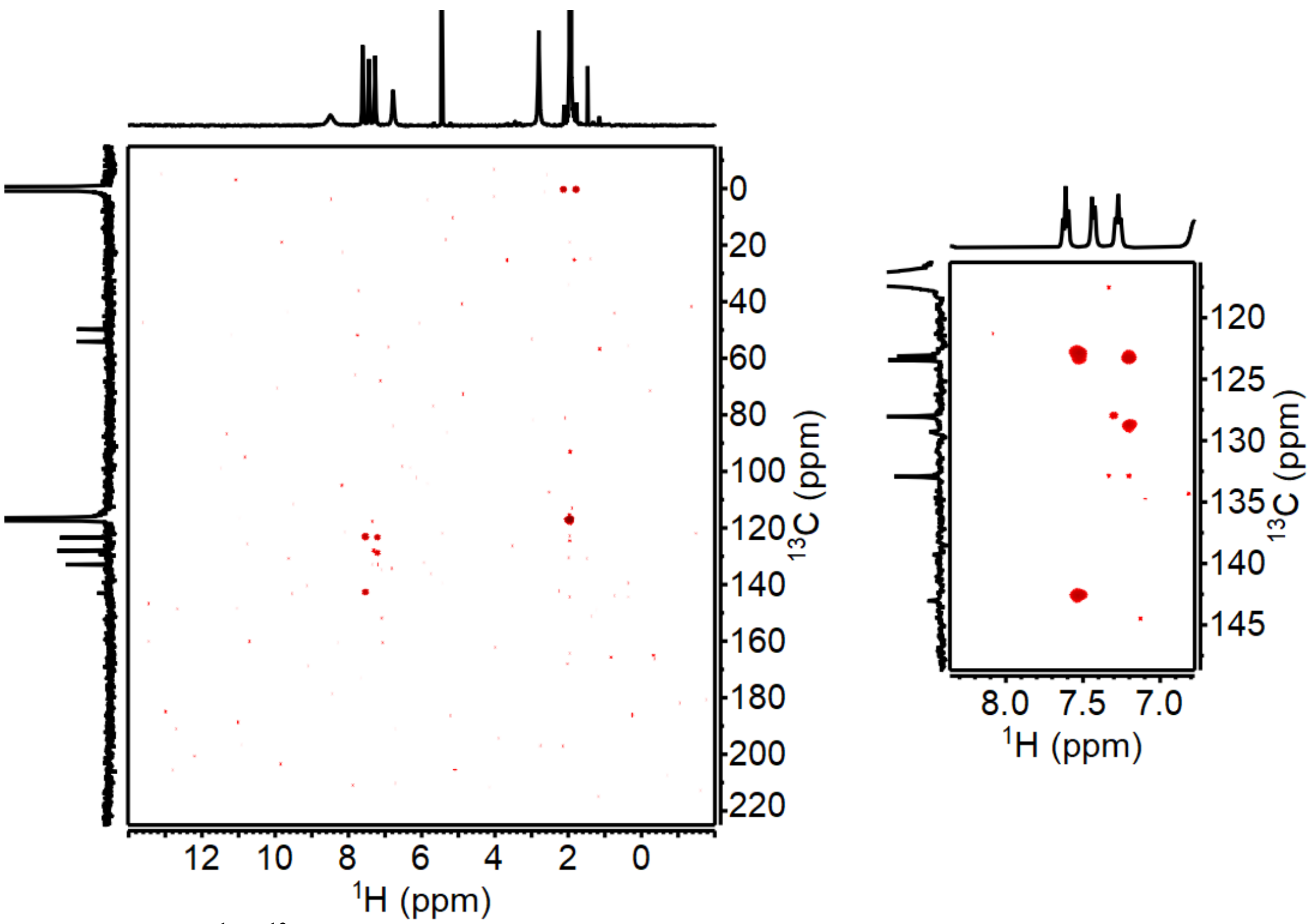

Figure S45. 2D ^{1}H, ^{13}C-HMBC of [$PuCl_4$(H-phthal)$_2$] in CD_3CN at 65 °C.

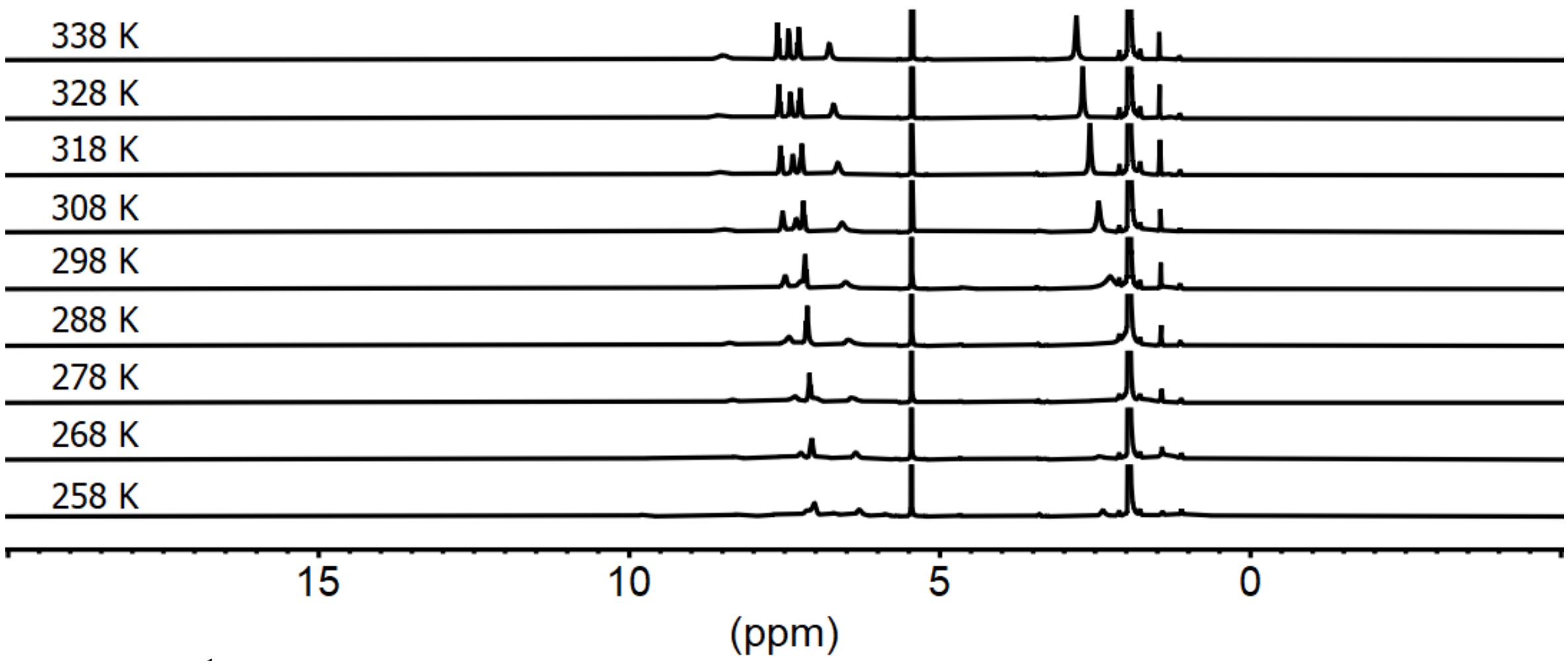

Figure S46. ^{1}H NMR temperature series of [$PuCl_4$(H-phthal)$_2$] in CD_3CN from 258 K to 338 K (−15 to 65 °C).

Table S27. NMR chemical shifts of [$PuCl_4$(H-phthal)$_2$] in CD_3CN at various temperatures.

**Temperature (K)**	**^{1}H shift (ppm)**					
	**NH**	**CH-5**	**CH-4**	**CH-6**	**CH-7**	**CH_2-3**
**288**	8.37	7.42		7.13	6.47	
**298**	8.41	7.48	7.25	7.16	6.51	2.25
**308**	8.47	7.53	7.31	7.19	6.57	2.44
**318**	8.5	7.56	7.36	7.22	6.64	2.58
**328**	8.51	7.59	7.4	7.25	6.71	2.7
**338**	8.48	7.61	7.43	7.27	6.77	2.8

NMR Characterization of [AnCl$_4$(HPyO)$_2$] complexes

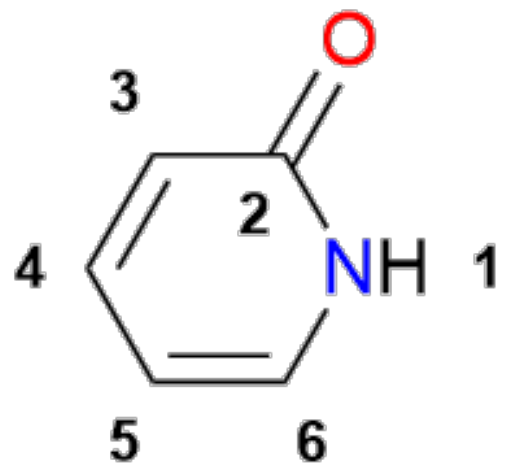

Figure S47. Structure of 2-pyridone (HPyO) indicating the numbering scheme used for NMR characterization.

[UCl$_4$(HPyO)$_2$]·2THF

^{1}H (CD$_3$CN, 65 °C): δ = 17.44 (br s, 2H, NH), 12.71 (br d, 2H, CH), 10.35 (br t, 2H, CH), 9.90 (br d, 2H, CH), 9.00 (br t, 2H, CH) ppm.

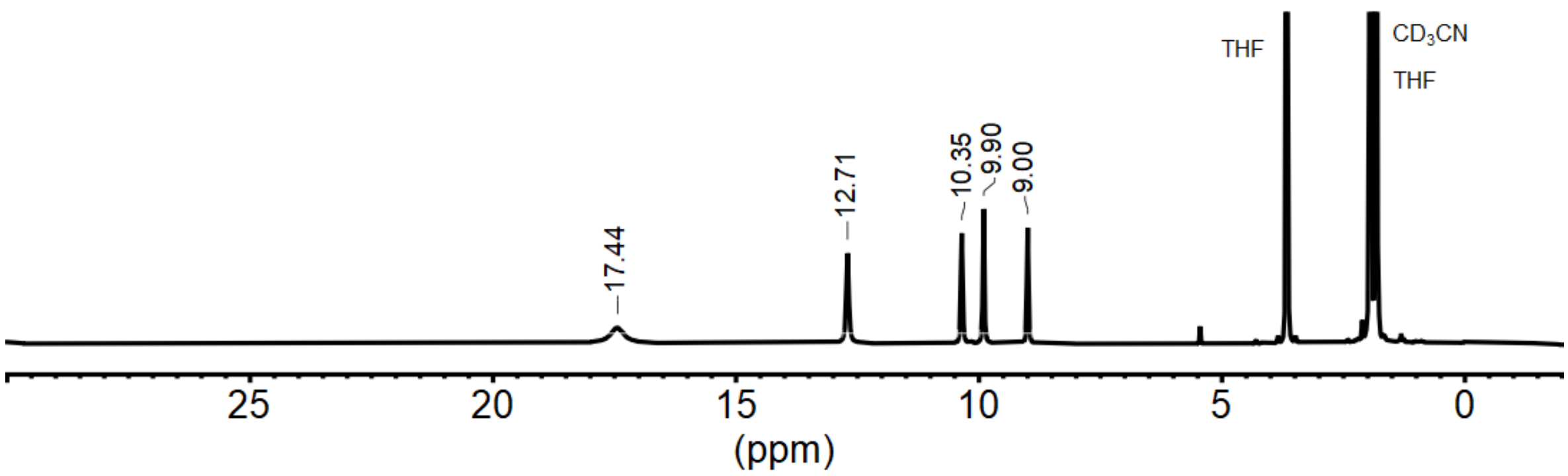

Figure S48. ^{1}H NMR spectrum of [UCl$_4$(HPyO)$_2$]·2THF in CD$_3$CN at 65 °C.

^{13}C (CD$_3$CN, 65 °C): δ = 148.7 (CH), 141.1 (CH), 130.2 (CH), 116.13 (CH) ppm.

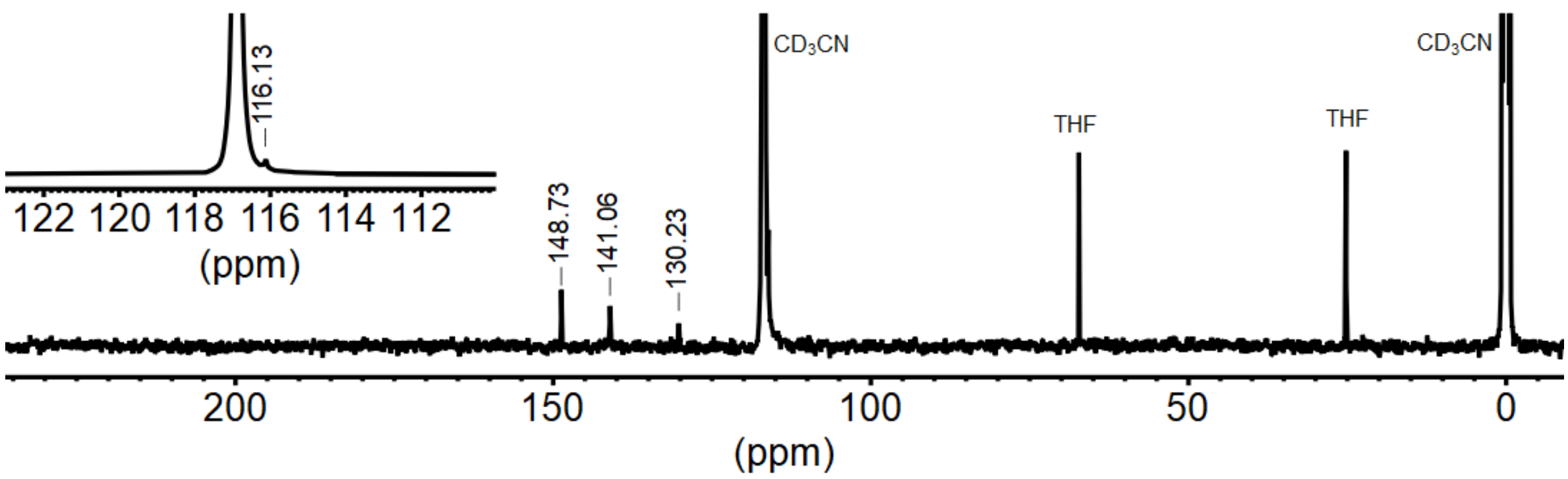

Figure S49. ^{13}C NMR spectrum of [UCl$_4$(HPyO)$_2$]·2THF in CD$_3$CN at 65 °C.

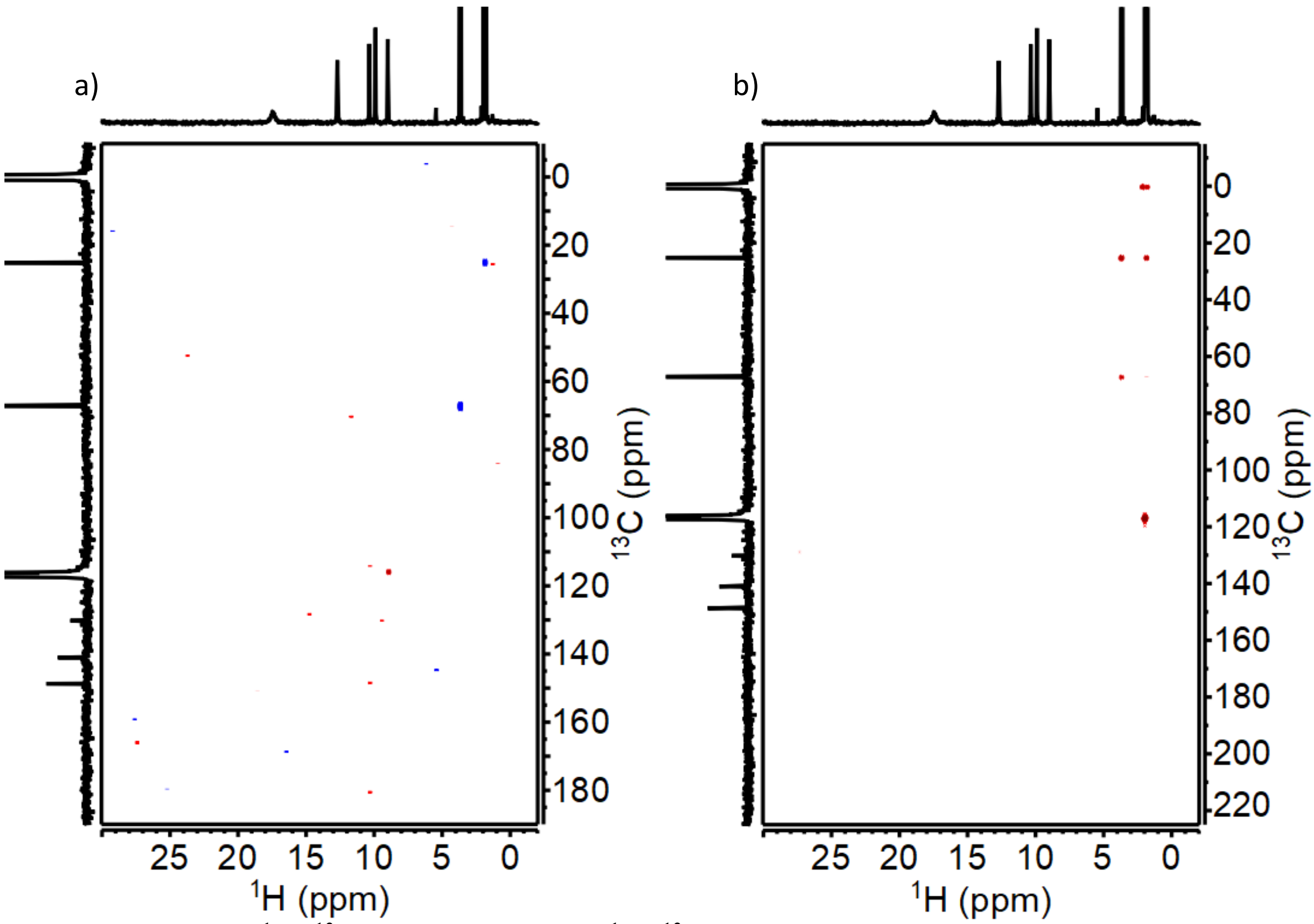

Figure S50. a) 2D ^{1}H, ^{13}C-HSQC and b) 2D ^{1}H, ^{13}C-HMBC spectrum of [$UCl_4(HPyO)_2$]·2THF in CD_3CN at 65 °C.

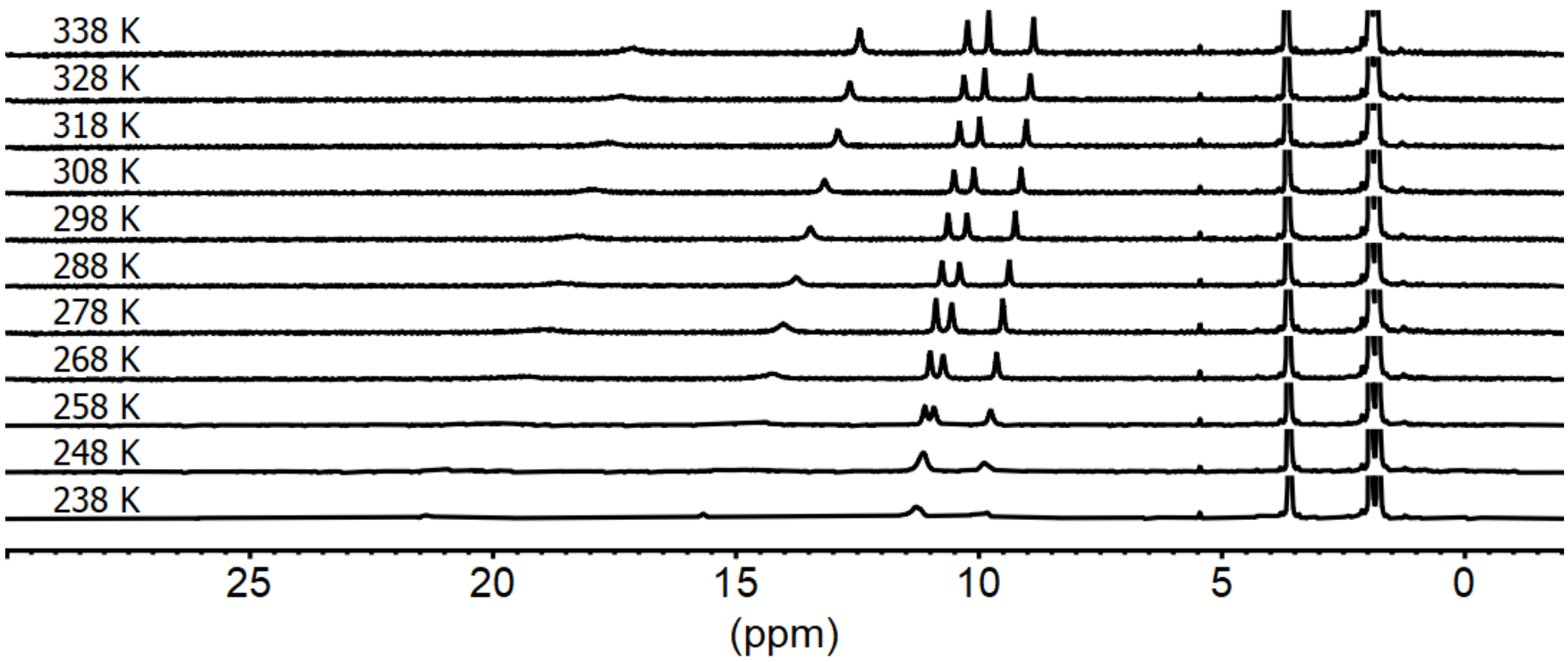

Figure S51. ^{1}H NMR temperature series of [$UCl_4(HPyO)_2$]·2THF in CD_3CN from 238 K to 338 K (−35 to 65 °C).

Table S28. NMR chemical shifts of $[UCl_4(HPyO)_2]\cdot 2THF$ in CD_3CN at various temperatures.

**Temperature (K)**	**^{1}H shift (ppm)**				
	NH	CH	CH	CH	CH
**238**	21.38	15.68	11.30	11.30	10.06
**248**	20.99	15.36	11.14	11.14	9.89
**258**	19.83	14.45	11.11	10.93	9.76
**268**	19.29	14.24	11.01	10.74	9.64
**278**	18.94	14.02	10.89	10.56	9.51
**288**	18.61	13.76	10.77	10.40	9.38
**298**	18.30	13.47	10.64	10.25	9.26
**308**	17.90	13.17	10.51	10.11	9.14
**318**	17.63	12.91	10.40	9.98	9.03
**328**	17.38	12.66	10.31	9.88	8.94
**338**	17.13	12.45	10.24	9.80	8.88

$[NpCl_4(HPyO)_2]$

^{1}H (CD_3CN, 65 °C): 13.12 (br s, 2H, NH), 8.66 (br s, 4H, CH), 8.21 (br s, 2H, CH), 8.02 (br s, 2H, CH) ppm.

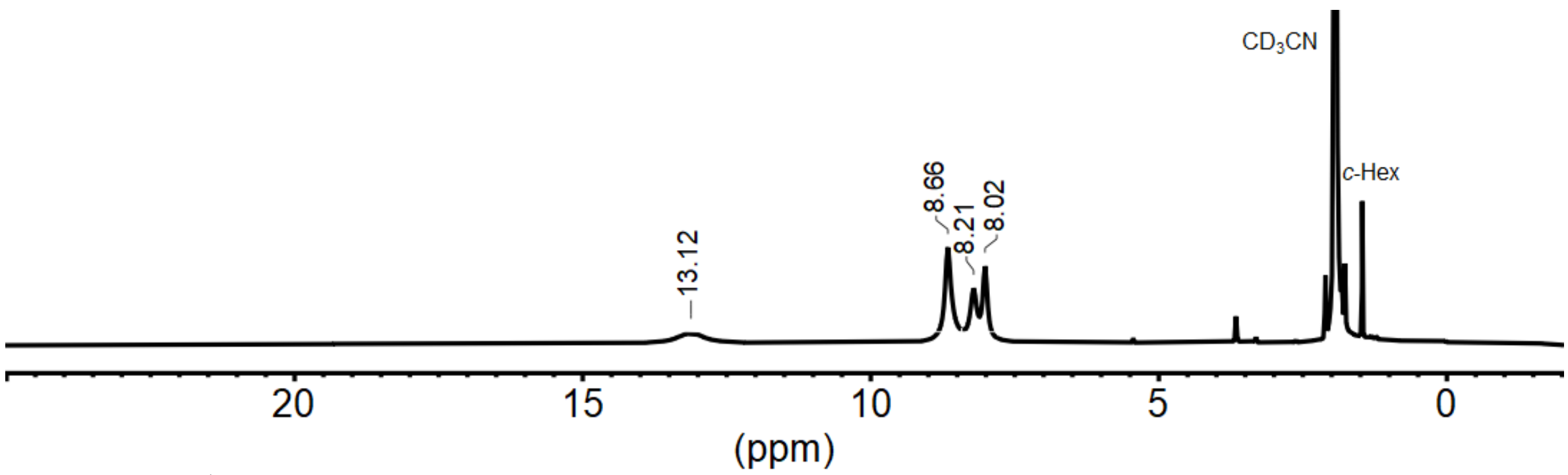

Figure S52. ^{1}H NMR spectrum of $[NpCl_4(HPyO)_2]$ in CD_3CN at 65 °C.

^{13}C (CD_3CN, 65 °C): δ = 145.8 (CH), 140.1 (CH), 122.7 (CH), 114.5 (CH) ppm.

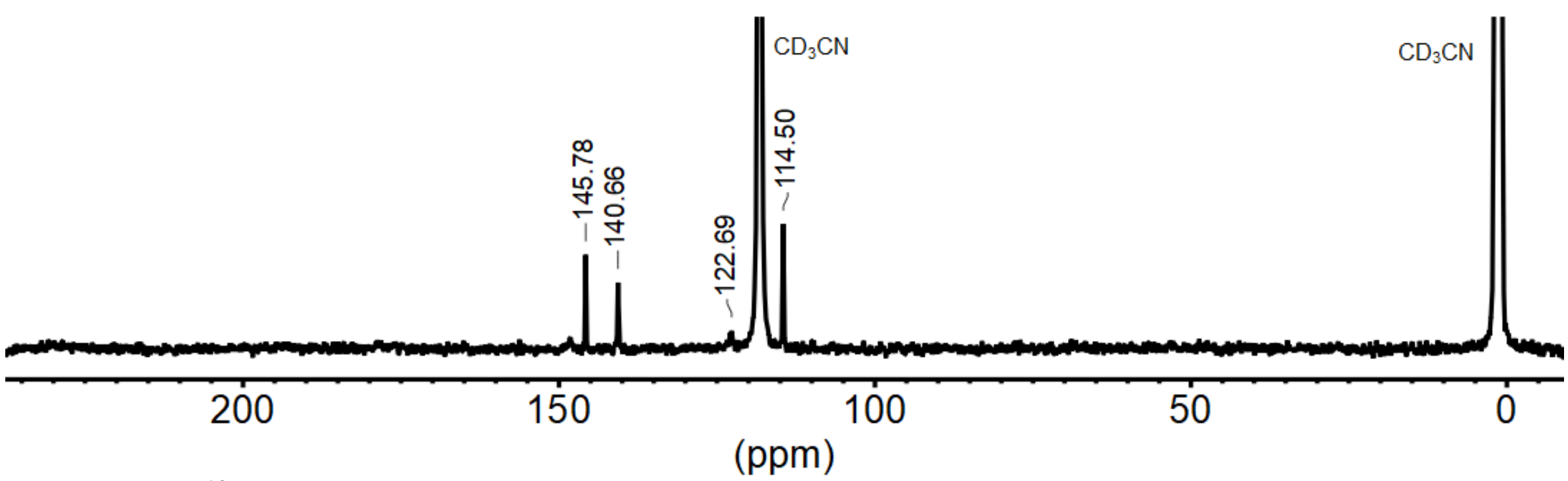

Figure S53. ^{13}C NMR spectrum of $[NpCl_4(HPyO)_2]$ in CD_3CN at 65 °C.

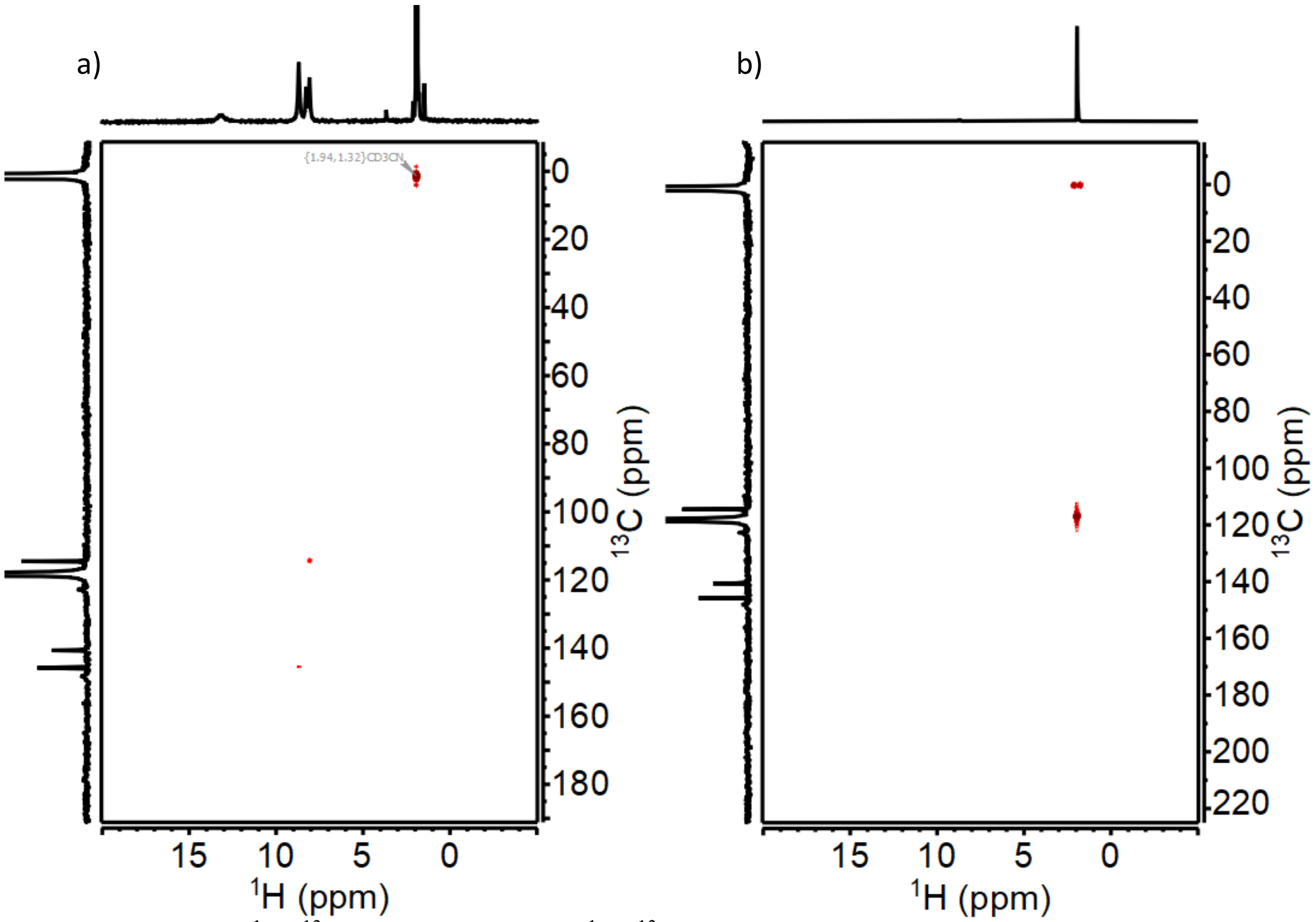

Figure S54. a) 2D ^{1}H, ^{13}C-HSQC and b) 2D ^{1}H, ^{13}C-HMBC spectrum of $[NpCl_4(HPyO)_2]$ in CD_3CN at 65 °C.

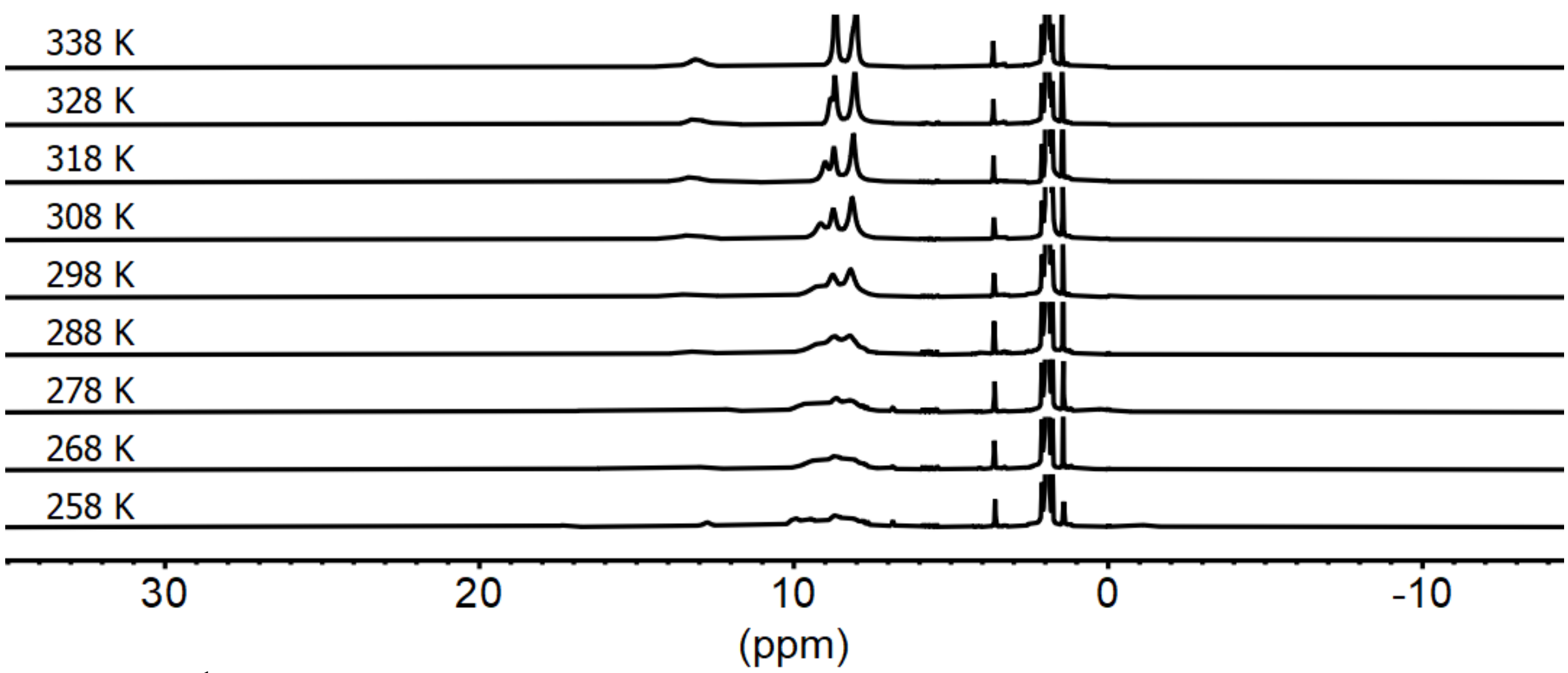

Figure S55. ^{1}H NMR temperature series of $[NpCl_4(HPyO)_2]$ in CD_3CN from 258 K to 338 K (−15 to 65 °C).

Table S29. NMR chemical shifts of [$NpCl_4(HPyO)_2$] in CD_3CN at various temperatures.

**Temperature (K)**	**^{1}H shift (ppm)**				
	NH	CH-5	CH-4	CH-6	CH-7
**288**	13.08	9.22	8.72	8.21	7.82
**298**	13.23	9.21	8.76	8.20	8.20
**308**	13.25	9.14	8.74	8.15	8.15
**318**	13.22	9.01	8.72	8.11	8.11
**328**	13.17	8.82	8.69	8.06	8.06
**338**	13.09	8.67	8.67	8.02	8.02

[$PuCl_4(HPyO)_2$]

^{1}H (CD_3CN, 65 °C): δ = 11.45 (br s, 2H, NH), 7.72 (br t, 2H, CH), 7.55 (br d, 2H, CH), 7.19 (br t, 2H, CH), 6.65 (br d, 2H, CH) ppm.

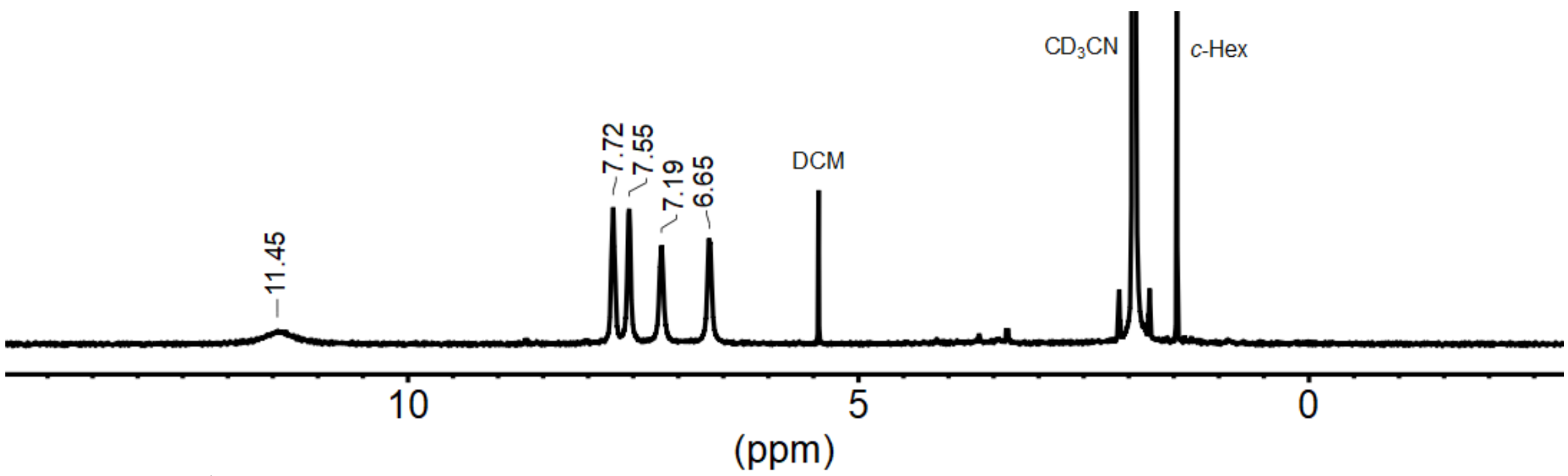

Figure S56. ^{1}H NMR spectrum of [$PuCl_4(HPyO)_2$] in CD_3CN at 65 °C.

^{13}C (CD_3CN, 65 °C): δ = 144.3 (CH), 138.7 (CH), 122.7 (CH), 112.6 (CH) ppm

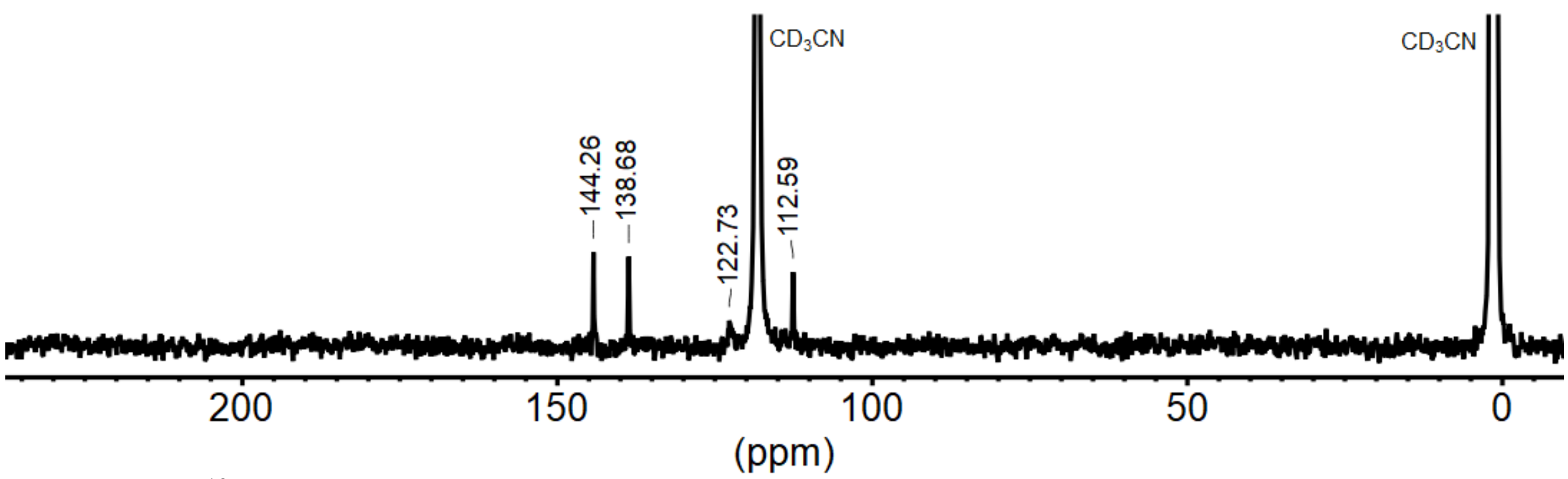

Figure S57. ^{13}C NMR spectrum of [$PuCl_4(HPyO)_2$] in CD_3CN at 65 °C.

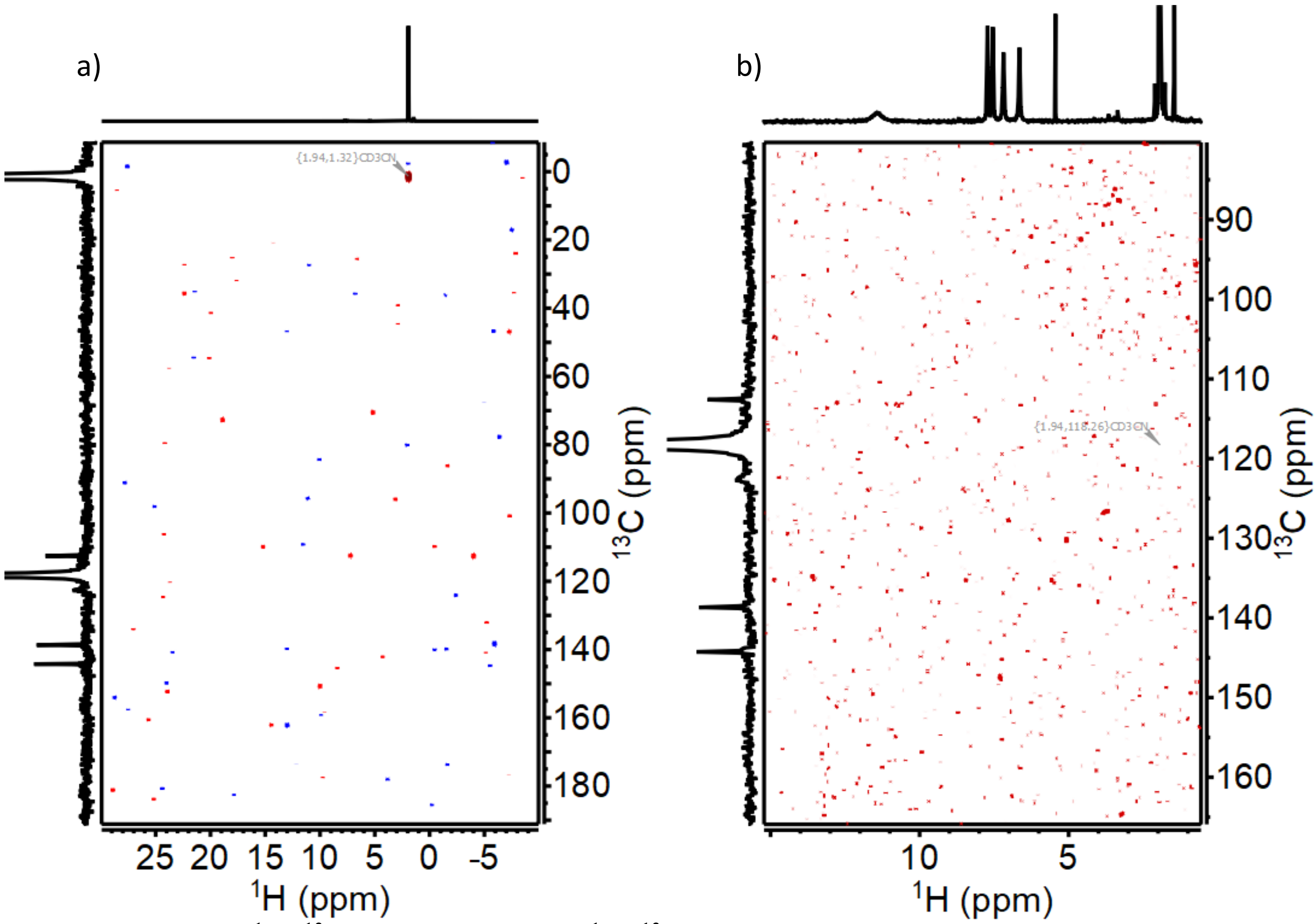

Figure S58. a) 2D ^{1}H, ^{13}C-HSQC and b) 2D ^{1}H, ^{13}C-HMBC spectrum of [$PuCl_4(HPyO)_2$] in CD_3CN at 65 °C.

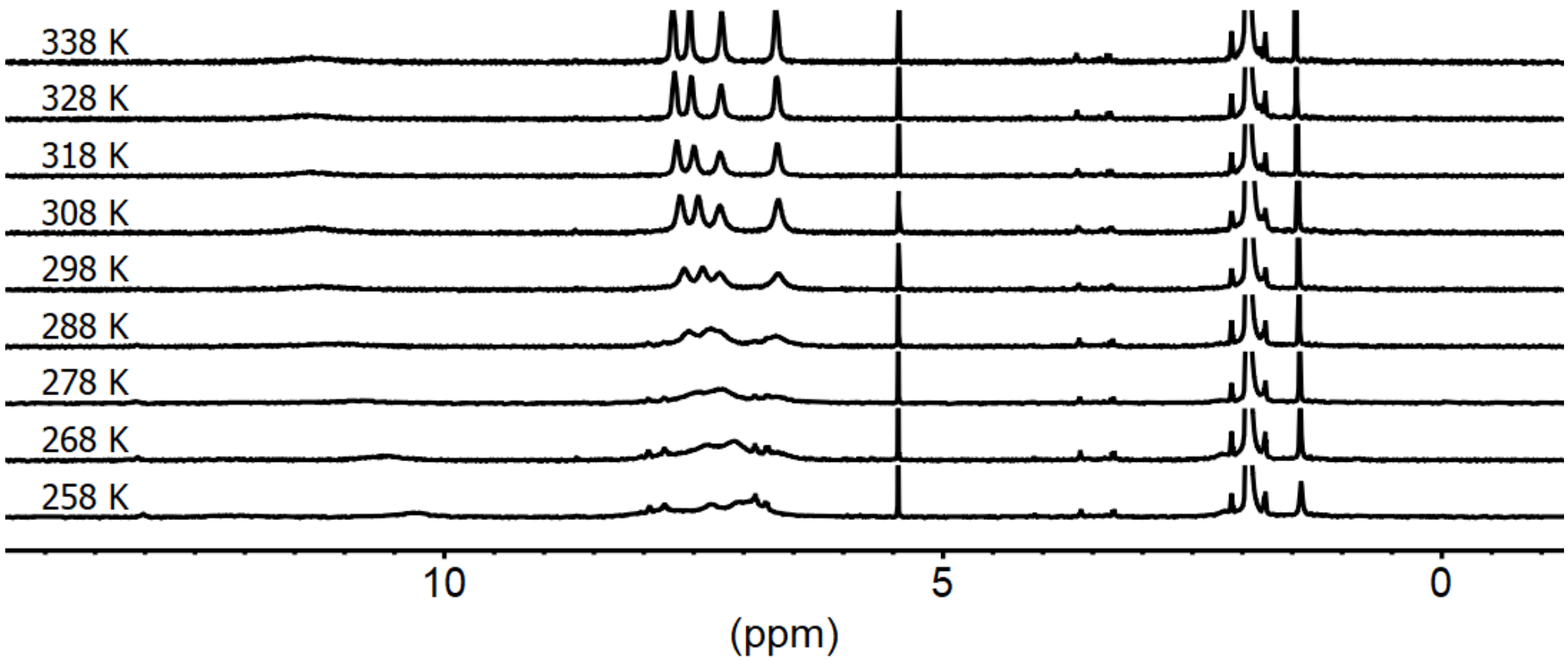

Figure S59. ^{1}H NMR temperature series of [$PuCl_4(HPyO)_2$] in CD_3CN from 258 K to 338 K (−15 to 65 °C).

Table S30. NMR chemical shifts of $[PuCl_4(HPyO)_2]$ in CD_3CN at various temperatures.

**Temperature (K)**	**^{1}H shift (ppm)**				
	N**H**	C**H**-5	C**H**-4	C**H**-6	C**H**-7
**288**		7.54	7.34	7.23	6.67
**298**	11.17	7.60	7.41	7.24	6.65
**308**	11.32	7.63	7.45	7.24	6.65
**318**	11.31	7.67	7.50	7.24	6.66
**328**	11.32	7.69	7.52	7.23	6.67
**338**	11.32	7.71	7.54	7.22	6.67

Cartesian coordinates (in Å) of re-optimized GOAT conformers of [UCl_4(H-phthal)$_2$]

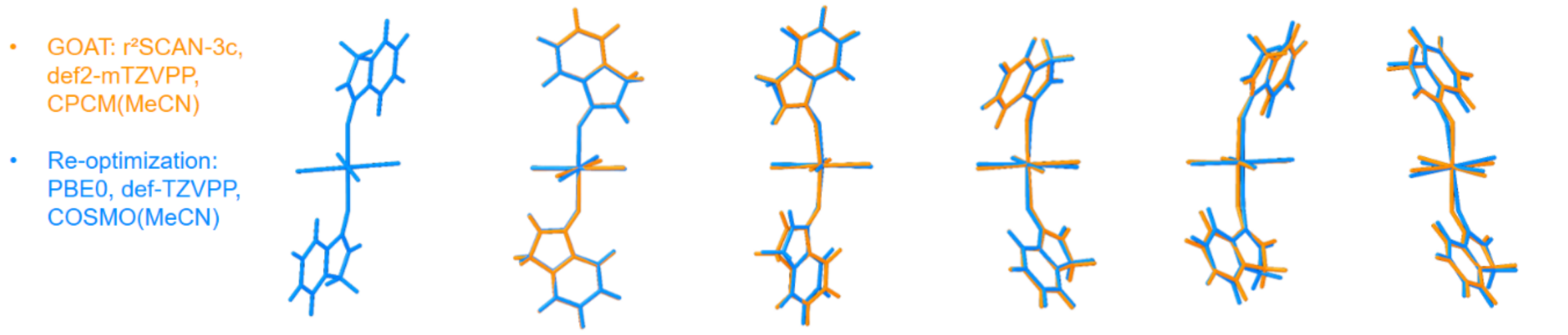

	Opt. from CS	G47	G148	G152	G154	G226
	straight anti	*stretched anti*	*stretched syn*	*bent syn*	*bent anti*	*oppositely bent anti*
ΔG / kJ/mol	0.00	6.02	8.33	4.50	2.15	2.93
Population / %	45.7	5.4	2.4	9.2	21.3	16.1

Figure S60. Selection of conformers for [UCl_4(H-phthal)$_2$]. The table below contains values of Δ*G* (relative to the lowest conformer) and the corresponding Boltzmann population at 338 K.

Table S31. G47

39				H	-3.24710	1.94687	4.45696
				Cl	2.49241	-0.57364	0.56875
U	0.00925	0.03402	0.00647	Cl	-0.31323	-2.39387	-0.86313
Cl	-2.47526	0.63771	-0.56185	N	2.46977	-0.80068	-2.88095
Cl	0.33570	2.46261	0.87010	H	2.63311	-1.30573	-2.02172
N	-2.52329	0.77055	2.87581	O	0.77842	0.51255	-2.06367
H	-2.70871	1.26213	2.01293	C	1.56368	0.15520	-2.98293
O	-0.76042	-0.45387	2.07236	C	1.63650	0.71204	-4.32608
C	-1.56952	-0.13665	2.98579	C	0.89731	1.72732	-4.91422
C	-1.62236	-0.69497	4.32913	H	0.12036	2.24341	-4.36408
C	-0.83487	-1.66921	4.92394	C	1.19612	2.05056	-6.22729
H	-0.02788	-2.14421	4.38007	H	0.64640	2.83784	-6.72788
C	-1.12561	-2.00625	6.23533	C	2.20295	1.37004	-6.91601
H	-0.53882	-2.76295	6.74095	H	2.41643	1.64297	-7.94261
C	-2.17180	-1.37893	6.91585	C	2.93713	0.35306	-6.31742
H	-2.37813	-1.66181	7.94122	H	3.71440	-0.16479	-6.86576
C	-2.95416	-0.40260	6.31065	C	2.64186	0.03006	-5.00556
H	-3.76159	0.07409	6.85269	C	3.23909	-0.99202	-4.09415
C	-2.66698	-0.06595	5.00043	H	4.30124	-0.81877	-3.90617
C	-3.31050	0.92225	4.08316	H	3.11892	-2.01134	-4.46849
H	-4.36063	0.69307	3.88754				

Table S32. G148

39			
U	-1.02960	0.12596	-0.37601
Cl	-2.85746	-1.44547	-1.33134
Cl	-2.59463	2.17087	-0.66090
N	-0.01057	-1.06702	3.22699
H	0.69321	-1.23377	2.52204
O	-1.59084	-0.24887	1.78507
C	-1.20887	-0.59232	2.93435
C	-1.99012	-0.53787	4.16220
C	-3.29281	-0.11272	4.37448
H	-3.90128	0.25327	3.55682
C	-3.77813	-0.17687	5.67002
H	-4.79030	0.14321	5.88376
C	-2.97336	-0.65161	6.70825
H	-3.37780	-0.69154	7.71264
C	-1.66824	-1.07392	6.48473
H	-1.05753	-1.43924	7.30118
C	-1.18225	-1.01234	5.19142
C	0.15160	-1.38623	4.63121
H	0.96808	-0.80512	5.06630
H	0.37929	-2.44696	4.76032
Cl	0.85446	1.65638	0.62155
Cl	0.60643	-1.89166	-0.06963
N	2.25116	0.93885	-2.40921
H	2.34663	1.06995	-1.41158
O	0.01235	0.46001	-2.35754
C	1.09244	0.65163	-2.97581
C	1.28985	0.59846	-4.41765
C	0.38580	0.33074	-5.43444
H	-0.65296	0.11607	-5.21618
C	0.86712	0.35046	-6.73291
H	0.19798	0.14775	-7.55980
C	2.21157	0.62961	-6.98884
H	2.56210	0.63883	-8.01404
C	3.10969	0.89551	-5.96203
H	4.14912	1.10994	-6.17794
C	2.63085	0.87760	-4.66470
C	3.32609	1.11865	-3.36418
H	3.74070	2.12682	-3.29023
H	4.13043	0.40311	-3.17820

Table S33. G152

39				H	-3.78970	-1.41511	4.93966
				Cl	0.80952	0.24534	0.31101
U	-1.34329	-0.75517	-0.75525	Cl	-0.84673	-3.05289	0.39002
Cl	-3.48942	-1.77457	-1.82384	N	1.55224	-2.37289	-3.69118
Cl	-1.89286	1.57923	-1.79235	H	1.13993	-3.28857	-3.60527
N	-2.87584	-1.44698	3.04918	O	-0.08151	-1.27638	-2.53960
H	-3.18807	-2.28622	2.58609	C	1.00361	-1.28588	-3.18090
O	-2.44722	-0.26615	1.13870	C	1.85841	-0.15002	-3.49754
C	-2.46854	-0.37590	2.39484	C	1.71271	1.19446	-3.18851
C	-2.06449	0.62891	3.36636	H	0.85893	1.54797	-2.62405
C	-1.55777	1.90499	3.17209	C	2.70249	2.05667	-3.62918
H	-1.40921	2.30103	2.17524	H	2.63114	3.11504	-3.41145
C	-1.24775	2.64184	4.30269	C	3.79662	1.57536	-4.35174
H	-0.84816	3.64315	4.20175	H	4.55775	2.27199	-4.68249
C	-1.44367	2.10489	5.57742	C	3.93268	0.22634	-4.65683
H	-1.19222	2.70296	6.44520	H	4.78695	-0.13086	-5.21860
C	-1.95003	0.82354	5.76050	C	2.94429	-0.63658	-4.22015
H	-2.09294	0.42198	6.75612	C	2.79846	-2.11392	-4.38417
C	-2.25940	0.08536	4.63275	H	3.61672	-2.67136	-3.92222
C	-2.80097	-1.29969	4.48951	H	2.72645	-2.41607	-5.43163
H	-2.13891	-2.05575	4.91802				

Table S34. G154

39				H	-3.65344	2.12313	4.79650
				Cl	1.54315	2.59720	-0.00583
U	0.45772	0.23250	0.24223	Cl	2.28857	-0.46226	1.97059
Cl	-0.67476	-2.09842	0.53966	N	2.43200	-1.84452	-3.17282
Cl	-1.34750	0.89324	-1.51769	H	2.53884	-2.67555	-2.61266
N	-2.74834	1.64366	2.96339	O	1.69500	-0.51773	-1.47327
H	-2.92520	2.42264	2.34880	C	2.00735	-0.69517	-2.68040
O	-0.83944	0.95827	1.92283	C	1.98120	0.28284	-3.75969
C	-1.70939	0.84047	2.82597	C	1.61720	1.62141	-3.74763
C	-1.76045	-0.15917	3.88363	H	1.28352	2.10064	-2.83586
C	-0.89790	-1.20825	4.16615	C	1.69949	2.31134	-4.94551
H	-0.02255	-1.39038	3.55527	H	1.42625	3.35847	-4.98396
C	-1.20709	-2.00638	5.25473	C	2.13147	1.67020	-6.10871
H	-0.56303	-2.83738	5.51403	H	2.18550	2.23380	-7.03255
C	-2.34512	-1.75239	6.02384	C	2.49405	0.32855	-6.11001
H	-2.56416	-2.39402	6.86903	H	2.82773	-0.15390	-7.02044
C	-3.20210	-0.69763	5.73270	C	2.41461	-0.36232	-4.91478
H	-4.07982	-0.51354	6.33988	C	2.73074	-1.78535	-4.58961
C	-2.89429	0.10262	4.64772	H	3.77818	-2.03612	-4.77295
C	-3.59643	1.29526	4.08581	H	2.10746	-2.49171	-5.14311
H	-4.60881	1.06608	3.74489				

Table S35. G226

39				H	-3.82049	2.02850	4.69864
				Cl	-1.15570	-2.27275	0.57110
U	-0.00672	-0.00650	-0.00482	Cl	-2.03978	0.66967	-1.50505
Cl	1.14310	2.25974	-0.57989	N	2.96766	-1.43288	-2.87216
Cl	2.02769	-0.68290	1.49394	H	3.27843	-2.10065	-2.18406
N	-2.96942	1.43313	2.87367	O	1.03747	-0.86746	-1.79467
H	-3.28138	2.10070	2.18591	C	1.84185	-0.75349	-2.75915
O	-1.04906	0.85569	1.78520	C	1.70779	0.10879	-3.92399
C	-1.84763	0.74808	2.75515	C	0.70378	1.00402	-4.26233
C	-1.70992	-0.11152	3.92156	H	-0.15177	1.15320	-3.61491
C	-0.70800	-1.01063	4.25579	C	0.84594	1.69249	-5.45524
H	0.14222	-1.16606	3.60288	H	0.08700	2.40114	-5.76249
C	-0.84513	-1.69445	5.45196	C	1.96156	1.48350	-6.26953
H	-0.08756	-2.40586	5.75621	H	2.04759	2.03607	-7.19758
C	-1.95380	-1.47706	6.27351	C	2.96293	0.58549	-5.91951
H	-2.03584	-2.02589	7.20413	H	3.82171	0.43580	-6.56220
C	-2.95319	-0.57529	5.92754	C	2.82251	-0.10512	-4.72971
H	-3.80657	-0.41908	6.57584	C	3.70377	-1.12235	-4.08177
C	-2.81790	0.11051	4.73436	H	4.69255	-0.72709	-3.83725
C	-3.69866	1.13000	4.08929	H	3.83427	-2.01828	-4.69310
H	-4.69107	0.73892	3.85289				

Superconducting Quantum Interference Device (SQUID) magnetization measurements

For the SQUID magnetization measurements and the calculation of magnetic parameters, CGS-emu and SI units as well as their conversions known from literature are used.[14] From the measurements, the temperature (K) and the magnetic moment (emu) were obtained, with the SquidLab program (version 2.9.1) being used for background correction and data adjustment with the Levenberg-Marquardt method.[11] The number of data points was reduced by averaging 18 measurement points and the molar magnetic susceptibility χ_{mol} (cm^3/mol)[15] and the effective magnetic moment μ_{eff} (μ_B)[14, 15] were calculated. Intrinsic diamagnetic correction was subtracted from χ_{mol} ([$AnCl_4$(Hphthal.)$_2$]: -0.00027808 $cm^3 \cdot mol^{-1}$; ([$AnCl_4$(HPyO)$_2$]: -0.00022512 $cm^3 \cdot mol^{-1}$).[16]

$$\chi_{\mathrm{mol}}\left(\frac{\mathrm{cm}^3}{\mathrm{mol}}\right) = \frac{\mu(\mathrm{emu})}{H(\mathrm{Oe}) \cdot \left(\frac{m(\mathrm{g})}{M(\mathrm{g}\cdot\mathrm{mol}^{-1})}\right)}$$

$$\mu_{\mathrm{eff}}(\mu_{\mathrm{B}}) = \sqrt{\frac{3k_{\mathrm{B}}}{N_{\mathrm{A}}\mu_{\mathrm{B}}^2}}\sqrt{\chi_{\mathrm{mol}} \cdot T} = \sqrt{8 \cdot \chi_{\mathrm{mol}}\left(\frac{\mathrm{cm}^3}{\mathrm{mol}}\right) \cdot T(\mathrm{K})}$$

To calculate the magnetization per formula unit,[15, 17] the magnetic field (Oe) and the magnetic moment (emu) were obtained from the measurements. By averaging two measurement points, the amount of data was reduced. The magnetic field $\mu_0 H$ (T) was expressed in SI units.

$$M_{f.u.}(\mu_{\mathrm{B}}) = \frac{\mu(\mathrm{emu}) \cdot M\left(\mathrm{g}\cdot\mathrm{mol}^{-1}\right)}{m(\mathrm{g}) \cdot N_A \cdot \mu_B}$$

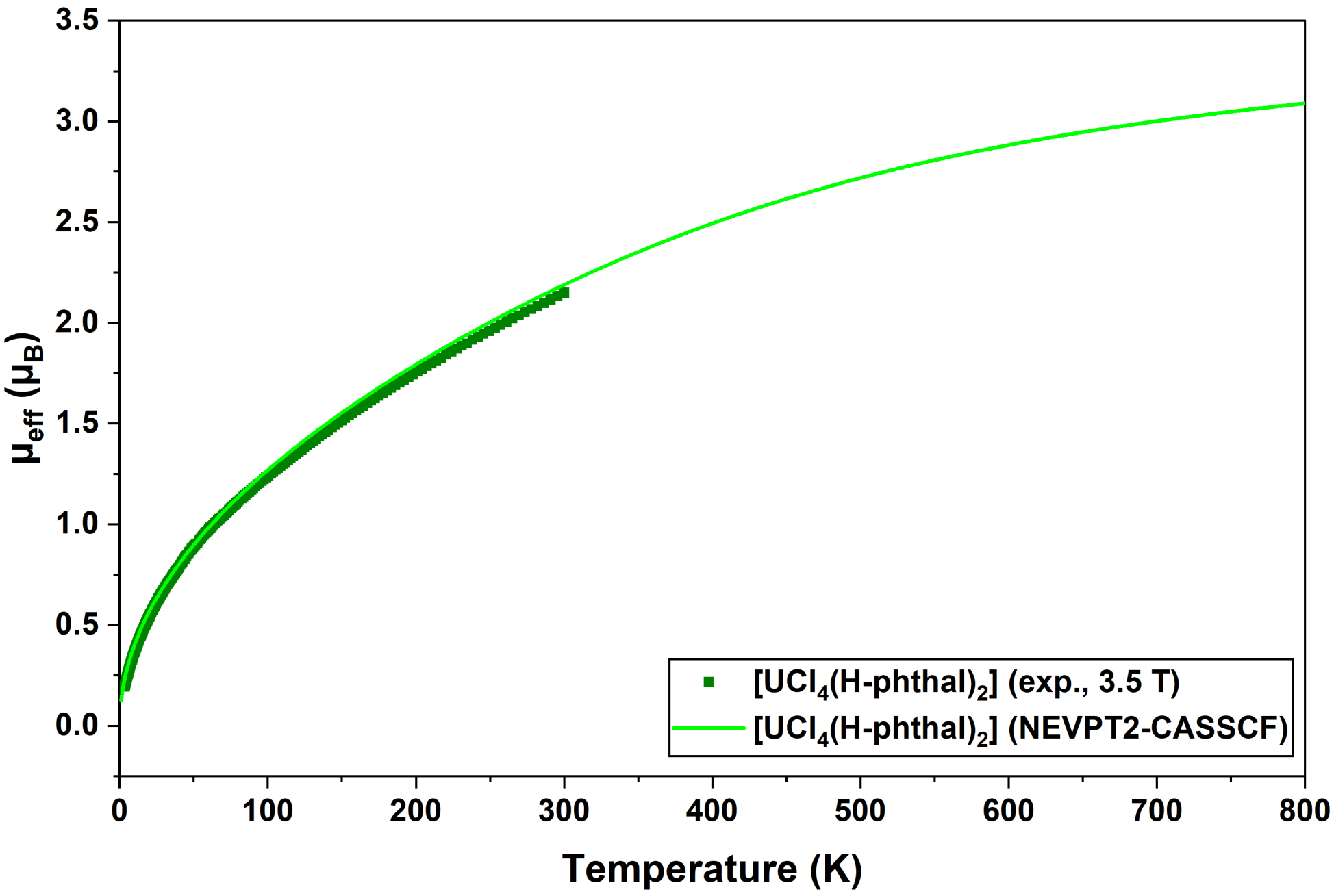

Figure S61. Calculated (NEVPT2-CASSCF, CAS[2,7]) plot of effective magnetic moment *versus* temperature for [UCl_4(H-phthal)$_2$] in the range 1–800 K and experimental curve from 3.5 to 300 K.

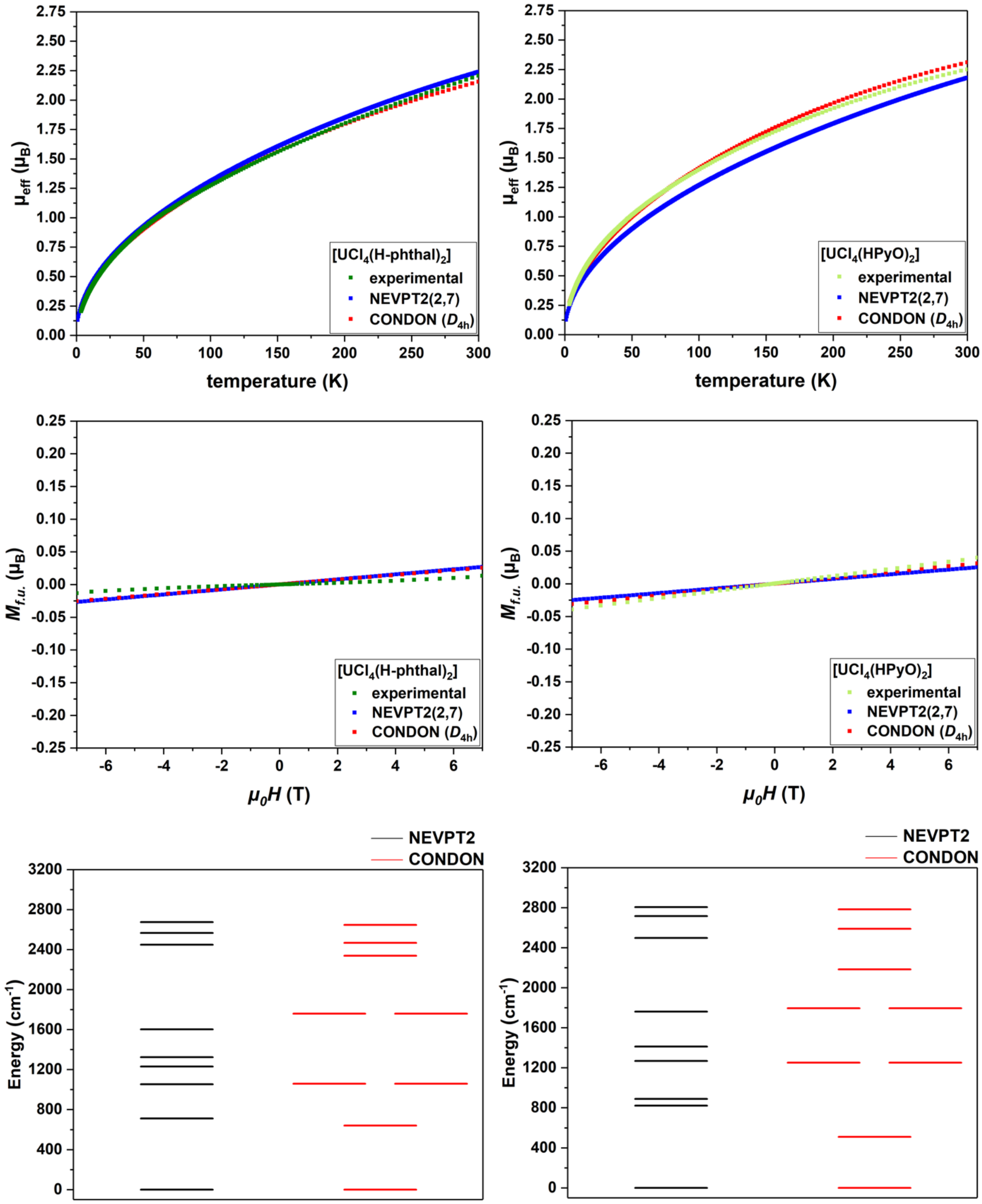

Figure S62. Comparison of effective magnetic moment *versus* temperature plots (top, at 3.5 T), magnetization versus applied magnetic field (middle, at 3.5 K), and energetic splitting of states (bottom), obtained from SQUID magnetometry measurements, CONDON fitting procedure, and NEVPT2 calculations for $[UCl_4(H\text{-phthal})_2]$ (left) and $[UCl_4(HPyO)_2]$ (right).

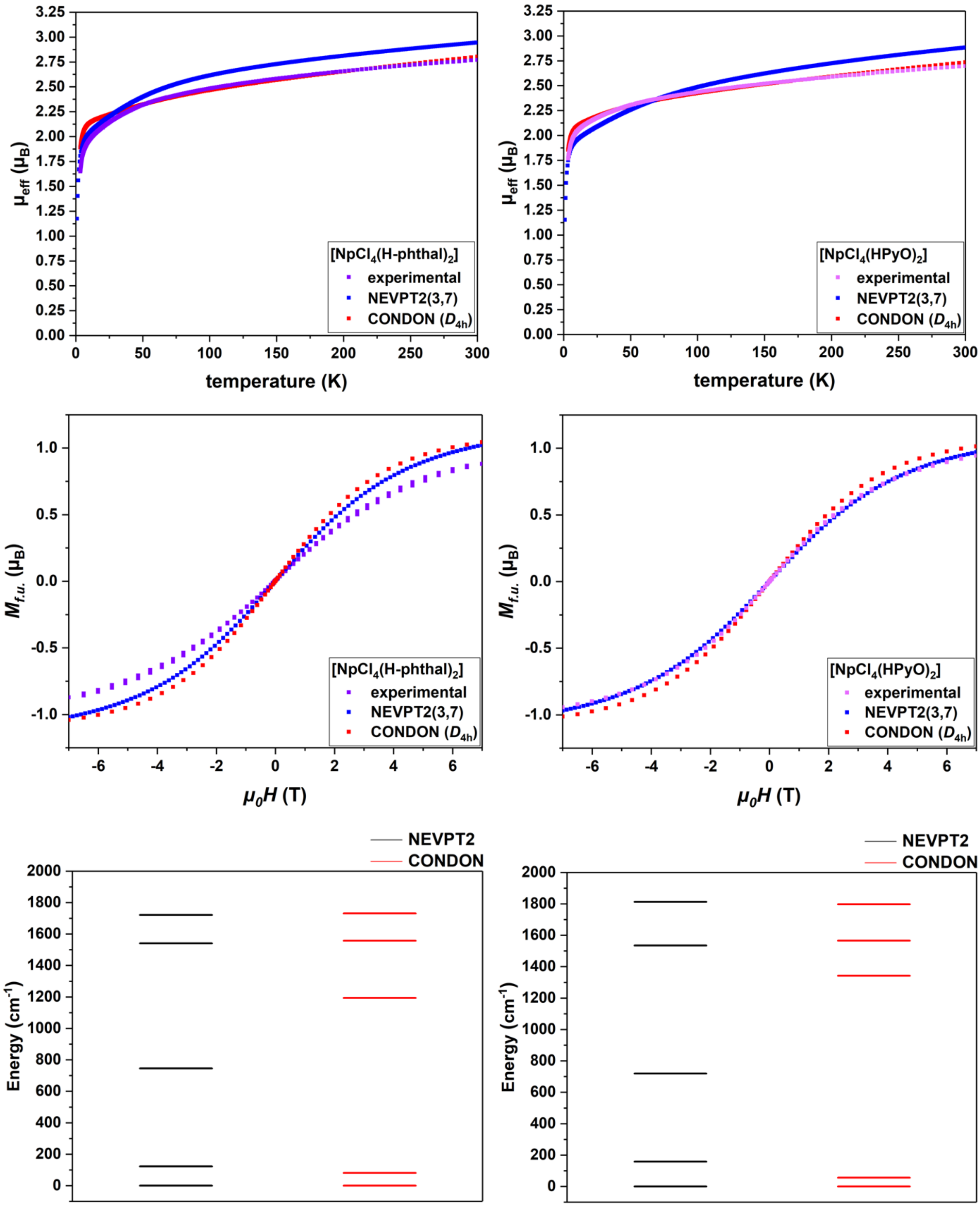

Figure S63. Comparison of effective magnetic moment *versus* temperature plots (top, at 3.5 T), magnetization versus applied magnetic field (middle, at 3.5 K), and energetic splitting of states (bottom), obtained from SQUID magnetometry measurements, CONDON fitting procedure, and NEVPT2 calculations for [$NpCl_4$(H-phthal)$_2$] (left) and [$NpCl_4$(HPyO)$_2$] (right).

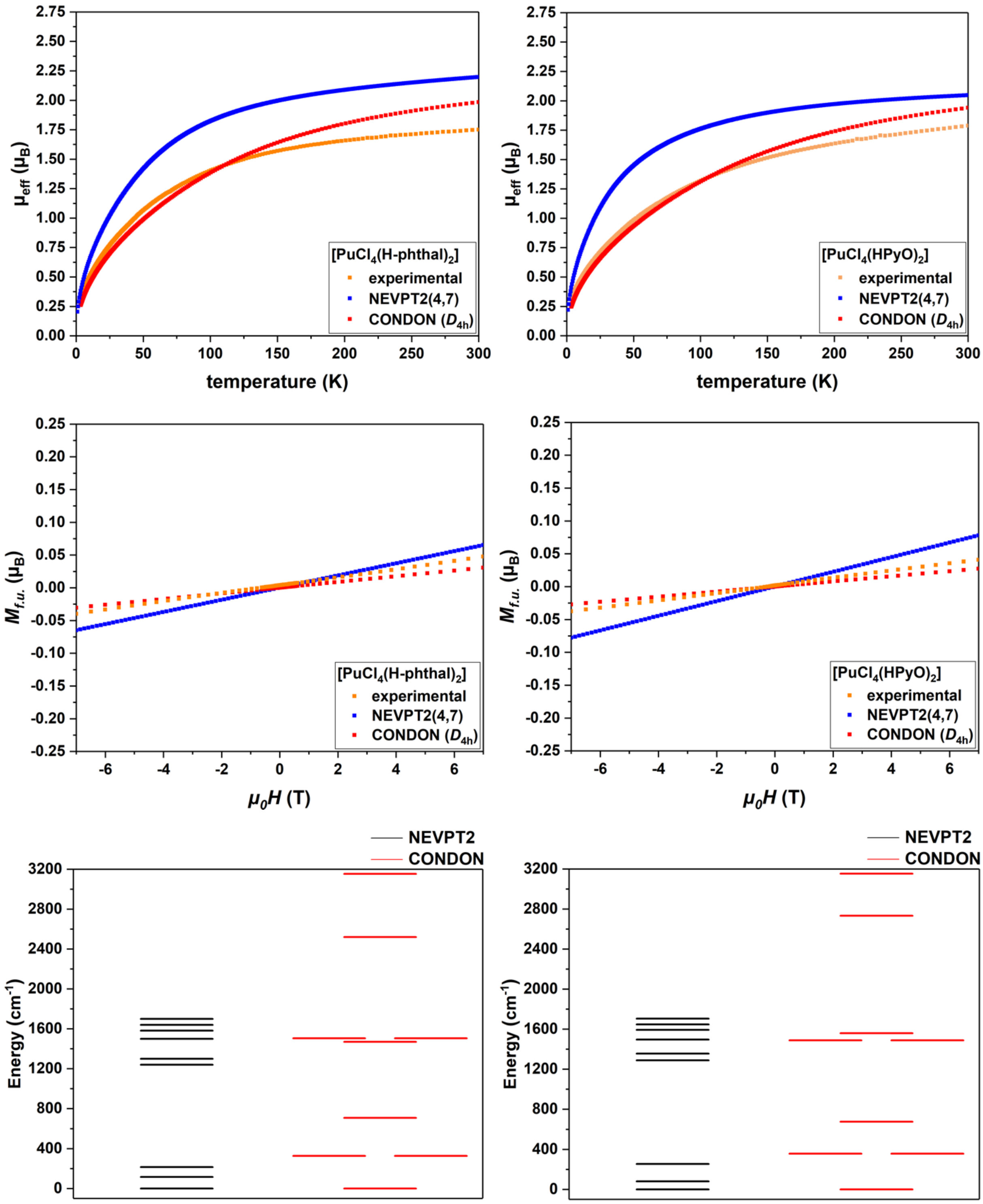

Figure S64. Comparison of effective magnetic moment *versus* temperature plots (top, at 3.5 T), magnetization versus applied magnetic field (middle, at 3.5 K), and energetic splitting of states (bottom), obtained from SQUID magnetometry measurements, CONDON fitting procedure, and NEVPT2 calculations for $[PuCl_4(H\text{-}phthal)_2]$ (left) and $[PuCl_4(HPyO)_2]$ (right).

Spin–orbit parentage analysis

CONDON:

Simulations and/or fitting procedures in CONDON are based on the so-called "full" model.[18, 19] This model employs a Hamiltonian considering interelectronic repulsion, spin-orbit coupling, the electrostatic effect of the ligands and the Zeeman effect of an external magnetic field **B**, respectively. The Hamiltonian acts on the basis set of the "full" basis of microstates.[20] All $(2(2l + 1))!/[N!\cdot(2(2l + 1) - N)!]$ states of the valence electron configuration (l : orbital angular quantum number of the N valence electrons) are incorporated in this basis set. These states may be described in terms of m_l and m_s of the occupied orbitals, e. g. for one state of a d^3 electron configuration as |↑|↑|↓| | | or (2^+, 1^+, 0^-), where "+" indicates spin up and "–" indicates spin down. If a calculation based on all parameters has been done, and while the results may be displayed as chosen by the user, all these results are derived from the eigenvalues (energies) and corresponding eigenstates (wave functions) in terms of the "full" basis of microstates. The latter may be changed to a basis of Russell-Saunders states (see Condon and Shortley, pp. 226 to 228, and p. 208).[20] In CONDON, the eigenstates are analyzed during this transformation, while most other information is lost due to technical reasons except the corresponding energies. All eigenstates in terms of the "full" basis of microstates are compared to a selected Russell-Saunders state. Therefore, starting with the "full" basis of microstates, the composition of the eigenstates in terms of the Russell-Saunders states are consecutively calculated (including the mapping of the corresponding energies).

CASSCF-NEVPT2:

Parentage assignments from ORCA 6.0.1 calculations reported in this study are prepared with a python script we programmed. The source code is available online at http://doi.org/10.14278/rodare.4733.[21] With this approach, the assignment is derived from the spin-free CASSCF and spin–orbit coupled (SOC) CASSCF wavefunctions reported in ORCA. This can be done for both CAS wavefunctions and also NEVPT2 wavefunctions, if available. Our code works for lanthanide- and actinide complexes in different oxidation states. In general, all $f^{\,n}$ configurations with $0 < n < 14$ are treatable. For this procedure to work a database of the free ions is necessary. Our code can both prepare and enlarge this free ion database as well as analyse the results of a complex molecule of interest using CAS or NEVPT2 calculation. In order to generate the reference data, the pure ion result file is fed to the script. The spin-free roots of the corresponding free ion are then grouped according to spin multiplicity and ordered by energy. Using the known Russell–Saunders term structure of the $f^{\,n}$ configuration, roots are partitioned into LS terms (L: total orbital angular quantum number, S: total spin quantum number) based on their characteristic 2L+1 degeneracies. This partitioning is stored in the database. The script checks if only CAS or both CAS and NEVPT2 values are available and extracts both if possible. With the appropriate values in the free ion database, it is then possible to establish a mapping between individual spin-free roots and the parent free-ion terms. The resulting assignment is subsequently used to analyse the spin-orbit eigenvectors as reported by ORCA. For each SOC state, the ORCA spin–orbit eigenvector expansion is analysed and the contributions of all spin-free basis functions belonging to the same LS term are summed. The accumulated weights yield the percentage parentage of each free-ion term in the final SOC state. Our method relies on the approximate preservation of free-ion term character and is therefore most reliable when the spin-free manifold retains a recognizable atomic ordering. In systems with strong ligand-field effects or extensive configuration interaction, substantial mixing between terms may occur, reducing the uniqueness of the assignment. A particular limitation arises when several LS terms possess identical or very similar degeneracies. For example, in the $5f^3$ configuration of Np^{IV}, multiple doublet terms such as ^2H, ^2G, ^2F and ^2D occur repeatedly and exhibit identical degeneracies within a given multiplicity manifold. In such cases, the automated assignment cannot distinguish terms solely from degeneracy and energy ordering. The procedure therefore assumes that the ordering of spin-free roots follows the conventional free-ion sequence. While this assumption is generally valid for weakly perturbed free-ion calculations, accidental reordering of nearly degenerate terms may lead to ambiguities in the assigned parentage. Consequently, the reported term labels should be interpreted as

dominant parent-term descriptors rather than rigorous quantum numbers in strongly mixed regions of the spectrum. For the values close to the ground state the mixing is not so strong as to cause any problems. An interesting additional problem can occur when two close lying multiplets would be grouped together and add up to a false multiplet. Our script has the correct number of states for all f^n configurations hardcoded. If a wrong multiplet is detected it is separated into the appropriate two multiplets by scanning the splitting in the multiplet. Despite the described limitations, the approach provides a practical and physically transparent connection between SOC states of the complex and the underlying free-ion electronic structure, allowing the evolution of atomic term character under ligand field and spin–orbit interactions to be quantified directly from the *ab initio* wavefunctions.

[UCl_4(H-phthal)$_2$]

Table S36. Calculated energies (cm^{-1}) of the electronic states in [UCl_4(H-phthal)$_2$] with term symbol and parentage as determined from CASSCF-NEVPT2 calculations using ORCA.

Ion	U(4+)	Configuration	$5f^2$	Energy source	NEVPT2

Section	Level	Energy / cm^{-1}	Label	Root(s)/state	Multiplicity/Parentage
Free ion spin-free	1	14.84	^3H	roots 0-10	3
	2	3295.63	^3F	roots 11-17	3
	3	4923.54	^1G	roots 0-8	1
	4	12094.74	^1D	roots 9-13	1
	5	15743.70	^3P	roots 18-20	3
	6	16434.30	^1I	roots 14-26	1
	7	37902.20	^1S	roots 27	1
Complex spin-free	1	0.00	^3H	roots 0-10	3
	2	5514.40	^3F	roots 11-17	3
	3	5533.80	^1G	roots 0-8	1
	4	11568.00	^1D	roots 9-13	1
	5	16036.30	^3P	roots 18-20	3
	6	16071.60	^1I	roots 14-26	1
	7	38128.40	^1S	roots 27	1
Complex SOC	1	0.00	^3H	STATE 0	^3H 91.0%; ^1G 7.9%
	2	711.02	^3H	STATE 1	^3H 90.0%; ^1G 9.0%; omitted 1.0%
	3	1053.42	^3H	STATE 2	^3H 89.8%; ^1G 9.3%
	4	1230.48	^3H	STATE 3	^3H 89.5%; ^1G 9.9%
	5	1324.22	^3H	STATE 4	^3H 87.5%; ^1G 11.5%
	6	1602.64	^3H	STATE 5	^3H 87.0%; ^1G 11.8%
	7	2448.63	^3H	STATE 6	^3H 86.3%; ^1G 7.2%; ^3F 5.6%
	8	2565.44	^3H	STATE 7	^3H 84.8%; ^1G 8.1%; ^3F 6.0%
	9	2675.10	^3H	STATE 8	^3H 84.6%; ^1G 7.4%; ^3F 6.9%
	10	5866.45	^3H	STATE 9	^3H 61.5%; ^3F 30.2%; ^1D 6.6%; omitted 1.3%
	11	5881.70	^3H	STATE 10	^3H 59.5%; ^3F 31.9%; ^1D 7.3%; omitted 1.0%
	12	6031.43	^3H	STATE 11	^3H 53.8%; ^3F 38.3%; ^1D 5.9%; omitted 1.1%
	13	6101.12	^3F	STATE 12	^3F 46.4%; ^3H 41.8%; ^1D 10.8%
	14	6167.28	^3F	STATE 13	^3F 54.7%; ^3H 32.4%; ^1D 11.4%
	15	6685.40	^3H	STATE 14	^3H 92.7%; ^3F 4.5%; ^1G 1.1%
	16	6786.15	^3H	STATE 15	^3H 89.0%; ^3F 8.0%; ^1D 1.3%; ^1G 1.3%
	17	6984.95	^3H	STATE 16	^3H 97.6%; ^1G 1.4%
	18	7425.28	^3H	STATE 17	^3H 69.3%; ^3F 26.0%; ^1D 3.3%
	19	7509.32	^3H	STATE 18	^3H 71.0%; ^3F 25.2%; ^1D 2.8%
	20	7679.04	^3H	STATE 19	^3H 56.3%; ^3F 37.9%; ^1D 4.8%

21	7941.85	^{3}H	STATE 20	^{3}H 95.5%; ^{1}G 2.6%; omitted 1.1%
22	8041.15	^{3}H	STATE 21	^{3}H 95.7%; ^{1}G 3.2%
23	8292.19	^{3}H	STATE 22	^{3}H 95.8%; ^{1}G 3.1%
24	8479.52	^{3}F	STATE 23	^{3}F 51.0%; ^{3}H 43.3%; ^{1}D 2.4%; ^{1}G 2.1%
25	8605.63	^{3}F	STATE 24	^{3}F 54.8%; ^{3}H 37.8%; ^{1}G 3.0%; ^{1}D 2.9%; omitted 1.1%
26	9874.26	^{3}H	STATE 25	^{3}H 47.1%; ^{1}G 38.4%; ^{3}F 13.8%
27	10436.01	^{3}H	STATE 26	^{3}H 42.5%; ^{3}F 33.1%; ^{1}G 23.8%
28	10476.25	^{3}H	STATE 27	^{3}H 40.8%; ^{3}F 32.7%; ^{1}G 26.1%
29	10561.31	^{3}H	STATE 28	^{3}H 39.9%; ^{3}F 36.2%; ^{1}G 22.6%; omitted 1.3%
30	10688.60	^{3}F	STATE 29	^{3}F 54.8%; ^{3}H 35.0%; ^{1}G 8.9%
31	10731.37	^{3}F	STATE 30	^{3}F 53.2%; ^{3}H 34.1%; ^{1}G 11.6%
32	10853.55	^{3}F	STATE 31	^{3}F 44.0%; ^{1}G 32.3%; ^{3}H 23.0%
33	10878.50	^{3}F	STATE 32	^{3}F 57.5%; ^{3}H 23.8%; ^{1}G 17.5%; omitted 1.2%
34	11016.12	^{1}G	STATE 33	^{1}G 36.3%; ^{3}H 33.1%; ^{3}F 29.0%
35	11296.22	^{3}F	STATE 34	^{3}F 85.5%; ^{3}H 7.4%; ^{1}G 6.2%
36	11413.82	^{3}F	STATE 35	^{3}F 60.4%; ^{3}H 21.3%; ^{1}G 16.6%; omitted 1.2%
37	11476.87	^{3}F	STATE 36	^{3}F 74.9%; ^{1}G 19.3%; ^{3}H 4.9%
38	11583.04	^{3}F	STATE 37	^{3}F 78.3%; ^{1}G 17.0%; ^{3}H 3.3%; omitted 1.4%
39	11687.46	^{3}H	STATE 38	^{3}H 77.1%; ^{3}F 13.1%; ^{1}G 6.3%; ^{1}I 1.7%
40	11801.02	^{3}H	STATE 39	^{3}H 66.1%; ^{3}F 23.8%; ^{1}G 6.5%; ^{1}I 1.5%
41	12023.32	^{3}H	STATE 40	^{3}H 87.4%; ^{3}F 5.4%; ^{1}G 2.9%; ^{1}I 2.0%; omitted 1.1%
42	12107.10	^{3}H	STATE 41	^{3}H 84.8%; ^{3}F 6.5%; ^{1}G 3.9%; ^{1}I 1.8%; omitted 1.2%
43	12256.01	^{3}H	STATE 42	^{3}H 75.5%; ^{3}F 15.4%; ^{1}G 5.2%; ^{1}I 1.7%; omitted 1.1%
44	12360.05	^{3}F	STATE 43	^{3}F 58.4%; ^{1}G 28.7%; ^{3}H 12.0%
45	12412.07	^{3}F	STATE 44	^{3}F 61.5%; ^{1}G 29.7%; ^{3}H 8.1%
46	12441.83	^{3}F	STATE 45	^{3}F 55.6%; ^{1}G 29.0%; ^{3}H 13.5%; omitted 1.4%
47	13011.69	^{3}H	STATE 46	^{3}H 82.5%; ^{3}F 12.8%; ^{1}I 3.3%; omitted 1.1%
48	13716.55	^{3}H	STATE 47	^{3}H 72.8%; ^{3}F 20.4%; ^{1}I 5.3%; ^{1}G 1.0%
49	13764.79	^{3}H	STATE 48	^{3}H 68.3%; ^{3}F 23.7%; ^{1}I 5.2%; ^{1}G 2.3%
50	13860.64	^{3}H	STATE 49	^{3}H 66.0%; ^{3}F 25.2%; ^{1}I 5.2%; ^{1}G 2.7%
51	14062.27	^{3}H	STATE 50	^{3}H 58.2%; ^{3}F 31.1%; ^{1}I 5.4%; ^{1}G 4.7%
52	14137.45	^{3}H	STATE 51	^{3}H 55.2%; ^{3}F 31.3%; ^{1}G 7.5%; ^{1}I 5.5%
53	14301.92	^{3}H	STATE 52	^{3}H 54.4%; ^{3}F 31.3%; ^{1}G 8.1%; ^{1}I 5.5%
54	14326.17	^{3}H	STATE 53	^{3}H 55.4%; ^{3}F 33.1%; ^{1}I 5.6%; ^{1}G 5.2%
55	17191.36	^{3}F	STATE 54	^{3}F 46.4%; ^{1}G 38.5%; ^{3}H 7.3%; ^{3}P 3.3%; omitted 1.2%
56	17398.34	^{3}F	STATE 55	^{3}F 51.1%; ^{1}G 28.8%; ^{3}H 6.9%; ^{3}P 6.0%; omitted 1.1%

57	17559.46	^{3}F	STATE 56	^{3}F 48.2%; ^{1}G 32.7%; ^{3}H 7.4%; ^{3}P 5.0%
58	17585.93	^{3}F	STATE 57	^{3}F 52.0%; ^{1}G 36.5%; ^{3}H 6.2%; ^{3}P 1.3%; omitted 1.9%
59	17689.50	^{3}F	STATE 58	^{3}F 51.6%; ^{1}G 38.2%; ^{3}H 6.4%; ^{1}I 1.4%; omitted 1.3%
60	17713.07	^{3}F	STATE 59	^{3}F 51.5%; ^{1}G 37.0%; ^{3}H 7.0%; ^{1}I 2.0%; omitted 1.6%
61	18005.96	^{1}D	STATE 60	^{1}D 50.3%; ^{3}P 23.6%; ^{3}F 17.7%; ^{1}G 5.7%; omitted 1.2%
62	18084.86	^{1}D	STATE 61	^{1}D 55.5%; ^{3}P 25.2%; ^{3}F 15.1%; ^{1}G 1.7%; omitted 1.1%
63	18254.04	^{1}D	STATE 62	^{1}D 56.3%; ^{3}P 23.9%; ^{3}F 14.9%; ^{1}G 2.7%; omitted 1.0%
64	19033.15	^{3}F	STATE 63	^{3}F 46.2%; ^{1}G 40.5%; ^{1}D 7.2%; ^{3}P 2.8%; omitted 1.8%
65	19234.09	^{1}G	STATE 64	^{1}G 41.4%; ^{3}F 40.2%; ^{3}P 9.0%; ^{1}D 5.7%; omitted 1.3%
66	19328.42	^{3}P	STATE 65	^{3}P 44.1%; ^{3}F 19.3%; ^{1}D 18.9%; ^{1}G 13.9%; omitted 1.2%
67	19358.08	^{1}G	STATE 66	^{1}G 44.6%; ^{3}F 42.7%; ^{3}P 5.2%; ^{1}D 4.7%; omitted 1.4%
68	19647.28	^{1}D	STATE 67	^{1}D 43.8%; ^{3}P 34.6%; ^{3}F 14.5%; ^{1}G 4.1%
69	19727.88	^{3}P	STATE 68	^{3}P 58.7%; ^{1}D 25.7%; ^{3}F 9.1%; ^{1}S 2.9%
70	21937.54	^{3}P	STATE 69	^{3}P 97.2%; ^{1}I 1.5%
71	21994.35	^{3}P	STATE 70	^{3}P 97.3%; ^{1}I 1.3%
72	22322.52	^{3}P	STATE 71	^{3}P 96.6%; ^{1}I 2.8%
73	23283.57	^{1}I	STATE 72	^{1}I 88.7%; ^{3}F 3.9%; ^{3}H 2.6%; ^{3}P 1.6%; omitted 1.9%
74	23320.49	^{1}I	STATE 73	^{1}I 90.6%; ^{3}F 4.8%; ^{3}H 2.5%; omitted 1.7%
75	23434.57	^{1}I	STATE 74	^{1}I 91.4%; ^{3}H 3.7%; ^{3}F 2.4%; omitted 1.7%
76	23501.22	^{1}I	STATE 75	^{1}I 89.0%; ^{3}H 3.6%; ^{3}F 2.9%; ^{3}P 2.2%; omitted 1.3%
77	23704.94	^{1}I	STATE 76	^{1}I 90.0%; ^{3}H 3.4%; ^{3}F 2.7%; ^{3}P 1.9%; omitted 1.5%
78	23755.59	^{1}I	STATE 77	^{1}I 92.1%; ^{3}H 3.8%; ^{3}F 1.7%; omitted 1.3%
79	24041.86	^{1}I	STATE 78	^{1}I 93.0%; ^{3}H 3.7%; ^{3}F 1.5%; omitted 1.5%
80	24804.10	^{1}I	STATE 79	^{1}I 87.4%; ^{3}P 4.2%; ^{1}D 4.2%; ^{3}H 3.1%; omitted 1.0%
81	25067.45	^{1}I	STATE 80	^{1}I 55.3%; ^{3}P 28.9%; ^{1}D 12.7%; ^{3}H 1.4%; omitted 1.4%
82	25333.25	^{3}P	STATE 81	^{3}P 48.1%; ^{1}I 31.5%; ^{1}D 17.7%; ^{3}H 1.1%
83	25484.69	^{1}I	STATE 82	^{1}I 47.9%; ^{3}P 28.5%; ^{1}D 20.9%; ^{3}H 1.4%; omitted 1.1%
84	25625.42	^{3}P	STATE 83	^{3}P 48.7%; ^{1}I 30.7%; ^{1}D 17.9%; omitted 1.3%
85	25731.97	^{1}I	STATE 84	^{1}I 42.5%; ^{3}P 29.1%; ^{1}D 25.4%; ^{3}H 1.5%; omitted 1.4%

86	27086.52	^{3}P	STATE 85	^{3}P 47.0%; ^{1}I 37.3%; ^{1}D 12.8%; ^{3}F 1.5%; omitted 1.3%
87	27318.13	^{1}I	STATE 86	^{1}I 64.9%; ^{3}P 25.5%; ^{1}D 6.3%; ^{3}F 1.1%; omitted 1.1%
88	27459.57	^{1}I	STATE 87	^{1}I 69.3%; ^{3}P 22.1%; ^{1}D 5.4%; ^{3}H 1.2%; omitted 1.1%
89	28502.99	^{1}I	STATE 88	^{1}I 56.8%; ^{3}P 30.0%; ^{1}D 10.8%; ^{3}F 1.2%; omitted 1.3%
90	28956.73	^{1}I	STATE 89	^{1}I 59.4%; ^{3}P 30.0%; ^{1}D 8.1%; ^{3}F 1.1%; omitted 1.2%
91	46357.56	^{1}S	STATE 90	^{1}S 93.6%; ^{3}P 6.3%

[$UCl_4(HPyO)_2$]

Table S37. Calculated energies (cm^{-1}) of the electronic states in [$UCl_4(HPyO)_2$] with term symbol and parentage as determined from CASSCF-NEVPT2 calculations using ORCA.

Ion	U(4+)	Configuration	$5f^2$	Energy source	NEVPT2

Section	Level	Energy / cm^{-1}	Label	Root(s)/state	Multiplicity/Parentage
Free ion spin-free	1	14.84	^3H	roots 0-10	3
	2	3295.63	^3F	roots 11-17	3
	3	4923.54	^1G	roots 0-8	1
	4	12094.74	^1D	roots 9-13	1
	5	15743.70	^3P	roots 18-20	3
	6	16434.30	^1I	roots 14-26	1
	7	37902.20	^1S	roots 27	1
Complex spin-free	1	0.00	^3H	roots 0-10	3
	2	5495.00	^1G	roots 0-8	1
	3	5769.20	^3F	roots 11-17	3
	4	11475.70	^1D	roots 9-13	1
	5	16038.70	^1I	roots 14-26	1
	6	16159.30	^3P	roots 18-20	3
	7	37969.60	^1S	roots 27	1
Complex SOC	1	0.00	^3H	STATE 0	^3H 91.2%; ^1G 8.0%
	2	821.35	^3H	STATE 1	^3H 90.3%; ^1G 8.9%
	3	888.65	^3H	STATE 2	^3H 90.1%; ^1G 9.2%
	4	1267.88	^3H	STATE 3	^3H 86.8%; ^1G 11.6%
	5	1411.08	^3H	STATE 4	^3H 89.5%; ^1G 9.5%
	6	1760.28	^3H	STATE 5	^3H 87.3%; ^1G 11.6%
	7	2496.45	^3H	STATE 6	^3H 86.2%; ^1G 7.0%; ^3F 5.6%
	8	2714.97	^3H	STATE 7	^3H 84.9%; ^1G 8.0%; ^3F 6.1%
	9	2804.92	^3H	STATE 8	^3H 84.6%; ^1G 7.4%; ^3F 6.9%
	10	5891.94	^3H	STATE 9	^3H 55.4%; ^3F 34.4%; ^1D 9.0%
	11	5970.94	^3H	STATE 10	^3H 62.5%; ^3F 30.7%; ^1D 5.4%
	12	6022.99	^3H	STATE 11	^3H 59.3%; ^3F 33.7%; ^1D 5.5%
	13	6126.82	^3H	STATE 12	^3H 44.3%; ^3F 43.8%; ^1D 10.3%
	14	6213.52	^3H	STATE 13	^3H 46.6%; ^3F 42.0%; ^1D 10.1%
	15	6699.09	^3H	STATE 14	^3H 94.7%; ^3F 2.4%; ^1G 1.6%
	16	6849.45	^3H	STATE 15	^3H 91.7%; ^3F 5.4%; ^1G 1.2%
	17	6895.44	^3H	STATE 16	^3H 84.6%; ^3F 11.6%; ^1D 2.0%
	18	7485.43	^3H	STATE 17	^3H 57.0%; ^3F 36.8%; ^1D 5.1%
	19	7641.55	^3H	STATE 18	^3H 68.7%; ^3F 27.3%; ^1D 3.1%
	20	7753.81	^3H	STATE 19	^3H 64.1%; ^3F 30.8%; ^1D 3.8%
	21	8033.71	^3H	STATE 20	^3H 95.0%; ^1G 3.4%
	22	8072.18	^3H	STATE 21	^3H 95.0%; ^1G 3.6%
	23	8287.64	^3H	STATE 22	^3H 92.5%; ^3F 3.6%; ^1G 3.2%

24	8668.48	^3F	STATE 23	^3F 54.0%; ^3H 39.2%; ^1G 2.8%; ^1D 2.6%
25	8677.43	^3F	STATE 24	^3F 52.1%; ^3H 40.0%; ^1G 4.0%; ^1D 2.6%
26	9812.73	^3H	STATE 25	^3H 48.3%; ^1G 38.2%; ^3F 12.5%
27	10488.89	^3H	STATE 26	^3H 44.8%; ^3F 29.7%; ^1G 24.8%
28	10518.72	^3H	STATE 27	^3H 44.8%; ^3F 30.9%; ^1G 23.6%
29	10642.69	^3H	STATE 28	^3H 43.9%; ^3F 32.1%; ^1G 23.4%
30	10702.29	^3F	STATE 29	^3F 53.5%; ^3H 34.7%; ^1G 11.0%
31	10750.52	^3F	STATE 30	^3F 55.4%; ^3H 34.3%; ^1G 9.3%
32	10899.07	^1G	STATE 31	^1G 40.9%; ^3F 31.3%; ^3H 26.9%
33	10969.52	^3F	STATE 32	^3F 57.6%; ^3H 28.8%; ^1G 12.4%; omitted 1.1%
34	11073.52	^1G	STATE 33	^1G 34.2%; ^3H 33.7%; ^3F 30.5%
35	11217.38	^3F	STATE 34	^3F 85.9%; ^3H 7.4%; ^1G 5.9%
36	11427.18	^3F	STATE 35	^3F 62.4%; ^1G 18.9%; ^3H 17.1%
37	11535.82	^3F	STATE 36	^3F 72.9%; ^3H 14.0%; ^1G 12.3%
38	11656.44	^3F	STATE 37	^3F 74.2%; ^1G 15.1%; ^3H 9.6%; omitted 1.0%
39	11727.33	^3H	STATE 38	^3H 50.3%; ^3F 31.4%; ^1G 15.2%; ^1D 1.2%
40	11840.82	^3H	STATE 39	^3H 91.7%; ^3F 2.3%; ^1I 2.2%; ^1D 1.2%; omitted 1.6%
41	11874.97	^3H	STATE 40	^3H 85.3%; ^3F 8.6%; ^1I 2.0%; ^1G 1.6%; omitted 1.4%
42	12118.00	^3H	STATE 41	^3H 73.9%; ^3F 14.6%; ^1G 7.1%; ^1D 1.8%; omitted 1.1%
43	12301.75	^3H	STATE 42	^3H 67.8%; ^3F 19.4%; ^1G 9.1%; ^1I 1.5%; omitted 1.1%
44	12452.19	^3F	STATE 43	^3F 55.8%; ^1G 25.5%; ^3H 17.4%
45	12507.33	^3F	STATE 44	^3F 64.6%; ^1G 30.5%; ^3H 4.0%
46	12516.06	^3F	STATE 45	^3F 55.6%; ^1G 26.8%; ^3H 16.2%
47	13062.94	^3H	STATE 46	^3H 82.1%; ^3F 13.5%; ^1I 3.2%
48	13845.77	^3H	STATE 47	^3H 68.4%; ^3F 22.7%; ^1I 5.1%; ^1G 2.9%
49	13903.64	^3H	STATE 48	^3H 67.9%; ^3F 25.3%; ^1I 5.1%; omitted 1.0%
50	14014.87	^3H	STATE 49	^3H 64.4%; ^3F 27.2%; ^1I 5.5%; ^1G 2.0%
51	14126.63	^3H	STATE 50	^3H 56.3%; ^3F 31.2%; ^1G 6.2%; ^1I 5.5%
52	14159.82	^3H	STATE 51	^3H 56.1%; ^3F 32.3%; ^1I 5.5%; ^1G 5.3%
53	14385.59	^3H	STATE 52	^3H 52.5%; ^3F 28.9%; ^1G 12.4%; ^1I 5.2%
54	14457.68	^3H	STATE 53	^3H 53.6%; ^3F 35.0%; ^1I 5.7%; ^1G 4.9%
55	17159.67	^3F	STATE 54	^3F 47.7%; ^1G 36.1%; ^3H 7.0%; ^3P 3.7%; omitted 1.4%
56	17429.68	^3F	STATE 55	^3F 52.3%; ^1G 28.2%; ^3H 7.0%; ^3P 5.4%; omitted 1.5%
57	17517.56	^3F	STATE 56	^3F 47.5%; ^1G 32.5%; ^3H 7.4%; ^3P 5.6%
58	17601.62	^3F	STATE 57	^3F 53.0%; ^1G 36.6%; ^3H 7.1%; ^1I 1.6%; omitted 1.2%

59	17660.83	^3F	STATE 58	^3F 51.9%; ^1G 34.3%; ^3H 6.8%; ^1D 2.6%
60	17708.08	^3F	STATE 59	^3F 50.1%; ^1G 38.5%; ^3H 7.2%; ^1I 2.0%; omitted 1.2%
61	17982.15	^1D	STATE 60	^1D 50.1%; ^3P 23.5%; ^3F 17.9%; ^1G 5.5%; omitted 1.2%
62	18161.71	^1D	STATE 61	^1D 55.0%; ^3P 24.3%; ^3F 15.2%; ^1G 2.9%; omitted 1.0%
63	18172.54	^1D	STATE 62	^1D 55.0%; ^3P 23.7%; ^3F 16.0%; ^1G 2.9%; omitted 1.2%
64	19105.68	^3F	STATE 63	^3F 46.4%; ^1G 41.6%; ^1D 6.3%; ^3P 3.0%; omitted 1.4%
65	19263.72	^1G	STATE 64	^1G 47.3%; ^3F 45.0%; ^1D 3.6%; ^3H 1.6%; omitted 1.2%
66	19336.46	^1G	STATE 65	^1G 39.1%; ^3F 38.1%; ^3P 13.5%; ^1D 5.6%; omitted 1.0%
67	19389.06	^3P	STATE 66	^3P 53.6%; ^3F 16.1%; ^1D 13.2%; ^1G 12.2%; omitted 1.2%
68	19547.73	^1D	STATE 67	^1D 44.4%; ^3P 33.5%; ^3F 15.1%; ^1G 4.5%
69	19861.94	^3P	STATE 68	^3P 49.7%; ^1D 33.2%; ^3F 10.5%; ^1G 2.5%
70	21808.69	^3P	STATE 69	^3P 98.0%; ^1I 1.4%
71	22179.07	^3P	STATE 70	^3P 96.1%; ^1I 2.1%
72	22231.90	^3P	STATE 71	^3P 95.1%; ^1I 3.3%
73	23210.59	^1I	STATE 72	^1I 88.6%; ^3H 4.1%; ^3F 2.3%; ^3P 1.9%; omitted 1.3%
74	23293.07	^1I	STATE 73	^1I 90.6%; ^3F 4.9%; ^3H 2.7%; omitted 1.2%
75	23338.91	^1I	STATE 74	^1I 88.9%; ^3F 4.0%; ^3H 2.8%; ^3P 2.1%; omitted 1.9%
76	23372.28	^1I	STATE 75	^1I 88.4%; ^3F 4.0%; ^3H 3.0%; ^3P 2.6%; omitted 1.6%
77	23673.54	^1I	STATE 76	^1I 90.0%; ^3F 4.0%; ^3H 3.1%; ^3P 1.2%; omitted 1.2%
78	23902.48	^1I	STATE 77	^1I 93.3%; ^3H 3.8%; ^3F 1.4%; omitted 1.1%
79	23983.88	^1I	STATE 78	^1I 92.8%; ^3H 3.6%; ^3F 1.6%; omitted 1.6%
80	24905.00	^1I	STATE 79	^1I 95.4%; ^3H 3.0%; omitted 1.1%
81	25148.46	^1I	STATE 80	^1I 46.6%; ^3P 36.9%; ^1D 13.5%; omitted 1.5%
82	25205.64	^1I	STATE 81	^1I 47.7%; ^3P 35.5%; ^1D 13.8%; ^3H 1.2%; omitted 1.5%
83	25353.25	^1I	STATE 82	^1I 45.0%; ^3P 29.3%; ^1D 22.7%; ^3H 1.6%; omitted 1.2%
84	25498.18	^3P	STATE 83	^3P 52.8%; ^1I 24.6%; ^1D 19.8%; ^3F 1.3%; omitted 1.1%
85	25748.63	^1I	STATE 84	^1I 34.4%; ^3P 33.4%; ^1D 29.2%; ^3H 1.0%; omitted 1.3%
86	27110.61	^1I	STATE 85	^1I 44.9%; ^3P 41.4%; ^1D 10.6%; ^3F 1.4%; omitted 1.1%
87	27118.65	^1I	STATE 86	^1I 45.8%; ^3P 40.6%; ^1D 10.4%; ^3F 1.5%; omitted 1.3%

88	27660.86	^{1}I	STATE 87	^{1}I 77.7%; ^{3}P 15.5%; ^{1}D 3.7%; ^{3}H 1.2%; omitted 1.3%
89	28750.58	^{1}I	STATE 88	^{1}I 60.7%; ^{3}P 28.1%; ^{1}D 8.7%; omitted 1.6%
90	28860.81	^{1}I	STATE 89	^{1}I 59.2%; ^{3}P 29.4%; ^{1}D 8.6%; omitted 1.9%
91	46191.07	^{1}S	STATE 90	^{1}S 93.6%; ^{3}P 6.3%

[$NpCl_4$(H-phthal)$_2$]

Table S38. 100 lowest calculated energies (cm^{-1}) of the electronic states in [$NpCl_4$(H-phthal)$_2$] with term symbol and parentage as determined from CASSCF-NEVPT2 calculations using ORCA.

Ion	Np(4+)	Configuration	$5f^3$	Energy source	NEVPT2

Section	Level	Energy / cm^{-1}	Label	Root(s)/state	Multiplicity/Parentage
Free ion spin-free	1	20.58	^{4}I	roots 0-12	4
	2	8230.33	^{4}F	roots 14-20	4
	3	8675.70	^{4}S	roots 13	4
	4	9539.78	^{2}H(1)	roots 0-10	2
	5	12307.93	^{2}G(1)	roots 11-19	2
	6	13297.38	^{4}G	roots 21-29	4
	7	14909.49	^{2}K	roots 20-34	2
	8	16674.80	^{2}D(1)	roots 35-39	2
	9	16907.50	^{2}P	roots 40-42	2
	10	21692.30	^{4}D	roots 30-34	4
	11	23206.96	^{2}I	roots 43-55	2
	12	23849.29	^{2}L	roots 56-72	2
	13	24826.98	^{2}D(2)	roots 84-88	2
	14	25142.19	^{2}H(2)	roots 73-83	2
	15	29845.04	^{2}F(1)	roots 89-95	2
	16	37727.71	^{2}G(2)	roots 96-104	2
	17	54220.54	^{2}F(2)	roots 105-111	2
Complex spin-free	1	0.00	^{4}I	roots 0-12	4
	2	8499.50	^{4}S	roots 13	4
	3	8529.50	^{4}F	roots 14-20	4
	4	10135.20	^{2}H(1)	roots 0-10	2
	5	11931.60	^{2}G(1)	roots 11-19	2
	6	12862.20	^{4}G	roots 21-29	4
	7	14885.10	^{2}K	roots 20-34	2
	8	17056.60	^{2}D(1)	roots 35-39	2
	9	17811.90	^{2}P	roots 40-42	2
	10	20563.70	^{4}D	roots 30-34	4
	11	20884.80	^{2}I	roots 43-55	2
	12	23505.10	^{2}L	roots 56-72	2
	13	24976.20	^{2}H(2)	roots 73-83	2
	14	26972.30	^{2}D(2)	roots 84-88	2
	15	29720.80	^{2}F(1)	roots 89-95	2
	16	36031.90	^{2}G(2)	roots 96-104	2
	17	49480.60	^{2}F(2)	roots 105-111	2
Complex SOC	1	0.00	^{4}I	STATE 0	^{4}I 84.8%; ^{2}H(1) 12.1%; ^{2}G(1) 1.5%; omitted 1.7%
	2	0.00	^{4}I	STATE 1	^{4}I 84.8%; ^{2}H(1) 12.1%; ^{2}G(1) 1.5%; omitted 1.7%

3	123.00	^4I	STATE 2	^4I 84.3%; $^2H(1)$ 12.6%; $^2G(1)$ 1.4%; omitted 1.8%
4	123.00	^4I	STATE 3	^4I 84.3%; $^2H(1)$ 12.6%; $^2G(1)$ 1.4%; omitted 1.8%
5	745.78	^4I	STATE 4	^4I 81.7%; $^2H(1)$ 15.3%; omitted 2.1%
6	745.78	^4I	STATE 5	^4I 81.7%; $^2H(1)$ 15.3%; omitted 2.1%
7	1541.15	^4I	STATE 6	^4I 81.5%; $^2H(1)$ 15.0%; omitted 2.8%
8	1541.15	^4I	STATE 7	^4I 81.5%; $^2H(1)$ 15.0%; omitted 2.8%
9	1721.32	^4I	STATE 8	^4I 81.1%; $^2H(1)$ 15.9%; omitted 2.2%
10	1721.32	^4I	STATE 9	^4I 81.1%; $^2H(1)$ 15.9%; omitted 2.2%
11	5803.81	^4I	STATE 10	^4I 93.1%; $^2H(1)$ 4.4%; omitted 2.5%
12	5803.81	^4I	STATE 11	^4I 93.1%; $^2H(1)$ 4.4%; omitted 2.5%
13	5864.84	^4I	STATE 12	^4I 94.1%; $^2H(1)$ 4.5%; omitted 1.5%
14	5864.84	^4I	STATE 13	^4I 94.1%; $^2H(1)$ 4.5%; omitted 1.5%
15	6255.98	^4I	STATE 14	^4I 93.3%; $^2H(1)$ 4.6%; omitted 2.1%
16	6255.98	^4I	STATE 15	^4I 93.3%; $^2H(1)$ 4.6%; omitted 2.1%
17	6883.48	^4I	STATE 16	^4I 93.4%; $^2H(1)$ 4.2%; omitted 1.7%
18	6883.48	^4I	STATE 17	^4I 93.4%; $^2H(1)$ 4.2%; omitted 1.7%
19	6993.47	^4I	STATE 18	^4I 93.9%; $^2H(1)$ 4.2%; omitted 1.4%
20	6993.47	^4I	STATE 19	^4I 93.9%; $^2H(1)$ 4.2%; omitted 1.4%
21	7079.83	^4I	STATE 20	^4I 93.9%; $^2H(1)$ 4.8%
22	7079.83	^4I	STATE 21	^4I 93.9%; $^2H(1)$ 4.8%
23	9942.38	^4F	STATE 22	^4F 49.0%; $^2D(1)$ 18.9%; ^4S 18.4%; ^4I 3.3%; omitted 4.4%
24	9942.38	^4F	STATE 23	^4F 49.0%; $^2D(1)$ 18.9%; ^4S 18.4%; ^4I 3.3%; omitted 4.4%
25	10219.01	^4F	STATE 24	^4F 49.3%; ^4I 20.1%; ^2P 10.2%; $^2D(1)$ 6.7%; omitted 4.1%
26	10219.01	^4F	STATE 25	^4F 49.3%; ^4I 20.1%; ^2P 10.2%; $^2D(1)$ 6.7%; omitted 4.1%
27	10450.81	^4I	STATE 26	^4I 87.1%; $^2G(1)$ 6.1%; ^2K 1.9%; ^4F 1.1%; omitted 3.1%
28	10450.81	^4I	STATE 27	^4I 87.1%; $^2G(1)$ 6.1%; ^2K 1.9%; ^4F 1.1%; omitted 3.1%
29	10528.90	^4I	STATE 28	^4I 69.4%; ^4F 14.3%; ^2P 3.0%; ^2K 2.8%; omitted 3.9%
30	10528.90	^4I	STATE 29	^4I 69.4%; ^4F 14.3%; ^2P 3.0%; ^2K 2.8%; omitted 3.9%
31	10921.02	^4I	STATE 30	^4I 87.4%; $^2G(1)$ 4.5%; ^2K 2.5%; omitted 3.6%
32	10921.02	^4I	STATE 31	^4I 87.4%; $^2G(1)$ 4.5%; ^2K 2.5%; omitted 3.6%
33	11655.55	^4I	STATE 32	^4I 89.2%; $^2G(1)$ 4.0%; ^2K 2.7%; omitted 3.6%
34	11655.55	^4I	STATE 33	^4I 89.2%; $^2G(1)$ 4.0%; ^2K 2.7%; omitted 3.6%
35	11716.99	^4I	STATE 34	^4I 90.5%; ^2K 2.9%; $^2G(1)$ 2.3%; omitted 3.6%

36	11716.99	^{4}I	STATE 35	^{4}I 90.5%; ^{2}K 2.9%; ^{2}G(1) 2.3%; omitted 3.6%
37	11867.99	^{4}I	STATE 36	^{4}I 90.5%; ^{2}K 4.2%; ^{2}G(1) 1.3%; omitted 3.4%
38	11867.99	^{4}I	STATE 37	^{4}I 90.5%; ^{2}K 4.2%; ^{2}G(1) 1.3%; omitted 3.4%
39	11937.55	^{4}I	STATE 38	^{4}I 90.0%; ^{2}K 3.9%; ^{2}G(1) 1.5%; omitted 3.2%
40	11937.55	^{4}I	STATE 39	^{4}I 90.0%; ^{2}K 3.9%; ^{2}G(1) 1.5%; omitted 3.2%
41	12527.11	^{4}F	STATE 40	^{4}F 25.8%; ^{2}H(1) 21.1%; ^{4}I 19.6%; ^{2}G(1) 18.4%; omitted 3.8%
42	12527.11	^{4}F	STATE 41	^{4}F 25.8%; ^{2}H(1) 21.1%; ^{4}I 19.6%; ^{2}G(1) 18.4%; omitted 3.8%
43	12596.45	^{4}I	STATE 42	^{4}I 24.7%; ^{2}H(1) 21.5%; ^{4}F 20.6%; ^{2}G(1) 19.3%; omitted 3.9%
44	12596.45	^{4}I	STATE 43	^{4}I 24.7%; ^{2}H(1) 21.5%; ^{4}F 20.6%; ^{2}G(1) 19.3%; omitted 3.9%
45	12797.89	^{2}H(1)	STATE 44	^{2}H(1) 33.2%; ^{2}G(1) 27.9%; ^{4}I 17.7%; ^{4}F 7.4%; omitted 3.8%
46	12797.89	^{2}H(1)	STATE 45	^{2}H(1) 33.2%; ^{2}G(1) 27.9%; ^{4}I 17.7%; ^{4}F 7.4%; omitted 3.8%
47	12936.00	^{2}H(1)	STATE 46	^{2}H(1) 34.2%; ^{2}G(1) 27.4%; ^{4}I 15.5%; ^{4}F 9.9%; omitted 4.4%
48	12936.00	^{2}H(1)	STATE 47	^{2}H(1) 34.2%; ^{2}G(1) 27.4%; ^{4}I 15.5%; ^{4}F 9.9%; omitted 4.4%
49	13028.02	^{2}G(1)	STATE 48	^{2}G(1) 29.4%; ^{2}H(1) 26.5%; ^{4}F 12.7%; ^{4}I 12.7%; omitted 4.4%
50	13028.02	^{2}G(1)	STATE 49	^{2}G(1) 29.4%; ^{2}H(1) 26.5%; ^{4}F 12.7%; ^{4}I 12.7%; omitted 4.4%
51	13623.68	^{4}F	STATE 50	^{4}F 47.9%; ^{4}S 23.2%; ^{4}G 7.5%; ^{2}D(1) 6.3%; omitted 3.3%
52	13623.68	^{4}F	STATE 51	^{4}F 47.9%; ^{4}S 23.2%; ^{4}G 7.5%; ^{2}D(1) 6.3%; omitted 3.3%
53	13776.94	^{4}F	STATE 52	^{4}F 43.6%; ^{4}G 13.1%; ^{2}H(1) 9.8%; ^{4}I 7.8%; omitted 4.2%
54	13776.94	^{4}F	STATE 53	^{4}F 43.6%; ^{4}G 13.1%; ^{2}H(1) 9.8%; ^{4}I 7.8%; omitted 4.2%
55	13843.61	^{4}F	STATE 54	^{4}F 47.4%; ^{4}G 13.0%; ^{2}H(1) 10.0%; ^{4}I 8.1%; omitted 3.5%
56	13843.61	^{4}F	STATE 55	^{4}F 47.4%; ^{4}G 13.0%; ^{2}H(1) 10.0%; ^{4}I 8.1%; omitted 3.5%
57	14260.78	^{4}F	STATE 56	^{4}F 47.7%; ^{4}G 33.3%; ^{2}G(1) 3.9%; ^{2}K 2.6%; omitted 4.2%
58	14260.78	^{4}F	STATE 57	^{4}F 47.7%; ^{4}G 33.3%; ^{2}G(1) 3.9%; ^{2}K 2.6%; omitted 4.2%
59	14737.03	^{4}I	STATE 58	^{4}I 69.8%; ^{2}K 10.4%; ^{4}F 6.9%; ^{2}H(1) 3.7%; omitted 3.4%
60	14737.03	^{4}I	STATE 59	^{4}I 69.8%; ^{2}K 10.4%; ^{4}F 6.9%; ^{2}H(1) 3.7%; omitted 3.4%

61	14819.90	^{4}I	STATE 60	^{4}I 58.8%; ^{4}F 12.8%; ^{2}K 11.9%; ^{2}H(1) 7.9%; omitted 4.8%
62	14819.90	^{4}I	STATE 61	^{4}I 58.8%; ^{4}F 12.8%; ^{2}K 11.9%; ^{2}H(1) 7.9%; omitted 4.8%
63	15041.69	^{4}I	STATE 62	^{4}I 38.2%; ^{4}F 27.6%; ^{2}G(1) 8.6%; ^{4}S 6.9%; omitted 4.3%
64	15041.69	^{4}I	STATE 63	^{4}I 38.2%; ^{4}F 27.6%; ^{2}G(1) 8.6%; ^{4}S 6.9%; omitted 4.3%
65	15202.27	^{4}F	STATE 64	^{4}F 65.5%; ^{2}D(1) 17.1%; ^{4}I 5.0%; ^{4}S 3.2%; omitted 4.2%
66	15202.27	^{4}F	STATE 65	^{4}F 65.5%; ^{2}D(1) 17.1%; ^{4}I 5.0%; ^{4}S 3.2%; omitted 4.2%
67	15414.74	^{4}F	STATE 66	^{4}F 57.0%; ^{4}I 18.6%; ^{2}P 9.5%; ^{2}D(1) 5.5%; omitted 4.8%
68	15414.74	^{4}F	STATE 67	^{4}F 57.0%; ^{4}I 18.6%; ^{2}P 9.5%; ^{2}D(1) 5.5%; omitted 4.8%
69	16004.86	^{4}G	STATE 68	^{4}G 36.9%; ^{4}F 24.2%; ^{2}G(1) 16.7%; ^{4}I 6.4%; omitted 4.7%
70	16004.86	^{4}G	STATE 69	^{4}G 36.9%; ^{4}F 24.2%; ^{2}G(1) 16.7%; ^{4}I 6.4%; omitted 4.7%
71	16059.12	^{4}F	STATE 70	^{4}F 28.7%; ^{4}I 19.1%; ^{2}G(1) 17.6%; ^{4}G 15.6%; omitted 4.3%
72	16059.12	^{4}F	STATE 71	^{4}F 28.7%; ^{4}I 19.1%; ^{2}G(1) 17.6%; ^{4}G 15.6%; omitted 4.3%
73	16112.83	^{4}G	STATE 72	^{4}G 39.7%; ^{4}F 26.8%; ^{2}G(1) 15.0%; ^{4}I 5.0%; omitted 4.3%
74	16112.83	^{4}G	STATE 73	^{4}G 39.7%; ^{4}F 26.8%; ^{2}G(1) 15.0%; ^{4}I 5.0%; omitted 4.3%
75	16219.10	^{4}I	STATE 74	^{4}I 56.5%; ^{4}F 13.9%; ^{2}K 10.6%; ^{2}G(1) 6.7%; omitted 5.0%
76	16219.10	^{4}I	STATE 75	^{4}I 56.5%; ^{4}F 13.9%; ^{2}K 10.6%; ^{2}G(1) 6.7%; omitted 5.0%
77	16291.95	^{4}I	STATE 76	^{4}I 67.2%; ^{2}K 11.1%; ^{4}F 6.9%; ^{4}G 3.6%; omitted 5.3%
78	16291.95	^{4}I	STATE 77	^{4}I 67.2%; ^{2}K 11.1%; ^{4}F 6.9%; ^{4}G 3.6%; omitted 5.3%
79	16353.65	^{4}I	STATE 78	^{4}I 36.4%; ^{4}F 26.1%; ^{2}G(1) 12.4%; ^{2}K 9.9%; omitted 3.4%
80	16353.65	^{4}I	STATE 79	^{4}I 36.4%; ^{4}F 26.1%; ^{2}G(1) 12.4%; ^{2}K 9.9%; omitted 3.4%
81	16391.71	^{4}I	STATE 80	^{4}I 65.2%; ^{2}K 12.2%; ^{4}F 8.7%; ^{2}G(1) 3.2%; omitted 5.6%
82	16391.71	^{4}I	STATE 81	^{4}I 65.2%; ^{2}K 12.2%; ^{4}F 8.7%; ^{2}G(1) 3.2%; omitted 5.6%
83	16560.95	^{4}I	STATE 82	^{4}I 71.7%; ^{2}K 13.6%; ^{4}G 3.4%; ^{4}F 3.1%; omitted 6.1%
84	16560.95	^{4}I	STATE 83	^{4}I 71.7%; ^{2}K 13.6%; ^{4}G 3.4%; ^{4}F 3.1%; omitted 6.1%
85	16637.61	^{4}I	STATE 84	^{4}I 54.6%; ^{4}G 11.4%; ^{2}K 11.3%; ^{4}F 10.6%; omitted 4.5%

86	16637.61	^4I	STATE 85	^4I 54.6%; ^4G 11.4%; ^2K 11.3%; ^4F 10.6%; omitted 4.5%
87	16732.71	^4G	STATE 86	^4G 37.9%; ^4I 28.3%; ^4F 9.6%; ^2K 5.4%; omitted 4.5%
88	16732.71	^4G	STATE 87	^4G 37.9%; ^4I 28.3%; ^4F 9.6%; ^2K 5.4%; omitted 4.5%
89	16776.34	^4G	STATE 88	^4G 41.8%; ^4F 22.1%; ^4I 14.1%; $^2G(1)$ 7.5%; omitted 4.6%
90	16776.34	^4G	STATE 89	^4G 41.8%; ^4F 22.1%; ^4I 14.1%; $^2G(1)$ 7.5%; omitted 4.6%
91	17963.06	^4G	STATE 90	^4G 57.2%; ^4F 23.3%; $^2G(1)$ 14.5%; $^2F(1)$ 1.3%; omitted 2.7%
92	17963.06	^4G	STATE 91	^4G 57.2%; ^4F 23.3%; $^2G(1)$ 14.5%; $^2F(1)$ 1.3%; omitted 2.7%
93	18549.66	^4G	STATE 92	^4G 49.8%; ^4F 23.0%; $^2H(1)$ 8.5%; $^2G(1)$ 7.5%; omitted 3.9%
94	18549.66	^4G	STATE 93	^4G 49.8%; ^4F 23.0%; $^2H(1)$ 8.5%; $^2G(1)$ 7.5%; omitted 3.9%
95	18689.87	^4G	STATE 94	^4G 49.4%; ^4F 19.8%; $^2H(1)$ 10.3%; $^2G(1)$ 8.4%; omitted 3.7%
96	18689.87	^4G	STATE 95	^4G 49.4%; ^4F 19.8%; $^2H(1)$ 10.3%; $^2G(1)$ 8.4%; omitted 3.7%
97	18926.44	^4G	STATE 96	^4G 68.8%; ^4F 12.3%; $^2G(1)$ 6.3%; $^2H(1)$ 3.1%; omitted 4.0%
98	18926.44	^4G	STATE 97	^4G 68.8%; ^4F 12.3%; $^2G(1)$ 6.3%; $^2H(1)$ 3.1%; omitted 4.0%
99	19374.77	^4F	STATE 98	^4F 41.6%; $^2H(1)$ 23.5%; ^4S 21.6%; ^4G 3.4%; omitted 5.5%
100	19374.77	^4F	STATE 99	^4F 41.6%; $^2H(1)$ 23.5%; ^4S 21.6%; ^4G 3.4%; omitted 5.5%

[$NpCl_4(HPyO)_2$]

Table S39. 100 lowest calculated energies (cm^{-1}) of the electronic states in [$NpCl_4(HPyO)_2$] with term symbol and parentage as determined from CASSCF-NEVPT2 calculations using ORCA.

Ion	Np(4+)	Configuration	$5f^3$	Energy source	NEVPT2

Section	Level	Energy / cm^{-1}	Label	Root(s)/state	Multiplicity/Parentage
Free ion spin-free	1	20.58	^4I	roots 0-12	4
	2	8230.33	^4F	roots 14-20	4
	3	8675.70	^4S	roots 13	4
	4	9539.78	$^2H(1)$	roots 0-10	2
	5	12307.93	$^2G(1)$	roots 11-19	2
	6	13297.38	^4G	roots 21-29	4
	7	14909.49	^2K	roots 20-34	2
	8	16674.80	$^2D(1)$	roots 35-39	2
	9	16907.50	^2P	roots 40-42	2
	10	21692.30	^4D	roots 30-34	4
	11	23206.96	^2I	roots 43-55	2
	12	23849.29	^2L	roots 56-72	2
	13	24826.98	$^2D(2)$	roots 84-88	2
	14	25142.19	$^2H(2)$	roots 73-83	2
	15	29845.04	$^2F(1)$	roots 89-95	2
	16	37727.71	$^2G(2)$	roots 96-104	2
	17	54220.54	$^2F(2)$	roots 105-111	2
Complex spin-free	1	0.00	^4I	roots 0-12	4
	2	8656.10	^4F	roots 14-20	4
	3	8677.60	^4S	roots 13	4
	4	10183.30	$^2H(1)$	roots 0-10	2
	5	11956.60	$^2G(1)$	roots 11-19	2
	6	13025.30	^4G	roots 21-29	4
	7	15076.40	^2K	roots 20-34	2
	8	17226.60	$^2D(1)$	roots 35-39	2
	9	17858.20	^2P	roots 40-42	2
	10	20670.90	^4D	roots 30-34	4
	11	20902.30	^2I	roots 43-55	2
	12	23695.50	^2L	roots 56-72	2
	13	25144.80	$^2H(2)$	roots 73-83	2
	14	27052.80	$^2D(2)$	roots 84-88	2
	15	29835.80	$^2F(1)$	roots 89-95	2
	16	36127.90	$^2G(2)$	roots 96-104	2
	17	49517.20	$^2F(2)$	roots 105-111	2
Complex SOC	1	0.00	^4I	STATE 0	^4I 84.8%; $^2H(1)$ 12.0%; $^2G(1)$ 1.5%; omitted 1.7%
	2	0.00	^4I	STATE 1	^4I 84.8%; $^2H(1)$ 12.0%; $^2G(1)$ 1.5%; omitted 1.7%

3	158.14	^{4}I	STATE 2	^{4}I 84.6%; ^{2}H(1) 12.6%; ^{2}G(1) 1.5%; omitted 1.3%
4	158.14	^{4}I	STATE 3	^{4}I 84.6%; ^{2}H(1) 12.6%; ^{2}G(1) 1.5%; omitted 1.3%
5	719.53	^{4}I	STATE 4	^{4}I 82.1%; ^{2}H(1) 15.5%; omitted 1.7%
6	719.53	^{4}I	STATE 5	^{4}I 82.1%; ^{2}H(1) 15.5%; omitted 1.7%
7	1534.73	^{4}I	STATE 6	^{4}I 81.8%; ^{2}H(1) 14.6%; omitted 2.6%
8	1534.73	^{4}I	STATE 7	^{4}I 81.8%; ^{2}H(1) 14.6%; omitted 2.6%
9	1812.48	^{4}I	STATE 8	^{4}I 81.1%; ^{2}H(1) 16.0%; ^{2}G(1) 1.0%; omitted 1.9%
10	1812.48	^{4}I	STATE 9	^{4}I 81.1%; ^{2}H(1) 16.0%; ^{2}G(1) 1.0%; omitted 1.9%
11	5847.52	^{4}I	STATE 10	^{4}I 93.1%; ^{2}H(1) 4.4%; omitted 2.5%
12	5847.52	^{4}I	STATE 11	^{4}I 93.1%; ^{2}H(1) 4.4%; omitted 2.5%
13	5935.45	^{4}I	STATE 12	^{4}I 94.2%; ^{2}H(1) 4.5%; omitted 1.3%
14	5935.45	^{4}I	STATE 13	^{4}I 94.2%; ^{2}H(1) 4.5%; omitted 1.3%
15	6162.17	^{4}I	STATE 14	^{4}I 93.6%; ^{2}H(1) 4.4%; omitted 2.0%
16	6162.17	^{4}I	STATE 15	^{4}I 93.6%; ^{2}H(1) 4.4%; omitted 2.0%
17	6887.51	^{4}I	STATE 16	^{4}I 94.0%; ^{2}H(1) 4.2%; omitted 1.2%
18	6887.51	^{4}I	STATE 17	^{4}I 94.0%; ^{2}H(1) 4.2%; omitted 1.2%
19	7036.65	^{4}I	STATE 18	^{4}I 93.9%; ^{2}H(1) 4.5%; omitted 1.1%
20	7036.65	^{4}I	STATE 19	^{4}I 93.9%; ^{2}H(1) 4.5%; omitted 1.1%
21	7140.36	^{4}I	STATE 20	^{4}I 93.7%; ^{2}H(1) 4.5%; omitted 1.2%
22	7140.36	^{4}I	STATE 21	^{4}I 93.7%; ^{2}H(1) 4.5%; omitted 1.2%
23	10017.55	^{4}F	STATE 22	^{4}F 50.8%; ^{2}D(1) 18.8%; ^{4}S 16.0%; ^{4}I 4.0%; omitted 5.1%
24	10017.55	^{4}F	STATE 23	^{4}F 50.8%; ^{2}D(1) 18.8%; ^{4}S 16.0%; ^{4}I 4.0%; omitted 5.1%
25	10218.79	^{4}F	STATE 24	^{4}F 48.1%; ^{4}I 20.4%; ^{2}P 10.4%; ^{2}D(1) 6.1%; omitted 4.0%
26	10218.79	^{4}F	STATE 25	^{4}F 48.1%; ^{4}I 20.4%; ^{2}P 10.4%; ^{2}D(1) 6.1%; omitted 4.0%
27	10511.81	^{4}I	STATE 26	^{4}I 87.1%; ^{2}G(1) 6.1%; ^{2}K 1.6%; omitted 4.5%
28	10511.81	^{4}I	STATE 27	^{4}I 87.1%; ^{2}G(1) 6.1%; ^{2}K 1.6%; omitted 4.5%
29	10592.15	^{4}I	STATE 28	^{4}I 69.4%; ^{4}F 14.1%; ^{2}P 3.0%; ^{2}G(1) 2.9%; omitted 3.5%
30	10592.15	^{4}I	STATE 29	^{4}I 69.4%; ^{4}F 14.1%; ^{2}P 3.0%; ^{2}G(1) 2.9%; omitted 3.5%
31	10799.84	^{4}I	STATE 30	^{4}I 87.4%; ^{2}G(1) 4.4%; ^{2}K 2.4%; ^{4}F 1.3%; omitted 3.5%
32	10799.84	^{4}I	STATE 31	^{4}I 87.4%; ^{2}G(1) 4.4%; ^{2}K 2.4%; ^{4}F 1.3%; omitted 3.5%
33	11655.61	^{4}I	STATE 32	^{4}I 89.2%; ^{2}G(1) 3.6%; ^{2}K 2.8%; omitted 3.8%
34	11655.61	^{4}I	STATE 33	^{4}I 89.2%; ^{2}G(1) 3.6%; ^{2}K 2.8%; omitted 3.8%
35	11718.50	^{4}I	STATE 34	^{4}I 89.9%; ^{2}K 3.3%; ^{2}G(1) 2.5%; omitted 3.7%

36	11718.50	^{4}I	STATE 35	^{4}I 89.9%; ^{2}K 3.3%; ^{2}G(1) 2.5%; omitted 3.7%
37	11919.22	^{4}I	STATE 36	^{4}I 89.9%; ^{2}K 3.4%; ^{2}G(1) 1.3%; omitted 4.2%
38	11919.22	^{4}I	STATE 37	^{4}I 89.9%; ^{2}K 3.4%; ^{2}G(1) 1.3%; omitted 4.2%
39	12012.51	^{4}I	STATE 38	^{4}I 89.4%; ^{2}K 4.1%; ^{2}G(1) 1.9%; omitted 3.7%
40	12012.51	^{4}I	STATE 39	^{4}I 89.4%; ^{2}K 4.1%; ^{2}G(1) 1.9%; omitted 3.7%
41	12528.52	^{4}F	STATE 40	^{4}F 25.8%; ^{4}I 21.9%; ^{2}H(1) 21.7%; ^{2}G(1) 16.6%; omitted 3.8%
42	12528.52	^{4}F	STATE 41	^{4}F 25.8%; ^{4}I 21.9%; ^{2}H(1) 21.7%; ^{2}G(1) 16.6%; omitted 3.8%
43	12600.75	^{4}I	STATE 42	^{4}I 25.3%; ^{2}H(1) 21.5%; ^{4}F 19.7%; ^{2}G(1) 19.2%; omitted 4.3%
44	12600.75	^{4}I	STATE 43	^{4}I 25.3%; ^{2}H(1) 21.5%; ^{4}F 19.7%; ^{2}G(1) 19.2%; omitted 4.3%
45	12842.81	^{2}H(1)	STATE 44	^{2}H(1) 35.5%; ^{2}G(1) 27.8%; ^{4}I 15.3%; ^{4}F 7.4%; omitted 3.5%
46	12842.81	^{2}H(1)	STATE 45	^{2}H(1) 35.5%; ^{2}G(1) 27.8%; ^{4}I 15.3%; ^{4}F 7.4%; omitted 3.5%
47	12922.16	^{2}H(1)	STATE 46	^{2}H(1) 32.3%; ^{2}G(1) 26.9%; ^{4}I 16.4%; ^{4}F 10.5%; omitted 4.2%
48	12922.16	^{2}H(1)	STATE 47	^{2}H(1) 32.3%; ^{2}G(1) 26.9%; ^{4}I 16.4%; ^{4}F 10.5%; omitted 4.2%
49	13026.37	^{2}G(1)	STATE 48	^{2}G(1) 30.3%; ^{2}H(1) 26.5%; ^{4}I 13.6%; ^{4}F 12.9%; omitted 4.0%
50	13026.37	^{2}G(1)	STATE 49	^{2}G(1) 30.3%; ^{2}H(1) 26.5%; ^{4}I 13.6%; ^{4}F 12.9%; omitted 4.0%
51	13652.49	^{4}F	STATE 50	^{4}F 48.1%; ^{4}S 22.2%; ^{4}G 9.7%; ^{2}D(1) 5.9%; omitted 3.8%
52	13652.49	^{4}F	STATE 51	^{4}F 48.1%; ^{4}S 22.2%; ^{4}G 9.7%; ^{2}D(1) 5.9%; omitted 3.8%
53	13807.62	^{4}F	STATE 52	^{4}F 43.4%; ^{4}G 12.8%; ^{2}H(1) 9.2%; ^{4}I 7.9%; omitted 3.8%
54	13807.62	^{4}F	STATE 53	^{4}F 43.4%; ^{4}G 12.8%; ^{2}H(1) 9.2%; ^{4}I 7.9%; omitted 3.8%
55	13868.36	^{4}F	STATE 54	^{4}F 47.1%; ^{2}H(1) 11.6%; ^{4}G 11.2%; ^{4}I 8.4%; omitted 3.9%
56	13868.36	^{4}F	STATE 55	^{4}F 47.1%; ^{2}H(1) 11.6%; ^{4}G 11.2%; ^{4}I 8.4%; omitted 3.9%
57	14229.73	^{4}F	STATE 56	^{4}F 49.8%; ^{4}G 32.8%; ^{2}G(1) 3.7%; ^{2}K 2.3%; omitted 4.0%
58	14229.73	^{4}F	STATE 57	^{4}F 49.8%; ^{4}G 32.8%; ^{2}G(1) 3.7%; ^{2}K 2.3%; omitted 4.0%
59	14823.65	^{4}I	STATE 58	^{4}I 69.5%; ^{2}K 9.6%; ^{4}F 8.0%; ^{2}G(1) 4.2%; omitted 4.0%
60	14823.65	^{4}I	STATE 59	^{4}I 69.5%; ^{2}K 9.6%; ^{4}F 8.0%; ^{2}G(1) 4.2%; omitted 4.0%

61	14883.93	^{4}I	STATE 60	^{4}I 55.7%; ^{4}F 14.5%; ^{2}K 11.4%; ^{2}H(1) 7.6%; omitted 4.3%
62	14883.93	^{4}I	STATE 61	^{4}I 55.7%; ^{4}F 14.5%; ^{2}K 11.4%; ^{2}H(1) 7.6%; omitted 4.3%
63	14994.96	^{4}I	STATE 62	^{4}I 52.9%; ^{4}F 16.7%; ^{2}K 8.6%; ^{2}H(1) 5.5%; omitted 3.5%
64	14994.96	^{4}I	STATE 63	^{4}I 52.9%; ^{4}F 16.7%; ^{2}K 8.6%; ^{2}H(1) 5.5%; omitted 3.5%
65	15316.90	^{4}F	STATE 64	^{4}F 62.9%; ^{2}D(1) 17.5%; ^{4}I 7.2%; ^{4}S 2.9%; omitted 4.1%
66	15316.90	^{4}F	STATE 65	^{4}F 62.9%; ^{2}D(1) 17.5%; ^{4}I 7.2%; ^{4}S 2.9%; omitted 4.1%
67	15441.77	^{4}F	STATE 66	^{4}F 62.6%; ^{2}P 10.7%; ^{4}I 10.3%; ^{2}D(1) 6.5%; omitted 4.0%
68	15441.77	^{4}F	STATE 67	^{4}F 62.6%; ^{2}P 10.7%; ^{4}I 10.3%; ^{2}D(1) 6.5%; omitted 4.0%
69	16066.90	^{4}G	STATE 68	^{4}G 31.7%; ^{4}F 27.2%; ^{2}G(1) 19.4%; ^{4}I 6.4%; omitted 4.5%
70	16066.90	^{4}G	STATE 69	^{4}G 31.7%; ^{4}F 27.2%; ^{2}G(1) 19.4%; ^{4}I 6.4%; omitted 4.5%
71	16107.83	^{4}F	STATE 70	^{4}F 30.4%; ^{2}G(1) 18.2%; ^{4}G 17.6%; ^{4}I 15.4%; omitted 4.4%
72	16107.83	^{4}F	STATE 71	^{4}F 30.4%; ^{2}G(1) 18.2%; ^{4}G 17.6%; ^{4}I 15.4%; omitted 4.4%
73	16153.63	^{4}G	STATE 72	^{4}G 35.6%; ^{4}F 27.4%; ^{2}G(1) 12.9%; ^{4}I 9.7%; omitted 4.8%
74	16153.63	^{4}G	STATE 73	^{4}G 35.6%; ^{4}F 27.4%; ^{2}G(1) 12.9%; ^{4}I 9.7%; omitted 4.8%
75	16234.70	^{4}I	STATE 74	^{4}I 56.9%; ^{4}F 14.3%; ^{2}K 10.9%; ^{2}G(1) 6.0%; omitted 4.4%
76	16234.70	^{4}I	STATE 75	^{4}I 56.9%; ^{4}F 14.3%; ^{2}K 10.9%; ^{2}G(1) 6.0%; omitted 4.4%
77	16252.01	^{4}I	STATE 76	^{4}I 53.4%; ^{4}G 12.3%; ^{4}F 10.6%; ^{2}K 8.9%; omitted 4.3%
78	16252.01	^{4}I	STATE 77	^{4}I 53.4%; ^{4}G 12.3%; ^{4}F 10.6%; ^{2}K 8.9%; omitted 4.3%
79	16376.42	^{4}I	STATE 78	^{4}I 56.1%; ^{4}F 13.6%; ^{2}K 10.9%; ^{2}G(1) 5.7%; omitted 4.1%
80	16376.42	^{4}I	STATE 79	^{4}I 56.1%; ^{4}F 13.6%; ^{2}K 10.9%; ^{2}G(1) 5.7%; omitted 4.1%
81	16411.17	^{4}I	STATE 80	^{4}I 50.2%; ^{4}F 16.5%; ^{2}K 11.1%; ^{2}G(1) 7.8%; omitted 4.5%
82	16411.17	^{4}I	STATE 81	^{4}I 50.2%; ^{4}F 16.5%; ^{2}K 11.1%; ^{2}G(1) 7.8%; omitted 4.5%
83	16623.75	^{4}I	STATE 82	^{4}I 64.3%; ^{2}K 13.0%; ^{4}G 8.8%; ^{4}F 4.2%; omitted 5.5%
84	16623.75	^{4}I	STATE 83	^{4}I 64.3%; ^{2}K 13.0%; ^{4}G 8.8%; ^{4}F 4.2%; omitted 5.5%

85	16690.09	^{4}I	STATE 84	^{4}I 32.8%; ^{4}G 27.0%; ^{4}F 13.5%; ^{2}K 6.6%; omitted 5.2%
86	16690.09	^{4}I	STATE 85	^{4}I 32.8%; ^{4}G 27.0%; ^{4}F 13.5%; ^{2}K 6.6%; omitted 5.2%
87	16792.45	^{4}G	STATE 86	^{4}G 44.5%; ^{4}F 20.4%; ^{4}I 13.1%; ^{2}G(1) 5.9%; omitted 4.4%
88	16792.45	^{4}G	STATE 87	^{4}G 44.5%; ^{4}F 20.4%; ^{4}I 13.1%; ^{2}G(1) 5.9%; omitted 4.4%
89	16862.03	^{4}I	STATE 88	^{4}I 60.1%; ^{4}G 11.5%; ^{2}K 11.3%; ^{4}F 5.3%; omitted 4.9%
90	16862.03	^{4}I	STATE 89	^{4}I 60.1%; ^{4}G 11.5%; ^{2}K 11.3%; ^{4}F 5.3%; omitted 4.9%
91	17978.04	^{4}G	STATE 90	^{4}G 57.1%; ^{4}F 24.0%; ^{2}G(1) 14.5%; ^{2}F(1) 1.4%; omitted 2.6%
92	17978.04	^{4}G	STATE 91	^{4}G 57.1%; ^{4}F 24.0%; ^{2}G(1) 14.5%; ^{2}F(1) 1.4%; omitted 2.6%
93	18616.48	^{4}G	STATE 92	^{4}G 48.5%; ^{4}F 23.2%; ^{2}H(1) 9.1%; ^{2}G(1) 8.3%; omitted 3.4%
94	18616.48	^{4}G	STATE 93	^{4}G 48.5%; ^{4}F 23.2%; ^{2}H(1) 9.1%; ^{2}G(1) 8.3%; omitted 3.4%
95	18693.92	^{4}G	STATE 94	^{4}G 47.6%; ^{4}F 21.7%; ^{2}H(1) 11.1%; ^{2}G(1) 7.7%; omitted 3.5%
96	18693.92	^{4}G	STATE 95	^{4}G 47.6%; ^{4}F 21.7%; ^{2}H(1) 11.1%; ^{2}G(1) 7.7%; omitted 3.5%
97	18942.52	^{4}G	STATE 96	^{4}G 68.7%; ^{4}F 13.2%; ^{2}G(1) 5.9%; ^{2}F(1) 2.8%; omitted 4.0%
98	18942.52	^{4}G	STATE 97	^{4}G 68.7%; ^{4}F 13.2%; ^{2}G(1) 5.9%; ^{2}F(1) 2.8%; omitted 4.0%
99	19373.71	^{4}F	STATE 98	^{4}F 44.2%; ^{2}H(1) 24.1%; ^{4}S 18.8%; ^{4}G 3.4%; omitted 5.0%
100	19373.71	^{4}F	STATE 99	^{4}F 44.2%; ^{2}H(1) 24.1%; ^{4}S 18.8%; ^{4}G 3.4%; omitted 5.0%

$[PuCl_4(H\text{-}phthal)_2]$

Table S40. 100 lowest calculated energies (cm^{-1}) of the electronic states in $[PuCl_4(H\text{-}phthal)_2]$ with term symbol and parentage as determined from CASSCF-NEVPT2 calculations using ORCA.

Ion	Pu(4+)	Configuration	$5f^4$	Energy source	NEVPT2

Section	Level	Energy / cm^{-1}	Label	Root(s)/state	Multiplicity/Parentage
Free ion spin-free	1	24.70	^5I	roots 0-12	5
	2	8090.50	^5S	roots 17	5
	3	8875.89	^5F	roots 13-16, 18-20	5
	4	13376.52	$^3K(1)$	roots 0-14	3
	5	13944.33	^5G	roots 21-29	5
	6	15739.86	$^3H(1)$	roots 15-25	3
	7	16853.24	$^3G(1)$	roots 26-34	3
	8	18362.92	$^3D(1)$	roots 52, 54, 57-59	3
	9	18365.10	$^3P(1)$	roots 53, 55-56	3
	10	18709.29	^3L	roots 35-51	3
	11	20628.47	$^3F(1)$	roots 79-85	3
	12	21746.56	$^1L(1)$	roots 0-16	1
	13	22365.81	^3M	roots 60-78	3
	14	22436.00	$^1D(1)$	roots 17-21	1
	15	22565.90	$^3I(1)$	roots 86-98	3
	16	22886.30	^5D	roots 30-34	5
	17	24022.37	$^1G(1)$	roots 22-30	1
	18	24445.69	$^3F(2)$	roots 99-105	3
	19	28011.91	$^1H(1)$	roots 44-54	1
	20	28273.20	$^1D(2)$	roots 70-74	1
	21	28907.15	$^1I(1)$	roots 31-43	1
	22	29230.05	$^3H(2)$	roots 106-116	3
	23	29716.95	^1K	roots 55-69	1
	24	31656.41	$^3G(2)$	roots 117-125	3
	25	33302.73	$^3F(3)$	roots 126-132	3
	26	35598.08	$^3K(2)$	roots 141-143, 146, 148-158	3
	27	35639.33	$^3H(3)$	roots 133-140, 144-145, 147	3
	28	35806.61	^1N	roots 77, 80-85, 87-89, 92, 95-104	1
	29	35875.03	$^1G(2)$	roots 75-76, 78-79, 86, 90-91, 93-94	1
	30	39824.06	$^1L(2)$	roots 105-121	1
	31	40171.39	$^3I(2)$	roots 159-171	3
	32	40861.63	$^3P(2)$	roots 172-174	3
	33	42365.49	$^3G(3)$	roots 175-183	3
	34	44228.05	$^1I(2)$	roots 122-134	1

	35	45742.64	^{1}G(3)	roots 135-143	1
	36	47305.10	^{1}F	roots 144-150	1
	37	47596.38	^{1}D(3)	roots 151-155	1
	38	47780.18	^{3}D(2)	roots 184-188	3
	39	54355.60	^{1}S(1)	roots 156	1
	40	55525.91	^{1}G(4)	roots 157-161, 163, 165-167	1
	41	55585.00	^{1}S(2)	roots 162	1
	42	61640.38	^{1}I(3)	roots 164, 168-175, 177-180	1
	43	57018.37	^{3}F(4)	roots 189-195	3
	44	60296.55	^{3}H(4)	roots 196-206	3
	45	60960.00	^{3}P(3)	roots 207-209	3
	46	72348.65	^{1}H(2)	roots 176, 181-190	1
	47	92320.56	^{1}D(4)	roots 191-195	1
Complex spin-free	1	0.00	^{5}I	roots 0-12	5
	2	7852.40	^{5}F	roots 13-16, 18-20	5
	3	8904.70	^{5}S	roots 17	5
	4	12966.90	^{3}K(1)	roots 0-14	3
	5	13245.30	^{5}G	roots 21-29	5
	6	14428.60	^{3}H(1)	roots 15-25	3
	7	15330.20	^{3}G(1)	roots 26-34	3
	8	17748.30	^{3}L	roots 35-51	3
	9	18170.30	^{3}P(1)	roots 53, 55-56	3
	10	18525.30	^{3}D(1)	roots 52, 54, 57-59	3
	11	19236.50	^{3}M	roots 60-78	3
	12	19412.40	^{5}D	roots 30-34	5
	13	20278.00	^{1}L(1)	roots 0-16	1
	14	21365.20	^{1}D(1)	roots 17-21	1
	15	21666.40	^{1}G(1)	roots 22-30	1
	16	22483.50	^{3}F(1)	roots 79-85	3
	17	22940.30	^{3}I(1)	roots 86-98	3
	18	23665.00	^{3}F(2)	roots 99-105	3
	19	25432.20	^{1}I(1)	roots 31-43	1
	20	26127.00	^{1}H(1)	roots 44-54	1
	21	27664.60	^{3}H(2)	roots 106-116	3
	22	29080.40	^{1}K	roots 55-69	1
	23	29738.30	^{3}G(2)	roots 117-125	3
	24	30354.60	^{1}D(2)	roots 70-74	1
	25	31391.30	^{3}F(3)	roots 126-132	3
	26	32224.30	^{1}G(2)	roots 75-76, 78-79, 86, 90-91, 93-94	1
	27	32408.90	^{3}H(3)	roots 133-140, 144-145, 147	3

	28	32510.50	^{1}N	roots 77, 80-85, 87-89, 92, 95-104	1
	29	33897.30	^{3}K(2)	roots 141-143, 146, 148-158	3
	30	36038.10	^{3}I(2)	roots 159-171	3
	31	36795.50	^{1}L(2)	roots 105-121	1
	32	38621.10	^{3}P(2)	roots 172-174	3
	33	39569.80	^{3}G(3)	roots 175-183	3
	34	41173.20	^{1}I(2)	roots 122-134	1
	35	42380.90	^{1}G(3)	roots 135-143	1
	36	43884.10	^{1}F	roots 144-150	1
	37	44472.20	^{3}D(2)	roots 184-188	3
	38	45872.10	^{1}D(3)	roots 151-155	1
	39	50661.80	^{1}S(1)	roots 156	1
	40	51135.40	^{1}G(4)	roots 157-161, 163, 165-167	1
	41	52278.60	^{3}F(4)	roots 189-195	3
	42	52820.60	^{1}S(2)	roots 162	1
	43	53009.70	^{1}I(3)	roots 164, 168-175, 177-180	1
	44	54411.10	^{3}H(4)	roots 196-206	3
	45	57573.10	^{3}P(3)	roots 207-209	3
	46	59153.70	^{1}H(2)	roots 176, 181-190	1
	47	81236.40	^{1}D(4)	roots 191-195	1
Complex SOC	1	0.00	^{5}I	STATE 0	^{5}I 79.4%; ^{3}H(1) 15.8%; omitted 3.1%
	2	115.99	^{5}I	STATE 1	^{5}I 79.0%; ^{3}H(1) 16.5%; omitted 3.4%
	3	214.92	^{5}I	STATE 2	^{5}I 78.6%; ^{3}H(1) 17.1%; omitted 3.3%
	4	1239.52	^{5}I	STATE 3	^{5}I 80.0%; ^{3}G(1) 8.6%; ^{3}H(1) 5.6%; omitted 4.9%
	5	1300.00	^{5}I	STATE 4	^{5}I 81.3%; ^{3}G(1) 8.7%; ^{3}H(1) 4.0%; omitted 4.8%
	6	1498.85	^{5}I	STATE 5	^{5}I 81.0%; ^{3}G(1) 7.7%; ^{3}H(1) 5.9%; omitted 4.8%
	7	1581.24	^{5}I	STATE 6	^{5}I 81.7%; ^{3}G(1) 7.4%; ^{3}H(1) 3.8%; omitted 5.5%
	8	1639.78	^{5}I	STATE 7	^{5}I 81.7%; ^{3}G(1) 8.2%; ^{3}H(1) 3.8%; omitted 5.0%
	9	1698.86	^{5}I	STATE 8	^{5}I 82.6%; ^{3}G(1) 8.8%; ^{3}L 1.5%; omitted 4.6%
	10	5447.58	^{5}I	STATE 9	^{5}I 89.4%; ^{3}H(1) 5.3%; ^{3}G(1) 2.4%; omitted 3.0%
	11	5470.58	^{5}I	STATE 10	^{5}I 88.4%; ^{3}G(1) 4.5%; ^{3}H(1) 3.6%; omitted 3.2%
	12	5564.49	^{5}I	STATE 11	^{5}I 88.4%; ^{3}G(1) 4.3%; ^{3}H(1) 3.7%; omitted 3.4%
	13	5608.89	^{5}I	STATE 12	^{5}I 89.2%; ^{3}H(1) 4.2%; ^{3}G(1) 3.1%; omitted 3.3%

14	5640.59	^5I	STATE 13	^5I 88.8%; $^3H(1)$ 4.1%; $^3G(1)$ 3.1%; omitted 3.7%
15	5736.87	^5I	STATE 14	^5I 90.1%; $^3G(1)$ 3.7%; $^3H(1)$ 3.2%; omitted 3.0%
16	5772.37	^5I	STATE 15	^5I 90.1%; $^3G(1)$ 4.1%; $^3H(1)$ 2.3%; omitted 3.4%
17	5941.49	^5I	STATE 16	^5I 90.1%; $^3G(1)$ 3.7%; $^3H(1)$ 2.6%; omitted 3.6%
18	6184.60	^5I	STATE 17	^5I 90.4%; $^3G(1)$ 4.2%; omitted 4.3%
19	6202.10	^5I	STATE 18	^5I 90.4%; $^3G(1)$ 4.5%; $^3H(1)$ 1.1%; omitted 3.6%
20	6237.13	^5I	STATE 19	^5I 90.5%; $^3G(1)$ 4.4%; $^3H(1)$ 1.0%; omitted 3.9%
21	9604.46	^5I	STATE 20	^5I 89.0%; $^3K(1)$ 4.4%; $^3H(1)$ 1.1%; omitted 4.5%
22	9612.35	^5I	STATE 21	^5I 88.6%; $^3K(1)$ 4.3%; $^3H(1)$ 1.0%; omitted 5.2%
23	9684.33	^5I	STATE 22	^5I 87.8%; $^3K(1)$ 4.2%; omitted 6.2%
24	9730.71	^5I	STATE 23	^5I 87.9%; $^3K(1)$ 4.9%; $^3G(1)$ 1.3%; omitted 4.6%
25	9747.85	^5I	STATE 24	^5I 87.7%; $^3K(1)$ 4.9%; $^3H(1)$ 1.3%; omitted 4.9%
26	9764.23	^5I	STATE 25	^5I 69.0%; ^5F 4.8%; $^3K(1)$ 4.4%; ^3L 4.1%; omitted 6.4%
27	9785.89	^5I	STATE 26	^5I 78.7%; $^3K(1)$ 4.8%; ^5F 2.8%; $^3G(1)$ 2.1%; omitted 6.9%
28	9803.33	^5I	STATE 27	^5I 82.8%; $^3K(1)$ 5.1%; ^3L 1.3%; $^3G(1)$ 1.2%; omitted 6.2%
29	9824.41	^5I	STATE 28	^5I 85.7%; $^3K(1)$ 5.0%; $^3G(1)$ 1.1%; omitted 6.3%
30	10029.63	^5I	STATE 29	^5I 84.9%; $^3K(1)$ 5.4%; $^3G(1)$ 1.5%; omitted 6.4%
31	10041.37	^5I	STATE 30	^5I 85.8%; $^3K(1)$ 5.8%; omitted 6.5%
32	10109.55	^5I	STATE 31	^5I 84.7%; $^3K(1)$ 6.4%; $^3G(1)$ 1.4%; omitted 6.4%
33	10135.65	^5I	STATE 32	^5I 84.5%; $^3K(1)$ 6.2%; $^3G(1)$ 1.3%; omitted 6.3%
34	10668.31	^5I	STATE 33	^5I 28.3%; ^5F 22.1%; ^3L 11.8%; $^3F(2)$ 8.4%; omitted 7.2%
35	11089.78	^5F	STATE 34	^5F 36.6%; ^5I 13.7%; $^3F(2)$ 9.8%; $^3D(1)$ 9.3%; omitted 7.3%
36	11920.06	^5F	STATE 35	^5F 37.1%; $^3F(2)$ 16.0%; ^5G 11.0%; $^3D(1)$ 6.8%; omitted 8.5%
37	12031.95	^5F	STATE 36	^5F 36.5%; $^3F(2)$ 16.4%; ^5G 11.4%; $^3P(1)$ 6.1%; omitted 8.1%
38	12483.16	^5S	STATE 37	^5S 19.6%; ^5F 16.8%; $^3F(2)$ 13.3%; ^5G 10.2%; omitted 6.8%
39	12850.79	^5I	STATE 38	^5I 71.4%; $^3K(1)$ 16.5%; ^5F 1.7%; ^3L 1.0%; omitted 5.9%

40	12872.71	^{5}I	STATE 39	^{5}I 40.2%; ^{5}F 28.6%; ^{5}S 8.8%; ^{3}K(1) 8.4%; omitted 5.3%
41	12901.06	^{5}I	STATE 40	^{5}I 53.4%; ^{5}F 20.8%; ^{3}K(1) 11.3%; ^{5}S 2.8%; omitted 5.3%
42	12925.43	^{5}I	STATE 41	^{5}I 60.0%; ^{5}F 14.3%; ^{3}K(1) 13.1%; ^{5}S 1.9%; omitted 6.3%
43	12944.74	^{5}I	STATE 42	^{5}I 66.6%; ^{3}K(1) 16.9%; ^{5}F 5.0%; ^{3}L 1.1%; omitted 6.3%
44	12985.52	^{5}I	STATE 43	^{5}I 63.5%; ^{3}K(1) 17.6%; ^{3}H(1) 4.6%; ^{5}F 1.7%; omitted 7.1%
45	13032.42	^{5}I	STATE 44	^{5}I 61.5%; ^{3}K(1) 17.1%; ^{5}F 5.4%; ^{3}H(1) 3.1%; omitted 7.0%
46	13046.35	^{5}I	STATE 45	^{5}I 56.4%; ^{3}K(1) 17.9%; ^{3}H(1) 6.2%; ^{5}F 4.1%; omitted 7.8%
47	13124.24	^{5}I	STATE 46	^{5}I 48.0%; ^{5}F 21.0%; ^{3}K(1) 11.9%; ^{5}S 5.7%; omitted 6.5%
48	13157.00	^{5}I	STATE 47	^{5}I 55.7%; ^{5}F 17.0%; ^{3}K(1) 13.4%; ^{3}L 2.1%; omitted 6.3%
49	13183.68	^{5}I	STATE 48	^{5}I 55.4%; ^{5}F 15.6%; ^{3}K(1) 13.2%; ^{5}S 2.7%; omitted 7.1%
50	13236.33	^{5}I	STATE 49	^{5}I 66.6%; ^{3}K(1) 18.0%; ^{5}F 5.0%; ^{3}L 1.3%; omitted 6.0%
51	13391.24	^{5}I	STATE 50	^{5}I 47.7%; ^{5}F 21.0%; ^{3}K(1) 11.6%; ^{5}S 3.4%; omitted 6.9%
52	13416.25	^{5}I	STATE 51	^{5}I 50.7%; ^{5}F 19.3%; ^{3}K(1) 12.5%; ^{3}H(1) 3.2%; omitted 7.3%
53	13433.53	^{5}I	STATE 52	^{5}I 54.1%; ^{3}K(1) 13.9%; ^{5}F 11.1%; ^{3}H(1) 4.4%; omitted 8.7%
54	13522.93	^{5}I	STATE 53	^{5}I 48.7%; ^{3}K(1) 12.7%; ^{5}F 9.4%; ^{3}H(1) 7.2%; omitted 7.8%
55	13551.35	^{5}I	STATE 54	^{5}I 49.9%; ^{5}F 14.1%; ^{3}K(1) 13.7%; ^{3}H(1) 5.1%; omitted 6.8%
56	13588.11	^{5}I	STATE 55	^{5}I 57.9%; ^{3}K(1) 15.3%; ^{3}H(1) 6.1%; ^{5}F 3.1%; omitted 8.7%
57	13754.37	^{5}I	STATE 56	^{5}I 39.8%; ^{3}H(1) 14.2%; ^{5}F 9.7%; ^{5}S 5.0%; omitted 8.8%
58	13781.86	^{5}I	STATE 57	^{5}I 33.2%; ^{3}H(1) 17.3%; ^{5}F 10.4%; ^{5}S 5.9%; omitted 7.9%
59	13954.88	^{5}I	STATE 58	^{5}I 22.5%; ^{3}H(1) 18.8%; ^{5}F 15.5%; ^{3}G(1) 6.0%; omitted 8.6%
60	14503.96	^{5}I	STATE 59	^{5}I 18.2%; ^{3}G(1) 13.5%; ^{3}H(1) 12.0%; ^{5}F 11.3%; omitted 10.1%
61	14780.50	^{5}F	STATE 60	^{5}F 23.2%; ^{5}I 15.1%; ^{3}G(1) 11.1%; ^{3}L 10.1%; omitted 9.2%
62	14875.66	^{5}I	STATE 61	^{5}I 15.9%; ^{3}G(1) 12.6%; ^{5}F 10.5%; ^{3}H(1) 9.4%; omitted 10.8%
63	15047.52	^{5}F	STATE 62	^{5}F 35.4%; ^{5}G 14.6%; ^{5}I 12.1%; ^{3}K(1) 5.4%; omitted 8.7%

64	15058.79	^{5}F	STATE 63	^{5}F 38.5%; ^{5}G 14.3%; ^{5}I 11.7%; ^{3}K(1) 5.0%; omitted 8.2%
65	15132.10	^{5}F	STATE 64	^{5}F 35.2%; ^{5}I 15.8%; ^{5}G 10.9%; ^{3}K(1) 6.4%; omitted 9.8%
66	15185.16	^{5}F	STATE 65	^{5}F 24.0%; ^{5}I 16.8%; ^{5}G 8.1%; ^{3}L 8.1%; omitted 11.1%
67	15294.68	^{5}F	STATE 66	^{5}F 22.7%; ^{5}I 20.6%; ^{3}K(1) 9.6%; ^{3}L 7.0%; omitted 9.9%
68	15433.94	^{5}I	STATE 67	^{5}I 28.6%; ^{5}F 17.9%; ^{3}K(1) 13.7%; ^{3}L 7.3%; omitted 8.8%
69	15441.16	^{5}F	STATE 68	^{5}F 39.9%; ^{5}I 17.4%; ^{5}S 7.7%; ^{3}K(1) 7.0%; omitted 8.9%
70	15517.85	^{5}I	STATE 69	^{5}I 43.2%; ^{3}K(1) 21.0%; ^{5}F 12.5%; ^{1}L(1) 3.4%; omitted 9.6%
71	15519.22	^{5}I	STATE 70	^{5}I 34.4%; ^{5}F 25.7%; ^{3}K(1) 16.4%; ^{3}H(1) 3.9%; omitted 7.6%
72	15548.27	^{5}F	STATE 71	^{5}F 29.9%; ^{5}I 29.7%; ^{3}K(1) 13.9%; ^{5}G 3.6%; omitted 10.3%
73	15550.62	^{5}I	STATE 72	^{5}I 35.1%; ^{5}F 24.1%; ^{3}K(1) 16.8%; ^{1}L(1) 2.7%; omitted 9.0%
74	15586.99	^{5}I	STATE 73	^{5}I 33.1%; ^{5}F 17.3%; ^{3}K(1) 15.8%; ^{5}S 4.9%; omitted 10.3%
75	15613.78	^{5}I	STATE 74	^{5}I 29.3%; ^{5}F 28.3%; ^{3}K(1) 11.9%; ^{5}S 5.4%; omitted 9.7%
76	15630.85	^{5}F	STATE 75	^{5}F 33.1%; ^{5}I 26.0%; ^{3}K(1) 10.4%; ^{5}S 4.9%; omitted 8.3%
77	15744.12	^{5}I	STATE 76	^{5}I 32.8%; ^{5}F 17.9%; ^{3}K(1) 14.2%; ^{5}G 8.1%; omitted 9.7%
78	15767.65	^{5}I	STATE 77	^{5}I 33.2%; ^{5}F 19.1%; ^{3}K(1) 14.9%; ^{5}G 5.1%; omitted 10.1%
79	15842.34	^{5}I	STATE 78	^{5}I 33.3%; ^{5}F 23.4%; ^{3}K(1) 16.2%; ^{5}G 3.9%; omitted 8.9%
80	15874.77	^{5}I	STATE 79	^{5}I 35.1%; ^{5}F 16.3%; ^{3}K(1) 15.0%; ^{5}G 8.1%; omitted 8.7%
81	15961.71	^{5}I	STATE 80	^{5}I 35.3%; ^{5}F 21.8%; ^{3}K(1) 17.7%; ^{3}L 6.7%; omitted 8.1%
82	15997.24	^{5}F	STATE 81	^{5}F 38.1%; ^{5}G 16.3%; ^{5}I 11.6%; ^{5}S 5.9%; omitted 9.3%
83	16122.90	^{5}I	STATE 82	^{5}I 25.6%; ^{5}F 20.5%; ^{3}K(1) 12.7%; ^{5}S 6.6%; omitted 9.2%
84	16149.00	^{5}I	STATE 83	^{5}I 32.0%; ^{5}F 16.9%; ^{3}K(1) 16.9%; ^{3}L 7.4%; omitted 9.0%
85	16151.43	^{5}F	STATE 84	^{5}F 28.5%; ^{3}H(1) 17.8%; ^{5}G 15.5%; ^{3}G(1) 10.2%; omitted 10.0%
86	16234.78	^{5}I	STATE 85	^{5}I 34.4%; ^{3}K(1) 19.5%; ^{5}F 18.3%; ^{3}L 4.2%; omitted 9.3%
87	16277.28	^{5}I	STATE 86	^{5}I 30.2%; ^{5}F 17.8%; ^{3}K(1) 16.5%; ^{5}S 9.0%; omitted 8.4%
88	16290.57	^{5}I	STATE 87	^{5}I 30.4%; ^{5}F 21.6%; ^{3}K(1) 17.2%; ^{5}S 4.3%; omitted 7.9%

89	16843.40	^{5}I	STATE 88	^{5}I 38.5%; ^{3}K(1) 19.0%; ^{5}F 11.7%; ^{5}G 5.6%; omitted 8.6%
90	16876.74	^{5}I	STATE 89	^{5}I 47.5%; ^{3}K(1) 23.3%; ^{3}L 5.2%; ^{5}G 3.3%; omitted 7.3%
91	16955.48	^{5}I	STATE 90	^{5}I 40.3%; ^{3}K(1) 23.7%; ^{3}L 5.3%; ^{5}G 4.6%; omitted 8.7%
92	16989.51	^{5}I	STATE 91	^{5}I 30.0%; ^{3}K(1) 16.7%; ^{5}G 12.1%; ^{5}F 11.8%; omitted 9.8%
93	17019.47	^{5}I	STATE 92	^{5}I 35.8%; ^{3}K(1) 19.3%; ^{3}L 7.9%; ^{5}G 6.0%; omitted 9.1%
94	17048.51	^{5}I	STATE 93	^{5}I 36.2%; ^{3}K(1) 21.2%; ^{5}F 6.8%; ^{5}G 6.4%; omitted 8.8%
95	17082.19	^{5}F	STATE 94	^{5}F 40.1%; ^{5}G 30.4%; ^{3}P(1) 3.4%; ^{3}D(1) 2.9%; omitted 9.0%
96	17125.89	^{5}G	STATE 95	^{5}G 33.1%; ^{5}F 25.4%; ^{5}S 15.7%; ^{3}D(1) 5.4%; omitted 9.0%
97	17139.55	^{5}F	STATE 96	^{5}F 29.3%; ^{5}G 27.7%; ^{5}I 9.1%; ^{3}F(2) 5.7%; omitted 9.6%
98	17203.90	^{5}G	STATE 97	^{5}G 34.6%; ^{5}F 32.5%; ^{5}I 4.3%; ^{3}F(2) 4.0%; omitted 10.4%
99	17216.95	^{5}G	STATE 98	^{5}G 34.3%; ^{5}F 26.4%; ^{5}I 7.3%; ^{3}F(2) 4.2%; omitted 9.8%
100	17590.02	^{5}G	STATE 99	^{5}G 41.9%; ^{5}F 30.1%; ^{3}F(2) 10.4%; ^{3}D(1) 3.2%; omitted 9.1%

[$PuCl_4(HPyO)_2$]

Table S41. 100 lowest calculated energies (cm^{-1}) of the electronic states in [$PuCl_4(HPyO)_2$] with term symbol and parentage as determined from CASSCF-NEVPT2 calculations using ORCA.

Ion	Pu(4+)	Configuration	$5f^4$	Energy source	NEVPT2

Section	Level	Energy / cm^{-1}	Label	Root(s)/state	Multiplicity/Parentage
Free ion spin-free	1	24.70	^{5}I	roots 0-12	5
	2	8090.50	^{5}S	roots 17	5
	3	8875.89	^{5}F	roots 13-16, 18-20	5
	4	13376.52	^{3}K(1)	roots 0-14	3
	5	13944.33	^{5}G	roots 21-29	5
	6	15739.86	^{3}H(1)	roots 15-25	3
	7	16853.24	^{3}G(1)	roots 26-34	3
	8	18362.92	^{3}D(1)	roots 52, 54, 57-59	3
	9	18365.10	^{3}P(1)	roots 53, 55-56	3
	10	18709.29	^{3}L	roots 35-51	3
	11	20628.47	^{3}F(1)	roots 79-85	3
	12	21746.56	^{1}L(1)	roots 0-16	1
	13	22365.81	^{3}M	roots 60-78	3
	14	22436.00	^{1}D(1)	roots 17-21	1
	15	22565.90	^{3}I(1)	roots 86-98	3
	16	22886.30	^{5}D	roots 30-34	5
	17	24022.37	^{1}G(1)	roots 22-30	1
	18	24445.69	^{3}F(2)	roots 99-105	3
	19	28011.91	^{1}H(1)	roots 44-54	1
	20	28273.20	^{1}D(2)	roots 70-74	1
	21	28907.15	^{1}I(1)	roots 31-43	1
	22	29230.05	^{3}H(2)	roots 106-116	3
	23	29716.95	^{1}K	roots 55-69	1
	24	31656.41	^{3}G(2)	roots 117-125	3
	25	33302.73	^{3}F(3)	roots 126-132	3
	26	35598.08	^{3}K(2)	roots 141-143, 146, 148-158	3
	27	35639.33	^{3}H(3)	roots 133-140, 144-145, 147	3
	28	35806.61	^{1}N	roots 77, 80-85, 87-89, 92, 95-104	1
	29	35875.03	^{1}G(2)	roots 75-76, 78-79, 86, 90-91, 93-94	1
	30	39824.06	^{1}L(2)	roots 105-121	1
	31	40171.39	^{3}I(2)	roots 159-171	3
	32	40861.63	^{3}P(2)	roots 172-174	3
	33	42365.49	^{3}G(3)	roots 175-183	3
	34	44228.05	^{1}I(2)	roots 122-134	1

	35	45742.64	^{1}G(3)	roots 135-143	1
	36	47305.10	^{1}F	roots 144-150	1
	37	47596.38	^{1}D(3)	roots 151-155	1
	38	47780.18	^{3}D(2)	roots 184-188	3
	39	54355.60	^{1}S(1)	roots 156	1
	40	55525.91	^{1}G(4)	roots 157-161, 163, 165-167	1
	41	55585.00	^{1}S(2)	roots 162	1
	42	61640.38	^{1}I(3)	roots 164, 168-175, 177-180	1
	43	57018.37	^{3}F(4)	roots 189-195	3
	44	60296.55	^{3}H(4)	roots 196-206	3
	45	60960.00	^{3}P(3)	roots 207-209	3
	46	72348.65	^{1}H(2)	roots 176, 181-190	1
	47	92320.56	^{1}D(4)	roots 191-195	1
Complex spin-free	1	0.00	^{5}I	roots 0-12	5
	2	7664.20	^{5}F	roots 13-16, 18-20	5
	3	9027.80	^{5}S	roots 17	5
	4	12982.70	^{3}K(1)	roots 0-14	3
	5	13260.90	^{5}G	roots 21-29	5
	6	14458.80	^{3}H(1)	roots 15-25	3
	7	15347.30	^{3}G(1)	roots 26-34	3
	8	17842.80	^{3}L	roots 35-51	3
	9	18567.10	^{3}D(1)	roots 52, 54, 57-59	3
	10	18672.80	^{3}P(1)	roots 53, 55-56	3
	11	19237.80	^{3}M	roots 60-78	3
	12	19658.40	^{5}D	roots 30-34	5
	13	20380.30	^{1}L(1)	roots 0-16	1
	14	21400.70	^{1}D(1)	roots 17-21	1
	15	21796.20	^{1}G(1)	roots 22-30	1
	16	22496.40	^{3}F(1)	roots 79-85	3
	17	23066.60	^{3}I(1)	roots 86-98	3
	18	24035.10	^{3}F(2)	roots 99-105	3
	19	25482.00	^{1}I(1)	roots 31-43	1
	20	26110.70	^{1}H(1)	roots 44-54	1
	21	27788.90	^{3}H(2)	roots 106-116	3
	22	29036.00	^{1}K	roots 55-69	1
	23	29800.50	^{3}G(2)	roots 117-125	3
	24	30477.20	^{1}D(2)	roots 70-74	1
	25	31434.60	^{3}F(3)	roots 126-132	3
	26	32433.10	^{1}G(2)	roots 75-76, 78-79, 86, 90-91, 93-94	1
	27	32554.70	^{3}H(3)	roots 133-140, 144-145, 147	3

	28	32687.60	¹N	roots 77, 80-85, 87-89, 92, 95-104	1
	29	34127.10	³K(2)	roots 141-143, 146, 148-158	3
	30	36131.20	³I(2)	roots 159-171	3
	31	36868.60	¹L(2)	roots 105-121	1
	32	38731.90	³P(2)	roots 172-174	3
	33	39667.60	³G(3)	roots 175-183	3
	34	41187.60	¹I(2)	roots 122-134	1
	35	42415.60	¹G(3)	roots 135-143	1
	36	44051.40	¹F	roots 144-150	1
	37	44517.40	³D(2)	roots 184-188	3
	38	46073.90	¹D(3)	roots 151-155	1
	39	50834.20	¹S(1)	roots 156	1
	40	51296.40	¹G(4)	roots 157-161, 163, 165-167	1
	41	52382.70	³F(4)	roots 189-195	3
	42	52670.90	¹S(2)	roots 162	1
	43	53217.00	¹I(3)	roots 164, 168-175, 177-180	1
	44	54476.60	³H(4)	roots 196-206	3
	45	57944.90	³P(3)	roots 207-209	3
	46	59515.20	¹H(2)	roots 176, 181-190	1
	47	81085.30	¹D(4)	roots 191-195	1
Complex SOC	1	0.00	⁵I	STATE 0	⁵I 79.4%; ³H(1) 15.7%; omitted 3.4%
	2	79.33	⁵I	STATE 1	⁵I 78.8%; ³H(1) 16.2%; omitted 4.1%
	3	252.69	⁵I	STATE 2	⁵I 78.8%; ³H(1) 16.8%; omitted 3.3%
	4	1288.39	⁵I	STATE 3	⁵I 80.5%; ³G(1) 7.7%; ³H(1) 6.3%; omitted 4.3%
	5	1356.43	⁵I	STATE 4	⁵I 81.3%; ³G(1) 8.2%; ³H(1) 4.4%; omitted 4.7%
	6	1494.98	⁵I	STATE 5	⁵I 80.6%; ³G(1) 7.7%; ³H(1) 5.8%; omitted 5.1%
	7	1592.59	⁵I	STATE 6	⁵I 81.6%; ³G(1) 7.7%; ³H(1) 3.4%; omitted 5.6%
	8	1647.56	⁵I	STATE 7	⁵I 81.2%; ³G(1) 8.5%; ³H(1) 4.1%; omitted 4.8%
	9	1705.17	⁵I	STATE 8	⁵I 82.5%; ³G(1) 8.8%; ³L 1.5%; ³K(1) 1.4%; omitted 5.2%
	10	5481.81	⁵I	STATE 9	⁵I 88.9%; ³H(1) 5.0%; ³G(1) 3.0%; omitted 3.2%
	11	5503.02	⁵I	STATE 10	⁵I 88.0%; ³H(1) 4.0%; ³G(1) 4.0%; omitted 3.8%
	12	5545.36	⁵I	STATE 11	⁵I 88.0%; ³G(1) 4.1%; ³H(1) 3.7%; omitted 3.8%

13	5587.49	^{5}I	STATE 12	^{5}I 88.9%; ^{3}H(1) 3.8%; ^{3}G(1) 3.2%; omitted 4.0%
14	5655.67	^{5}I	STATE 13	^{5}I 89.1%; ^{3}H(1) 4.7%; ^{3}G(1) 2.9%; omitted 3.2%
15	5765.81	^{5}I	STATE 14	^{5}I 90.5%; ^{3}G(1) 3.5%; ^{3}H(1) 2.6%; omitted 3.3%
16	5790.77	^{5}I	STATE 15	^{5}I 90.5%; ^{3}G(1) 3.8%; ^{3}H(1) 2.3%; omitted 3.3%
17	5931.11	^{5}I	STATE 16	^{5}I 90.5%; ^{3}G(1) 3.7%; ^{3}H(1) 2.2%; omitted 3.6%
18	6197.35	^{5}I	STATE 17	^{5}I 90.2%; ^{3}G(1) 4.5%; ^{3}H(1) 1.0%; omitted 4.1%
19	6215.55	^{5}I	STATE 18	^{5}I 90.2%; ^{3}G(1) 4.4%; omitted 4.4%
20	6258.40	^{5}I	STATE 19	^{5}I 90.3%; ^{3}G(1) 4.3%; omitted 4.1%
21	9624.39	^{5}I	STATE 20	^{5}I 88.6%; ^{3}K(1) 4.7%; ^{3}H(1) 1.0%; omitted 4.8%
22	9652.18	^{5}I	STATE 21	^{5}I 88.4%; ^{3}K(1) 4.7%; ^{3}G(1) 1.1%; omitted 4.9%
23	9675.99	^{5}I	STATE 22	^{5}I 88.8%; ^{3}K(1) 4.8%; ^{3}H(1) 1.1%; omitted 4.7%
24	9706.85	^{5}I	STATE 23	^{5}I 86.0%; ^{3}K(1) 5.0%; ^{3}G(1) 1.0%; omitted 6.8%
25	9743.20	^{5}I	STATE 24	^{5}I 86.6%; ^{3}K(1) 4.9%; ^{3}G(1) 1.0%; omitted 5.8%
26	9774.22	^{5}I	STATE 25	^{5}I 85.9%; ^{3}K(1) 5.2%; ^{3}G(1) 1.2%; omitted 6.8%
27	9796.01	^{5}I	STATE 26	^{5}I 70.9%; ^{5}F 4.8%; ^{3}K(1) 4.8%; ^{3}L 3.6%; omitted 6.5%
28	9826.28	^{5}I	STATE 27	^{5}I 80.7%; ^{3}K(1) 5.0%; ^{5}F 1.8%; ^{3}G(1) 1.5%; omitted 7.8%
29	9834.36	^{5}I	STATE 28	^{5}I 83.7%; ^{3}K(1) 4.8%; ^{3}G(1) 1.2%; ^{5}F 1.1%; omitted 8.3%
30	10020.43	^{5}I	STATE 29	^{5}I 85.7%; ^{3}K(1) 6.0%; omitted 6.6%
31	10042.55	^{5}I	STATE 30	^{5}I 84.4%; ^{3}K(1) 5.7%; ^{3}G(1) 1.7%; omitted 6.1%
32	10122.13	^{5}I	STATE 31	^{5}I 84.8%; ^{3}K(1) 6.3%; ^{3}G(1) 1.5%; omitted 6.1%
33	10146.45	^{5}I	STATE 32	^{5}I 84.5%; ^{3}K(1) 6.4%; ^{3}G(1) 1.5%; omitted 5.9%
34	10803.47	^{5}F	STATE 33	^{5}F 25.8%; ^{5}I 24.9%; ^{3}L 12.3%; ^{3}F(2) 8.5%; omitted 7.2%
35	11075.91	^{5}F	STATE 34	^{5}F 32.5%; ^{5}I 14.1%; ^{3}F(2) 9.8%; ^{3}D(1) 9.7%; omitted 7.5%
36	12039.34	^{5}F	STATE 35	^{5}F 36.4%; ^{3}F(2) 15.5%; ^{5}G 11.1%; ^{3}P(1) 5.6%; omitted 8.1%
37	12119.93	^{5}F	STATE 36	^{5}F 30.7%; ^{3}F(2) 15.5%; ^{5}G 11.2%; ^{3}P(1) 6.2%; omitted 7.9%
38	12412.54	^{5}F	STATE 37	^{5}F 24.3%; ^{3}F(2) 13.5%; ^{5}S 11.9%; ^{5}G 10.2%; omitted 7.3%

39	12842.00	^{5}I	STATE 38	^{5}I 69.8%; ^{3}K(1) 16.9%; ^{5}F 1.8%; omitted 7.0%
40	12909.36	^{5}I	STATE 39	^{5}I 69.5%; ^{3}K(1) 17.0%; ^{5}F 1.9%; ^{3}M 1.0%; omitted 6.6%
41	12921.21	^{5}I	STATE 40	^{5}I 57.1%; ^{5}F 15.8%; ^{3}K(1) 12.1%; ^{3}L 3.7%; omitted 5.4%
42	12936.63	^{5}I	STATE 41	^{5}I 62.4%; ^{3}K(1) 13.1%; ^{5}F 12.1%; ^{5}S 1.8%; omitted 5.9%
43	12938.80	^{5}I	STATE 42	^{5}I 64.6%; ^{3}K(1) 13.7%; ^{5}F 9.6%; ^{5}S 1.9%; omitted 6.6%
44	13002.77	^{5}I	STATE 43	^{5}I 59.9%; ^{3}K(1) 18.2%; ^{3}H(1) 5.6%; ^{5}F 4.0%; omitted 8.0%
45	13014.04	^{5}I	STATE 44	^{5}I 60.1%; ^{3}K(1) 18.0%; ^{3}H(1) 4.0%; ^{5}F 3.5%; omitted 8.0%
46	13042.30	^{5}I	STATE 45	^{5}I 63.7%; ^{3}K(1) 18.9%; ^{3}H(1) 2.6%; ^{3}G(1) 2.3%; omitted 6.5%
47	13170.20	^{5}I	STATE 46	^{5}I 61.0%; ^{3}K(1) 15.3%; ^{5}F 11.1%; ^{3}L 1.4%; omitted 6.5%
48	13220.04	^{5}I	STATE 47	^{5}I 53.8%; ^{5}F 15.2%; ^{3}K(1) 13.2%; ^{5}S 4.0%; omitted 6.3%
49	13222.34	^{5}I	STATE 48	^{5}I 46.7%; ^{5}F 19.5%; ^{3}K(1) 11.8%; ^{5}S 6.8%; omitted 7.3%
50	13268.09	^{5}I	STATE 49	^{5}I 65.5%; ^{3}K(1) 16.7%; ^{5}F 6.7%; ^{3}L 1.6%; omitted 7.3%
51	13414.26	^{5}I	STATE 50	^{5}I 40.3%; ^{5}F 32.0%; ^{3}K(1) 10.2%; ^{3}L 3.9%; omitted 7.0%
52	13448.63	^{5}I	STATE 51	^{5}I 53.1%; ^{3}K(1) 13.7%; ^{5}F 10.9%; ^{3}H(1) 4.1%; omitted 8.2%
53	13467.87	^{5}I	STATE 52	^{5}I 49.8%; ^{5}F 13.8%; ^{3}K(1) 12.5%; ^{3}H(1) 4.8%; omitted 7.9%
54	13564.42	^{5}I	STATE 53	^{5}I 32.0%; ^{5}F 29.2%; ^{3}K(1) 8.6%; ^{5}S 8.1%; omitted 7.0%
55	13592.82	^{5}I	STATE 54	^{5}I 51.5%; ^{3}K(1) 13.5%; ^{5}F 9.1%; ^{3}H(1) 5.7%; omitted 9.6%
56	13605.64	^{5}I	STATE 55	^{5}I 40.6%; ^{5}F 19.8%; ^{3}K(1) 10.6%; ^{5}S 6.8%; omitted 7.5%
57	13767.11	^{5}I	STATE 56	^{5}I 40.2%; ^{3}H(1) 13.2%; ^{5}F 12.1%; ^{3}G(1) 5.0%; omitted 7.5%
58	13819.43	^{5}I	STATE 57	^{5}I 31.5%; ^{3}H(1) 17.5%; ^{5}F 11.1%; ^{5}S 5.6%; omitted 9.0%
59	13936.99	^{5}I	STATE 58	^{5}I 25.2%; ^{3}H(1) 19.6%; ^{5}F 13.7%; ^{5}G 4.9%; omitted 7.3%
60	14559.43	^{5}I	STATE 59	^{5}I 18.2%; ^{5}F 15.4%; ^{3}H(1) 12.0%; ^{3}G(1) 11.5%; omitted 8.7%
61	14749.61	^{5}F	STATE 60	^{5}F 22.1%; ^{5}I 16.1%; ^{3}G(1) 13.1%; ^{3}L 9.2%; omitted 8.6%
62	14905.71	^{5}F	STATE 61	^{5}F 41.7%; ^{5}G 14.9%; ^{5}I 10.9%; ^{3}K(1) 4.9%; omitted 8.7%
63	14937.31	^{5}F	STATE 62	^{5}F 33.7%; ^{5}I 13.0%; ^{5}G 12.5%; ^{3}K(1) 6.0%; omitted 8.4%

64	14963.00	^{5}F	STATE 63	^{5}F 41.5%; ^{5}G 13.9%; ^{5}I 10.5%; ^{3}D(1) 5.2%; omitted 10.5%
65	15002.06	^{5}F	STATE 64	^{5}F 25.6%; ^{5}I 13.8%; ^{5}G 9.4%; ^{3}G(1) 8.1%; omitted 10.6%
66	15157.18	^{5}I	STATE 65	^{5}I 17.4%; ^{5}F 15.8%; ^{3}G(1) 9.6%; ^{3}L 8.6%; omitted 9.8%
67	15216.32	^{5}I	STATE 66	^{5}I 17.8%; ^{5}F 16.8%; ^{3}H(1) 9.0%; ^{3}G(1) 8.5%; omitted 11.1%
68	15417.95	^{5}I	STATE 67	^{5}I 28.7%; ^{3}K(1) 15.7%; ^{5}F 14.6%; ^{3}H(1) 7.9%; omitted 7.9%
69	15522.45	^{5}I	STATE 68	^{5}I 43.1%; ^{3}K(1) 20.5%; ^{5}F 17.6%; ^{1}L(1) 2.9%; omitted 7.3%
70	15530.68	^{5}I	STATE 69	^{5}I 38.7%; ^{5}F 22.5%; ^{3}K(1) 18.2%; ^{1}L(1) 2.8%; omitted 8.2%
71	15539.56	^{5}I	STATE 70	^{5}I 39.1%; ^{5}F 18.0%; ^{3}K(1) 17.6%; ^{5}S 3.4%; omitted 8.3%
72	15546.56	^{5}I	STATE 71	^{5}I 41.2%; ^{3}K(1) 19.0%; ^{5}F 16.8%; ^{1}L(1) 2.9%; omitted 8.6%
73	15565.01	^{5}F	STATE 72	^{5}F 31.4%; ^{5}I 25.7%; ^{3}K(1) 10.8%; ^{5}S 7.8%; omitted 8.6%
74	15588.99	^{5}I	STATE 73	^{5}I 37.3%; ^{3}K(1) 17.4%; ^{5}F 10.7%; ^{5}S 7.9%; omitted 9.7%
75	15630.24	^{5}F	STATE 74	^{5}F 34.3%; ^{5}I 24.3%; ^{3}K(1) 9.4%; ^{5}S 4.3%; omitted 9.6%
76	15642.17	^{5}F	STATE 75	^{5}F 35.6%; ^{5}I 26.6%; ^{3}K(1) 11.0%; ^{3}F(2) 3.2%; omitted 9.7%
77	15739.90	^{5}I	STATE 76	^{5}I 38.1%; ^{3}K(1) 16.0%; ^{5}F 15.9%; ^{5}S 4.0%; omitted 10.1%
78	15762.32	^{5}I	STATE 77	^{5}I 31.8%; ^{5}F 21.1%; ^{3}K(1) 14.0%; ^{5}G 7.0%; omitted 8.6%
79	15829.76	^{5}I	STATE 78	^{5}I 36.3%; ^{5}F 19.7%; ^{3}K(1) 15.9%; ^{3}L 4.2%; omitted 8.6%
80	15888.48	^{5}I	STATE 79	^{5}I 30.3%; ^{5}F 24.2%; ^{3}K(1) 12.9%; ^{5}G 7.9%; omitted 7.9%
81	16003.90	^{5}F	STATE 80	^{5}F 32.6%; ^{5}I 22.3%; ^{3}K(1) 10.6%; ^{5}G 8.8%; omitted 9.2%
82	16012.15	^{5}F	STATE 81	^{5}F 26.3%; ^{5}I 22.9%; ^{3}K(1) 12.3%; ^{5}S 8.1%; omitted 9.8%
83	16100.77	^{5}I	STATE 82	^{5}I 28.2%; ^{5}F 24.4%; ^{3}K(1) 13.7%; ^{3}L 8.1%; omitted 8.4%
84	16167.69	^{5}F	STATE 83	^{5}F 26.5%; ^{3}H(1) 15.8%; ^{5}G 14.1%; ^{5}I 9.5%; omitted 9.3%
85	16174.10	^{5}I	STATE 84	^{5}I 23.0%; ^{5}F 22.2%; ^{3}K(1) 11.2%; ^{3}H(1) 7.1%; omitted 9.3%
86	16276.60	^{5}I	STATE 85	^{5}I 30.3%; ^{5}F 25.9%; ^{3}K(1) 17.7%; ^{3}L 4.1%; omitted 8.2%
87	16314.15	^{5}I	STATE 86	^{5}I 27.7%; ^{5}F 23.7%; ^{3}K(1) 16.0%; ^{5}S 6.1%; omitted 9.6%

88	16325.67	^{5}I	STATE 87	^{5}I 26.9%; ^{5}F 18.9%; ^{3}K(1) 16.0%; ^{5}S 11.4%; omitted 9.4%
89	16909.41	^{5}I	STATE 88	^{5}I 33.6%; ^{3}K(1) 17.5%; ^{5}F 12.0%; ^{5}G 10.4%; omitted 9.6%
90	16933.08	^{5}I	STATE 89	^{5}I 35.4%; ^{3}K(1) 19.6%; ^{5}F 8.4%; ^{5}G 8.0%; omitted 9.7%
91	16971.61	^{5}I	STATE 90	^{5}I 45.9%; ^{3}K(1) 23.6%; ^{3}L 5.3%; ^{5}G 3.3%; omitted 8.5%
92	17018.39	^{5}I	STATE 91	^{5}I 32.7%; ^{3}K(1) 18.0%; ^{5}G 10.9%; ^{5}F 8.7%; omitted 11.4%
93	17042.77	^{5}I	STATE 92	^{5}I 37.6%; ^{3}K(1) 19.8%; ^{3}L 7.9%; ^{5}G 5.6%; omitted 10.7%
94	17063.50	^{5}I	STATE 93	^{5}I 31.4%; ^{3}K(1) 16.9%; ^{5}F 9.4%; ^{5}G 8.9%; omitted 9.8%
95	17098.52	^{5}G	STATE 94	^{5}G 32.2%; ^{5}F 31.7%; ^{5}S 6.2%; ^{3}D(1) 4.1%; omitted 10.5%
96	17115.59	^{5}G	STATE 95	^{5}G 30.6%; ^{5}F 30.3%; ^{5}I 6.0%; ^{5}S 5.4%; omitted 10.6%
97	17165.55	^{5}G	STATE 96	^{5}G 32.3%; ^{5}F 27.4%; ^{5}I 8.8%; ^{3}F(2) 5.3%; omitted 9.4%
98	17189.60	^{5}G	STATE 97	^{5}G 33.0%; ^{5}F 30.8%; ^{3}D(1) 4.8%; ^{5}S 4.8%; omitted 10.9%
99	17230.60	^{5}G	STATE 98	^{5}G 39.4%; ^{5}F 16.1%; ^{5}I 11.0%; ^{3}K(1) 5.9%; omitted 7.4%
100	17493.63	^{5}G	STATE 99	^{5}G 39.1%; ^{5}F 32.6%; ^{3}F(2) 11.3%; ^{3}H(1) 2.4%; omitted 7.9%

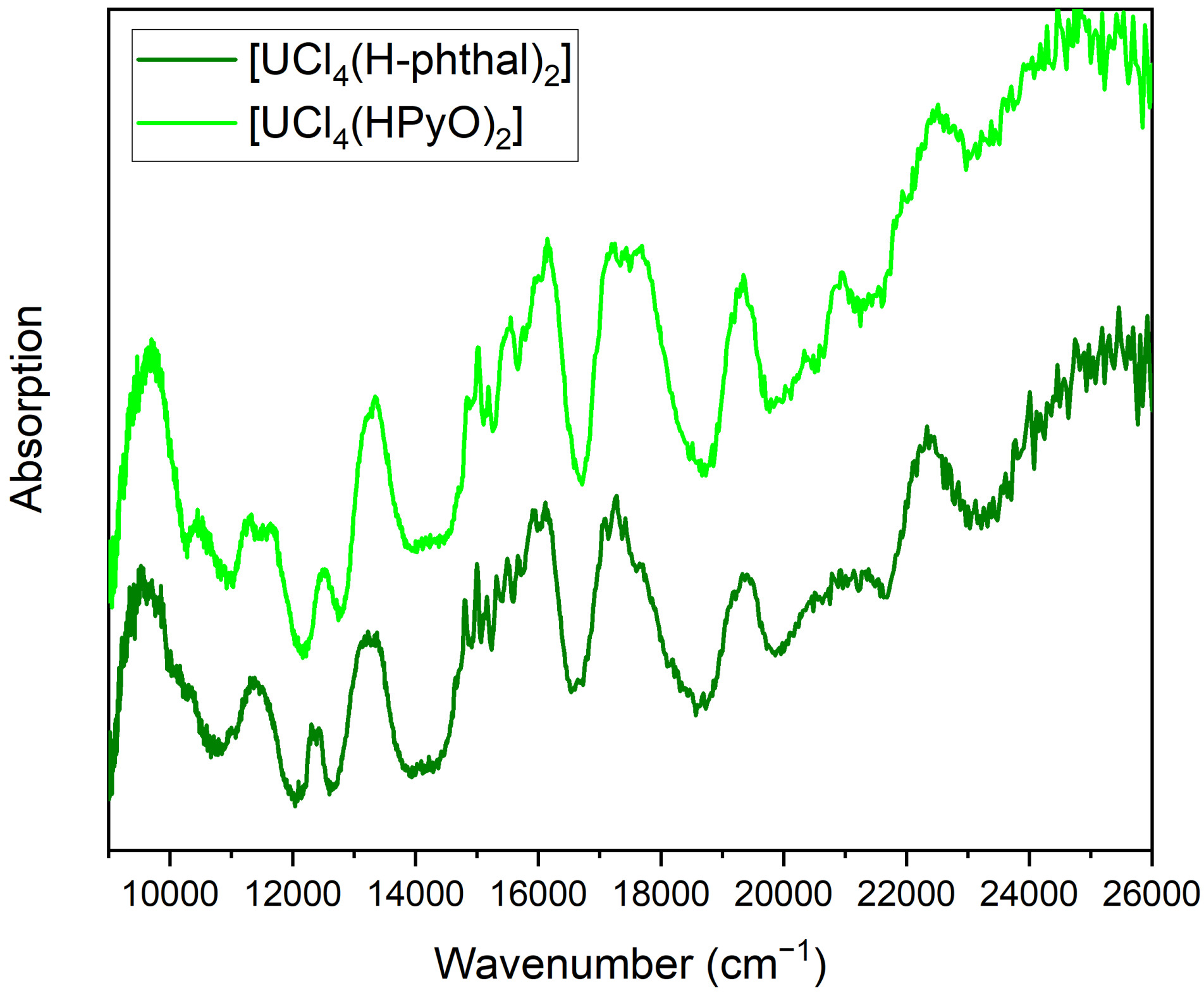

Figure S65. Solid state Vis-NIR spectra of [UCl_4(H-phthal)$_2$] and [UCl_4(HPyO)$_2$].

Literature

(1) Morss, L.; Edelstein, N.; Fuger, J. *The Chemistry of the Actinide and Transactinide Elements*; 2006. DOI: 10.1007/1-4020-3598-5.

(2) Patel, D.; Wooles, A. J.; Hashem, E.; Omorodion, H.; Baker, R. J.; Liddle, S. T. Comments on reactions of oxide derivatives of uranium with hexachloropropene to give UCl_4. *New Journal of Chemistry* **2015**, *39* (10), 7559-7562. DOI: 10.1039/C5NJ00476D.

(3) Reilly, S. D.; Brown, J. L.; Scott, B. L.; Gaunt, A. J. Synthesis and characterization of $NpCl_4(DME)_2$ and $PuCl_4(DME)_2$ neutral transuranic An(iv) starting materials. *Dalton Transactions* **2014**, *43* (4), 1498-1501. DOI: 10.1039/C3DT53058B.

(4) *APEX4*; Bruker AXS Inc.: Madison, Wisconsin, USA, 2021.

(5) Blessing, R. An empirical correction for absorption anisotropy. *Acta Crystallographica Section A* **1995**, *51* (1), 33-38. DOI: doi:10.1107/S0108767394005726.

(6) Sheldrick, G. *SHELXT* - Integrated space-group and crystal-structure determination. *Acta Crystallographica Section A* **2015**, *71* (1), 3-8. DOI: doi:10.1107/S2053273314026370.

(7) Sheldrick, G. Crystal structure refinement with *SHELXL*. *Acta Crystallographica Section C* **2015**, *71* (1), 3-8. DOI: doi:10.1107/S2053229614024218.

(8) Farrugia, L. WinGX and ORTEP for Windows: an update. *Journal of Applied Crystallography* **2012**, *45* (4), 849-854. DOI: doi:10.1107/S0021889812029111.

(9) Scheinost, A. C.; Claussner, J.; Exner, J.; Feig, M.; Findeisen, S.; Hennig, C.; Kvashnina, K. O.; Naudet, D.; Prieur, D.; Rossberg, A.; et al. ROBL-II at ESRF: a synchrotron toolbox for actinide research. *Journal of Synchrotron Radiation* **2021**, *28* (1), 333-349. DOI: doi:10.1107/S1600577520014265.

(10) Kvashnina, K. O.; Scheinost, A. C. A Johann-type X-ray emission spectrometer at the Rossendorf beamline. *Journal of Synchrotron Radiation* **2016**, *23* (3), 836-841. DOI: doi:10.1107/S1600577516004483.

(11) Coak, M. J.; Liu, C.; Jarvis, D. M.; Park, S.; Cliffe, M. J.; Goddard, P. A. SquidLab—A user-friendly program for background subtraction and fitting of magnetization data. *Review of Scientific Instruments* **2020**, *91* (2). DOI: 10.1063/1.5137820 (acccessed 1/19/2026).

(12) Cordero, B.; Gómez, V.; Platero-Prats, A. E.; Revés, M.; Echeverría, J.; Cremades, E.; Barragán, F.; Alvarez, S. Covalent radii revisited. *Dalton Transactions* **2008**, (21), 2832-2838, 10.1039/B801115J. DOI: 10.1039/B801115J.

(13) Lu, T.; Chen, F. Multiwfn: A Multifunctional Wavefunction Analyzer. *Journal of Computational Chemistry* **2012**, *33* (5), 580-592. DOI: https://doi.org/10.1002/jcc.22885.

(14) Lueken, H. Praktische Anleitung zur Messung und Interpretation magnetischer Eigenschaften. *Angewandte Chemie* **2006**, *118* (47), 8233-8240. DOI: https://doi.org/10.1002/ange.200603275.

(15) Lueken, H. *Magnetochemie*; Vieweg+Teubner Verlag Wiesbaden, 1999. DOI: https://doi.org/10.1007/978-3-322-80118-0.

(16) Bain, G. A.; Berry, J. F. Diamagnetic Corrections and Pascal's Constants. *Journal of Chemical Education* **2008**, *85* (4), 532. DOI: 10.1021/ed085p532.

(17) Grödler, D.; Weidemann, M. L.; Lichtenberg, A.; Greven, T.; Nickstadt, R.; Haydo, M.; Wickleder, M.; Klein, A.; Johrendt, D.; Mathur, S.; et al. Heterobimetallic uranyl(VI) alkoxides of lanthanoids: formation through simple ligand exchange. *Chemical Communications* **2022**, *58* (6), 835-838. DOI: 10.1039/D1CC05444A.

(18) van Leusen, J.; Speldrich, M.; Schilder, H.; Kögerler, P. Comprehensive insight into molecular magnetism via CONDON: Full vs. effective models. *Coordination Chemistry Reviews* **2015**, *289-290*, 137-148. DOI: https://doi.org/10.1016/j.ccr.2014.10.011.

(19) Speldrich, M.; van Leusen, J.; Kögerler, P. CONDON 3.0: An Updated Software Package for Magnetochemical Analysis-All the Way to Polynuclear Actinide Complexes. *Journal of Computational Chemistry* **2018**, *39* (25), 2133-2145. DOI: https://doi.org/10.1002/jcc.25389.

(20) Condon, E. U.; Shortley, G. H. *The Theory of Atomic Spectra*; Cambridge University Press, 1970.

(21) *SOC parentage python script and database*; 2026. http://doi.org/10.14278/rodare.4733 (accessed 06/26/2026).
